# A protective role of genetically predicted sex hormone-binding globulin on stroke

**DOI:** 10.1016/j.heliyon.2024.e28556

**Published:** 2024-03-30

**Authors:** Qiang He, Wenjing Wang, Yang Xiong, Chuanyuan Tao, Lu Ma, Jinming Han, Chao You

**Affiliations:** a Department of Neurosurgery, West China Hospital, Sichuan University, 37 Guoxue Lane, Wuhou District, Chengdu, 610041, Sichuan, China; b Department of Pharmacy, State Key Laboratory of Biotherapy, West China Hospital, Sichuan University, Chengdu, China; c Department of Urology, West China Hospital, Sichuan University, Chengdu, China; d Department of Neurology, Xuanwu Hospital, Capital Medical University, Beijing, China

**Keywords:** Sex hormone-binding globulin, Stroke, Stroke subtypes, Mendelian randomization, Causal association

## Abstract

**Introduction:**

The role of sex hormone-binding globulin (SHBG) on stroke has been investigated in several observational studies. To provide the causal estimates of SHBG on stroke and its subtypes, bi-directional and multivariable Mendelian randomization (MR) analyses are performed.

**Methods:**

The genetic instruments of SHBG were obtained from the UK Biobank. Outcome datasets for stroke and its subtypes were taken from the MEGASTROKE Consortium. The main analysis used in this study is the inverse variance weighting, complemented by other sensitivity approaches to verify the conformity of findings.

**Results:**

We found that the risk of stroke grew by 13% (odd ratio [OR] = 0.87, 95% confidence interval [CI] = 0.79–0.95, P = 0.0041) and the risk of ischemic stroke grew by 15% (OR = 0.85, 95%CI = 0.77–0.95, P = 0.0038) caused by genetically predicted SHBG. The causal association remains robust in the reverse MR and multivariable MR analyses for stroke (reverse MR: all *P* > 0.01 for the IVW method; MVMR: OR = 0.72, 95%CI = 0.59–0.87, *P* = 0.0011) and ischemic stroke (reverse MR: all *P* > 0.01 for IVW; MVMR: OR = 0.70, 95%CI = 0.56–0.86, *P* = 0.0007).

**Conclusion:**

Our MR study provides novel evidence that SHBG has an inverse association with stroke and ischemic stroke, exerting protective effects on stroke.

## 1 Introduction

As the second leading cause of death in the world, stroke is estimated to cause more than 6.5 million deaths annually. Long-term disability in survivors poses a heavy burden on global health [1]. Previous basic research and clinical trials have made tremendous breakthroughs in the treatment of stroke. However, the disease burden caused by stroke is still heavy [2]. It is urgent to explore novel treatments and effective preventive strategies for stroke. Apartment from conventional risk factors like hypertension and diabetes, the identification of new risk factors and protective factors is needed.

Over 75% of U.S. Food and Drug Administration (FDA) approved drugs are targeted proteins. Proteins in the plasma actively take part in a variety of pathological processes [[3], [4], [5]]. Stroke is particularly associated with plasma proteins due to its close relationship with blood vessels. However, previous studies suggested conflicting conclusions regarding the association between estradiol and the risk of stroke [[6], [7], [8], [9], [10], [11], [12], [13], [14], [15]]Therefore, the role of sex hormones and their related proteins in stroke needs to be further explored.

Sex hormones binding-globulin (SHBG) is a protein regulating the level of bioavailable testosterone and estradiol [9,12,[16], [17], [18], [19], [20], [21]]. SHBG may play a protective role by decreasing the risk of vascular diseases [22,23]. Potential mechanism can be attributed to the regulation of testosterone and estradiol, as well as independent effects of sex steroids [24].

Evidence regarding the role of SHBG in the pathogenesis of stroke are limited. One previous study from the Women's Health Initiative supported an inverse association between serum SHBG levels and ischemic stroke (IS) risk [20]. However, potential selection bias in previous studies should not be ignored and may have effects on the interpretation of results. Moreover, the causal association between SHBG and stroke cannot be concluded based on previous studies. Mendelian randomization (MR) analysis can overcome confounders, and utilize genetic variants as instrumental variables (IVs) to test the causal association between exposures and outcomes [25]. Alleles are randomly assorted during the gestation, equalizing confounding factors [26]. In the present study, bi-directional and multivariable MR analyses (MVMR) were conducted to explore the causal relationship between SHBG and stroke (and its subtypes).

## 2 Methods

### 2.1 Data source

The exposure trait SHBG was taken from approximately 500,000 participants of European participants in the UK Biobank (UKBB). In this study, a total of 34 biomarkers were measured. The level of SHBG was measured by the two-step sandwich immunoassay analysis from the blood sample collected at the initial visit (including 425,097 participants of European ancestry). All participants provided written informed consent, and the UKBB study was approved by the National Research Ethics Service Committee North West-Haydock. Further detailed information is available in the original article [18].

In the MEGASTROKE consortium, the summary-level statistics of stroke (its subtypes) in European individuals were obtained [27], consisting of stroke (67,162 patients), IS (60,341 patients), large artery stroke (LAS, 6688 patients), cardioembolic stroke (CES, 9006 patients) and small vessel stroke (SVS, 11,710 patients). According to the stroke definition from World Health Organization (WHO), patients are diagnosed with the symptoms of rapidly displaying symptoms of focal or global cerebral abnormity exceeding 24 h or leading to death without obvious condition other than that of artery source. Based on the Trial of Org 10,172 in Acute Stroke Treatment (TOAST) criteria, IS can be divided into LAS, CES, and SVS [28].

### 2.2 Genetic instrument selection

To obtain independent single nucleotide polymorphisms (SNPs) of SHBG, statistical significance threshold is defined at a genome-wide significance level (*P* < 5 × 10^−8^). Then, the linkage disequilibrium (LD) r^2^ < 0.01 at a 5000 kb window size is chosen referring tothe 1000 Genomes European reference panel. Moreover, we also calculate the F-statistics to represent genetic instruments (Table 1). The formula is F-statistics=(Bets/Se) [2]. Generally, when the F-statistics is less than 10, it is considered as weak IVs, which then are pruned. In this step, the F-statistics of all SNPs exceeds 10. Furthermore, the MR pleiotropy Residual Sum and Outlier (MR-PRESSO) method is applied to explore the significant horizontal pleiotropicSNPs. No IVs in this step are deleted, suggesting the absence of pleiotropy [29]. In MVMR analysis, same genetic instrument selection standard has been performed.

**Table 1:** The R^2^ and F-statistics for the genetic instruments in the MR and reverse MR analyses.

Exposure	Outcome	No. SNP	R [2]	F-statistic
MR				
**SHBG**	Stroke	580	18.49%	121.87
**SHBG**	IS	585	18.60%	121.67
**SHBG**	LAS	591	18.71%	121.34
**SHBG**	CES	589	18.79%	122.31
**SHBG**	SVS	590	18.69%	121.50
Re-MR				
**Stroke**	SHBG	6	0.07%	41.83
**IS**	SHBG	8	0.60%	40.95
**LAS**	SHBG	3	0.09%	37.32
**CES**	SHBG	4	0.14%	74.29
**SVS**	SHBG	–	–	–

SNP: single nucleotide polymorphism; MR: mendelian randomization; SHBG, sex hormone binding globulin; IS ischemic stroke; LAS: large artery stroke; CES: cardioembolic stroke; SVS: small vessel stroke.

### 2.3 Statistical analysis

In our MR analysis, we chose the random effect inverse variance weighting (IVW) approach as the primary method to illuminate the causal relationship of SHBG on stroke and its subtypes. IVW can generate an overall causal effect of SHBG on stroke by merging the wald ratios of every IV into overall causal estimate in the absence of horizontal pleiotropy [30]. The statistical significance is the corrected *P* < 0.01(0.05/5 = 0.01) based on Bonferroni correction. All MR analyses were undergone in the R software (Version 4.1.2, Foundation for Statistical Computing, Vienna, Austria). The R packages “TwoSampleMR”, MR-PRESSO, “mr.raps”, and “frostplot” were used in this study.

### 2.4 Sensitivity analysis

Five methods, including MR-Egger, MR-PRESSO, Maximum likelihood, and MR robust adjusted profile score (MR-RAPS) methods were used to explore the potential pleiotropy and heterogeneity in the sensitivity analysis. The value of the intercept term was used to explain the directional pleiotropy in the MR-Egger analysis. MR-Egger provides a causal effect even if all IVs are invalid [31]. A more robust causal estimate can be produced using MR-PRESSSO method after undergoing detecting horizontal pleiotropy and correcting significant outliers [32]. The direct maximization of likelihood was used to yield the causal effect, and further provide a linear relationship between exposures and outcomes in Maximum likelihood. In MR-RAPS analysis, a robust estimate can be obtained in the condition of normal school of pleiotropic influences and even weak IVs and organized and idiosyncratic horizontal pleiotropy are not noted. The heterogeneity of all IVs was analyzed in Cochran's *Q* method. Relevant SNPs were detected using the leave-one-out approach to evaluate the causal links and verify the robustness of causal estimation [33].

## 3 Results

### 3.1 Genetic association of SHBG on stroke (its subtypes) using single-variable Mendelian randomization (SVMR)

**Fig. 1:**
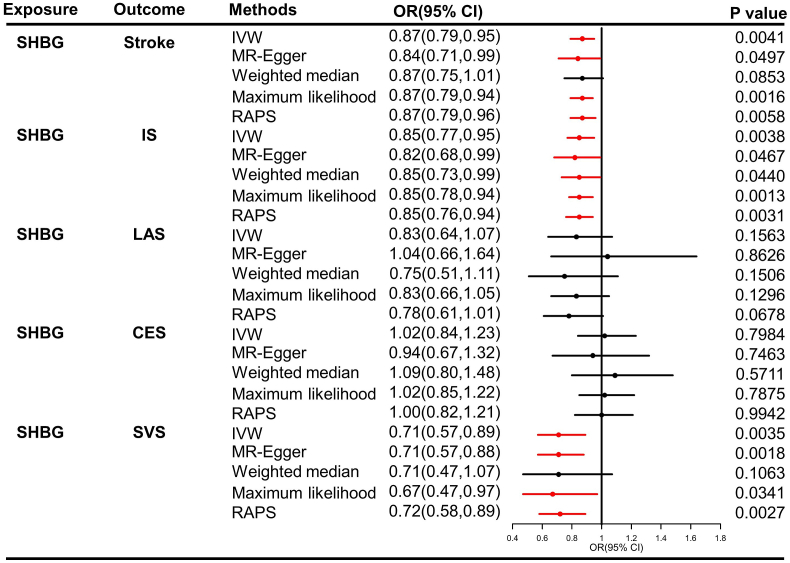
Causal effect estimates of SHBG on stroke and its subtypes in SVMR. SHBG, sex hormone-binding globulin; OR, odds ratio; CI, confidence interval; IVW, inverse variance weighted method; RAPS, robust adjusted profile score; MR, Mendelian randomization; SVMR, single-variable Mendelian randomization. The red line means the threshold of *P* < 0.01. (For interpretation of the references to colour in this figure legend, the reader is referred to the Web version of this article.)

**Fig. 2:**
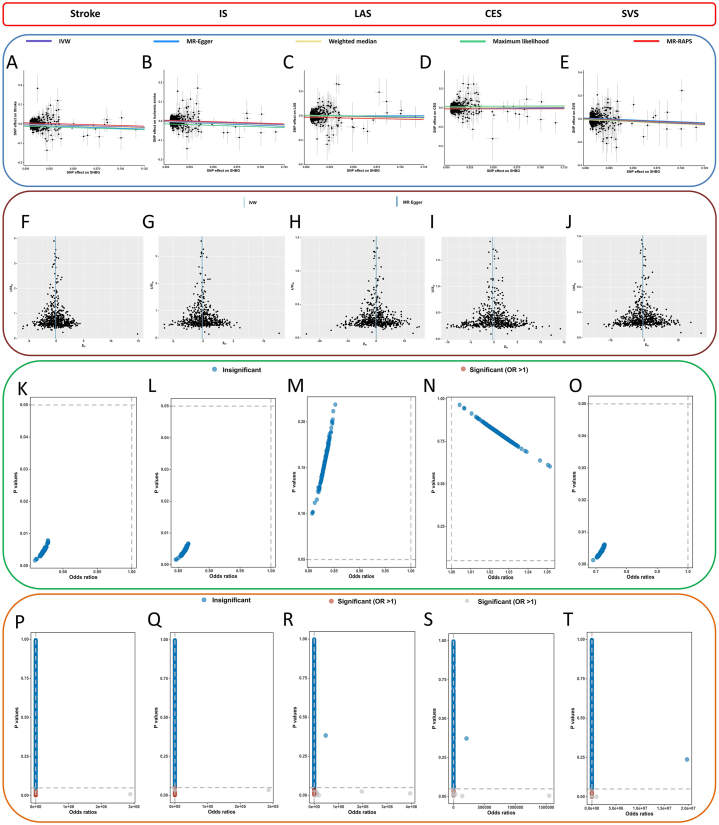
A–E, The scatter plots of the association between genetically predicted SHBG on stroke and its subtypes in SVMR. F-J, The funnel plots of the association between genetically predicted SHBG on stroke and its subtypes in SVMR. K–O, The leave-one-out analysis of the association between genetically SHBG on stroke and stroke and its subtypes in SVMR. P-T, The frost plots of the association between genetically SHBG on stroke and its subtypes in SVMR.

SNPs used as IVs for SHBG in MR analysis was presented in Table S1. In Fig. 1, genetically predicted SHBG causally increases the stroke risk by 13%, 15%, and 29% decrease in stroke (odds ratio [OR] = 0.87, 95% confidence interval [CI] = 0.79–0.95, *P* = 0.0041), IS (OR = 0.85, 95%CI = 0.77–0.95, *P* = 0.0038) and SVS (OR = 0.71, 95%CI = 0.57–0.89, *P* = 0.0035) risk, respectively. The causal associations remain stable in the results of Maximum likelihood and MR-RAPS (all *P* < 0.01). Our results demonstrated that the effect of IVs on stroke, IS and SVS are reduced with the growth of SNPs’ effects on SHBG (Fig. 2A, B, 2E). In the contrast, no causal association of SHBG was detected in LAS and CES (all *P* > 0.01, Fig. 1). Our results do not support the causal effects of SHBG on LAS and CES (Fig. 2C–D).

Sensitivity analysis showed that no obvious signs of pleiotropy for stroke and its subtypes (MR-Egger: all *P* > 0.05; MR-PRESSO global test: all *P* > 0.05), as shown in Table 2. The heterogeneity was detected in Cochran's *Q* statistics (*P* < 0.05), and the random effects IVW method was further applied to estimate the causal effect between SHBG and its stroke subtypes. Our results indicated that no significant outliers are detected (Fig. 2F–J). Additionally, the positive association remains robust in the leave-one-out analysis, suggesting that no influential IVs are found (Fig. 2K–O, Table S2). The effect estimate of each SHBG's SNP on stroke and its subtypes was displayed in Fig. 2P–T.

**Table 2:** Results of heterogeneity and pleiotropy tests in SVMR.

Exposure	Outcome	Methods	Egger_intercept	*P*-Egger_intercept	Cochran's	Cochran's *P*
**SHBG**	Stroke	IVW			708.88	0.0001
		MR-Egger	0.0004	0.6701	708.66	0.0001
**SHBG**	IS	IVW			730.05	3.43e-05
		MR-Egger	0.0004	0.6522	729.79	3.13e-05
**SHBG**	LAS	IVW			590	7.77e-05
		MR-Egger	−0.0030	0.2459	589	8.22e-05
**SHBG**	CES	IVW			588	0.0173
		MR-Egger	0.0019	0.5732	587	0.0165
**SHBG**	SVS	IVW			671.28	0.0103
		MR-Egger	−4.31e-05	0.9851	671.28	0.0095

SHBG, sex hormone binding globulin; SVMR, single-variable Mendelian randomization; IS ischemic stroke; LAS: large artery stroke; CES: cardioembolic stroke; SVS: small vessel stroke; IVW, inverse variance weighted method.

### 3.2 Causal effects of stroke and its subtypes on SHBG in SVMR

**Fig. 3:**
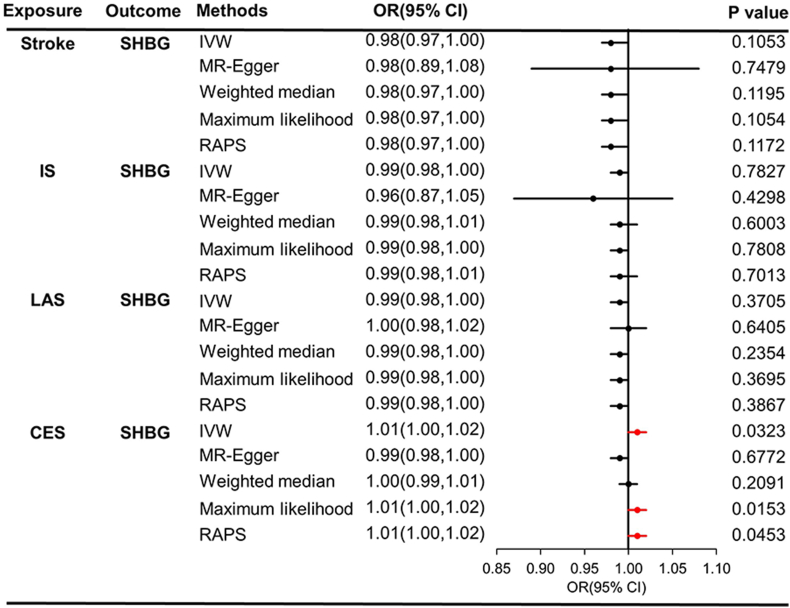
Causal effect estimates of stroke and its subtypes on SHBG in reverse MR.

**Fig. 4:**
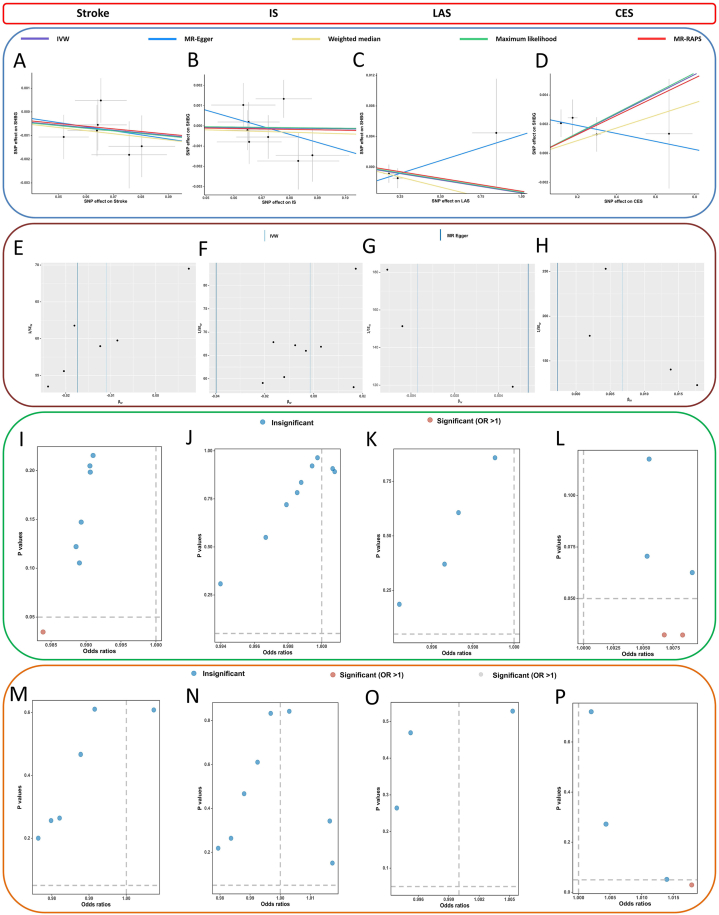
A–D, The scatter plots of the association between genetically predicted stroke, IS, LAS, and CES on SHBG in reverse SVMR. E-H, the funnel plots of the association between genetically predicted SHBG on stroke, IS, LAS, and CES in SVMR. I-L, The leave-one-out analysis of the association between genetically SHBG on stroke, IS, LAS, and CES in SVMR. M − P, the frost plots of the association between genetically SHBG on stroke, IS, LAS, and CES in SVMR.

SNPs used as IVs for stroke and its subtypes in reverse MR analysis was displayed in Tables S3–S6. In the reverse MR analysis, we found that stroke, IS, LAS, and CES do not significantly increase the level of SHBG (all *P* > 0.01), as shown in Fig. 3. Furthermore, no obvious causal effects of stroke, IS, LAS, and CES on SHBG are detected (Fig. 4A–D). No obvious signs of heterogeneity were observed based on Cochran's *Q* test (all *P* > 0.05) as summarized in Table 3. Additionally, no IVs were identified in both MR-Egger regression and MR-PRESSO analysis (all *P* > 0.05, Table 3). No significant outliers were observed in the funnel plots (Fig. 4E–H). We also found that no causal association is detected in the leave-one-out analysis (Fig. 4I–L). The causal effects of every SHBG's SNP on stroke and its subtypes was displayed in Fig. 4M–P.

**Table 3:** Results of heterogeneity and pleiotropy tests in reverse SVMR.

Exposure	Outcome	Methods	Egger_intercept	*P*-Egger_intercept	Cochran's	Cochran's *P*
**Stroke**	SHBG	IVW			5	0.6291
		MR-Egger	0.0032	0.9049	4	0.7608
**IS**	SHBG	IVW			7	0.4809
		MR-Egger	0.0027	0.4433	6	0.4409
**LAS**	SHBG	IVW			2	0.5050
		MR-Egger	0.0024	0.4933	1	0.5692
**CES**	SHBG	IVW			3	0.8775
		MR-Egger	0.0024	0.1963	2	0.2716
**SVS**	SHBG	IVW	–	–	–	–
		MR-Egger			–	–

SHBG, sex hormone binding globulin; SVMR, single-variable Mendelian randomization; IS ischemic stroke; LAS: large artery stroke; CES: cardioembolic stroke; SVS: small vessel stroke; IVW, inverse variance weighted method.

### 3.3 Causal effect estimates of SHBG on stroke and its subtypes in MVMR

**Fig. 5:**
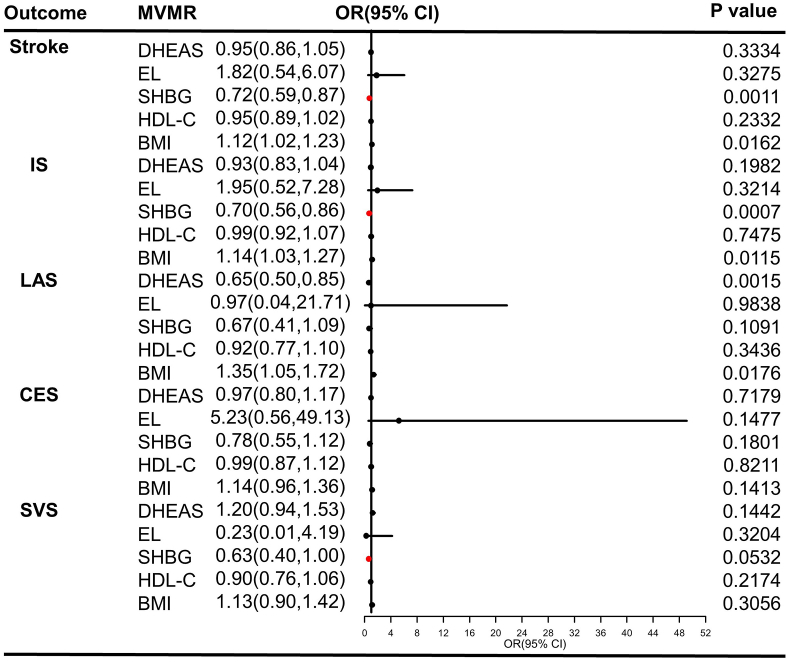
Causal estimates of SHBG on stroke and its subtypes in MVMR. S The red line means the threshold of *P* < 0.01. (For interpretation of the references to colour in this figure legend, the reader is referred to the Web version of this article.)

SNPs used as IVs for stroke and its subtypes in reverse MR analysis was displayed in Table S7. In the MVMR analysis, the results adjusted for dehydroepiandrosterone sulfate levels (DSL), estradiol levels (EL), high-density lipoprotein cholesterol (HDL-C), and body mass index (BMI) showed that increased SHBG level remains a protective factor for stroke (OR = 0.72, 95%CI = 0.59–0.87, *P* = 0.0011) and IS (OR = 0.70, 95%CI = 0.56–0.86, *P* = 0.0007), as shown in Fig. 5. However, the casual association between SHBG and SVS is suggestive in the MVMR analysis (OR = 0.63, 95%CI = 0.40–1,00, *P* = 0.0532).

## 4 Discussion

In our MR analyses, statistically significant inverse association between SHBG and stroke, and IS was noted. Moreover, these results remain stable in the reverse MR and multivariable MR analyses. Our study provides novel genetic insights that SHBG may play a protective role in stroke risk. Some previous studies reported that the association between SHBG and stroke can be attributed to several mediating factors such as BMI. In our MR study, the inverse association remains robust after performing reverse MR and adjusting confounding factors like BMI, DSL, and EL. Additionally, we confirm the direction of the causal effect of SHBG on stroke, while not the opposite.

Generally, the risk of stroke in women is lower than in men after adjusting the age. However, the incidence of stroke in women and men is similar in the aged, especially in postmenopausal women [34]. Different levels of endogenous sex hormones may partially explain the difference. In postmenopausal women, the lack of protective sex hormones may lead to an increased risk of stroke [9,12,[20], [21], [22], [23],^26^,[35], [36], [37], [38], [39]]. In this study, our findings demonstrate that SHBG exerts a protective effect on stroke and IS [12,[40], [41], [42], [43], [44], [45], [46], [47], [48]].

Although a potential link between SHBG and stroke is established in our study, our results cannot fully explain specific biological mechanisms related to protective effects of SHBG on stroke. Potential mechanisms include large vessel atherosclerosis or thrombogenesis. ^49-54^In our MR analysis, protective effects of SHBG on stroke are increased after adjusting DSL, EL, HDL-C, and BMI. The role of these factors may provide potential direction for the exploration of biological mechanisms [18,49]. In the analyses of subtypes, protective effects of SHBG on stroke are not detected, indicating the heterogeneity of different subtypes. In the reverse MR analysis, no causal effects of stroke and its subtypes on SHBG. The level of SHBG may be influenced by multiple factors after stroke. It is known that inflammatory responses were noted following stroke, such as inflammatory response, the dysfunction of neuroendocrine system, drug prescription, and metabolic changes.

The results of our MR analysis provide important clinical implications for the prediction of stroke risk. There are several strengths in our study. For example, we used several methods, including SVMR and MVMR, to prove the robustness of the results. Furthermore, several datasets are utilized to strength the reliability of our results. Finally, the application of SHBG test can be easily used as a effective tool to screen individuals with a high stroke risk.

This is the first MR study to illuminate the causal association between SHBG and stroke (subtypes). This MR analysis excludes conventional confounding factors in observational studies and yields causal inferences. In addition, these findings may provide valuable clinical insights for doctors in the clinic, especially for postmenopausal women. Mainour study has several limitations. Data are obtained from European individuals, which limits the further generalization of our findings. Additionally, we did not explore the role of SHBG on stroke in sex stratification due to unavailability of male and female stroke dataset. Sex differences in stroke incidence, pathophysiology, and outcomes are well-documented in the literature. It is known that SHBG can regulate the level of bioavailability of sex hormones such as testosterone and estradiol [49], which have been implicated in stroke risk. Therefore, incorporating SHBG levels into stroke risk prediction models, particularly when stratified by sex, could provide valuable insights into the interplay between SHBG and stroke.

## 5 Conclusion

In our bi-directional and multivariable MR study, an inverse causal association between SHBG and stroke (and IS) is observed, indicating that SHBG may play a protective effect on stroke and IS.

## Data availability statement

All data in our MR analyses are available from public databases.

## Ethics statement

Exposure and outcome datasets in this MR study were taken from de-identified public websites/studies, and ethical approval and informed consent were taken by the ethics committee in original articles [18,27]. Therefore, ethical approval was exempted from our MR study.

## Funding

This funding of this study was sourced from the 1·3·5 project for disciplines of excellence-Clinical Research Incubation Project, 10.13039/501100013365West China Hospital, Sichuan University (2018HXFH010).

## CRediT authorship contribution statement

**Qiang He:** Writing – original draft, Supervision, Software, Methodology, Investigation, Formal analysis, Data curation, Conceptualization. **Wenjing Wang:** Software, Methodology, Formal analysis. **Yang Xiong:** Writing – review & editing, Visualization, Validation, Supervision. **Chuanyuan Tao:** Project administration. **Lu Ma:** Visualization, Validation. **Jinming Han:** Writing – review & editing. **Chao You:** Writing – review & editing, Supervision, Project administration.

## Declaration of competing interest

All authors declare no potential conflict of interest.
