# Supplementary material for: A protective role of genetically predicted sex hormone-binding globulin on stroke
Source: Heliyon. 2024 Mar 30;10(7):e28556. doi: 10.1016/j.heliyon.2024.e28556 (PMC11002575; doi:10.1016/j.heliyon.2024.e28556)
Supplement: Multimedia component 1 [file mmc1.docx]

**Description**

**Table S1** SNPs used as IVs for SHBG in MR analysis

**Table S2** The OR and 95%CI in leave-one-out analysis

**Table S3** SNPs used as IVs for stroke in reverse MR analysis

**Table S4** SNPs used as IVs for IS in reverse MR analysis

**Table S5** SNPs used as IVs for LAS in reverse MR analysis

**Table S6** SNPs used as IVs for CES in reverse MR analysis

**Table S7** SNPs used as IVs for BMI, HDL-C, Estradiol, DHEAS and SHBG in MVMR analysis

**Table S1** SNPs used as IVs for SHBG in MR analysis

| SNP | effect allele | | | other allele | | pval | se | | | beta |
| --- | --- | --- | --- | --- | --- | --- | --- | --- | --- | --- |
| rs41309159 | T | | C | | -0.0135 | 0.0019 | | | 1.20E-12 | |
| rs9439469 | A | | G | | 0.0063 | 0.0010 | | | 4.00E-10 | |
| rs16835135 | G | | A | | 0.0143 | 0.0017 | | | 6.90E-18 | |
| rs237438 | G | | T | | -0.0062 | 0.0010 | | | 2.60E-11 | |
| rs4970837 | T | | G | | 0.0065 | 0.0010 | | | 4.10E-10 | |
| rs203777 | T | | C | | 0.0061 | 0.0010 | | | 8.10E-11 | |
| rs78444298 | A | | G | | -0.0288 | 0.0034 | | | 1.00E-19 | |
| rs857152 | T | | C | | 0.0087 | 0.0015 | | | 3.40E-11 | |
| rs2196943 | A | | G | | -0.0116 | 0.0010 | | | 3.40E-34 | |
| rs10900446 | A | | G | | -0.0062 | 0.0010 | | | 1.50E-11 | |
| rs115521489 | G | | A | | -0.0137 | 0.0022 | | | 4.10E-10 | |
| rs7540115 | C | | A | | 0.0068 | 0.0012 | | | 3.90E-09 | |
| rs1883783 | G | | T | | -0.0063 | 0.0009 | | | 4.80E-11 | |
| rs559986 | T | | C | | -0.0105 | 0.0016 | | | 1.80E-12 | |
| rs57754494 | A | | G | | 0.0112 | 0.0016 | | | 7.50E-13 | |
| rs6684464 | T | | C | | -0.0071 | 0.0011 | | | 6.50E-13 | |
| rs10864070 | A | | G | | 0.0083 | 0.0016 | | | 4.70E-08 | |
| rs340835 | A | | G | | -0.0076 | 0.0009 | | | 3.40E-18 | |
| rs113430755 | C | | T | | -0.0258 | 0.0050 | | | 1.90E-08 | |
| rs2275355 | T | | C | | 0.0090 | 0.0010 | | | 7.50E-20 | |
| rs7552207 | A | | G | | 0.0050 | 0.0010 | | | 9.60E-09 | |
| rs140584594 | G | | A | | -0.0132 | 0.0010 | | | 7.10E-38 | |
| rs7535528 | A | | G | | 0.0056 | 0.0010 | | | 8.20E-10 | |
| rs3001032 | C | | T | | 0.0092 | 0.0010 | | | 1.70E-20 | |
| rs2642438 | G | | A | | 0.0121 | 0.0010 | | | 2.00E-34 | |
| rs2807861 | G | | A | | -0.0056 | 0.0011 | | | 1.20E-08 | |
| rs11165493 | A | | G | | -0.0052 | 0.0010 | | | 2.20E-08 | |
| rs1730862 | A | | G | | -0.0237 | 0.0010 | | | 5.11E-133 | |
| rs11121522 | C | | T | | 0.0084 | 0.0015 | | | 3.00E-09 | |
| rs111331455 | G | | A | | 0.0057 | 0.0010 | | | 8.90E-09 | |
| rs35070405 | C | | T | | -0.0055 | 0.0010 | | | 1.30E-08 | |
| rs2273368 | T | | C | | 0.0067 | 0.0012 | | | 2.70E-09 | |
| rs1969213 | G | | A | | -0.0047 | 0.0009 | | | 2.40E-08 | |
| rs267733 | G | | A | | -0.0129 | 0.0013 | | | 5.00E-26 | |
| rs114165349 | C | | G | | -0.0782 | 0.0031 | | | 7.29E-150 | |
| rs2234922 | G | | A | | -0.0075 | 0.0012 | | | 1.10E-09 | |
| rs35346083 | A | | C | | -0.0087 | 0.0009 | | | 1.10E-21 | |
| rs59708846 | A | | G | | 0.0181 | 0.0018 | | | 1.60E-26 | |
| rs2022865 | G | | A | | 0.0064 | 0.0013 | | | 4.10E-08 | |
| rs72694845 | C | | A | | -0.0181 | 0.0028 | | | 1.80E-11 | |
| rs4147563 | T | | C | | 0.0130 | 0.0016 | | | 3.30E-17 | |
| rs822508 | C | | T | | 0.0077 | 0.0010 | | | 2.20E-15 | |
| rs12059956 | A | G | -0.0076 | | 0.0009 | 7.00E-17 | |  |  |  |
| rs12138461 | G | A | 0.0056 | | 0.0010 | 3.10E-09 | |  |  |  |
| rs12758998 | A | G | 0.0116 | | 0.0017 | 2.00E-13 | |  |  |  |
| rs2782657 | C | G | -0.0049 | | 0.0009 | 2.70E-08 | |  |  |  |
| rs12089078 | C | T | -0.0092 | | 0.0011 | 2.10E-16 | |  |  |  |
| rs10888696 | A | G | 0.0067 | | 0.0009 | 8.20E-14 | |  |  |  |
| rs17583875 | A | G | 0.0266 | | 0.0032 | 2.60E-18 | |  |  |  |
| rs6691427 | C | G | 0.0051 | | 0.0009 | 3.60E-08 | |  |  |  |
| rs138755456 | C | T | 0.0102 | | 0.0014 | 1.20E-13 | |  |  |  |
| rs6422513 | A | G | 0.0113 | | 0.0011 | 4.30E-23 | |  |  |  |
| rs9427104 | T | C | 0.0120 | | 0.0009 | 1.90E-41 | |  |  |  |
| rs114469183 | A | G | 0.0105 | | 0.0019 | 1.00E-08 | |  |  |  |
| rs12030554 | T | A | 0.0058 | | 0.0010 | 1.20E-09 | |  |  |  |
| rs4639796 | A | G | -0.0140 | | 0.0013 | 1.70E-28 | |  |  |  |
| rs1870927 | A | T | 0.0072 | | 0.0010 | 1.80E-14 | |  |  |  |
| rs413426 | G | C | -0.0125 | | 0.0021 | 3.10E-09 | |  |  |  |
| rs662026 | G | A | -0.0099 | | 0.0012 | 2.20E-18 | |  |  |  |
| rs75077113 | C | A | 0.0071 | | 0.0010 | 1.90E-11 | |  |  |  |
| rs36086195 | T | C | 0.0117 | | 0.0009 | 9.80E-36 | |  |  |  |
| rs61779331 | A | C | -0.0150 | | 0.0011 | 6.10E-43 | |  |  |  |
| rs4674669 | T | C | -0.0068 | | 0.0013 | 2.30E-08 | |  |  |  |
| rs1420385 | G | A | -0.0051 | | 0.0009 | 1.50E-08 | |  |  |  |
| rs13035806 | A | G | -0.0089 | | 0.0015 | 2.20E-10 | |  |  |  |
| rs12476661 | A | G | 0.0071 | | 0.0011 | 1.30E-10 | |  |  |  |
| rs4668732 | T | A | 0.0062 | | 0.0010 | 4.40E-13 | |  |  |  |
| rs112833123 | T | C | -0.0195 | | 0.0019 | 1.90E-26 | |  |  |  |
| rs2305144 | C | A | -0.0066 | | 0.0009 | 6.50E-12 | |  |  |  |
| rs11164095 | T | C | 0.0057 | | 0.0010 | 2.10E-09 | |  |  |  |
| rs6546096 | G | A | -0.0214 | | 0.0011 | 8.70E-97 | |  |  |  |
| rs13000027 | C | A | 0.0054 | | 0.0009 | 2.20E-09 | |  |  |  |
| rs35633876 | T | G | -0.0078 | | 0.0009 | 2.70E-17 | |  |  |  |
| rs12616420 | G | C | 0.0083 | | 0.0010 | 2.60E-16 | |  |  |  |
| rs111905890 | T | C | -0.0155 | | 0.0026 | 7.90E-11 | |  |  |  |
| rs62130544 | T | A | 0.0168 | | 0.0031 | 4.30E-08 | |  |  |  |
| rs182636083 | T | C | 0.0081 | | 0.0011 | 2.30E-15 | |  |  |  |
| rs11690176 | A | G | 0.0058 | | 0.0010 | 5.00E-09 | |  |  |  |
| rs62182125 | A | G | -0.0065 | | 0.0009 | 9.90E-13 | |  |  |  |
| rs11682084 | A | C | -0.0068 | | 0.0010 | 2.10E-11 | |  |  |  |
| rs12989083 | G | A | 0.0069 | | 0.0010 | 3.60E-12 | |  |  |  |
| rs3770781 | G | A | 0.0055 | | 0.0009 | 3.30E-09 | |  |  |  |
| rs6736913 | G | A | -0.0323 | | 0.0032 | 1.40E-25 | |  |  |  |
| rs11688682 | C | G | 0.0070 | | 0.0011 | 9.20E-12 | |  |  |  |
| rs78058190 | A | G | -0.0194 | | 0.0024 | 5.50E-17 | |  |  |  |
| rs78757437 | A | T | -0.0057 | | 0.0010 | 9.70E-09 | |  |  |  |
| rs787976 | C | T | 0.0061 | | 0.0012 | 1.40E-08 | |  |  |  |
| rs2551644 | T | A | 0.0120 | | 0.0012 | 2.30E-25 | |  |  |  |
| rs2723067 | G | A | 0.0064 | | 0.0009 | 1.40E-12 | |  |  |  |
| rs62195072 | C | T | 0.0073 | | 0.0010 | 7.80E-14 | |  |  |  |
| rs6755571 | A | C | -0.0109 | | 0.0019 | 4.70E-08 | |  |  |  |
| rs882186 | T | A | -0.0050 | | 0.0010 | 3.00E-08 | |  |  |  |
| rs4671328 | G | T | 0.0067 | | 0.0009 | 1.40E-14 | |  |  |  |
| rs12373799 | A | G | 0.0127 | | 0.0016 | 3.70E-16 | |  |  |  |
| rs72836346 | C | G | 0.0094 | | 0.0018 | 3.30E-08 | |  |  |  |
| rs3761706 | A | G | -0.0155 | | 0.0019 | 9.70E-18 | |  |  |  |
| rs149214447 | G | C | -0.0212 | | 0.0037 | 1.70E-08 | |  |  |  |
| rs114940462 | T | G | 0.0251 | | 0.0023 | 1.90E-27 | |  |  |  |
| rs72810505 | T | C | 0.0088 | | 0.0017 | 3.10E-08 | |  |  |  |
| rs2304686 | A | G | 0.0055 | | 0.0009 | 2.70E-10 | |  |  |  |
| rs72787511 | C | G | 0.0158 | | 0.0025 | 5.90E-10 | |  |  |  |
| rs201570119 | C | T | 0.0073 | | 0.0011 | 2.60E-11 | |  |  |  |
| rs10187560 | C | T | -0.0051 | | 0.0010 | 2.20E-08 | |  |  |  |
| rs11682797 | A | T | 0.0075 | | 0.0011 | 1.30E-12 | |  |  |  |
| rs702876 | T | C | 0.0053 | | 0.0009 | 1.80E-09 | |  |  |  |
| rs17008851 | G | A | 0.0091 | | 0.0014 | 3.50E-12 | |  |  |  |
| rs6710171 | T | C | -0.0047 | | 0.0009 | 3.10E-08 | |  |  |  |
| rs4665972 | C | T | 0.0349 | | 0.0010 | 1.00E-200 | |  |  |  |
| rs13405815 | T | C | 0.0052 | | 0.0009 | 3.00E-08 | |  |  |  |
| rs13427019 | A | T | -0.0097 | | 0.0011 | 1.60E-18 | |  |  |  |
| rs13389219 | T | C | 0.0137 | | 0.0009 | 6.40E-53 | |  |  |  |
| rs10208512 | G | A | -0.0145 | | 0.0023 | 9.00E-12 | |  |  |  |
| rs715 | C | T | 0.0071 | | 0.0010 | 1.60E-12 | |  |  |  |
| rs116713089 | A | G | 0.0094 | | 0.0018 | 7.90E-09 | |  |  |  |
| rs6741180 | A | G | -0.0048 | | 0.0010 | 1.50E-08 | |  |  |  |
| rs55761545 | T | C | -0.0064 | | 0.0011 | 3.80E-10 | |  |  |  |
| rs57467915 | A | G | -0.0241 | | 0.0038 | 1.50E-10 | |  |  |  |
| rs2972145 | C | T | -0.0135 | | 0.0010 | 1.00E-46 | |  |  |  |
| rs4665710 | C | A | 0.0071 | | 0.0011 | 3.40E-10 | |  |  |  |
| rs13094241 | G | T | 0.0080 | | 0.0010 | 3.40E-15 | |  |  |  |
| rs6791074 | T | C | 0.0134 | | 0.0024 | 1.70E-08 | |  |  |  |
| rs35333155 | T | C | -0.0072 | | 0.0009 | 1.50E-16 | |  |  |  |
| rs149102638 | A | G | -0.0143 | | 0.0026 | 4.50E-08 | |  |  |  |
| rs9823108 | A | C | -0.0063 | | 0.0010 | 6.80E-13 | |  |  |  |
| rs1823227 | G | T | 0.0100 | | 0.0011 | 3.50E-21 | |  |  |  |
| rs79287178 | A | G | -0.0347 | | 0.0028 | 3.70E-36 | |  |  |  |
| rs57158761 | G | A | -0.0072 | | 0.0009 | 1.40E-16 | |  |  |  |
| rs784504 | C | G | 0.0070 | | 0.0012 | 2.00E-10 | |  |  |  |
| rs33807 | A | G | -0.0073 | | 0.0014 | 1.60E-08 | |  |  |  |
| rs12696304 | G | C | 0.0068 | | 0.0011 | 1.60E-10 | |  |  |  |
| rs9823118 | A | G | 0.0062 | | 0.0010 | 1.40E-10 | |  |  |  |
| rs9968070 | A | G | -0.0050 | | 0.0009 | 1.90E-08 | |  |  |  |
| rs13315174 | A | G | -0.0076 | | 0.0011 | 1.20E-12 | |  |  |  |
| rs7638782 | A | G | 0.0085 | | 0.0016 | 5.40E-09 | |  |  |  |
| rs687339 | T | C | -0.0312 | | 0.0011 | 4.20E-182 | |  |  |  |
| rs9831794 | A | G | 0.0062 | | 0.0009 | 1.50E-11 | |  |  |  |
| rs9844972 | C | G | -0.0144 | | 0.0018 | 2.60E-16 | |  |  |  |
| rs6804915 | A | C | -0.0064 | | 0.0010 | 2.70E-10 | |  |  |  |
| rs7617967 | C | T | -0.0070 | | 0.0013 | 4.90E-08 | |  |  |  |
| rs35983031 | T | C | -0.0093 | | 0.0016 | 3.50E-10 | |  |  |  |
| rs13063578 | A | T | -0.0077 | | 0.0010 | 4.10E-18 | |  |  |  |
| rs4974310 | T | C | 0.0053 | | 0.0009 | 7.00E-09 | |  |  |  |
| rs2176887 | T | C | 0.0118 | | 0.0019 | 1.60E-11 | |  |  |  |
| rs34970607 | A | G | 0.0054 | | 0.0011 | 1.20E-08 | |  |  |  |
| rs61292904 | G | A | -0.0075 | | 0.0014 | 4.00E-08 | |  |  |  |
| rs9871160 | C | T | 0.0111 | | 0.0013 | 6.60E-18 | |  |  |  |
| rs59662471 | T | C | 0.0059 | | 0.0010 | 1.30E-08 | |  |  |  |
| rs35226891 | A | G | 0.0070 | | 0.0010 | 2.10E-13 | |  |  |  |
| rs6792725 | G | A | 0.0141 | | 0.0010 | 2.00E-46 | |  |  |  |
| rs12636106 | T | C | 0.0073 | | 0.0013 | 2.10E-09 | |  |  |  |
| rs9849171 | C | G | 0.0081 | | 0.0010 | 9.30E-18 | |  |  |  |
| rs1801282 | G | C | 0.0183 | | 0.0014 | 1.30E-37 | |  |  |  |
| rs13086465 | T | C | -0.0112 | | 0.0010 | 3.60E-30 | |  |  |  |
| rs234051 | G | A | -0.0079 | | 0.0010 | 2.90E-17 | |  |  |  |
| rs73193388 | T | C | 0.0102 | | 0.0014 | 7.30E-14 | |  |  |  |
| rs72666817 | A | G | 0.0103 | | 0.0012 | 4.70E-19 | |  |  |  |
| rs3468 | A | G | 0.0067 | | 0.0010 | 5.80E-13 | |  |  |  |
| rs13149606 | T | C | 0.0062 | | 0.0011 | 4.10E-09 | |  |  |  |
| rs114627598 | C | T | -0.0115 | | 0.0022 | 1.30E-08 | |  |  |  |
| rs114053844 | C | T | 0.0282 | | 0.0037 | 3.40E-15 | |  |  |  |
| rs7678138 | A | G | -0.0099 | | 0.0014 | 1.90E-13 | |  |  |  |
| rs62334584 | T | C | 0.0064 | | 0.0010 | 3.30E-11 | |  |  |  |
| rs2970877 | G | T | 0.0077 | | 0.0010 | 2.00E-13 | |  |  |  |
| rs7668413 | C | T | -0.0058 | | 0.0010 | 2.20E-11 | |  |  |  |
| rs10026753 | G | A | -0.0066 | | 0.0011 | 8.40E-10 | |  |  |  |
| rs28925904 | T | C | -0.0242 | | 0.0030 | 7.00E-17 | |  |  |  |
| rs41280463 | A | G | -0.0101 | | 0.0012 | 4.50E-16 | |  |  |  |
| rs6552186 | A | G | -0.0492 | | 0.0065 | 1.90E-15 | |  |  |  |
| rs13150068 | G | A | -0.0188 | | 0.0009 | 7.30E-95 | |  |  |  |
| rs34311866 | C | T | -0.0083 | | 0.0012 | 2.20E-12 | |  |  |  |
| rs4274814 | T | C | 0.0058 | | 0.0009 | 3.90E-10 | |  |  |  |
| rs2627690 | G | A | 0.0065 | | 0.0010 | 5.70E-10 | |  |  |  |
| rs144459202 | A | G | -0.0223 | | 0.0039 | 3.70E-08 | |  |  |  |
| rs4568281 | A | G | 0.0082 | | 0.0010 | 4.10E-16 | |  |  |  |
| rs363072 | T | A | -0.0086 | | 0.0014 | 6.00E-09 | |  |  |  |
| rs3958042 | A | G | -0.0058 | | 0.0011 | 2.90E-08 | |  |  |  |
| rs182848434 | A | G | -0.0226 | | 0.0039 | 1.60E-10 | |  |  |  |
| rs570946718 | C | A | 0.0332 | | 0.0049 | 3.00E-12 | |  |  |  |
| rs7696472 | A | G | 0.0132 | | 0.0009 | 6.70E-48 | |  |  |  |
| rs189595752 | G | A | 0.0117 | | 0.0015 | 4.90E-15 | |  |  |  |
| rs28507491 | A | G | 0.0125 | | 0.0010 | 1.90E-38 | |  |  |  |
| rs78890745 | A | G | 0.0178 | | 0.0015 | 8.80E-32 | |  |  |  |
| rs2705619 | A | G | -0.0094 | | 0.0010 | 2.40E-22 | |  |  |  |
| rs13108218 | G | A | -0.0228 | | 0.0010 | 9.20E-129 | |  |  |  |
| rs112850234 | A | G | -0.0336 | | 0.0045 | 9.30E-15 | |  |  |  |
| rs56257546 | C | G | 0.0197 | | 0.0027 | 8.70E-15 | |  |  |  |
| rs10027275 | C | G | -0.0107 | | 0.0011 | 1.10E-26 | |  |  |  |
| rs7688970 | C | G | 0.0061 | | 0.0011 | 2.40E-08 | |  |  |  |
| rs1433210 | C | A | 0.0067 | | 0.0011 | 2.20E-10 | |  |  |  |
| rs2602856 | A | C | -0.0136 | | 0.0010 | 8.50E-44 | |  |  |  |
| rs182132993 | A | G | -0.0271 | | 0.0049 | 1.20E-09 | |  |  |  |
| rs2724475 | C | T | -0.0077 | | 0.0011 | 4.50E-13 | |  |  |  |
| rs34707604 | C | T | 0.0172 | | 0.0011 | 3.10E-56 | |  |  |  |
| rs999634 | A | C | 0.0051 | | 0.0009 | 8.40E-09 | |  |  |  |
| rs11957006 | G | C | 0.0124 | | 0.0021 | 1.20E-10 | |  |  |  |
| rs6556402 | A | G | 0.0055 | | 0.0010 | 4.80E-09 | |  |  |  |
| rs329122 | A | G | -0.0060 | | 0.0009 | 5.30E-11 | |  |  |  |
| rs2431752 | A | G | 0.0093 | | 0.0015 | 2.40E-10 | |  |  |  |
| rs1981808 | A | T | 0.0070 | | 0.0010 | 2.20E-12 | |  |  |  |
| rs72753349 | T | C | 0.0128 | | 0.0024 | 9.30E-09 | |  |  |  |
| rs6864575 | A | T | 0.0045 | | 0.0009 | 3.70E-08 | |  |  |  |
| rs79354983 | G | A | -0.0126 | | 0.0016 | 3.90E-16 | |  |  |  |
| rs11748938 | A | G | 0.0064 | | 0.0011 | 1.70E-08 | |  |  |  |
| rs2885582 | A | G | -0.0075 | | 0.0012 | 3.60E-11 | |  |  |  |
| rs4976033 | G | A | -0.0059 | | 0.0010 | 1.80E-11 | |  |  |  |
| rs2063245 | G | A | -0.0069 | | 0.0010 | 8.50E-12 | |  |  |  |
| rs359431 | T | C | 0.0046 | | 0.0009 | 2.20E-08 | |  |  |  |
| rs4073358 | T | C | 0.0056 | | 0.0009 | 1.70E-09 | |  |  |  |
| rs6879874 | T | A | 0.0052 | | 0.0010 | 7.30E-09 | |  |  |  |
| rs12916 | C | T | 0.0063 | | 0.0010 | 8.60E-13 | |  |  |  |
| rs72709458 | T | C | -0.0058 | | 0.0012 | 4.90E-08 | |  |  |  |
| rs34651 | T | C | 0.0093 | | 0.0017 | 3.50E-09 | |  |  |  |
| rs6860245 | C | G | 0.0100 | | 0.0011 | 1.90E-20 | |  |  |  |
| rs3733892 | C | A | 0.0060 | | 0.0010 | 9.30E-10 | |  |  |  |
| rs112959387 | C | G | -0.0086 | | 0.0015 | 1.60E-08 | |  |  |  |
| rs9686661 | T | C | -0.0113 | | 0.0012 | 1.30E-23 | |  |  |  |
| rs2522054 | T | G | 0.0083 | | 0.0012 | 2.60E-11 | |  |  |  |
| rs1650548 | C | G | -0.0096 | | 0.0011 | 2.10E-18 | |  |  |  |
| rs10041660 | C | T | 0.0055 | | 0.0009 | 4.80E-09 | |  |  |  |
| rs40270 | C | A | -0.0167 | | 0.0011 | 6.80E-51 | |  |  |  |
| rs79683734 | A | G | 0.0122 | | 0.0022 | 9.70E-09 | |  |  |  |
| rs185406435 | G | A | -0.0168 | | 0.0015 | 3.30E-30 | |  |  |  |
| rs11743810 | T | C | 0.0077 | | 0.0009 | 4.40E-16 | |  |  |  |
| rs11155787 | T | C | -0.0054 | | 0.0010 | 9.00E-10 | |  |  |  |
| rs6940266 | G | C | -0.0065 | | 0.0010 | 1.10E-11 | |  |  |  |
| rs111637026 | A | G | -0.0070 | | 0.0012 | 1.60E-09 | |  |  |  |
| rs117108573 | T | C | -0.0116 | | 0.0018 | 3.70E-11 | |  |  |  |
| rs3813498 | T | C | -0.0068 | | 0.0012 | 7.40E-09 | |  |  |  |
| rs3004179 | G | A | -0.0052 | | 0.0010 | 6.60E-09 | |  |  |  |
| rs1408270 | G | A | 0.0098 | | 0.0011 | 1.00E-21 | |  |  |  |
| rs150359 | T | A | 0.0062 | | 0.0009 | 2.20E-11 | |  |  |  |
| rs112928223 | T | C | 0.0235 | | 0.0040 | 4.40E-09 | |  |  |  |
| rs9495298 | G | A | 0.0077 | | 0.0012 | 1.60E-10 | |  |  |  |
| rs10946313 | C | T | -0.0070 | | 0.0010 | 1.00E-13 | |  |  |  |
| rs7756992 | G | A | -0.0063 | | 0.0011 | 5.10E-09 | |  |  |  |
| rs1570360 | G | A | -0.0059 | | 0.0010 | 2.20E-09 | |  |  |  |
| rs998584 | A | C | -0.0074 | | 0.0009 | 6.80E-16 | |  |  |  |
| rs62396733 | T | C | -0.0163 | | 0.0014 | 1.20E-34 | |  |  |  |
| rs9267088 | A | T | -0.0132 | | 0.0014 | 1.10E-24 | |  |  |  |
| rs6910879 | G | A | -0.0124 | | 0.0017 | 7.80E-15 | |  |  |  |
| rs9465601 | A | G | 0.0058 | | 0.0011 | 6.40E-09 | |  |  |  |
| rs11153046 | G | A | -0.0060 | | 0.0010 | 5.50E-10 | |  |  |  |
| rs4709746 | T | C | 0.0076 | | 0.0014 | 8.40E-09 | |  |  |  |
| rs58321169 | T | C | -0.0083 | | 0.0011 | 3.30E-14 | |  |  |  |
| rs11542663 | C | A | -0.0057 | | 0.0010 | 1.10E-08 | |  |  |  |
| rs1775125 | C | T | 0.0065 | | 0.0011 | 3.00E-10 | |  |  |  |
| rs1743954 | T | C | -0.0049 | | 0.0010 | 4.10E-08 | |  |  |  |
| rs545366210 | C | T | -0.0166 | | 0.0033 | 4.00E-08 | |  |  |  |
| rs186766320 | A | G | 0.0258 | | 0.0029 | 1.80E-22 | |  |  |  |
| rs201474301 | T | C | -0.0099 | | 0.0015 | 1.10E-11 | |  |  |  |
| rs9379084 | A | G | -0.0145 | | 0.0015 | 4.60E-23 | |  |  |  |
| rs575147125 | G | T | -0.0090 | | 0.0015 | 1.20E-10 | |  |  |  |
| rs374943348 | A | T | -0.0090 | | 0.0011 | 2.00E-15 | |  |  |  |
| rs9278026 | T | A | -0.0091 | | 0.0013 | 2.80E-13 | |  |  |  |
| rs7451021 | C | T | -0.0094 | | 0.0010 | 3.10E-21 | |  |  |  |
| rs668871 | T | C | 0.0161 | | 0.0009 | 1.70E-76 | |  |  |  |
| rs9267551 | G | C | -0.0159 | | 0.0013 | 1.00E-29 | |  |  |  |
| rs28890929 | T | G | -0.0081 | | 0.0014 | 4.40E-11 | |  |  |  |
| rs9492 | C | A | -0.0096 | | 0.0013 | 2.20E-14 | |  |  |  |
| rs13207809 | C | G | -0.0103 | | 0.0015 | 7.20E-13 | |  |  |  |
| rs76345703 | G | A | 0.0066 | | 0.0012 | 1.00E-09 | |  |  |  |
| rs6939861 | A | G | -0.0147 | | 0.0011 | 1.70E-43 | |  |  |  |
| rs543504257 | C | A | -0.0088 | | 0.0011 | 9.00E-18 | |  |  |  |
| rs4135240 | C | T | 0.0058 | | 0.0010 | 1.30E-09 | |  |  |  |
| rs2908522 | G | C | 0.0052 | | 0.0010 | 8.10E-09 | |  |  |  |
| rs1738380 | T | C | 0.0056 | | 0.0011 | 3.40E-08 | |  |  |  |
| rs38197 | C | G | 0.0066 | | 0.0011 | 1.40E-08 | |  |  |  |
| rs10951130 | G | T | -0.0093 | | 0.0013 | 2.90E-14 | |  |  |  |
| rs2246223 | T | C | 0.0055 | | 0.0009 | 1.00E-08 | |  |  |  |
| rs738134 | G | C | -0.0069 | | 0.0010 | 8.80E-11 | |  |  |  |
| rs157935 | G | T | 0.0110 | | 0.0010 | 5.70E-29 | |  |  |  |
| rs2551774 | G | A | -0.0077 | | 0.0010 | 6.60E-16 | |  |  |  |
| rs7015 | G | A | 0.0350 | | 0.0012 | 1.50E-195 | |  |  |  |
| rs2074683 | T | C | 0.0062 | | 0.0009 | 1.10E-12 | |  |  |  |
| rs7809920 | T | C | 0.0070 | | 0.0010 | 6.30E-12 | |  |  |  |
| rs1799831 | T | C | -0.0075 | | 0.0013 | 1.10E-08 | |  |  |  |
| rs4725944 | C | G | 0.0072 | | 0.0010 | 2.80E-13 | |  |  |  |
| rs10258433 | G | A | -0.0048 | | 0.0009 | 1.00E-08 | |  |  |  |
| rs42238 | T | C | 0.0068 | | 0.0012 | 3.50E-09 | |  |  |  |
| rs540655284 | A | G | -0.0270 | | 0.0041 | 6.60E-12 | |  |  |  |
| rs10273476 | A | G | 0.0053 | | 0.0009 | 4.20E-10 | |  |  |  |
| rs111363146 | C | T | 0.0071 | | 0.0014 | 2.20E-08 | |  |  |  |
| rs73705826 | A | G | 0.0105 | | 0.0017 | 2.40E-08 | |  |  |  |
| rs1635025 | A | T | 0.0111 | | 0.0015 | 1.60E-13 | |  |  |  |
| rs114949263 | C | T | 0.0167 | | 0.0015 | 4.60E-33 | |  |  |  |
| rs12667888 | C | T | 0.0172 | | 0.0012 | 5.50E-48 | |  |  |  |
| rs10238028 | G | A | 0.0164 | | 0.0019 | 3.60E-19 | |  |  |  |
| rs1352084 | C | A | 0.0070 | | 0.0010 | 4.90E-13 | |  |  |  |
| rs2404976 | A | C | -0.0071 | | 0.0011 | 6.70E-12 | |  |  |  |
| rs1059698 | C | A | -0.0061 | | 0.0011 | 9.40E-09 | |  |  |  |
| rs11556924 | T | C | 0.0088 | | 0.0010 | 1.80E-19 | |  |  |  |
| rs38304 | A | G | -0.0077 | | 0.0012 | 2.90E-09 | |  |  |  |
| rs58429317 | A | G | 0.0067 | | 0.0010 | 1.00E-11 | |  |  |  |
| rs368214 | T | C | 0.0084 | | 0.0009 | 2.40E-20 | |  |  |  |
| rs1534696 | A | C | 0.0052 | | 0.0009 | 1.10E-08 | |  |  |  |
| rs445 | T | C | -0.0121 | | 0.0016 | 5.00E-15 | |  |  |  |
| rs149092986 | C | T | -0.0188 | | 0.0032 | 4.30E-08 | |  |  |  |
| rs1229498 | G | T | -0.0111 | | 0.0011 | 6.20E-27 | |  |  |  |
| rs38855 | G | A | -0.0061 | | 0.0009 | 6.00E-11 | |  |  |  |
| rs11764444 | A | T | 0.0055 | | 0.0011 | 4.10E-08 | |  |  |  |
| rs11765639 | A | G | -0.0076 | | 0.0010 | 1.30E-15 | |  |  |  |
| rs34372369 | A | G | 0.0166 | | 0.0021 | 6.90E-13 | |  |  |  |
| rs71538127 | G | C | -0.0087 | | 0.0014 | 1.40E-10 | |  |  |  |
| rs1708302 | T | C | 0.0090 | | 0.0009 | 4.10E-24 | |  |  |  |
| rs9297994 | A | G | 0.0115 | | 0.0010 | 3.10E-31 | |  |  |  |
| rs2280838 | T | C | -0.0065 | | 0.0009 | 5.90E-12 | |  |  |  |
| rs2942202 | C | A | -0.0063 | | 0.0009 | 4.80E-13 | |  |  |  |
| rs7828742 | G | A | -0.0088 | | 0.0010 | 3.20E-22 | |  |  |  |
| rs7464506 | T | A | -0.0065 | | 0.0010 | 8.60E-11 | |  |  |  |
| rs113973451 | T | G | -0.0059 | | 0.0012 | 2.70E-08 | |  |  |  |
| rs10110651 | C | T | -0.0073 | | 0.0013 | 1.40E-08 | |  |  |  |
| rs72663937 | C | T | -0.0076 | | 0.0014 | 6.80E-09 | |  |  |  |
| rs34880012 | C | T | -0.0135 | | 0.0015 | 4.50E-21 | |  |  |  |
| rs10504731 | C | T | 0.0072 | | 0.0011 | 1.40E-11 | |  |  |  |
| rs55831924 | T | C | 0.0063 | | 0.0010 | 1.50E-11 | |  |  |  |
| rs1431659 | G | A | 0.0059 | | 0.0011 | 3.80E-08 | |  |  |  |
| rs575452 | G | A | 0.0192 | | 0.0013 | 1.50E-54 | |  |  |  |
| rs10808843 | T | A | 0.0152 | | 0.0011 | 6.40E-46 | |  |  |  |
| rs4841133 | G | A | 0.0216 | | 0.0016 | 9.60E-45 | |  |  |  |
| rs10095103 | C | T | -0.0050 | | 0.0009 | 2.10E-08 | |  |  |  |
| rs12543287 | C | G | 0.0106 | | 0.0010 | 1.50E-30 | |  |  |  |
| rs11997548 | A | C | 0.0054 | | 0.0010 | 5.10E-09 | |  |  |  |
| rs2241261 | T | C | -0.0063 | | 0.0009 | 1.70E-11 | |  |  |  |
| rs10111451 | C | T | 0.0050 | | 0.0009 | 6.90E-09 | |  |  |  |
| rs13251458 | A | G | 0.0095 | | 0.0009 | 9.20E-27 | |  |  |  |
| rs11994858 | G | A | 0.0124 | | 0.0010 | 5.30E-40 | |  |  |  |
| rs59203582 | T | A | 0.0218 | | 0.0013 | 7.10E-73 | |  |  |  |
| rs62515079 | G | A | 0.0260 | | 0.0034 | 6.10E-15 | |  |  |  |
| rs7000496 | T | C | -0.0123 | | 0.0017 | 2.40E-13 | |  |  |  |
| rs34563869 | A | T | -0.0080 | | 0.0014 | 3.00E-09 | |  |  |  |
| rs10964337 | G | A | -0.0069 | | 0.0012 | 1.60E-08 | |  |  |  |
| rs11791747 | G | A | 0.0073 | | 0.0010 | 1.40E-13 | |  |  |  |
| rs3780190 | G | A | 0.0103 | | 0.0009 | 1.60E-29 | |  |  |  |
| rs35233014 | A | C | -0.0139 | | 0.0011 | 5.00E-37 | |  |  |  |
| rs1330307 | C | A | -0.0077 | | 0.0009 | 4.00E-18 | |  |  |  |
| rs1567353 | G | C | -0.0072 | | 0.0010 | 3.20E-11 | |  |  |  |
| rs56021343 | T | G | -0.0073 | | 0.0011 | 1.80E-11 | |  |  |  |
| rs62580766 | T | C | 0.0109 | | 0.0012 | 9.80E-23 | |  |  |  |
| rs10733608 | T | G | 0.0095 | | 0.0009 | 1.20E-23 | |  |  |  |
| rs17372936 | C | T | -0.0071 | | 0.0011 | 5.40E-12 | |  |  |  |
| rs10125995 | T | C | 0.0046 | | 0.0009 | 8.40E-09 | |  |  |  |
| rs1411432 | C | A | -0.0066 | | 0.0012 | 2.50E-08 | |  |  |  |
| rs62565259 | T | C | 0.0088 | | 0.0012 | 3.40E-13 | |  |  |  |
| rs796004 | T | C | 0.0235 | | 0.0011 | 1.80E-108 | |  |  |  |
| rs7863263 | C | T | 0.0054 | | 0.0010 | 7.30E-10 | |  |  |  |
| rs8176693 | T | C | -0.0114 | | 0.0019 | 1.60E-08 | |  |  |  |
| rs6476065 | T | C | -0.0048 | | 0.0010 | 1.80E-08 | |  |  |  |
| rs2488274 | C | G | -0.0063 | | 0.0012 | 1.20E-08 | |  |  |  |
| rs74551598 | C | A | -0.0059 | | 0.0011 | 4.40E-08 | |  |  |  |
| rs4837794 | C | T | -0.0082 | | 0.0010 | 8.50E-19 | |  |  |  |
| rs10815276 | G | A | 0.0068 | | 0.0009 | 1.00E-13 | |  |  |  |
| rs10123811 | T | C | 0.0088 | | 0.0009 | 3.00E-22 | |  |  |  |
| rs820503 | A | C | -0.0118 | | 0.0014 | 1.60E-19 | |  |  |  |
| rs700085 | C | T | -0.0105 | | 0.0017 | 2.40E-12 | |  |  |  |
| rs72766607 | G | T | -0.0318 | | 0.0033 | 1.00E-21 | |  |  |  |
| rs9697210 | A | G | -0.0167 | | 0.0013 | 2.70E-39 | |  |  |  |
| rs80226362 | T | G | -0.0063 | | 0.0012 | 4.40E-08 | |  |  |  |
| rs117135073 | T | C | -0.0178 | | 0.0030 | 1.30E-08 | |  |  |  |
| rs4745876 | A | G | 0.0090 | | 0.0013 | 7.10E-11 | |  |  |  |
| rs75156222 | A | G | -0.0325 | | 0.0037 | 5.60E-18 | |  |  |  |
| rs10761749 | T | C | 0.0367 | | 0.0011 | 1.00E-200 | |  |  |  |
| rs79093169 | T | A | -0.0426 | | 0.0043 | 9.30E-24 | |  |  |  |
| rs1864390 | T | C | 0.0352 | | 0.0010 | 1.00E-200 | |  |  |  |
| rs17755271 | A | G | 0.0107 | | 0.0014 | 2.40E-16 | |  |  |  |
| rs79717793 | A | G | -0.0226 | | 0.0013 | 2.00E-78 | |  |  |  |
| rs3824655 | C | G | 0.0060 | | 0.0009 | 1.40E-11 | |  |  |  |
| rs146188397 | C | T | 0.0436 | | 0.0064 | 2.90E-12 | |  |  |  |
| rs7918533 | C | A | -0.0165 | | 0.0025 | 7.30E-12 | |  |  |  |
| rs10761676 | C | T | 0.0084 | | 0.0009 | 4.60E-21 | |  |  |  |
| rs190712219 | A | G | 0.0346 | | 0.0038 | 1.60E-21 | |  |  |  |
| rs72815155 | G | A | -0.0065 | | 0.0010 | 5.90E-10 | |  |  |  |
| rs2792022 | C | T | 0.0081 | | 0.0010 | 4.30E-17 | |  |  |  |
| rs72819010 | G | C | -0.0070 | | 0.0012 | 9.50E-10 | |  |  |  |
| rs11191841 | C | T | 0.0056 | | 0.0009 | 5.20E-09 | |  |  |  |
| rs12413488 | A | G | -0.0066 | | 0.0012 | 3.00E-09 | |  |  |  |
| rs4367880 | C | G | -0.0077 | | 0.0012 | 4.60E-12 | |  |  |  |
| rs11190245 | C | T | 0.0060 | | 0.0010 | 6.40E-09 | |  |  |  |
| rs1530439 | T | G | 0.0101 | | 0.0010 | 4.90E-23 | |  |  |  |
| rs1782652 | A | T | -0.0117 | | 0.0010 | 1.70E-36 | |  |  |  |
| rs11202594 | A | G | -0.0061 | | 0.0013 | 4.00E-08 | |  |  |  |
| rs11187142 | C | T | 0.0078 | | 0.0015 | 3.20E-08 | |  |  |  |
| rs4300303 | T | C | 0.0056 | | 0.0009 | 1.30E-08 | |  |  |  |
| rs145931818 | A | G | 0.0306 | | 0.0037 | 1.20E-16 | |  |  |  |
| rs11815167 | C | A | -0.0409 | | 0.0060 | 6.00E-12 | |  |  |  |
| rs33999979 | G | A | 0.0127 | | 0.0015 | 9.00E-18 | |  |  |  |
| rs2862954 | C | T | 0.0100 | | 0.0009 | 3.20E-24 | |  |  |  |
| rs10822130 | A | G | -0.0202 | | 0.0011 | 7.50E-88 | |  |  |  |
| rs61856602 | G | A | 0.0056 | | 0.0010 | 4.00E-08 | |  |  |  |
| rs12263369 | T | C | -0.0105 | | 0.0009 | 5.00E-30 | |  |  |  |
| rs117589665 | G | A | -0.0151 | | 0.0018 | 5.80E-17 | |  |  |  |
| rs112672290 | G | A | -0.0128 | | 0.0017 | 1.10E-14 | |  |  |  |
| rs11188604 | G | T | 0.0077 | | 0.0010 | 2.60E-16 | |  |  |  |
| rs1561442 | A | G | -0.0075 | | 0.0014 | 1.80E-08 | |  |  |  |
| rs117692193 | G | A | 0.0394 | | 0.0058 | 2.60E-12 | |  |  |  |
| rs188949713 | G | A | 0.0130 | | 0.0022 | 5.90E-10 | |  |  |  |
| rs3737178 | G | A | -0.0119 | | 0.0022 | 2.00E-09 | |  |  |  |
| rs61854630 | A | G | 0.0058 | | 0.0012 | 4.00E-08 | |  |  |  |
| rs16934748 | C | T | -0.0069 | | 0.0013 | 2.80E-08 | |  |  |  |
| rs61853560 | A | G | 0.0303 | | 0.0035 | 1.20E-19 | |  |  |  |
| rs145696003 | T | C | 0.0300 | | 0.0047 | 2.00E-09 | |  |  |  |
| rs112201601 | C | T | 0.0275 | | 0.0046 | 1.60E-09 | |  |  |  |
| rs141899843 | T | C | 0.0291 | | 0.0044 | 3.90E-11 | |  |  |  |
| rs608300 | T | C | 0.0073 | | 0.0010 | 7.90E-14 | |  |  |  |
| rs10824742 | G | A | -0.0052 | | 0.0009 | 1.60E-08 | |  |  |  |
| rs1772189 | A | T | -0.0115 | | 0.0009 | 6.70E-39 | |  |  |  |
| rs7899096 | T | G | -0.0086 | | 0.0010 | 1.20E-17 | |  |  |  |
| rs11030100 | T | G | 0.0063 | | 0.0012 | 3.40E-08 | |  |  |  |
| rs7944853 | A | G | -0.0215 | | 0.0037 | 4.20E-09 | |  |  |  |
| rs11029441 | C | T | -0.0098 | | 0.0017 | 5.40E-09 | |  |  |  |
| rs174554 | G | A | -0.0104 | | 0.0010 | 1.00E-26 | |  |  |  |
| rs3741368 | A | G | 0.0065 | | 0.0009 | 4.30E-12 | |  |  |  |
| rs11607762 | A | T | -0.0112 | | 0.0021 | 1.60E-08 | |  |  |  |
| rs2450128 | A | G | 0.0078 | | 0.0013 | 4.80E-09 | |  |  |  |
| rs11038673 | T | G | -0.0054 | | 0.0010 | 1.70E-09 | |  |  |  |
| rs115352086 | G | C | -0.0176 | | 0.0029 | 1.30E-09 | |  |  |  |
| rs17592998 | T | C | -0.0094 | | 0.0016 | 1.50E-09 | |  |  |  |
| rs55771168 | C | T | 0.0081 | | 0.0011 | 8.40E-16 | |  |  |  |
| rs2156804 | G | T | 0.0049 | | 0.0009 | 8.50E-09 | |  |  |  |
| rs6486122 | T | C | -0.0138 | | 0.0010 | 1.50E-44 | |  |  |  |
| rs4309185 | T | C | -0.0099 | | 0.0014 | 5.80E-13 | |  |  |  |
| rs11601507 | A | C | 0.0154 | | 0.0018 | 3.40E-18 | |  |  |  |
| rs11564722 | T | C | 0.0112 | | 0.0011 | 2.00E-25 | |  |  |  |
| rs10838681 | A | G | 0.0076 | | 0.0011 | 4.90E-13 | |  |  |  |
| rs143709973 | C | A | -0.0119 | | 0.0023 | 2.80E-08 | |  |  |  |
| rs2277283 | C | T | 0.0063 | | 0.0010 | 6.20E-10 | |  |  |  |
| rs12575636 | G | T | -0.0100 | | 0.0012 | 4.70E-17 | |  |  |  |
| rs7131509 | T | C | -0.0082 | | 0.0010 | 1.60E-18 | |  |  |  |
| rs10895276 | T | C | -0.0084 | | 0.0010 | 5.00E-18 | |  |  |  |
| rs512715 | G | C | 0.0085 | | 0.0011 | 2.30E-19 | |  |  |  |
| rs2459974 | T | C | 0.0056 | | 0.0010 | 1.80E-08 | |  |  |  |
| rs138526953 | A | C | -0.0190 | | 0.0033 | 9.40E-10 | |  |  |  |
| rs10794307 | T | C | -0.0065 | | 0.0010 | 1.20E-11 | |  |  |  |
| rs75713100 | G | T | -0.0100 | | 0.0016 | 3.00E-10 | |  |  |  |
| rs12280075 | G | T | 0.0104 | | 0.0010 | 4.80E-27 | |  |  |  |
| rs11032076 | T | C | -0.0070 | | 0.0013 | 2.50E-08 | |  |  |  |
| rs2351958 | A | C | -0.0104 | | 0.0010 | 2.20E-31 | |  |  |  |
| rs12797706 | A | G | 0.0132 | | 0.0011 | 1.00E-31 | |  |  |  |
| rs7123361 | G | A | 0.0064 | | 0.0010 | 2.00E-10 | |  |  |  |
| rs7117818 | T | C | 0.0154 | | 0.0011 | 1.90E-46 | |  |  |  |
| rs78312641 | A | T | 0.0090 | | 0.0014 | 2.00E-10 | |  |  |  |
| rs148118632 | G | A | 0.0233 | | 0.0032 | 1.50E-13 | |  |  |  |
| rs12287551 | T | A | -0.0088 | | 0.0013 | 8.70E-10 | |  |  |  |
| rs62618693 | T | C | 0.0174 | | 0.0022 | 2.10E-15 | |  |  |  |
| rs2121650 | A | G | -0.0058 | | 0.0010 | 2.20E-09 | |  |  |  |
| rs73079476 | C | A | -0.0297 | | 0.0013 | 3.70E-120 | |  |  |  |
| rs75130744 | C | G | -0.0278 | | 0.0018 | 3.60E-58 | |  |  |  |
| rs78458381 | A | T | -0.0063 | | 0.0010 | 9.10E-12 | |  |  |  |
| rs933306 | T | A | 0.0098 | | 0.0011 | 8.40E-18 | |  |  |  |
| rs12302952 | A | G | -0.0120 | | 0.0016 | 2.00E-13 | |  |  |  |
| rs76475417 | A | G | -0.0150 | | 0.0028 | 3.20E-08 | |  |  |  |
| rs2393791 | T | C | 0.0155 | | 0.0010 | 7.10E-61 | |  |  |  |
| rs7953508 | T | C | -0.0058 | | 0.0011 | 3.00E-08 | |  |  |  |
| rs78581485 | C | G | -0.0596 | | 0.0047 | 4.10E-42 | |  |  |  |
| rs6538389 | A | T | -0.0077 | | 0.0011 | 5.40E-11 | |  |  |  |
| rs73375029 | A | G | -0.0104 | | 0.0019 | 2.10E-11 | |  |  |  |
| rs55974289 | T | C | -0.0085 | | 0.0013 | 2.90E-10 | |  |  |  |
| rs10880872 | C | T | -0.0072 | | 0.0011 | 4.70E-12 | |  |  |  |
| rs118080406 | G | A | 0.0182 | | 0.0030 | 3.40E-09 | |  |  |  |
| rs1993669 | T | C | -0.0096 | | 0.0016 | 1.50E-08 | |  |  |  |
| rs56365029 | G | A | -0.0178 | | 0.0032 | 8.20E-09 | |  |  |  |
| rs12311848 | G | A | 0.0081 | | 0.0010 | 1.30E-18 | |  |  |  |
| rs112575738 | T | C | -0.0225 | | 0.0026 | 2.40E-17 | |  |  |  |
| rs78536975 | A | G | 0.0127 | | 0.0024 | 7.80E-09 | |  |  |  |
| rs7314285 | G | T | 0.0346 | | 0.0018 | 5.40E-82 | |  |  |  |
| rs10492118 | T | C | 0.0067 | | 0.0012 | 4.50E-09 | |  |  |  |
| rs78973091 | T | C | -0.0192 | | 0.0032 | 1.80E-09 | |  |  |  |
| rs4584635 | T | A | -0.0146 | | 0.0009 | 1.70E-56 | |  |  |  |
| rs10773049 | C | T | 0.0075 | | 0.0010 | 1.80E-15 | |  |  |  |
| rs3782735 | A | G | 0.0087 | | 0.0010 | 2.70E-18 | |  |  |  |
| rs11110390 | T | C | -0.0089 | | 0.0010 | 7.80E-20 | |  |  |  |
| rs77597993 | A | G | -0.0152 | | 0.0022 | 7.00E-13 | |  |  |  |
| rs2122982 | A | G | 0.0158 | | 0.0011 | 4.00E-50 | |  |  |  |
| rs11111274 | A | G | -0.0087 | | 0.0010 | 5.20E-18 | |  |  |  |
| rs531321977 | A | G | 0.0188 | | 0.0014 | 6.60E-41 | |  |  |  |
| rs531760844 | G | A | 0.0166 | | 0.0025 | 5.00E-11 | |  |  |  |
| rs12424336 | G | A | 0.0065 | | 0.0010 | 5.70E-11 | |  |  |  |
| rs12818938 | G | T | -0.0106 | | 0.0013 | 1.30E-16 | |  |  |  |
| rs3764002 | T | C | 0.0057 | | 0.0011 | 1.40E-08 | |  |  |  |
| rs36124182 | G | A | -0.0259 | | 0.0025 | 7.10E-27 | |  |  |  |
| rs73223295 | C | A | 0.0135 | | 0.0022 | 7.10E-09 | |  |  |  |
| rs7308634 | T | C | -0.0123 | | 0.0014 | 8.20E-21 | |  |  |  |
| rs555038236 | G | A | -0.0246 | | 0.0036 | 3.50E-13 | |  |  |  |
| rs73047887 | C | T | -0.0067 | | 0.0011 | 3.00E-10 | |  |  |  |
| rs864899 | G | A | -0.0116 | | 0.0010 | 2.20E-35 | |  |  |  |
| rs56196860 | A | C | 0.0201 | | 0.0027 | 2.50E-14 | |  |  |  |
| rs34325 | C | T | 0.0049 | | 0.0009 | 1.10E-08 | |  |  |  |
| rs7298924 | T | C | 0.0116 | | 0.0012 | 7.50E-25 | |  |  |  |
| rs11045472 | A | T | 0.0085 | | 0.0010 | 1.10E-17 | |  |  |  |
| rs11108061 | T | C | 0.0059 | | 0.0009 | 1.50E-09 | |  |  |  |
| rs12809946 | G | T | 0.0055 | | 0.0009 | 1.50E-08 | |  |  |  |
| rs1042725 | T | C | -0.0081 | | 0.0009 | 3.60E-19 | |  |  |  |
| rs74581461 | T | A | 0.0151 | | 0.0027 | 1.40E-08 | |  |  |  |
| rs7301634 | G | A | 0.0076 | | 0.0012 | 5.80E-11 | |  |  |  |
| rs184304 | C | T | 0.0075 | | 0.0014 | 7.00E-09 | |  |  |  |
| rs3217860 | G | A | -0.0066 | | 0.0011 | 3.20E-10 | |  |  |  |
| rs2535404 | T | C | 0.0058 | | 0.0010 | 9.30E-10 | |  |  |  |
| rs76895963 | G | T | 0.0651 | | 0.0036 | 4.30E-79 | |  |  |  |
| rs111289824 | G | A | 0.0172 | | 0.0026 | 1.50E-12 | |  |  |  |
| rs4762962 | A | G | 0.0067 | | 0.0010 | 3.40E-10 | |  |  |  |
| rs183015141 | A | G | -0.0218 | | 0.0030 | 8.60E-15 | |  |  |  |
| rs536338 | G | C | -0.0079 | | 0.0010 | 4.50E-16 | |  |  |  |
| rs9597811 | A | G | -0.0055 | | 0.0010 | 6.20E-09 | |  |  |  |
| rs2812208 | C | G | 0.0282 | | 0.0032 | 1.00E-20 | |  |  |  |
| rs3116625 | T | C | -0.0068 | | 0.0012 | 4.40E-10 | |  |  |  |
| rs9556403 | G | A | 0.0084 | | 0.0010 | 5.20E-20 | |  |  |  |
| rs112035922 | T | C | -0.0073 | | 0.0011 | 1.20E-11 | |  |  |  |
| rs4381470 | T | C | 0.0068 | | 0.0012 | 1.90E-09 | |  |  |  |
| rs7323372 | C | T | -0.0051 | | 0.0010 | 2.10E-08 | |  |  |  |
| rs8001781 | G | A | -0.0056 | | 0.0010 | 9.00E-09 | |  |  |  |
| rs9533843 | A | G | 0.0059 | | 0.0009 | 1.30E-10 | |  |  |  |
| rs35812759 | G | A | 0.0123 | | 0.0011 | 1.50E-33 | |  |  |  |
| rs11621792 | T | C | -0.0183 | | 0.0009 | 6.90E-90 | |  |  |  |
| rs2239222 | G | A | 0.0098 | | 0.0010 | 7.80E-23 | |  |  |  |
| rs1951244 | G | A | -0.0062 | | 0.0011 | 1.50E-10 | |  |  |  |
| rs17751614 | T | C | -0.0076 | | 0.0015 | 2.20E-08 | |  |  |  |
| rs28929474 | T | C | 0.1010 | | 0.0033 | 1.00E-200 | |  |  |  |
| rs6118 | T | C | -0.0126 | | 0.0016 | 5.80E-18 | |  |  |  |
| rs362413 | C | T | -0.0084 | | 0.0016 | 4.70E-08 | |  |  |  |
| rs35102588 | C | T | -0.0089 | | 0.0009 | 5.80E-23 | |  |  |  |
| rs3742366 | C | T | 0.0057 | | 0.0010 | 1.50E-10 | |  |  |  |
| rs147259681 | T | C | -0.0190 | | 0.0031 | 6.00E-10 | |  |  |  |
| rs12879423 | G | A | -0.0089 | | 0.0010 | 1.30E-18 | |  |  |  |
| rs17580 | A | T | 0.0259 | | 0.0022 | 3.80E-35 | |  |  |  |
| rs2498786 | G | C | -0.0109 | | 0.0010 | 1.40E-30 | |  |  |  |
| rs6575439 | C | T | -0.0059 | | 0.0010 | 3.80E-09 | |  |  |  |
| rs13379043 | C | T | 0.0095 | | 0.0011 | 2.30E-21 | |  |  |  |
| rs34029605 | A | T | -0.0069 | | 0.0013 | 3.60E-08 | |  |  |  |
| rs1005421 | C | T | 0.0075 | | 0.0010 | 4.10E-17 | |  |  |  |
| rs72683923 | C | T | 0.0338 | | 0.0033 | 3.20E-26 | |  |  |  |
| rs7157184 | G | A | 0.0063 | | 0.0010 | 9.70E-12 | |  |  |  |
| rs68062403 | G | A | -0.0054 | | 0.0010 | 1.90E-08 | |  |  |  |
| rs139974673 | C | T | -0.0609 | | 0.0030 | 1.50E-96 | |  |  |  |
| rs76549335 | C | T | -0.0244 | | 0.0043 | 1.30E-08 | |  |  |  |
| rs35951728 | G | C | -0.0076 | | 0.0009 | 1.60E-17 | |  |  |  |
| rs744200 | T | C | -0.0055 | | 0.0010 | 4.00E-08 | |  |  |  |
| rs72767773 | C | T | 0.0132 | | 0.0019 | 5.90E-13 | |  |  |  |
| rs11856606 | A | G | 0.0090 | | 0.0014 | 1.30E-10 | |  |  |  |
| rs72753908 | T | C | -0.0110 | | 0.0018 | 1.40E-08 | |  |  |  |
| rs7183456 | G | A | 0.0059 | | 0.0010 | 4.30E-10 | |  |  |  |
| rs8043101 | T | C | -0.0236 | | 0.0032 | 2.70E-15 | |  |  |  |
| rs8031716 | C | T | 0.0060 | | 0.0010 | 1.10E-11 | |  |  |  |
| rs61735385 | C | G | -0.0264 | | 0.0015 | 2.20E-73 | |  |  |  |
| rs117411982 | A | C | 0.0221 | | 0.0038 | 1.80E-09 | |  |  |  |
| rs79391862 | C | A | -0.0830 | | 0.0040 | 4.20E-99 | |  |  |  |
| rs17184382 | C | A | 0.0124 | | 0.0009 | 3.80E-41 | |  |  |  |
| rs56332871 | A | C | 0.0341 | | 0.0011 | 1.00E-200 | |  |  |  |
| rs11633147 | C | T | -0.0060 | | 0.0011 | 7.70E-09 | |  |  |  |
| rs62012946 | G | T | -0.0092 | | 0.0016 | 2.40E-09 | |  |  |  |
| rs115177602 | T | A | 0.0227 | | 0.0039 | 1.30E-08 | |  |  |  |
| rs28562483 | T | G | -0.0104 | | 0.0014 | 2.80E-12 | |  |  |  |
| rs55754498 | T | C | 0.0147 | | 0.0021 | 1.80E-12 | |  |  |  |
| rs142035705 | T | C | -0.0270 | | 0.0046 | 1.20E-08 | |  |  |  |
| rs55800572 | G | C | 0.0085 | | 0.0010 | 1.60E-18 | |  |  |  |
| rs3848119 | G | A | 0.0049 | | 0.0010 | 4.10E-08 | |  |  |  |
| rs6495962 | T | C | -0.0049 | | 0.0010 | 3.40E-08 | |  |  |  |
| rs619526 | A | G | 0.0093 | | 0.0013 | 2.60E-13 | |  |  |  |
| rs484943 | C | T | 0.0065 | | 0.0010 | 5.10E-10 | |  |  |  |
| rs11856926 | A | G | -0.0102 | | 0.0009 | 7.20E-29 | |  |  |  |
| rs10163091 | T | C | 0.0097 | | 0.0012 | 4.60E-16 | |  |  |  |
| rs671948 | C | A | -0.0097 | | 0.0010 | 6.50E-24 | |  |  |  |
| rs7164175 | A | G | 0.0138 | | 0.0027 | 1.00E-08 | |  |  |  |
| rs3848125 | A | G | 0.0057 | | 0.0009 | 1.70E-09 | |  |  |  |
| rs720130 | T | G | -0.0059 | | 0.0010 | 2.90E-10 | |  |  |  |
| rs750155 | T | C | -0.0056 | | 0.0010 | 8.50E-09 | |  |  |  |
| rs4782568 | G | C | 0.0120 | | 0.0009 | 2.80E-38 | |  |  |  |
| rs12928099 | A | C | 0.0099 | | 0.0010 | 1.40E-23 | |  |  |  |
| rs56292801 | A | G | 0.0088 | | 0.0011 | 1.50E-18 | |  |  |  |
| rs12325400 | G | C | -0.0069 | | 0.0010 | 7.40E-14 | |  |  |  |
| rs3743588 | A | G | -0.0083 | | 0.0010 | 4.50E-18 | |  |  |  |
| rs35391516 | A | G | 0.0102 | | 0.0017 | 1.50E-09 | |  |  |  |
| rs8049669 | T | A | 0.0054 | | 0.0010 | 3.70E-09 | |  |  |  |
| rs9972653 | T | G | -0.0089 | | 0.0010 | 7.30E-20 | |  |  |  |
| rs246192 | C | G | -0.0074 | | 0.0009 | 9.40E-17 | |  |  |  |
| rs2925979 | C | T | 0.0070 | | 0.0010 | 5.80E-14 | |  |  |  |
| rs12926107 | G | A | -0.0114 | | 0.0009 | 1.80E-36 | |  |  |  |
| rs62037803 | T | C | -0.0083 | | 0.0013 | 5.90E-10 | |  |  |  |
| rs841194 | A | G | -0.0099 | | 0.0012 | 4.50E-17 | |  |  |  |
| rs74998771 | A | G | 0.0150 | | 0.0027 | 1.90E-08 | |  |  |  |
| rs61733486 | T | C | -0.0116 | | 0.0020 | 2.10E-09 | |  |  |  |
| rs188889872 | T | C | 0.1003 | | 0.0041 | 5.11E-139 | |  |  |  |
| rs4297769 | A | G | -0.0114 | | 0.0009 | 7.70E-34 | |  |  |  |
| rs434325 | C | A | 0.0047 | | 0.0009 | 3.50E-08 | |  |  |  |
| rs11078681 | C | T | -0.0205 | | 0.0028 | 1.70E-14 | |  |  |  |
| rs17669311 | A | G | -0.0090 | | 0.0009 | 1.10E-22 | |  |  |  |
| rs79875164 | G | A | -0.0223 | | 0.0029 | 8.10E-16 | |  |  |  |
| rs4794008 | T | C | 0.0091 | | 0.0010 | 1.50E-21 | |  |  |  |
| rs150895955 | A | C | -0.0091 | | 0.0017 | 3.70E-08 | |  |  |  |
| rs740516 | G | C | -0.0094 | | 0.0013 | 1.10E-12 | |  |  |  |
| rs11078597 | C | T | 0.0178 | | 0.0012 | 8.10E-55 | |  |  |  |
| rs61759532 | T | C | 0.0200 | | 0.0011 | 5.90E-84 | |  |  |  |
| rs72840987 | T | C | 0.0146 | | 0.0025 | 7.70E-09 | |  |  |  |
| rs111604078 | A | G | 0.0229 | | 0.0038 | 6.80E-10 | |  |  |  |
| rs183855978 | C | G | -0.0972 | | 0.0031 | 1.00E-200 | |  |  |  |
| rs11078701 | T | C | -0.1160 | | 0.0035 | 1.00E-200 | |  |  |  |
| rs117355285 | A | G | 0.0346 | | 0.0053 | 1.60E-11 | |  |  |  |
| rs62623385 | T | A | -0.0851 | | 0.0025 | 1.00E-200 | |  |  |  |
| rs73972648 | G | A | 0.0181 | | 0.0029 | 1.60E-09 | |  |  |  |
| rs78975791 | T | A | 0.0062 | | 0.0010 | 8.30E-10 | |  |  |  |
| rs11079872 | T | C | 0.0182 | | 0.0016 | 1.30E-30 | |  |  |  |
| rs8074363 | C | T | 0.0071 | | 0.0011 | 4.40E-10 | |  |  |  |
| rs12601778 | T | C | -0.0062 | | 0.0009 | 5.20E-12 | |  |  |  |
| rs2233364 | T | C | 0.0207 | | 0.0027 | 2.50E-15 | |  |  |  |
| rs12185242 | C | A | -0.0313 | | 0.0009 | 1.00E-200 | |  |  |  |
| rs10153315 | C | T | -0.0080 | | 0.0009 | 5.80E-20 | |  |  |  |
| rs76708468 | C | T | -0.0133 | | 0.0023 | 2.20E-08 | |  |  |  |
| rs1801689 | C | A | -0.0372 | | 0.0027 | 2.60E-45 | |  |  |  |
| rs9893194 | G | T | -0.0075 | | 0.0009 | 1.10E-18 | |  |  |  |
| rs7406661 | C | T | 0.0221 | | 0.0011 | 4.60E-89 | |  |  |  |
| rs72842808 | T | C | -0.0157 | | 0.0015 | 1.60E-26 | |  |  |  |
| rs144989856 | A | G | -0.0853 | | 0.0051 | 1.00E-61 | |  |  |  |
| rs78555071 | C | T | -0.0712 | | 0.0020 | 1.00E-200 | |  |  |  |
| rs35547626 | C | T | 0.0350 | | 0.0010 | 1.00E-200 | |  |  |  |
| rs7221716 | G | A | -0.0067 | | 0.0013 | 2.80E-08 | |  |  |  |
| rs2631308 | T | A | 0.0076 | | 0.0013 | 1.70E-09 | |  |  |  |
| rs79600740 | A | C | -0.0158 | | 0.0023 | 4.30E-12 | |  |  |  |
| rs4338849 | G | A | -0.0148 | | 0.0009 | 9.00E-61 | |  |  |  |
| rs9913535 | G | C | -0.0072 | | 0.0012 | 2.00E-10 | |  |  |  |
| rs62078692 | G | C | 0.0058 | | 0.0009 | 1.70E-10 | |  |  |  |
| rs72844546 | T | C | -0.0099 | | 0.0010 | 5.50E-27 | |  |  |  |
| rs139805419 | T | C | 0.0237 | | 0.0036 | 3.80E-12 | |  |  |  |
| rs113523273 | C | A | -0.0182 | | 0.0032 | 3.60E-09 | |  |  |  |
| rs5017726 | C | A | -0.0078 | | 0.0011 | 5.50E-13 | |  |  |  |
| rs145046801 | C | G | -0.0369 | | 0.0017 | 5.30E-110 | |  |  |  |
| rs10432029 | G | A | -0.0488 | | 0.0013 | 1.00E-200 | |  |  |  |
| rs142331290 | G | A | -0.0859 | | 0.0036 | 4.10E-129 | |  |  |  |
| rs2279620 | C | G | 0.0172 | | 0.0014 | 1.60E-35 | |  |  |  |
| rs591939 | G | A | -0.0090 | | 0.0010 | 9.30E-19 | |  |  |  |
| rs12943365 | G | C | 0.0105 | | 0.0009 | 6.20E-31 | |  |  |  |
| rs17881850 | A | G | 0.0774 | | 0.0045 | 9.80E-67 | |  |  |  |
| rs200995850 | A | G | -0.0280 | | 0.0013 | 2.10E-107 | |  |  |  |
| rs8069105 | G | T | 0.0091 | | 0.0010 | 8.00E-20 | |  |  |  |
| rs9902384 | A | G | 0.0070 | | 0.0011 | 3.20E-11 | |  |  |  |
| rs78496430 | G | A | 0.0735 | | 0.0020 | 1.00E-200 | |  |  |  |
| rs113364399 | A | G | -0.0129 | | 0.0024 | 4.80E-08 | |  |  |  |
| rs55910553 | A | G | -0.0505 | | 0.0025 | 1.20E-92 | |  |  |  |
| rs8079418 | T | C | -0.0129 | | 0.0009 | 7.60E-44 | |  |  |  |
| rs659377 | C | G | -0.0180 | | 0.0030 | 2.10E-10 | |  |  |  |
| rs8077323 | T | C | 0.0063 | | 0.0011 | 2.00E-09 | |  |  |  |
| rs2277641 | A | G | -0.0287 | | 0.0031 | 5.50E-22 | |  |  |  |
| rs199851449 | C | A | -0.0121 | | 0.0019 | 8.70E-11 | |  |  |  |
| rs8066941 | G | T | -0.0122 | | 0.0011 | 2.50E-31 | |  |  |  |
| rs11552708 | A | G | 0.0347 | | 0.0014 | 2.50E-143 | |  |  |  |
| rs11664106 | T | A | 0.0061 | | 0.0010 | 4.60E-10 | |  |  |  |
| rs12965052 | A | G | -0.0056 | | 0.0010 | 1.40E-09 | |  |  |  |
| rs10084025 | T | C | 0.0058 | | 0.0010 | 1.20E-09 | |  |  |  |
| rs6567160 | C | T | -0.0098 | | 0.0011 | 1.20E-19 | |  |  |  |
| rs6567230 | A | T | -0.0055 | | 0.0010 | 4.40E-08 | |  |  |  |
| rs3810027 | G | C | -0.0071 | | 0.0010 | 2.00E-13 | |  |  |  |
| rs4092465 | G | A | 0.0092 | | 0.0010 | 3.90E-22 | |  |  |  |
| rs12454712 | C | T | 0.0072 | | 0.0010 | 1.40E-15 | |  |  |  |
| rs3829639 | G | A | -0.0074 | | 0.0010 | 1.10E-13 | |  |  |  |
| rs8087306 | C | G | -0.0056 | | 0.0010 | 4.20E-09 | |  |  |  |
| rs55737395 | A | G | 0.0052 | | 0.0010 | 1.10E-08 | |  |  |  |
| rs117169274 | A | G | -0.0175 | | 0.0030 | 5.50E-11 | |  |  |  |
| rs14129 | A | G | -0.0118 | | 0.0014 | 6.60E-16 | |  |  |  |
| rs10413329 | G | A | 0.0051 | | 0.0009 | 1.80E-08 | |  |  |  |
| rs901886 | C | T | 0.0055 | | 0.0009 | 8.30E-09 | |  |  |  |
| rs10411893 | G | C | 0.0066 | | 0.0010 | 2.40E-12 | |  |  |  |
| rs885683 | A | G | -0.0106 | | 0.0011 | 1.70E-26 | |  |  |  |
| rs2965196 | A | G | -0.0149 | | 0.0023 | 1.70E-10 | |  |  |  |
| rs73597479 | T | C | -0.0319 | | 0.0025 | 7.10E-39 | |  |  |  |
| rs2972438 | T | C | 0.0067 | | 0.0012 | 4.30E-08 | |  |  |  |
| rs34010237 | A | G | -0.0075 | | 0.0013 | 2.50E-10 | |  |  |  |
| rs1007851 | T | C | 0.0127 | | 0.0016 | 1.40E-14 | |  |  |  |
| rs140105410 | A | G | -0.0259 | | 0.0030 | 1.80E-20 | |  |  |  |
| rs45512696 | T | C | 0.0201 | | 0.0012 | 1.50E-64 | |  |  |  |
| rs62111692 | T | G | -0.0135 | | 0.0024 | 1.40E-08 | |  |  |  |
| rs59708898 | T | C | -0.0106 | | 0.0020 | 4.60E-08 | |  |  |  |
| rs10411932 | C | T | -0.0054 | | 0.0009 | 7.80E-10 | |  |  |  |
| rs11545185 | A | G | 0.0088 | | 0.0013 | 2.00E-14 | |  |  |  |
| rs5112 | G | C | 0.0073 | | 0.0010 | 1.10E-14 | |  |  |  |
| rs111981233 | G | T | 0.0210 | | 0.0017 | 2.40E-37 | |  |  |  |
| rs7250869 | C | T | 0.0079 | | 0.0010 | 2.80E-17 | |  |  |  |
| rs483082 | T | G | 0.0110 | | 0.0011 | 1.40E-23 | |  |  |  |
| rs2914005 | C | T | 0.0069 | | 0.0011 | 3.40E-10 | |  |  |  |
| rs116573491 | A | G | 0.0050 | | 0.0010 | 2.80E-08 | |  |  |  |
| rs350832 | A | G | -0.0076 | | 0.0011 | 2.70E-11 | |  |  |  |
| rs8107967 | G | A | 0.0077 | | 0.0009 | 8.90E-17 | |  |  |  |
| rs8101895 | T | A | -0.0069 | | 0.0011 | 3.10E-11 | |  |  |  |
| rs1640269 | C | A | 0.0195 | | 0.0010 | 2.10E-85 | |  |  |  |
| rs11539938 | C | T | 0.0094 | | 0.0010 | 6.90E-21 | |  |  |  |
| rs60018147 | G | A | 0.0133 | | 0.0015 | 1.00E-19 | |  |  |  |
| rs7252372 | C | G | -0.0087 | | 0.0010 | 2.30E-22 | |  |  |  |
| rs202200760 | C | G | 0.0748 | | 0.0026 | 7.91E-191 | |  |  |  |
| rs8106047 | A | T | -0.0077 | | 0.0013 | 1.10E-08 | |  |  |  |
| rs113251204 | C | T | -0.0107 | | 0.0018 | 4.50E-11 | |  |  |  |
| rs66921136 | C | T | 0.0097 | | 0.0010 | 1.00E-20 | |  |  |  |
| rs4147913 | T | C | -0.0061 | | 0.0010 | 9.70E-12 | |  |  |  |
| rs6510692 | G | C | -0.0062 | | 0.0011 | 1.30E-08 | |  |  |  |
| rs17356664 | T | C | -0.0069 | | 0.0010 | 2.60E-11 | |  |  |  |
| rs34255979 | T | C | 0.0271 | | 0.0014 | 9.10E-82 | |  |  |  |
| rs10416080 | A | G | -0.0118 | | 0.0016 | 8.90E-15 | |  |  |  |
| rs61599759 | G | A | -0.0081 | | 0.0011 | 1.20E-13 | |  |  |  |
| rs17207107 | C | A | 0.0052 | | 0.0009 | 5.60E-09 | |  |  |  |
| rs4804414 | T | C | -0.0146 | | 0.0009 | 1.20E-58 | |  |  |  |
| rs2618566 | T | G | -0.0074 | | 0.0010 | 5.50E-14 | |  |  |  |
| rs6018424 | T | C | 0.0077 | | 0.0012 | 4.70E-11 | |  |  |  |
| rs2746829 | T | C | -0.0063 | | 0.0010 | 4.40E-10 | |  |  |  |
| rs6062381 | G | A | -0.0050 | | 0.0009 | 3.50E-08 | |  |  |  |
| rs7262150 | C | T | 0.0093 | | 0.0011 | 1.00E-17 | |  |  |  |
| rs34587839 | A | G | -0.0133 | | 0.0013 | 1.00E-25 | |  |  |  |
| rs6129802 | T | C | 0.0115 | | 0.0011 | 3.60E-24 | |  |  |  |
| rs6073431 | T | C | 0.0171 | | 0.0010 | 1.60E-73 | |  |  |  |
| rs55966194 | G | C | 0.0062 | | 0.0010 | 2.40E-10 | |  |  |  |
| rs4812336 | A | G | 0.0060 | | 0.0010 | 1.20E-09 | |  |  |  |
| rs4811050 | A | G | -0.0074 | | 0.0012 | 1.80E-09 | |  |  |  |
| rs6120663 | A | C | -0.0088 | | 0.0009 | 8.70E-23 | |  |  |  |
| rs736820 | A | G | -0.0059 | | 0.0010 | 8.80E-12 | |  |  |  |
| rs4599176 | T | G | 0.0080 | | 0.0011 | 1.00E-15 | |  |  |  |
| rs78319058 | T | C | 0.0204 | | 0.0032 | 1.20E-11 | |  |  |  |
| rs55987409 | T | C | 0.0165 | | 0.0018 | 7.40E-19 | |  |  |  |
| rs6048205 | G | A | -0.0140 | | 0.0023 | 6.00E-09 | |  |  |  |
| rs7261425 | G | C | 0.0058 | | 0.0010 | 3.00E-08 | |  |  |  |
| rs403694 | T | C | -0.0057 | | 0.0009 | 3.00E-10 | |  |  |  |
| rs8134638 | C | T | -0.0064 | | 0.0010 | 8.50E-12 | |  |  |  |
| rs9614162 | A | G | -0.0055 | | 0.0010 | 3.40E-09 | |  |  |  |
| rs4680 | A | G | -0.0074 | | 0.0009 | 9.40E-17 | |  |  |  |
| rs111700120 | T | C | -0.0084 | | 0.0013 | 4.00E-12 | |  |  |  |
| rs9610329 | T | C | -0.0056 | | 0.0010 | 7.40E-09 | |  |  |  |
| rs13057133 | T | C | -0.0083 | | 0.0010 | 2.60E-17 | |  |  |  |
| rs175184 | C | G | -0.0054 | | 0.0010 | 5.60E-09 | |  |  |  |
| rs2075915 | A | G | 0.0075 | | 0.0011 | 8.80E-12 | |  |  |  |
| rs695272 | C | T | -0.0129 | | 0.0010 | 1.20E-38 | |  |  |  |
| rs759404 | T | C | -0.0107 | | 0.0019 | 3.10E-08 | |  |  |  |
| rs35598889 | C | T | 0.0079 | | 0.0011 | 2.60E-15 | |  |  |  |
| rs5760120 | A | G | -0.0080 | | 0.0009 | 4.30E-18 | |  |  |  |
| rs757869 | A | G | -0.0113 | | 0.0010 | 1.20E-30 | |  |  |  |
| rs738409 | G | C | 0.0245 | | 0.0011 | 4.30E-104 | |  |  |  |
| rs4820091 | G | T | 0.0105 | | 0.0012 | 2.80E-18 | |  |  |  |

SNP, single nucleotide polymorphism; se, standard error; IV, instrumental variable; MR, mendelian randomization

**Table S2** The OR and 95%CI in leave-one-out analysis

| **outcome** | **exposure** | **SNP** | **OR(95%CI)** |
| --- | --- | --- | --- |
| Stroke | SHBG | rs10026753 | 0.87(0.79,0.96) |
| Stroke | SHBG | rs10041660 | 0.87(0.79,0.96) |
| Stroke | SHBG | rs1005421 | 0.87(0.79,0.96) |
| Stroke | SHBG | rs1007851 | 0.87(0.79,0.96) |
| Stroke | SHBG | rs10084025 | 0.87(0.79,0.96) |
| Stroke | SHBG | rs10095103 | 0.87(0.79,0.96) |
| Stroke | SHBG | rs10110651 | 0.87(0.79,0.96) |
| Stroke | SHBG | rs10111451 | 0.87(0.79,0.96) |
| Stroke | SHBG | rs10123811 | 0.87(0.79,0.96) |
| Stroke | SHBG | rs10125995 | 0.87(0.79,0.96) |
| Stroke | SHBG | rs10153315 | 0.87(0.79,0.96) |
| Stroke | SHBG | rs10163091 | 0.87(0.79,0.96) |
| Stroke | SHBG | rs10187560 | 0.87(0.79,0.96) |
| Stroke | SHBG | rs10208512 | 0.87(0.79,0.96) |
| Stroke | SHBG | rs10238028 | 0.87(0.79,0.96) |
| Stroke | SHBG | rs10258433 | 0.87(0.79,0.96) |
| Stroke | SHBG | rs10273476 | 0.87(0.79,0.96) |
| Stroke | SHBG | rs10411932 | 0.87(0.79,0.96) |
| Stroke | SHBG | rs10413329 | 0.87(0.79,0.96) |
| Stroke | SHBG | rs10416080 | 0.87(0.79,0.96) |
| Stroke | SHBG | rs1042725 | 0.87(0.79,0.96) |
| Stroke | SHBG | rs10432029 | 0.88(0.80,0.97) |
| Stroke | SHBG | rs10492118 | 0.87(0.79,0.96) |
| Stroke | SHBG | rs10504731 | 0.87(0.79,0.96) |
| Stroke | SHBG | rs1059698 | 0.87(0.79,0.96) |
| Stroke | SHBG | rs10733608 | 0.87(0.79,0.96) |
| Stroke | SHBG | rs10761676 | 0.87(0.79,0.96) |
| Stroke | SHBG | rs10761749 | 0.88(0.80,0.96) |
| Stroke | SHBG | rs10773049 | 0.87(0.79,0.96) |
| Stroke | SHBG | rs10794307 | 0.87(0.79,0.96) |
| Stroke | SHBG | rs10815276 | 0.87(0.79,0.96) |
| Stroke | SHBG | rs10822130 | 0.87(0.79,0.96) |
| Stroke | SHBG | rs10824742 | 0.87(0.79,0.96) |
| Stroke | SHBG | rs10838681 | 0.87(0.79,0.95) |
| Stroke | SHBG | rs10864070 | 0.87(0.79,0.96) |
| Stroke | SHBG | rs10880872 | 0.87(0.79,0.95) |
| Stroke | SHBG | rs10888696 | 0.87(0.79,0.96) |
| Stroke | SHBG | rs10895276 | 0.87(0.79,0.95) |
| Stroke | SHBG | rs10900446 | 0.87(0.79,0.96) |
| Stroke | SHBG | rs10946313 | 0.87(0.79,0.96) |
| Stroke | SHBG | rs10951130 | 0.87(0.79,0.95) |
| Stroke | SHBG | rs11029441 | 0.87(0.79,0.96) |
| Stroke | SHBG | rs11030100 | 0.87(0.79,0.96) |
| Stroke | SHBG | rs11032076 | 0.87(0.79,0.96) |
| Stroke | SHBG | rs11038673 | 0.87(0.79,0.96) |
| Stroke | SHBG | rs11078597 | 0.87(0.79,0.96) |
| Stroke | SHBG | rs11078681 | 0.87(0.79,0.96) |
| Stroke | SHBG | rs11078701 | 0.87(0.79,0.96) |
| Stroke | SHBG | rs11079872 | 0.87(0.79,0.96) |
| Stroke | SHBG | rs11108061 | 0.87(0.79,0.96) |
| Stroke | SHBG | rs11110390 | 0.87(0.79,0.96) |
| Stroke | SHBG | rs11111274 | 0.87(0.79,0.96) |
| Stroke | SHBG | rs11121522 | 0.87(0.79,0.96) |
| Stroke | SHBG | rs111289824 | 0.87(0.79,0.96) |
| Stroke | SHBG | rs111331455 | 0.87(0.79,0.96) |
| Stroke | SHBG | rs111363146 | 0.87(0.79,0.96) |
| Stroke | SHBG | rs11153046 | 0.87(0.79,0.96) |
| Stroke | SHBG | rs11155787 | 0.87(0.79,0.96) |
| Stroke | SHBG | rs111604078 | 0.87(0.79,0.96) |
| Stroke | SHBG | rs111637026 | 0.87(0.79,0.96) |
| Stroke | SHBG | rs11164095 | 0.87(0.79,0.96) |
| Stroke | SHBG | rs11165493 | 0.87(0.79,0.96) |
| Stroke | SHBG | rs111700120 | 0.87(0.79,0.96) |
| Stroke | SHBG | rs11187142 | 0.87(0.79,0.96) |
| Stroke | SHBG | rs11188604 | 0.87(0.79,0.96) |
| Stroke | SHBG | rs11190245 | 0.87(0.79,0.96) |
| Stroke | SHBG | rs111905890 | 0.87(0.79,0.96) |
| Stroke | SHBG | rs111981233 | 0.87(0.79,0.96) |
| Stroke | SHBG | rs11202594 | 0.87(0.79,0.96) |
| Stroke | SHBG | rs112035922 | 0.87(0.79,0.96) |
| Stroke | SHBG | rs112575738 | 0.87(0.79,0.96) |
| Stroke | SHBG | rs112672290 | 0.87(0.79,0.96) |
| Stroke | SHBG | rs112833123 | 0.87(0.79,0.96) |
| Stroke | SHBG | rs112850234 | 0.87(0.79,0.96) |
| Stroke | SHBG | rs112928223 | 0.87(0.79,0.96) |
| Stroke | SHBG | rs113251204 | 0.87(0.79,0.96) |
| Stroke | SHBG | rs113364399 | 0.87(0.79,0.96) |
| Stroke | SHBG | rs113523273 | 0.87(0.79,0.96) |
| Stroke | SHBG | rs113973451 | 0.87(0.79,0.96) |
| Stroke | SHBG | rs114053844 | 0.87(0.79,0.96) |
| Stroke | SHBG | rs114469183 | 0.87(0.79,0.96) |
| Stroke | SHBG | rs114627598 | 0.87(0.79,0.96) |
| Stroke | SHBG | rs114940462 | 0.87(0.79,0.95) |
| Stroke | SHBG | rs114949263 | 0.87(0.79,0.96) |
| Stroke | SHBG | rs11539938 | 0.87(0.79,0.96) |
| Stroke | SHBG | rs11542663 | 0.87(0.79,0.96) |
| Stroke | SHBG | rs11545185 | 0.87(0.79,0.96) |
| Stroke | SHBG | rs115521489 | 0.87(0.79,0.96) |
| Stroke | SHBG | rs11552708 | 0.86(0.78,0.94) |
| Stroke | SHBG | rs11556924 | 0.87(0.79,0.96) |
| Stroke | SHBG | rs11564722 | 0.87(0.79,0.96) |
| Stroke | SHBG | rs11601507 | 0.87(0.79,0.96) |
| Stroke | SHBG | rs11621792 | 0.87(0.79,0.96) |
| Stroke | SHBG | rs11633147 | 0.87(0.79,0.96) |
| Stroke | SHBG | rs116573491 | 0.87(0.79,0.96) |
| Stroke | SHBG | rs116713089 | 0.87(0.79,0.96) |
| Stroke | SHBG | rs11682084 | 0.87(0.79,0.96) |
| Stroke | SHBG | rs11690176 | 0.87(0.79,0.96) |
| Stroke | SHBG | rs117108573 | 0.87(0.79,0.96) |
| Stroke | SHBG | rs117135073 | 0.87(0.79,0.96) |
| Stroke | SHBG | rs117169274 | 0.87(0.79,0.96) |
| Stroke | SHBG | rs117411982 | 0.87(0.79,0.96) |
| Stroke | SHBG | rs11743810 | 0.87(0.79,0.96) |
| Stroke | SHBG | rs11748938 | 0.87(0.79,0.96) |
| Stroke | SHBG | rs117589665 | 0.87(0.79,0.96) |
| Stroke | SHBG | rs11765639 | 0.87(0.79,0.96) |
| Stroke | SHBG | rs11791747 | 0.87(0.79,0.96) |
| Stroke | SHBG | rs118080406 | 0.87(0.79,0.96) |
| Stroke | SHBG | rs11856606 | 0.87(0.79,0.96) |
| Stroke | SHBG | rs11856926 | 0.87(0.79,0.96) |
| Stroke | SHBG | rs11994858 | 0.87(0.79,0.95) |
| Stroke | SHBG | rs11997548 | 0.87(0.79,0.96) |
| Stroke | SHBG | rs12059956 | 0.87(0.79,0.96) |
| Stroke | SHBG | rs12138461 | 0.87(0.79,0.96) |
| Stroke | SHBG | rs12185242 | 0.87(0.79,0.96) |
| Stroke | SHBG | rs12263369 | 0.87(0.80,0.96) |
| Stroke | SHBG | rs12280075 | 0.87(0.79,0.96) |
| Stroke | SHBG | rs1229498 | 0.87(0.79,0.96) |
| Stroke | SHBG | rs12302952 | 0.87(0.79,0.96) |
| Stroke | SHBG | rs12311848 | 0.87(0.79,0.96) |
| Stroke | SHBG | rs12373799 | 0.87(0.79,0.96) |
| Stroke | SHBG | rs12413488 | 0.87(0.79,0.96) |
| Stroke | SHBG | rs12424336 | 0.87(0.79,0.96) |
| Stroke | SHBG | rs12454712 | 0.87(0.79,0.95) |
| Stroke | SHBG | rs12476661 | 0.87(0.79,0.96) |
| Stroke | SHBG | rs12575636 | 0.87(0.79,0.96) |
| Stroke | SHBG | rs12601778 | 0.87(0.79,0.96) |
| Stroke | SHBG | rs12636106 | 0.87(0.79,0.96) |
| Stroke | SHBG | rs12667888 | 0.87(0.79,0.96) |
| Stroke | SHBG | rs12758998 | 0.87(0.79,0.96) |
| Stroke | SHBG | rs12809946 | 0.87(0.79,0.96) |
| Stroke | SHBG | rs12818938 | 0.87(0.79,0.96) |
| Stroke | SHBG | rs12879423 | 0.87(0.79,0.95) |
| Stroke | SHBG | rs12916 | 0.87(0.79,0.96) |
| Stroke | SHBG | rs12926107 | 0.87(0.80,0.96) |
| Stroke | SHBG | rs12928099 | 0.87(0.79,0.96) |
| Stroke | SHBG | rs12965052 | 0.87(0.79,0.96) |
| Stroke | SHBG | rs12989083 | 0.87(0.79,0.96) |
| Stroke | SHBG | rs13000027 | 0.87(0.79,0.96) |
| Stroke | SHBG | rs13035806 | 0.87(0.79,0.96) |
| Stroke | SHBG | rs13057133 | 0.87(0.79,0.96) |
| Stroke | SHBG | rs13086465 | 0.88(0.80,0.96) |
| Stroke | SHBG | rs13094241 | 0.87(0.79,0.96) |
| Stroke | SHBG | rs13108218 | 0.86(0.78,0.95) |
| Stroke | SHBG | rs13149606 | 0.87(0.79,0.96) |
| Stroke | SHBG | rs13150068 | 0.88(0.80,0.96) |
| Stroke | SHBG | rs13251458 | 0.87(0.79,0.96) |
| Stroke | SHBG | rs1330307 | 0.87(0.79,0.96) |
| Stroke | SHBG | rs13315174 | 0.87(0.79,0.96) |
| Stroke | SHBG | rs13379043 | 0.87(0.79,0.95) |
| Stroke | SHBG | rs13389219 | 0.87(0.79,0.96) |
| Stroke | SHBG | rs13405815 | 0.87(0.79,0.96) |
| Stroke | SHBG | rs1352084 | 0.87(0.79,0.96) |
| Stroke | SHBG | rs138526953 | 0.87(0.79,0.96) |
| Stroke | SHBG | rs138755456 | 0.87(0.79,0.96) |
| Stroke | SHBG | rs139805419 | 0.87(0.79,0.96) |
| Stroke | SHBG | rs139974673 | 0.88(0.80,0.97) |
| Stroke | SHBG | rs140105410 | 0.87(0.79,0.96) |
| Stroke | SHBG | rs1408270 | 0.87(0.79,0.96) |
| Stroke | SHBG | rs1411432 | 0.87(0.79,0.96) |
| Stroke | SHBG | rs14129 | 0.87(0.79,0.96) |
| Stroke | SHBG | rs141899843 | 0.87(0.79,0.96) |
| Stroke | SHBG | rs1420385 | 0.87(0.79,0.96) |
| Stroke | SHBG | rs142331290 | 0.87(0.79,0.96) |
| Stroke | SHBG | rs1431659 | 0.87(0.79,0.96) |
| Stroke | SHBG | rs1433210 | 0.87(0.79,0.96) |
| Stroke | SHBG | rs143709973 | 0.87(0.79,0.96) |
| Stroke | SHBG | rs144459202 | 0.87(0.79,0.96) |
| Stroke | SHBG | rs144989856 | 0.87(0.79,0.96) |
| Stroke | SHBG | rs145931818 | 0.87(0.79,0.96) |
| Stroke | SHBG | rs147259681 | 0.87(0.79,0.96) |
| Stroke | SHBG | rs148118632 | 0.87(0.79,0.96) |
| Stroke | SHBG | rs149092986 | 0.87(0.79,0.96) |
| Stroke | SHBG | rs149102638 | 0.87(0.79,0.96) |
| Stroke | SHBG | rs150895955 | 0.87(0.79,0.96) |
| Stroke | SHBG | rs1530439 | 0.87(0.79,0.96) |
| Stroke | SHBG | rs1534696 | 0.87(0.79,0.96) |
| Stroke | SHBG | rs1561442 | 0.87(0.79,0.96) |
| Stroke | SHBG | rs1570360 | 0.87(0.79,0.96) |
| Stroke | SHBG | rs157935 | 0.87(0.79,0.96) |
| Stroke | SHBG | rs1640269 | 0.87(0.79,0.96) |
| Stroke | SHBG | rs16835135 | 0.87(0.79,0.96) |
| Stroke | SHBG | rs16934748 | 0.87(0.79,0.96) |
| Stroke | SHBG | rs1708302 | 0.87(0.79,0.96) |
| Stroke | SHBG | rs17184382 | 0.87(0.79,0.96) |
| Stroke | SHBG | rs17207107 | 0.87(0.79,0.96) |
| Stroke | SHBG | rs1730862 | 0.87(0.79,0.95) |
| Stroke | SHBG | rs17356664 | 0.87(0.79,0.96) |
| Stroke | SHBG | rs17372936 | 0.87(0.79,0.96) |
| Stroke | SHBG | rs1738380 | 0.87(0.79,0.96) |
| Stroke | SHBG | rs1743954 | 0.87(0.79,0.96) |
| Stroke | SHBG | rs174554 | 0.87(0.79,0.95) |
| Stroke | SHBG | rs17583875 | 0.87(0.79,0.96) |
| Stroke | SHBG | rs17592998 | 0.87(0.79,0.96) |
| Stroke | SHBG | rs17669311 | 0.87(0.79,0.96) |
| Stroke | SHBG | rs1775125 | 0.87(0.79,0.96) |
| Stroke | SHBG | rs17751614 | 0.87(0.79,0.96) |
| Stroke | SHBG | rs17755271 | 0.87(0.79,0.96) |
| Stroke | SHBG | rs17881850 | 0.87(0.79,0.96) |
| Stroke | SHBG | rs1799831 | 0.87(0.79,0.96) |
| Stroke | SHBG | rs1801689 | 0.87(0.80,0.96) |
| Stroke | SHBG | rs182132993 | 0.87(0.79,0.96) |
| Stroke | SHBG | rs1823227 | 0.87(0.79,0.96) |
| Stroke | SHBG | rs182848434 | 0.87(0.79,0.96) |
| Stroke | SHBG | rs183015141 | 0.87(0.79,0.95) |
| Stroke | SHBG | rs184304 | 0.87(0.79,0.96) |
| Stroke | SHBG | rs185406435 | 0.87(0.79,0.96) |
| Stroke | SHBG | rs1864390 | 0.87(0.79,0.96) |
| Stroke | SHBG | rs186766320 | 0.87(0.79,0.96) |
| Stroke | SHBG | rs1883783 | 0.87(0.79,0.96) |
| Stroke | SHBG | rs188889872 | 0.87(0.79,0.96) |
| Stroke | SHBG | rs188949713 | 0.87(0.79,0.96) |
| Stroke | SHBG | rs189595752 | 0.87(0.79,0.96) |
| Stroke | SHBG | rs190712219 | 0.87(0.79,0.96) |
| Stroke | SHBG | rs1951244 | 0.87(0.79,0.96) |
| Stroke | SHBG | rs1969213 | 0.87(0.79,0.96) |
| Stroke | SHBG | rs1993669 | 0.87(0.79,0.95) |
| Stroke | SHBG | rs201570119 | 0.87(0.79,0.96) |
| Stroke | SHBG | rs2022865 | 0.87(0.79,0.96) |
| Stroke | SHBG | rs203777 | 0.87(0.79,0.96) |
| Stroke | SHBG | rs2063245 | 0.87(0.79,0.96) |
| Stroke | SHBG | rs2074683 | 0.87(0.79,0.96) |
| Stroke | SHBG | rs2075915 | 0.87(0.79,0.96) |
| Stroke | SHBG | rs2121650 | 0.87(0.79,0.96) |
| Stroke | SHBG | rs2122982 | 0.87(0.79,0.96) |
| Stroke | SHBG | rs2156804 | 0.87(0.79,0.96) |
| Stroke | SHBG | rs2176887 | 0.87(0.79,0.96) |
| Stroke | SHBG | rs2196943 | 0.87(0.79,0.96) |
| Stroke | SHBG | rs2233364 | 0.87(0.79,0.96) |
| Stroke | SHBG | rs2234922 | 0.87(0.79,0.95) |
| Stroke | SHBG | rs2239222 | 0.87(0.79,0.96) |
| Stroke | SHBG | rs2241261 | 0.87(0.79,0.96) |
| Stroke | SHBG | rs2246223 | 0.87(0.79,0.96) |
| Stroke | SHBG | rs2273368 | 0.87(0.79,0.95) |
| Stroke | SHBG | rs2275355 | 0.87(0.79,0.96) |
| Stroke | SHBG | rs2277283 | 0.87(0.79,0.96) |
| Stroke | SHBG | rs2277641 | 0.87(0.79,0.95) |
| Stroke | SHBG | rs2280838 | 0.87(0.79,0.96) |
| Stroke | SHBG | rs2304686 | 0.87(0.79,0.96) |
| Stroke | SHBG | rs2305144 | 0.87(0.79,0.96) |
| Stroke | SHBG | rs234051 | 0.87(0.79,0.95) |
| Stroke | SHBG | rs2351958 | 0.87(0.79,0.96) |
| Stroke | SHBG | rs237438 | 0.87(0.79,0.96) |
| Stroke | SHBG | rs2393791 | 0.87(0.79,0.96) |
| Stroke | SHBG | rs2404976 | 0.87(0.79,0.96) |
| Stroke | SHBG | rs2431752 | 0.87(0.79,0.95) |
| Stroke | SHBG | rs2450128 | 0.87(0.79,0.96) |
| Stroke | SHBG | rs2522054 | 0.87(0.79,0.96) |
| Stroke | SHBG | rs2535404 | 0.87(0.79,0.95) |
| Stroke | SHBG | rs2551774 | 0.87(0.79,0.96) |
| Stroke | SHBG | rs2602856 | 0.87(0.79,0.96) |
| Stroke | SHBG | rs2618566 | 0.87(0.79,0.96) |
| Stroke | SHBG | rs2627690 | 0.87(0.79,0.96) |
| Stroke | SHBG | rs2642438 | 0.87(0.79,0.96) |
| Stroke | SHBG | rs267733 | 0.87(0.79,0.96) |
| Stroke | SHBG | rs2705619 | 0.87(0.79,0.96) |
| Stroke | SHBG | rs2723067 | 0.87(0.79,0.96) |
| Stroke | SHBG | rs2724475 | 0.87(0.79,0.96) |
| Stroke | SHBG | rs2746829 | 0.87(0.79,0.96) |
| Stroke | SHBG | rs2792022 | 0.87(0.79,0.96) |
| Stroke | SHBG | rs2807861 | 0.87(0.79,0.96) |
| Stroke | SHBG | rs28507491 | 0.88(0.80,0.96) |
| Stroke | SHBG | rs28562483 | 0.87(0.79,0.96) |
| Stroke | SHBG | rs2862954 | 0.87(0.79,0.96) |
| Stroke | SHBG | rs2885582 | 0.87(0.79,0.95) |
| Stroke | SHBG | rs28890929 | 0.87(0.79,0.96) |
| Stroke | SHBG | rs28925904 | 0.87(0.79,0.96) |
| Stroke | SHBG | rs28929474 | 0.87(0.79,0.95) |
| Stroke | SHBG | rs2914005 | 0.87(0.79,0.96) |
| Stroke | SHBG | rs2925979 | 0.87(0.79,0.96) |
| Stroke | SHBG | rs2942202 | 0.87(0.79,0.96) |
| Stroke | SHBG | rs2965196 | 0.87(0.79,0.96) |
| Stroke | SHBG | rs2970877 | 0.87(0.79,0.96) |
| Stroke | SHBG | rs2972145 | 0.87(0.79,0.96) |
| Stroke | SHBG | rs2972438 | 0.87(0.79,0.96) |
| Stroke | SHBG | rs3001032 | 0.87(0.79,0.95) |
| Stroke | SHBG | rs3004179 | 0.87(0.79,0.96) |
| Stroke | SHBG | rs3116625 | 0.87(0.79,0.96) |
| Stroke | SHBG | rs3217860 | 0.87(0.79,0.96) |
| Stroke | SHBG | rs329122 | 0.87(0.79,0.96) |
| Stroke | SHBG | rs33807 | 0.87(0.79,0.96) |
| Stroke | SHBG | rs33999979 | 0.87(0.79,0.96) |
| Stroke | SHBG | rs34010237 | 0.87(0.79,0.96) |
| Stroke | SHBG | rs340835 | 0.87(0.79,0.96) |
| Stroke | SHBG | rs34255979 | 0.87(0.79,0.96) |
| Stroke | SHBG | rs34311866 | 0.87(0.79,0.96) |
| Stroke | SHBG | rs34325 | 0.87(0.79,0.96) |
| Stroke | SHBG | rs34372369 | 0.87(0.79,0.96) |
| Stroke | SHBG | rs34587839 | 0.87(0.79,0.96) |
| Stroke | SHBG | rs34651 | 0.87(0.79,0.96) |
| Stroke | SHBG | rs3468 | 0.87(0.79,0.96) |
| Stroke | SHBG | rs34707604 | 0.87(0.79,0.96) |
| Stroke | SHBG | rs34880012 | 0.87(0.79,0.96) |
| Stroke | SHBG | rs34970607 | 0.87(0.79,0.96) |
| Stroke | SHBG | rs35070405 | 0.87(0.79,0.96) |
| Stroke | SHBG | rs350832 | 0.87(0.80,0.96) |
| Stroke | SHBG | rs35102588 | 0.87(0.80,0.96) |
| Stroke | SHBG | rs35226891 | 0.87(0.79,0.96) |
| Stroke | SHBG | rs35233014 | 0.87(0.79,0.96) |
| Stroke | SHBG | rs35333155 | 0.87(0.79,0.96) |
| Stroke | SHBG | rs35346083 | 0.87(0.80,0.96) |
| Stroke | SHBG | rs35391516 | 0.87(0.79,0.96) |
| Stroke | SHBG | rs35547626 | 0.87(0.79,0.95) |
| Stroke | SHBG | rs35598889 | 0.87(0.79,0.96) |
| Stroke | SHBG | rs35633876 | 0.87(0.79,0.96) |
| Stroke | SHBG | rs35812759 | 0.87(0.79,0.96) |
| Stroke | SHBG | rs359431 | 0.87(0.79,0.96) |
| Stroke | SHBG | rs35983031 | 0.87(0.79,0.96) |
| Stroke | SHBG | rs36086195 | 0.87(0.79,0.95) |
| Stroke | SHBG | rs36124182 | 0.87(0.79,0.95) |
| Stroke | SHBG | rs362413 | 0.87(0.79,0.96) |
| Stroke | SHBG | rs368214 | 0.87(0.79,0.96) |
| Stroke | SHBG | rs3733892 | 0.87(0.79,0.96) |
| Stroke | SHBG | rs3737178 | 0.87(0.79,0.96) |
| Stroke | SHBG | rs3741368 | 0.87(0.79,0.96) |
| Stroke | SHBG | rs3742366 | 0.87(0.79,0.96) |
| Stroke | SHBG | rs3743588 | 0.87(0.79,0.96) |
| Stroke | SHBG | rs3761706 | 0.87(0.79,0.96) |
| Stroke | SHBG | rs3764002 | 0.87(0.79,0.96) |
| Stroke | SHBG | rs3770781 | 0.87(0.79,0.96) |
| Stroke | SHBG | rs3780190 | 0.87(0.79,0.95) |
| Stroke | SHBG | rs3782735 | 0.87(0.79,0.96) |
| Stroke | SHBG | rs3813498 | 0.87(0.79,0.95) |
| Stroke | SHBG | rs3829639 | 0.87(0.79,0.96) |
| Stroke | SHBG | rs38304 | 0.87(0.79,0.96) |
| Stroke | SHBG | rs3848119 | 0.87(0.79,0.95) |
| Stroke | SHBG | rs3848125 | 0.87(0.79,0.95) |
| Stroke | SHBG | rs38855 | 0.87(0.79,0.96) |
| Stroke | SHBG | rs40270 | 0.87(0.79,0.96) |
| Stroke | SHBG | rs403694 | 0.87(0.79,0.96) |
| Stroke | SHBG | rs4073358 | 0.87(0.79,0.96) |
| Stroke | SHBG | rs4092465 | 0.87(0.79,0.96) |
| Stroke | SHBG | rs41280463 | 0.87(0.79,0.96) |
| Stroke | SHBG | rs4147563 | 0.87(0.79,0.96) |
| Stroke | SHBG | rs4147913 | 0.87(0.79,0.96) |
| Stroke | SHBG | rs42238 | 0.87(0.79,0.96) |
| Stroke | SHBG | rs4274814 | 0.87(0.79,0.96) |
| Stroke | SHBG | rs4297769 | 0.87(0.80,0.96) |
| Stroke | SHBG | rs4300303 | 0.87(0.79,0.96) |
| Stroke | SHBG | rs4309185 | 0.87(0.79,0.96) |
| Stroke | SHBG | rs4338849 | 0.87(0.79,0.96) |
| Stroke | SHBG | rs434325 | 0.87(0.79,0.96) |
| Stroke | SHBG | rs4381470 | 0.87(0.79,0.96) |
| Stroke | SHBG | rs445 | 0.87(0.79,0.96) |
| Stroke | SHBG | rs45512696 | 0.87(0.79,0.95) |
| Stroke | SHBG | rs4568281 | 0.87(0.79,0.96) |
| Stroke | SHBG | rs4599176 | 0.87(0.79,0.95) |
| Stroke | SHBG | rs4639796 | 0.87(0.79,0.95) |
| Stroke | SHBG | rs4665710 | 0.87(0.79,0.96) |
| Stroke | SHBG | rs4665972 | 0.87(0.79,0.96) |
| Stroke | SHBG | rs4671328 | 0.87(0.79,0.96) |
| Stroke | SHBG | rs4674669 | 0.87(0.79,0.96) |
| Stroke | SHBG | rs4680 | 0.87(0.79,0.96) |
| Stroke | SHBG | rs4745876 | 0.87(0.79,0.96) |
| Stroke | SHBG | rs4762962 | 0.87(0.79,0.96) |
| Stroke | SHBG | rs4794008 | 0.87(0.79,0.96) |
| Stroke | SHBG | rs4804414 | 0.87(0.79,0.95) |
| Stroke | SHBG | rs4811050 | 0.87(0.79,0.96) |
| Stroke | SHBG | rs4812336 | 0.87(0.79,0.96) |
| Stroke | SHBG | rs4820091 | 0.87(0.79,0.95) |
| Stroke | SHBG | rs483082 | 0.87(0.79,0.96) |
| Stroke | SHBG | rs4837794 | 0.87(0.79,0.95) |
| Stroke | SHBG | rs4841133 | 0.87(0.79,0.96) |
| Stroke | SHBG | rs484943 | 0.87(0.79,0.96) |
| Stroke | SHBG | rs4970837 | 0.87(0.79,0.96) |
| Stroke | SHBG | rs4974310 | 0.87(0.79,0.96) |
| Stroke | SHBG | rs4976033 | 0.87(0.79,0.96) |
| Stroke | SHBG | rs5017726 | 0.87(0.79,0.96) |
| Stroke | SHBG | rs55737395 | 0.87(0.79,0.96) |
| Stroke | SHBG | rs55761545 | 0.87(0.79,0.95) |
| Stroke | SHBG | rs55771168 | 0.87(0.79,0.95) |
| Stroke | SHBG | rs55831924 | 0.87(0.79,0.96) |
| Stroke | SHBG | rs55910553 | 0.87(0.79,0.96) |
| Stroke | SHBG | rs55974289 | 0.87(0.79,0.95) |
| Stroke | SHBG | rs55987409 | 0.87(0.79,0.95) |
| Stroke | SHBG | rs559986 | 0.87(0.79,0.96) |
| Stroke | SHBG | rs56021343 | 0.87(0.79,0.96) |
| Stroke | SHBG | rs56196860 | 0.87(0.79,0.96) |
| Stroke | SHBG | rs56292801 | 0.87(0.79,0.96) |
| Stroke | SHBG | rs56332871 | 0.86(0.78,0.95) |
| Stroke | SHBG | rs56365029 | 0.87(0.79,0.96) |
| Stroke | SHBG | rs57158761 | 0.87(0.79,0.96) |
| Stroke | SHBG | rs57467915 | 0.87(0.79,0.96) |
| Stroke | SHBG | rs575452 | 0.87(0.79,0.96) |
| Stroke | SHBG | rs5760120 | 0.87(0.79,0.96) |
| Stroke | SHBG | rs57754494 | 0.87(0.79,0.96) |
| Stroke | SHBG | rs58321169 | 0.87(0.79,0.96) |
| Stroke | SHBG | rs58429317 | 0.87(0.79,0.96) |
| Stroke | SHBG | rs591939 | 0.87(0.79,0.96) |
| Stroke | SHBG | rs59662471 | 0.87(0.79,0.96) |
| Stroke | SHBG | rs59708846 | 0.87(0.79,0.96) |
| Stroke | SHBG | rs59708898 | 0.87(0.79,0.96) |
| Stroke | SHBG | rs60018147 | 0.87(0.79,0.96) |
| Stroke | SHBG | rs6018424 | 0.87(0.79,0.96) |
| Stroke | SHBG | rs6048205 | 0.87(0.79,0.96) |
| Stroke | SHBG | rs6062381 | 0.87(0.79,0.96) |
| Stroke | SHBG | rs6073431 | 0.87(0.79,0.96) |
| Stroke | SHBG | rs608300 | 0.87(0.79,0.95) |
| Stroke | SHBG | rs6118 | 0.87(0.79,0.96) |
| Stroke | SHBG | rs6120663 | 0.87(0.79,0.96) |
| Stroke | SHBG | rs61292904 | 0.87(0.79,0.96) |
| Stroke | SHBG | rs6129802 | 0.87(0.79,0.96) |
| Stroke | SHBG | rs61599759 | 0.87(0.79,0.96) |
| Stroke | SHBG | rs61733486 | 0.87(0.79,0.96) |
| Stroke | SHBG | rs61759532 | 0.87(0.79,0.96) |
| Stroke | SHBG | rs61779331 | 0.87(0.79,0.96) |
| Stroke | SHBG | rs61853560 | 0.87(0.79,0.96) |
| Stroke | SHBG | rs61854630 | 0.87(0.79,0.96) |
| Stroke | SHBG | rs61856602 | 0.87(0.79,0.96) |
| Stroke | SHBG | rs619526 | 0.87(0.79,0.96) |
| Stroke | SHBG | rs62012946 | 0.87(0.79,0.96) |
| Stroke | SHBG | rs62037803 | 0.87(0.79,0.96) |
| Stroke | SHBG | rs62111692 | 0.87(0.79,0.96) |
| Stroke | SHBG | rs62182125 | 0.87(0.79,0.95) |
| Stroke | SHBG | rs62195072 | 0.87(0.79,0.96) |
| Stroke | SHBG | rs62334584 | 0.87(0.79,0.96) |
| Stroke | SHBG | rs62396733 | 0.87(0.79,0.95) |
| Stroke | SHBG | rs62515079 | 0.87(0.79,0.95) |
| Stroke | SHBG | rs62565259 | 0.87(0.79,0.96) |
| Stroke | SHBG | rs62580766 | 0.87(0.79,0.95) |
| Stroke | SHBG | rs62618693 | 0.87(0.79,0.96) |
| Stroke | SHBG | rs6422513 | 0.87(0.79,0.96) |
| Stroke | SHBG | rs6476065 | 0.87(0.79,0.96) |
| Stroke | SHBG | rs6486122 | 0.87(0.79,0.96) |
| Stroke | SHBG | rs6495962 | 0.87(0.79,0.96) |
| Stroke | SHBG | rs6546096 | 0.87(0.79,0.96) |
| Stroke | SHBG | rs6556402 | 0.87(0.79,0.96) |
| Stroke | SHBG | rs6567160 | 0.87(0.79,0.96) |
| Stroke | SHBG | rs6575439 | 0.87(0.79,0.96) |
| Stroke | SHBG | rs662026 | 0.87(0.79,0.96) |
| Stroke | SHBG | rs6684464 | 0.87(0.79,0.96) |
| Stroke | SHBG | rs668871 | 0.87(0.79,0.95) |
| Stroke | SHBG | rs66921136 | 0.87(0.79,0.96) |
| Stroke | SHBG | rs6710171 | 0.87(0.79,0.96) |
| Stroke | SHBG | rs6736913 | 0.87(0.79,0.95) |
| Stroke | SHBG | rs6741180 | 0.87(0.79,0.96) |
| Stroke | SHBG | rs6755571 | 0.87(0.79,0.96) |
| Stroke | SHBG | rs6791074 | 0.87(0.79,0.96) |
| Stroke | SHBG | rs6792725 | 0.87(0.79,0.96) |
| Stroke | SHBG | rs6804915 | 0.87(0.79,0.96) |
| Stroke | SHBG | rs68062403 | 0.87(0.79,0.96) |
| Stroke | SHBG | rs687339 | 0.87(0.79,0.96) |
| Stroke | SHBG | rs6910879 | 0.87(0.79,0.96) |
| Stroke | SHBG | rs6939861 | 0.87(0.79,0.96) |
| Stroke | SHBG | rs695272 | 0.87(0.80,0.96) |
| Stroke | SHBG | rs7000496 | 0.87(0.79,0.96) |
| Stroke | SHBG | rs700085 | 0.87(0.79,0.96) |
| Stroke | SHBG | rs7015 | 0.88(0.80,0.97) |
| Stroke | SHBG | rs702876 | 0.87(0.79,0.96) |
| Stroke | SHBG | rs7117818 | 0.87(0.79,0.96) |
| Stroke | SHBG | rs7123361 | 0.87(0.79,0.95) |
| Stroke | SHBG | rs7131509 | 0.87(0.79,0.96) |
| Stroke | SHBG | rs715 | 0.87(0.79,0.96) |
| Stroke | SHBG | rs7157184 | 0.87(0.79,0.96) |
| Stroke | SHBG | rs7164175 | 0.87(0.79,0.96) |
| Stroke | SHBG | rs7183456 | 0.87(0.79,0.96) |
| Stroke | SHBG | rs720130 | 0.87(0.79,0.96) |
| Stroke | SHBG | rs7221716 | 0.87(0.79,0.96) |
| Stroke | SHBG | rs7250869 | 0.87(0.79,0.96) |
| Stroke | SHBG | rs7262150 | 0.87(0.79,0.96) |
| Stroke | SHBG | rs72663937 | 0.87(0.79,0.96) |
| Stroke | SHBG | rs72666817 | 0.87(0.79,0.96) |
| Stroke | SHBG | rs72683923 | 0.87(0.79,0.96) |
| Stroke | SHBG | rs72694845 | 0.87(0.79,0.96) |
| Stroke | SHBG | rs72709458 | 0.87(0.79,0.96) |
| Stroke | SHBG | rs72753349 | 0.87(0.79,0.96) |
| Stroke | SHBG | rs72753908 | 0.87(0.79,0.96) |
| Stroke | SHBG | rs72766607 | 0.87(0.79,0.96) |
| Stroke | SHBG | rs72767773 | 0.87(0.79,0.95) |
| Stroke | SHBG | rs72810505 | 0.87(0.79,0.96) |
| Stroke | SHBG | rs72815155 | 0.87(0.79,0.96) |
| Stroke | SHBG | rs72840987 | 0.87(0.79,0.96) |
| Stroke | SHBG | rs72842808 | 0.87(0.79,0.96) |
| Stroke | SHBG | rs72844546 | 0.87(0.79,0.95) |
| Stroke | SHBG | rs7298924 | 0.88(0.80,0.96) |
| Stroke | SHBG | rs7301634 | 0.87(0.79,0.96) |
| Stroke | SHBG | rs73047887 | 0.87(0.79,0.96) |
| Stroke | SHBG | rs7308634 | 0.87(0.79,0.96) |
| Stroke | SHBG | rs7314285 | 0.88(0.80,0.96) |
| Stroke | SHBG | rs73193388 | 0.87(0.79,0.96) |
| Stroke | SHBG | rs73223295 | 0.87(0.79,0.96) |
| Stroke | SHBG | rs7323372 | 0.87(0.79,0.96) |
| Stroke | SHBG | rs73375029 | 0.87(0.79,0.96) |
| Stroke | SHBG | rs73597479 | 0.87(0.79,0.96) |
| Stroke | SHBG | rs736820 | 0.87(0.79,0.96) |
| Stroke | SHBG | rs73705826 | 0.87(0.79,0.96) |
| Stroke | SHBG | rs73972648 | 0.87(0.79,0.96) |
| Stroke | SHBG | rs7406661 | 0.87(0.79,0.96) |
| Stroke | SHBG | rs744200 | 0.87(0.79,0.96) |
| Stroke | SHBG | rs7451021 | 0.87(0.79,0.96) |
| Stroke | SHBG | rs74551598 | 0.87(0.79,0.96) |
| Stroke | SHBG | rs74998771 | 0.87(0.79,0.96) |
| Stroke | SHBG | rs750155 | 0.87(0.79,0.96) |
| Stroke | SHBG | rs75077113 | 0.87(0.79,0.96) |
| Stroke | SHBG | rs75156222 | 0.87(0.79,0.96) |
| Stroke | SHBG | rs7535528 | 0.87(0.79,0.96) |
| Stroke | SHBG | rs7540115 | 0.87(0.79,0.96) |
| Stroke | SHBG | rs7552207 | 0.87(0.79,0.96) |
| Stroke | SHBG | rs75713100 | 0.87(0.79,0.96) |
| Stroke | SHBG | rs757869 | 0.87(0.79,0.96) |
| Stroke | SHBG | rs759404 | 0.87(0.79,0.96) |
| Stroke | SHBG | rs7617967 | 0.87(0.79,0.96) |
| Stroke | SHBG | rs76345703 | 0.87(0.79,0.96) |
| Stroke | SHBG | rs7638782 | 0.87(0.79,0.96) |
| Stroke | SHBG | rs76475417 | 0.87(0.79,0.96) |
| Stroke | SHBG | rs76549335 | 0.87(0.79,0.96) |
| Stroke | SHBG | rs7668413 | 0.87(0.79,0.96) |
| Stroke | SHBG | rs76708468 | 0.87(0.79,0.96) |
| Stroke | SHBG | rs7678138 | 0.87(0.79,0.96) |
| Stroke | SHBG | rs76895963 | 0.87(0.79,0.96) |
| Stroke | SHBG | rs7696472 | 0.87(0.79,0.95) |
| Stroke | SHBG | rs7756992 | 0.87(0.79,0.96) |
| Stroke | SHBG | rs77597993 | 0.87(0.79,0.96) |
| Stroke | SHBG | rs78058190 | 0.87(0.79,0.96) |
| Stroke | SHBG | rs7809920 | 0.87(0.79,0.96) |
| Stroke | SHBG | rs7828742 | 0.87(0.79,0.95) |
| Stroke | SHBG | rs78319058 | 0.87(0.79,0.96) |
| Stroke | SHBG | rs78444298 | 0.87(0.79,0.96) |
| Stroke | SHBG | rs78496430 | 0.87(0.79,0.96) |
| Stroke | SHBG | rs78536975 | 0.87(0.79,0.96) |
| Stroke | SHBG | rs78555071 | 0.87(0.79,0.96) |
| Stroke | SHBG | rs7863263 | 0.87(0.79,0.96) |
| Stroke | SHBG | rs787976 | 0.87(0.79,0.96) |
| Stroke | SHBG | rs78890745 | 0.87(0.79,0.96) |
| Stroke | SHBG | rs78973091 | 0.87(0.79,0.96) |
| Stroke | SHBG | rs7899096 | 0.87(0.79,0.96) |
| Stroke | SHBG | rs7918533 | 0.87(0.79,0.96) |
| Stroke | SHBG | rs79287178 | 0.87(0.79,0.96) |
| Stroke | SHBG | rs79354983 | 0.87(0.79,0.96) |
| Stroke | SHBG | rs79391862 | 0.87(0.79,0.96) |
| Stroke | SHBG | rs7944853 | 0.87(0.79,0.96) |
| Stroke | SHBG | rs7953508 | 0.87(0.79,0.96) |
| Stroke | SHBG | rs796004 | 0.87(0.79,0.95) |
| Stroke | SHBG | rs79600740 | 0.87(0.79,0.96) |
| Stroke | SHBG | rs79683734 | 0.87(0.79,0.96) |
| Stroke | SHBG | rs79717793 | 0.87(0.80,0.96) |
| Stroke | SHBG | rs79875164 | 0.87(0.79,0.96) |
| Stroke | SHBG | rs8001781 | 0.87(0.79,0.96) |
| Stroke | SHBG | rs80226362 | 0.87(0.79,0.96) |
| Stroke | SHBG | rs8031716 | 0.87(0.79,0.96) |
| Stroke | SHBG | rs8043101 | 0.87(0.79,0.95) |
| Stroke | SHBG | rs8066941 | 0.87(0.79,0.96) |
| Stroke | SHBG | rs8069105 | 0.87(0.79,0.96) |
| Stroke | SHBG | rs8074363 | 0.87(0.79,0.96) |
| Stroke | SHBG | rs8077323 | 0.87(0.79,0.96) |
| Stroke | SHBG | rs8079418 | 0.88(0.80,0.96) |
| Stroke | SHBG | rs8107967 | 0.87(0.79,0.96) |
| Stroke | SHBG | rs8134638 | 0.87(0.79,0.96) |
| Stroke | SHBG | rs8176693 | 0.87(0.79,0.95) |
| Stroke | SHBG | rs820503 | 0.87(0.79,0.96) |
| Stroke | SHBG | rs822508 | 0.87(0.79,0.96) |
| Stroke | SHBG | rs841194 | 0.87(0.79,0.96) |
| Stroke | SHBG | rs857152 | 0.87(0.79,0.96) |
| Stroke | SHBG | rs864899 | 0.87(0.79,0.96) |
| Stroke | SHBG | rs885683 | 0.87(0.79,0.96) |
| Stroke | SHBG | rs901886 | 0.87(0.79,0.96) |
| Stroke | SHBG | rs9297994 | 0.87(0.80,0.96) |
| Stroke | SHBG | rs9379084 | 0.87(0.79,0.95) |
| Stroke | SHBG | rs9427104 | 0.87(0.79,0.96) |
| Stroke | SHBG | rs9439469 | 0.87(0.79,0.96) |
| Stroke | SHBG | rs9465601 | 0.87(0.79,0.96) |
| Stroke | SHBG | rs9492 | 0.87(0.79,0.96) |
| Stroke | SHBG | rs9495298 | 0.87(0.79,0.96) |
| Stroke | SHBG | rs9533843 | 0.87(0.79,0.96) |
| Stroke | SHBG | rs9556403 | 0.87(0.79,0.96) |
| Stroke | SHBG | rs9597811 | 0.87(0.79,0.96) |
| Stroke | SHBG | rs9610329 | 0.87(0.79,0.96) |
| Stroke | SHBG | rs9614162 | 0.87(0.79,0.96) |
| Stroke | SHBG | rs9686661 | 0.87(0.79,0.96) |
| Stroke | SHBG | rs9697210 | 0.87(0.79,0.96) |
| Stroke | SHBG | rs9823108 | 0.87(0.79,0.96) |
| Stroke | SHBG | rs9823118 | 0.87(0.79,0.95) |
| Stroke | SHBG | rs9831794 | 0.87(0.79,0.96) |
| Stroke | SHBG | rs9871160 | 0.87(0.79,0.96) |
| Stroke | SHBG | rs9893194 | 0.87(0.79,0.96) |
| Stroke | SHBG | rs9902384 | 0.87(0.79,0.96) |
| Stroke | SHBG | rs9968070 | 0.87(0.79,0.96) |
| Stroke | SHBG | rs9972653 | 0.87(0.79,0.96) |
| Stroke | SHBG | rs998584 | 0.87(0.79,0.96) |
| Stroke | SHBG | rs999634 | 0.87(0.79,0.96) |
| IS | SHBG | rs10026753 | 0.86(0.77,0.95) |
| IS | SHBG | rs10041660 | 0.86(0.77,0.95) |
| IS | SHBG | rs1005421 | 0.86(0.77,0.95) |
| IS | SHBG | rs1007851 | 0.86(0.78,0.95) |
| IS | SHBG | rs10084025 | 0.86(0.77,0.95) |
| IS | SHBG | rs10095103 | 0.86(0.77,0.95) |
| IS | SHBG | rs10110651 | 0.86(0.77,0.95) |
| IS | SHBG | rs10111451 | 0.86(0.77,0.95) |
| IS | SHBG | rs10123811 | 0.86(0.77,0.95) |
| IS | SHBG | rs10125995 | 0.86(0.77,0.95) |
| IS | SHBG | rs10153315 | 0.86(0.77,0.95) |
| IS | SHBG | rs10163091 | 0.86(0.78,0.95) |
| IS | SHBG | rs10187560 | 0.86(0.77,0.95) |
| IS | SHBG | rs10208512 | 0.86(0.78,0.95) |
| IS | SHBG | rs10238028 | 0.86(0.77,0.95) |
| IS | SHBG | rs10258433 | 0.86(0.77,0.95) |
| IS | SHBG | rs10273476 | 0.86(0.78,0.95) |
| IS | SHBG | rs10411932 | 0.86(0.78,0.95) |
| IS | SHBG | rs10413329 | 0.86(0.78,0.95) |
| IS | SHBG | rs10416080 | 0.86(0.77,0.95) |
| IS | SHBG | rs1042725 | 0.86(0.78,0.95) |
| IS | SHBG | rs10432029 | 0.86(0.78,0.96) |
| IS | SHBG | rs10492118 | 0.86(0.77,0.95) |
| IS | SHBG | rs10504731 | 0.86(0.78,0.95) |
| IS | SHBG | rs1059698 | 0.86(0.77,0.95) |
| IS | SHBG | rs10733608 | 0.86(0.78,0.95) |
| IS | SHBG | rs10761676 | 0.86(0.77,0.95) |
| IS | SHBG | rs10761749 | 0.86(0.78,0.96) |
| IS | SHBG | rs10773049 | 0.86(0.78,0.95) |
| IS | SHBG | rs10794307 | 0.86(0.78,0.95) |
| IS | SHBG | rs10815276 | 0.86(0.78,0.95) |
| IS | SHBG | rs10822130 | 0.86(0.77,0.95) |
| IS | SHBG | rs10824742 | 0.86(0.77,0.95) |
| IS | SHBG | rs10838681 | 0.86(0.77,0.95) |
| IS | SHBG | rs10864070 | 0.86(0.78,0.95) |
| IS | SHBG | rs10880872 | 0.86(0.77,0.95) |
| IS | SHBG | rs10888696 | 0.86(0.78,0.95) |
| IS | SHBG | rs10895276 | 0.86(0.77,0.95) |
| IS | SHBG | rs10900446 | 0.86(0.77,0.95) |
| IS | SHBG | rs10946313 | 0.86(0.77,0.95) |
| IS | SHBG | rs10951130 | 0.86(0.77,0.95) |
| IS | SHBG | rs10964337 | 0.86(0.78,0.95) |
| IS | SHBG | rs11029441 | 0.86(0.78,0.95) |
| IS | SHBG | rs11030100 | 0.86(0.78,0.95) |
| IS | SHBG | rs11032076 | 0.86(0.77,0.95) |
| IS | SHBG | rs11038673 | 0.86(0.78,0.95) |
| IS | SHBG | rs11078597 | 0.86(0.77,0.95) |
| IS | SHBG | rs11078681 | 0.86(0.77,0.95) |
| IS | SHBG | rs11078701 | 0.86(0.78,0.96) |
| IS | SHBG | rs11079872 | 0.86(0.78,0.95) |
| IS | SHBG | rs11108061 | 0.86(0.78,0.95) |
| IS | SHBG | rs11110390 | 0.86(0.77,0.95) |
| IS | SHBG | rs11111274 | 0.86(0.77,0.95) |
| IS | SHBG | rs11121522 | 0.86(0.77,0.95) |
| IS | SHBG | rs111289824 | 0.86(0.77,0.95) |
| IS | SHBG | rs111331455 | 0.86(0.78,0.95) |
| IS | SHBG | rs111363146 | 0.86(0.78,0.95) |
| IS | SHBG | rs11153046 | 0.86(0.77,0.95) |
| IS | SHBG | rs11155787 | 0.86(0.77,0.95) |
| IS | SHBG | rs111604078 | 0.86(0.77,0.95) |
| IS | SHBG | rs111637026 | 0.86(0.77,0.95) |
| IS | SHBG | rs11164095 | 0.86(0.77,0.95) |
| IS | SHBG | rs11165493 | 0.86(0.77,0.95) |
| IS | SHBG | rs111700120 | 0.86(0.77,0.95) |
| IS | SHBG | rs11187142 | 0.86(0.77,0.95) |
| IS | SHBG | rs11188604 | 0.86(0.77,0.95) |
| IS | SHBG | rs11190245 | 0.86(0.77,0.95) |
| IS | SHBG | rs111905890 | 0.86(0.77,0.95) |
| IS | SHBG | rs11191841 | 0.86(0.77,0.95) |
| IS | SHBG | rs111981233 | 0.86(0.78,0.95) |
| IS | SHBG | rs11202594 | 0.86(0.77,0.95) |
| IS | SHBG | rs112035922 | 0.86(0.78,0.95) |
| IS | SHBG | rs112575738 | 0.86(0.78,0.95) |
| IS | SHBG | rs112672290 | 0.86(0.77,0.95) |
| IS | SHBG | rs112833123 | 0.86(0.77,0.95) |
| IS | SHBG | rs112850234 | 0.86(0.77,0.95) |
| IS | SHBG | rs112928223 | 0.86(0.77,0.95) |
| IS | SHBG | rs113251204 | 0.86(0.78,0.95) |
| IS | SHBG | rs113364399 | 0.86(0.77,0.95) |
| IS | SHBG | rs113523273 | 0.86(0.78,0.95) |
| IS | SHBG | rs113973451 | 0.86(0.77,0.95) |
| IS | SHBG | rs114053844 | 0.86(0.78,0.95) |
| IS | SHBG | rs114469183 | 0.86(0.77,0.95) |
| IS | SHBG | rs114627598 | 0.86(0.77,0.95) |
| IS | SHBG | rs114940462 | 0.86(0.77,0.95) |
| IS | SHBG | rs114949263 | 0.86(0.77,0.95) |
| IS | SHBG | rs11539938 | 0.86(0.77,0.95) |
| IS | SHBG | rs11542663 | 0.86(0.77,0.95) |
| IS | SHBG | rs11545185 | 0.86(0.78,0.95) |
| IS | SHBG | rs115521489 | 0.86(0.78,0.95) |
| IS | SHBG | rs11552708 | 0.85(0.76,0.94) |
| IS | SHBG | rs11556924 | 0.86(0.77,0.95) |
| IS | SHBG | rs11564722 | 0.86(0.77,0.95) |
| IS | SHBG | rs11601507 | 0.86(0.77,0.95) |
| IS | SHBG | rs11621792 | 0.86(0.77,0.95) |
| IS | SHBG | rs11633147 | 0.86(0.77,0.95) |
| IS | SHBG | rs116573491 | 0.86(0.77,0.95) |
| IS | SHBG | rs116713089 | 0.86(0.77,0.95) |
| IS | SHBG | rs11682084 | 0.86(0.77,0.95) |
| IS | SHBG | rs11690176 | 0.86(0.77,0.95) |
| IS | SHBG | rs117108573 | 0.86(0.77,0.95) |
| IS | SHBG | rs117135073 | 0.86(0.77,0.95) |
| IS | SHBG | rs117169274 | 0.86(0.78,0.95) |
| IS | SHBG | rs117411982 | 0.86(0.77,0.95) |
| IS | SHBG | rs11743810 | 0.86(0.77,0.95) |
| IS | SHBG | rs11748938 | 0.86(0.78,0.95) |
| IS | SHBG | rs117589665 | 0.86(0.78,0.95) |
| IS | SHBG | rs11765639 | 0.86(0.77,0.95) |
| IS | SHBG | rs11791747 | 0.86(0.77,0.95) |
| IS | SHBG | rs118080406 | 0.86(0.77,0.95) |
| IS | SHBG | rs11856606 | 0.86(0.77,0.95) |
| IS | SHBG | rs11856926 | 0.86(0.77,0.95) |
| IS | SHBG | rs11994858 | 0.86(0.77,0.95) |
| IS | SHBG | rs11997548 | 0.86(0.77,0.95) |
| IS | SHBG | rs12059956 | 0.86(0.78,0.95) |
| IS | SHBG | rs12138461 | 0.86(0.77,0.95) |
| IS | SHBG | rs12185242 | 0.86(0.78,0.96) |
| IS | SHBG | rs12263369 | 0.86(0.78,0.96) |
| IS | SHBG | rs12280075 | 0.86(0.77,0.95) |
| IS | SHBG | rs1229498 | 0.86(0.77,0.95) |
| IS | SHBG | rs12302952 | 0.86(0.77,0.95) |
| IS | SHBG | rs12311848 | 0.86(0.78,0.95) |
| IS | SHBG | rs12373799 | 0.86(0.77,0.95) |
| IS | SHBG | rs12413488 | 0.86(0.77,0.95) |
| IS | SHBG | rs12424336 | 0.86(0.77,0.95) |
| IS | SHBG | rs12454712 | 0.86(0.77,0.95) |
| IS | SHBG | rs12476661 | 0.86(0.78,0.95) |
| IS | SHBG | rs12575636 | 0.86(0.77,0.95) |
| IS | SHBG | rs12601778 | 0.86(0.77,0.95) |
| IS | SHBG | rs12636106 | 0.86(0.77,0.95) |
| IS | SHBG | rs12667888 | 0.86(0.77,0.95) |
| IS | SHBG | rs12758998 | 0.86(0.77,0.95) |
| IS | SHBG | rs12797706 | 0.86(0.78,0.96) |
| IS | SHBG | rs12809946 | 0.86(0.77,0.95) |
| IS | SHBG | rs12818938 | 0.86(0.77,0.95) |
| IS | SHBG | rs12879423 | 0.86(0.77,0.95) |
| IS | SHBG | rs12916 | 0.86(0.78,0.95) |
| IS | SHBG | rs12926107 | 0.86(0.78,0.96) |
| IS | SHBG | rs12928099 | 0.86(0.77,0.95) |
| IS | SHBG | rs12965052 | 0.86(0.77,0.95) |
| IS | SHBG | rs12989083 | 0.86(0.77,0.95) |
| IS | SHBG | rs13000027 | 0.86(0.78,0.95) |
| IS | SHBG | rs13035806 | 0.86(0.78,0.95) |
| IS | SHBG | rs13057133 | 0.86(0.77,0.95) |
| IS | SHBG | rs13086465 | 0.86(0.78,0.96) |
| IS | SHBG | rs13094241 | 0.86(0.78,0.95) |
| IS | SHBG | rs13108218 | 0.85(0.77,0.94) |
| IS | SHBG | rs13149606 | 0.86(0.77,0.95) |
| IS | SHBG | rs13150068 | 0.87(0.78,0.96) |
| IS | SHBG | rs13251458 | 0.86(0.77,0.95) |
| IS | SHBG | rs1330307 | 0.86(0.78,0.95) |
| IS | SHBG | rs13315174 | 0.86(0.77,0.95) |
| IS | SHBG | rs13379043 | 0.86(0.77,0.95) |
| IS | SHBG | rs13389219 | 0.86(0.78,0.95) |
| IS | SHBG | rs13405815 | 0.86(0.77,0.95) |
| IS | SHBG | rs1352084 | 0.86(0.78,0.95) |
| IS | SHBG | rs138526953 | 0.86(0.77,0.95) |
| IS | SHBG | rs138755456 | 0.86(0.77,0.95) |
| IS | SHBG | rs139805419 | 0.86(0.78,0.95) |
| IS | SHBG | rs139974673 | 0.87(0.78,0.96) |
| IS | SHBG | rs140105410 | 0.86(0.77,0.95) |
| IS | SHBG | rs1408270 | 0.86(0.77,0.95) |
| IS | SHBG | rs1411432 | 0.86(0.77,0.95) |
| IS | SHBG | rs14129 | 0.86(0.77,0.95) |
| IS | SHBG | rs141899843 | 0.86(0.77,0.95) |
| IS | SHBG | rs1420385 | 0.86(0.77,0.95) |
| IS | SHBG | rs142331290 | 0.86(0.77,0.95) |
| IS | SHBG | rs1431659 | 0.86(0.77,0.95) |
| IS | SHBG | rs1433210 | 0.86(0.77,0.95) |
| IS | SHBG | rs143709973 | 0.86(0.77,0.95) |
| IS | SHBG | rs144459202 | 0.86(0.78,0.95) |
| IS | SHBG | rs144989856 | 0.86(0.77,0.95) |
| IS | SHBG | rs145931818 | 0.86(0.78,0.95) |
| IS | SHBG | rs147259681 | 0.86(0.77,0.95) |
| IS | SHBG | rs148118632 | 0.86(0.77,0.95) |
| IS | SHBG | rs149092986 | 0.86(0.77,0.95) |
| IS | SHBG | rs149102638 | 0.86(0.77,0.95) |
| IS | SHBG | rs150895955 | 0.86(0.78,0.95) |
| IS | SHBG | rs1530439 | 0.86(0.78,0.95) |
| IS | SHBG | rs1534696 | 0.86(0.77,0.95) |
| IS | SHBG | rs1561442 | 0.86(0.78,0.95) |
| IS | SHBG | rs1570360 | 0.86(0.77,0.95) |
| IS | SHBG | rs157935 | 0.86(0.78,0.95) |
| IS | SHBG | rs1640269 | 0.86(0.77,0.95) |
| IS | SHBG | rs16835135 | 0.86(0.77,0.95) |
| IS | SHBG | rs16934748 | 0.86(0.78,0.95) |
| IS | SHBG | rs1708302 | 0.86(0.78,0.95) |
| IS | SHBG | rs17184382 | 0.86(0.78,0.95) |
| IS | SHBG | rs17207107 | 0.86(0.77,0.95) |
| IS | SHBG | rs1730862 | 0.86(0.77,0.95) |
| IS | SHBG | rs17356664 | 0.86(0.77,0.95) |
| IS | SHBG | rs17372936 | 0.86(0.77,0.95) |
| IS | SHBG | rs1738380 | 0.86(0.77,0.95) |
| IS | SHBG | rs1743954 | 0.86(0.78,0.95) |
| IS | SHBG | rs174554 | 0.85(0.77,0.95) |
| IS | SHBG | rs17583875 | 0.86(0.78,0.95) |
| IS | SHBG | rs17592998 | 0.86(0.77,0.95) |
| IS | SHBG | rs17669311 | 0.86(0.77,0.95) |
| IS | SHBG | rs1775125 | 0.86(0.78,0.95) |
| IS | SHBG | rs17751614 | 0.86(0.77,0.95) |
| IS | SHBG | rs17755271 | 0.86(0.78,0.95) |
| IS | SHBG | rs17881850 | 0.86(0.78,0.95) |
| IS | SHBG | rs1799831 | 0.86(0.77,0.95) |
| IS | SHBG | rs1801689 | 0.86(0.78,0.96) |
| IS | SHBG | rs182132993 | 0.86(0.77,0.95) |
| IS | SHBG | rs1823227 | 0.86(0.77,0.95) |
| IS | SHBG | rs182848434 | 0.86(0.77,0.95) |
| IS | SHBG | rs183015141 | 0.86(0.77,0.95) |
| IS | SHBG | rs184304 | 0.86(0.78,0.95) |
| IS | SHBG | rs185406435 | 0.86(0.77,0.95) |
| IS | SHBG | rs1864390 | 0.85(0.77,0.95) |
| IS | SHBG | rs186766320 | 0.86(0.77,0.95) |
| IS | SHBG | rs1883783 | 0.86(0.77,0.95) |
| IS | SHBG | rs188889872 | 0.86(0.78,0.95) |
| IS | SHBG | rs188949713 | 0.86(0.77,0.95) |
| IS | SHBG | rs189595752 | 0.86(0.77,0.95) |
| IS | SHBG | rs190712219 | 0.86(0.77,0.95) |
| IS | SHBG | rs1951244 | 0.86(0.77,0.95) |
| IS | SHBG | rs1969213 | 0.86(0.77,0.95) |
| IS | SHBG | rs1993669 | 0.86(0.77,0.95) |
| IS | SHBG | rs201570119 | 0.86(0.77,0.95) |
| IS | SHBG | rs2022865 | 0.86(0.77,0.95) |
| IS | SHBG | rs203777 | 0.86(0.77,0.95) |
| IS | SHBG | rs2063245 | 0.86(0.77,0.95) |
| IS | SHBG | rs2074683 | 0.86(0.77,0.95) |
| IS | SHBG | rs2075915 | 0.86(0.77,0.95) |
| IS | SHBG | rs2121650 | 0.86(0.77,0.95) |
| IS | SHBG | rs2122982 | 0.86(0.78,0.95) |
| IS | SHBG | rs2156804 | 0.86(0.78,0.95) |
| IS | SHBG | rs2176887 | 0.86(0.77,0.95) |
| IS | SHBG | rs2196943 | 0.86(0.77,0.95) |
| IS | SHBG | rs2233364 | 0.86(0.78,0.95) |
| IS | SHBG | rs2234922 | 0.86(0.77,0.95) |
| IS | SHBG | rs2239222 | 0.86(0.78,0.95) |
| IS | SHBG | rs2241261 | 0.86(0.77,0.95) |
| IS | SHBG | rs2246223 | 0.86(0.78,0.95) |
| IS | SHBG | rs2273368 | 0.86(0.77,0.95) |
| IS | SHBG | rs2275355 | 0.86(0.77,0.95) |
| IS | SHBG | rs2277283 | 0.86(0.77,0.95) |
| IS | SHBG | rs2277641 | 0.86(0.77,0.95) |
| IS | SHBG | rs2280838 | 0.86(0.77,0.95) |
| IS | SHBG | rs2304686 | 0.86(0.77,0.95) |
| IS | SHBG | rs2305144 | 0.86(0.77,0.95) |
| IS | SHBG | rs234051 | 0.86(0.77,0.95) |
| IS | SHBG | rs2351958 | 0.86(0.78,0.95) |
| IS | SHBG | rs237438 | 0.86(0.78,0.95) |
| IS | SHBG | rs2393791 | 0.86(0.77,0.95) |
| IS | SHBG | rs2404976 | 0.86(0.77,0.95) |
| IS | SHBG | rs2431752 | 0.86(0.77,0.95) |
| IS | SHBG | rs2450128 | 0.86(0.77,0.95) |
| IS | SHBG | rs2522054 | 0.86(0.78,0.95) |
| IS | SHBG | rs2535404 | 0.86(0.77,0.95) |
| IS | SHBG | rs2551774 | 0.86(0.77,0.95) |
| IS | SHBG | rs2602856 | 0.86(0.78,0.95) |
| IS | SHBG | rs2618566 | 0.86(0.77,0.95) |
| IS | SHBG | rs2627690 | 0.86(0.78,0.95) |
| IS | SHBG | rs2642438 | 0.86(0.78,0.95) |
| IS | SHBG | rs267733 | 0.86(0.77,0.95) |
| IS | SHBG | rs2705619 | 0.86(0.77,0.95) |
| IS | SHBG | rs2723067 | 0.86(0.78,0.95) |
| IS | SHBG | rs2724475 | 0.86(0.78,0.96) |
| IS | SHBG | rs2746829 | 0.86(0.78,0.95) |
| IS | SHBG | rs2792022 | 0.86(0.77,0.95) |
| IS | SHBG | rs2807861 | 0.86(0.77,0.95) |
| IS | SHBG | rs28507491 | 0.86(0.78,0.96) |
| IS | SHBG | rs28562483 | 0.86(0.77,0.95) |
| IS | SHBG | rs2862954 | 0.86(0.78,0.95) |
| IS | SHBG | rs2885582 | 0.86(0.77,0.95) |
| IS | SHBG | rs28890929 | 0.86(0.77,0.95) |
| IS | SHBG | rs28925904 | 0.86(0.77,0.95) |
| IS | SHBG | rs28929474 | 0.85(0.77,0.95) |
| IS | SHBG | rs2914005 | 0.86(0.78,0.95) |
| IS | SHBG | rs2925979 | 0.86(0.78,0.95) |
| IS | SHBG | rs2942202 | 0.86(0.77,0.95) |
| IS | SHBG | rs2965196 | 0.86(0.77,0.95) |
| IS | SHBG | rs2970877 | 0.86(0.77,0.95) |
| IS | SHBG | rs2972145 | 0.86(0.77,0.95) |
| IS | SHBG | rs2972438 | 0.86(0.77,0.95) |
| IS | SHBG | rs3001032 | 0.86(0.77,0.95) |
| IS | SHBG | rs3004179 | 0.86(0.78,0.95) |
| IS | SHBG | rs3116625 | 0.86(0.77,0.95) |
| IS | SHBG | rs3217860 | 0.86(0.77,0.95) |
| IS | SHBG | rs329122 | 0.86(0.77,0.95) |
| IS | SHBG | rs33807 | 0.86(0.78,0.95) |
| IS | SHBG | rs33999979 | 0.86(0.77,0.95) |
| IS | SHBG | rs34010237 | 0.86(0.77,0.95) |
| IS | SHBG | rs340835 | 0.86(0.77,0.95) |
| IS | SHBG | rs34255979 | 0.86(0.77,0.95) |
| IS | SHBG | rs34311866 | 0.86(0.78,0.95) |
| IS | SHBG | rs34325 | 0.86(0.77,0.95) |
| IS | SHBG | rs34372369 | 0.86(0.77,0.95) |
| IS | SHBG | rs34587839 | 0.86(0.77,0.95) |
| IS | SHBG | rs34651 | 0.86(0.77,0.95) |
| IS | SHBG | rs3468 | 0.86(0.77,0.95) |
| IS | SHBG | rs34707604 | 0.86(0.77,0.95) |
| IS | SHBG | rs34880012 | 0.86(0.78,0.95) |
| IS | SHBG | rs34970607 | 0.86(0.78,0.95) |
| IS | SHBG | rs35070405 | 0.86(0.77,0.95) |
| IS | SHBG | rs350832 | 0.86(0.78,0.96) |
| IS | SHBG | rs35102588 | 0.86(0.78,0.96) |
| IS | SHBG | rs35226891 | 0.86(0.78,0.95) |
| IS | SHBG | rs35233014 | 0.86(0.77,0.95) |
| IS | SHBG | rs35333155 | 0.86(0.77,0.95) |
| IS | SHBG | rs35346083 | 0.86(0.78,0.96) |
| IS | SHBG | rs35391516 | 0.86(0.77,0.95) |
| IS | SHBG | rs35547626 | 0.86(0.77,0.95) |
| IS | SHBG | rs35598889 | 0.86(0.77,0.95) |
| IS | SHBG | rs35633876 | 0.86(0.77,0.95) |
| IS | SHBG | rs35812759 | 0.86(0.77,0.95) |
| IS | SHBG | rs359431 | 0.86(0.77,0.95) |
| IS | SHBG | rs35983031 | 0.86(0.77,0.95) |
| IS | SHBG | rs36086195 | 0.86(0.77,0.95) |
| IS | SHBG | rs36124182 | 0.86(0.77,0.95) |
| IS | SHBG | rs362413 | 0.86(0.77,0.95) |
| IS | SHBG | rs368214 | 0.86(0.78,0.95) |
| IS | SHBG | rs3733892 | 0.86(0.77,0.95) |
| IS | SHBG | rs3737178 | 0.86(0.77,0.95) |
| IS | SHBG | rs3741368 | 0.86(0.77,0.95) |
| IS | SHBG | rs3742366 | 0.86(0.77,0.95) |
| IS | SHBG | rs3743588 | 0.86(0.77,0.95) |
| IS | SHBG | rs3761706 | 0.86(0.77,0.95) |
| IS | SHBG | rs3764002 | 0.86(0.77,0.95) |
| IS | SHBG | rs3770781 | 0.86(0.77,0.95) |
| IS | SHBG | rs3780190 | 0.85(0.77,0.95) |
| IS | SHBG | rs3782735 | 0.86(0.77,0.95) |
| IS | SHBG | rs3813498 | 0.86(0.77,0.95) |
| IS | SHBG | rs3829639 | 0.86(0.77,0.95) |
| IS | SHBG | rs38304 | 0.86(0.77,0.95) |
| IS | SHBG | rs3848119 | 0.86(0.77,0.95) |
| IS | SHBG | rs3848125 | 0.86(0.77,0.95) |
| IS | SHBG | rs38855 | 0.86(0.77,0.95) |
| IS | SHBG | rs40270 | 0.86(0.78,0.95) |
| IS | SHBG | rs403694 | 0.86(0.77,0.95) |
| IS | SHBG | rs4073358 | 0.86(0.77,0.95) |
| IS | SHBG | rs4092465 | 0.86(0.77,0.95) |
| IS | SHBG | rs41280463 | 0.86(0.78,0.95) |
| IS | SHBG | rs41309159 | 0.86(0.78,0.96) |
| IS | SHBG | rs4147563 | 0.86(0.78,0.95) |
| IS | SHBG | rs4147913 | 0.86(0.77,0.95) |
| IS | SHBG | rs42238 | 0.86(0.78,0.95) |
| IS | SHBG | rs4274814 | 0.86(0.78,0.95) |
| IS | SHBG | rs4297769 | 0.86(0.78,0.95) |
| IS | SHBG | rs4300303 | 0.86(0.77,0.95) |
| IS | SHBG | rs4309185 | 0.86(0.78,0.95) |
| IS | SHBG | rs4338849 | 0.86(0.78,0.95) |
| IS | SHBG | rs434325 | 0.86(0.78,0.95) |
| IS | SHBG | rs4381470 | 0.86(0.78,0.95) |
| IS | SHBG | rs445 | 0.86(0.77,0.95) |
| IS | SHBG | rs45512696 | 0.86(0.77,0.95) |
| IS | SHBG | rs4568281 | 0.86(0.78,0.95) |
| IS | SHBG | rs4599176 | 0.86(0.77,0.95) |
| IS | SHBG | rs4639796 | 0.86(0.77,0.95) |
| IS | SHBG | rs4665710 | 0.86(0.78,0.95) |
| IS | SHBG | rs4665972 | 0.86(0.78,0.96) |
| IS | SHBG | rs4671328 | 0.86(0.77,0.95) |
| IS | SHBG | rs4674669 | 0.86(0.77,0.95) |
| IS | SHBG | rs4680 | 0.86(0.77,0.95) |
| IS | SHBG | rs4709746 | 0.86(0.78,0.95) |
| IS | SHBG | rs4745876 | 0.86(0.77,0.95) |
| IS | SHBG | rs4762962 | 0.86(0.78,0.95) |
| IS | SHBG | rs4794008 | 0.86(0.77,0.95) |
| IS | SHBG | rs4804414 | 0.86(0.77,0.95) |
| IS | SHBG | rs4811050 | 0.86(0.77,0.95) |
| IS | SHBG | rs4812336 | 0.86(0.78,0.95) |
| IS | SHBG | rs4820091 | 0.86(0.77,0.95) |
| IS | SHBG | rs483082 | 0.86(0.78,0.96) |
| IS | SHBG | rs4837794 | 0.86(0.77,0.95) |
| IS | SHBG | rs4841133 | 0.86(0.78,0.95) |
| IS | SHBG | rs484943 | 0.86(0.78,0.95) |
| IS | SHBG | rs4970837 | 0.86(0.77,0.95) |
| IS | SHBG | rs4974310 | 0.86(0.77,0.95) |
| IS | SHBG | rs4976033 | 0.86(0.77,0.95) |
| IS | SHBG | rs5017726 | 0.86(0.77,0.95) |
| IS | SHBG | rs55737395 | 0.86(0.77,0.95) |
| IS | SHBG | rs55761545 | 0.86(0.77,0.95) |
| IS | SHBG | rs55771168 | 0.86(0.77,0.95) |
| IS | SHBG | rs55831924 | 0.86(0.77,0.95) |
| IS | SHBG | rs55910553 | 0.86(0.77,0.95) |
| IS | SHBG | rs55974289 | 0.86(0.77,0.95) |
| IS | SHBG | rs55987409 | 0.86(0.77,0.95) |
| IS | SHBG | rs559986 | 0.86(0.77,0.95) |
| IS | SHBG | rs56021343 | 0.86(0.77,0.95) |
| IS | SHBG | rs56196860 | 0.86(0.77,0.95) |
| IS | SHBG | rs56292801 | 0.86(0.77,0.95) |
| IS | SHBG | rs56332871 | 0.85(0.76,0.94) |
| IS | SHBG | rs56365029 | 0.86(0.78,0.95) |
| IS | SHBG | rs57158761 | 0.86(0.78,0.95) |
| IS | SHBG | rs57467915 | 0.86(0.77,0.95) |
| IS | SHBG | rs575452 | 0.86(0.78,0.96) |
| IS | SHBG | rs5760120 | 0.86(0.78,0.95) |
| IS | SHBG | rs57754494 | 0.86(0.77,0.95) |
| IS | SHBG | rs58321169 | 0.86(0.78,0.95) |
| IS | SHBG | rs58429317 | 0.86(0.77,0.95) |
| IS | SHBG | rs591939 | 0.86(0.77,0.95) |
| IS | SHBG | rs59662471 | 0.86(0.77,0.95) |
| IS | SHBG | rs59708846 | 0.86(0.77,0.95) |
| IS | SHBG | rs59708898 | 0.86(0.77,0.95) |
| IS | SHBG | rs60018147 | 0.86(0.77,0.95) |
| IS | SHBG | rs6018424 | 0.86(0.77,0.95) |
| IS | SHBG | rs6048205 | 0.86(0.77,0.95) |
| IS | SHBG | rs6062381 | 0.86(0.77,0.95) |
| IS | SHBG | rs6073431 | 0.86(0.77,0.95) |
| IS | SHBG | rs608300 | 0.86(0.77,0.95) |
| IS | SHBG | rs6118 | 0.86(0.77,0.95) |
| IS | SHBG | rs6120663 | 0.86(0.78,0.95) |
| IS | SHBG | rs61292904 | 0.86(0.77,0.95) |
| IS | SHBG | rs6129802 | 0.86(0.77,0.95) |
| IS | SHBG | rs61599759 | 0.86(0.78,0.95) |
| IS | SHBG | rs61733486 | 0.86(0.77,0.95) |
| IS | SHBG | rs61759532 | 0.86(0.77,0.95) |
| IS | SHBG | rs61779331 | 0.86(0.77,0.95) |
| IS | SHBG | rs61853560 | 0.86(0.77,0.95) |
| IS | SHBG | rs61854630 | 0.86(0.77,0.95) |
| IS | SHBG | rs61856602 | 0.86(0.77,0.95) |
| IS | SHBG | rs619526 | 0.86(0.78,0.95) |
| IS | SHBG | rs62012946 | 0.86(0.77,0.95) |
| IS | SHBG | rs62037803 | 0.86(0.77,0.95) |
| IS | SHBG | rs62111692 | 0.86(0.77,0.95) |
| IS | SHBG | rs62182125 | 0.86(0.77,0.95) |
| IS | SHBG | rs62195072 | 0.86(0.77,0.95) |
| IS | SHBG | rs62334584 | 0.86(0.78,0.95) |
| IS | SHBG | rs62396733 | 0.86(0.77,0.95) |
| IS | SHBG | rs62515079 | 0.86(0.77,0.95) |
| IS | SHBG | rs62565259 | 0.86(0.77,0.95) |
| IS | SHBG | rs62580766 | 0.86(0.77,0.95) |
| IS | SHBG | rs62618693 | 0.86(0.78,0.95) |
| IS | SHBG | rs6422513 | 0.86(0.78,0.95) |
| IS | SHBG | rs6476065 | 0.86(0.77,0.95) |
| IS | SHBG | rs6486122 | 0.86(0.78,0.95) |
| IS | SHBG | rs6495962 | 0.86(0.78,0.95) |
| IS | SHBG | rs6546096 | 0.86(0.77,0.95) |
| IS | SHBG | rs6556402 | 0.86(0.78,0.95) |
| IS | SHBG | rs6567160 | 0.86(0.77,0.95) |
| IS | SHBG | rs6575439 | 0.86(0.77,0.95) |
| IS | SHBG | rs662026 | 0.86(0.77,0.95) |
| IS | SHBG | rs6684464 | 0.86(0.78,0.95) |
| IS | SHBG | rs668871 | 0.86(0.77,0.95) |
| IS | SHBG | rs66921136 | 0.86(0.77,0.95) |
| IS | SHBG | rs6710171 | 0.86(0.77,0.95) |
| IS | SHBG | rs671948 | 0.86(0.77,0.95) |
| IS | SHBG | rs6736913 | 0.86(0.77,0.95) |
| IS | SHBG | rs6741180 | 0.86(0.78,0.95) |
| IS | SHBG | rs6755571 | 0.86(0.78,0.95) |
| IS | SHBG | rs6791074 | 0.86(0.77,0.95) |
| IS | SHBG | rs6792725 | 0.86(0.77,0.95) |
| IS | SHBG | rs6804915 | 0.86(0.77,0.95) |
| IS | SHBG | rs68062403 | 0.86(0.77,0.95) |
| IS | SHBG | rs687339 | 0.86(0.77,0.95) |
| IS | SHBG | rs6910879 | 0.86(0.77,0.95) |
| IS | SHBG | rs6939861 | 0.86(0.77,0.95) |
| IS | SHBG | rs695272 | 0.86(0.78,0.96) |
| IS | SHBG | rs7000496 | 0.86(0.77,0.95) |
| IS | SHBG | rs700085 | 0.86(0.78,0.95) |
| IS | SHBG | rs7015 | 0.87(0.78,0.96) |
| IS | SHBG | rs702876 | 0.86(0.77,0.95) |
| IS | SHBG | rs7117818 | 0.86(0.78,0.95) |
| IS | SHBG | rs7123361 | 0.86(0.77,0.95) |
| IS | SHBG | rs7131509 | 0.86(0.78,0.95) |
| IS | SHBG | rs715 | 0.86(0.77,0.95) |
| IS | SHBG | rs7157184 | 0.86(0.77,0.95) |
| IS | SHBG | rs7164175 | 0.86(0.77,0.95) |
| IS | SHBG | rs7183456 | 0.86(0.77,0.95) |
| IS | SHBG | rs720130 | 0.86(0.77,0.95) |
| IS | SHBG | rs7221716 | 0.86(0.77,0.95) |
| IS | SHBG | rs7250869 | 0.86(0.77,0.95) |
| IS | SHBG | rs7262150 | 0.86(0.78,0.95) |
| IS | SHBG | rs72663937 | 0.86(0.77,0.95) |
| IS | SHBG | rs72666817 | 0.86(0.77,0.95) |
| IS | SHBG | rs72683923 | 0.86(0.78,0.95) |
| IS | SHBG | rs72694845 | 0.86(0.77,0.95) |
| IS | SHBG | rs72709458 | 0.86(0.77,0.95) |
| IS | SHBG | rs72753349 | 0.86(0.77,0.95) |
| IS | SHBG | rs72753908 | 0.86(0.77,0.95) |
| IS | SHBG | rs72766607 | 0.86(0.78,0.95) |
| IS | SHBG | rs72767773 | 0.86(0.77,0.95) |
| IS | SHBG | rs72810505 | 0.86(0.77,0.95) |
| IS | SHBG | rs72815155 | 0.86(0.77,0.95) |
| IS | SHBG | rs72840987 | 0.86(0.77,0.95) |
| IS | SHBG | rs72842808 | 0.86(0.78,0.95) |
| IS | SHBG | rs72844546 | 0.85(0.77,0.95) |
| IS | SHBG | rs7298924 | 0.86(0.78,0.96) |
| IS | SHBG | rs7301634 | 0.86(0.78,0.95) |
| IS | SHBG | rs73047887 | 0.86(0.77,0.95) |
| IS | SHBG | rs7308634 | 0.86(0.78,0.95) |
| IS | SHBG | rs7314285 | 0.87(0.78,0.96) |
| IS | SHBG | rs73193388 | 0.86(0.78,0.95) |
| IS | SHBG | rs73223295 | 0.86(0.77,0.95) |
| IS | SHBG | rs7323372 | 0.86(0.77,0.95) |
| IS | SHBG | rs73375029 | 0.86(0.77,0.95) |
| IS | SHBG | rs73597479 | 0.86(0.77,0.95) |
| IS | SHBG | rs73705826 | 0.86(0.78,0.95) |
| IS | SHBG | rs73972648 | 0.86(0.77,0.95) |
| IS | SHBG | rs7406661 | 0.86(0.77,0.95) |
| IS | SHBG | rs744200 | 0.86(0.77,0.95) |
| IS | SHBG | rs7451021 | 0.86(0.78,0.96) |
| IS | SHBG | rs74551598 | 0.86(0.77,0.95) |
| IS | SHBG | rs74998771 | 0.86(0.77,0.95) |
| IS | SHBG | rs750155 | 0.86(0.77,0.95) |
| IS | SHBG | rs75077113 | 0.86(0.77,0.95) |
| IS | SHBG | rs75156222 | 0.86(0.77,0.95) |
| IS | SHBG | rs7535528 | 0.86(0.78,0.95) |
| IS | SHBG | rs7540115 | 0.86(0.77,0.95) |
| IS | SHBG | rs7552207 | 0.86(0.77,0.95) |
| IS | SHBG | rs75713100 | 0.86(0.77,0.95) |
| IS | SHBG | rs757869 | 0.86(0.77,0.95) |
| IS | SHBG | rs759404 | 0.86(0.77,0.95) |
| IS | SHBG | rs7617967 | 0.86(0.78,0.95) |
| IS | SHBG | rs76345703 | 0.86(0.77,0.95) |
| IS | SHBG | rs7638782 | 0.86(0.77,0.95) |
| IS | SHBG | rs76475417 | 0.86(0.77,0.95) |
| IS | SHBG | rs76549335 | 0.86(0.77,0.95) |
| IS | SHBG | rs7668413 | 0.86(0.77,0.95) |
| IS | SHBG | rs76708468 | 0.86(0.77,0.95) |
| IS | SHBG | rs7678138 | 0.86(0.78,0.95) |
| IS | SHBG | rs76895963 | 0.86(0.78,0.95) |
| IS | SHBG | rs7696472 | 0.86(0.77,0.95) |
| IS | SHBG | rs7756992 | 0.86(0.77,0.95) |
| IS | SHBG | rs77597993 | 0.86(0.78,0.95) |
| IS | SHBG | rs78058190 | 0.86(0.77,0.95) |
| IS | SHBG | rs7809920 | 0.86(0.78,0.95) |
| IS | SHBG | rs7828742 | 0.86(0.77,0.95) |
| IS | SHBG | rs78319058 | 0.86(0.77,0.95) |
| IS | SHBG | rs78444298 | 0.86(0.77,0.95) |
| IS | SHBG | rs78496430 | 0.86(0.78,0.96) |
| IS | SHBG | rs78536975 | 0.86(0.77,0.95) |
| IS | SHBG | rs78555071 | 0.86(0.77,0.95) |
| IS | SHBG | rs7863263 | 0.86(0.77,0.95) |
| IS | SHBG | rs787976 | 0.86(0.77,0.95) |
| IS | SHBG | rs78890745 | 0.86(0.77,0.95) |
| IS | SHBG | rs78973091 | 0.86(0.77,0.95) |
| IS | SHBG | rs7899096 | 0.86(0.77,0.95) |
| IS | SHBG | rs7918533 | 0.86(0.77,0.95) |
| IS | SHBG | rs79287178 | 0.86(0.77,0.95) |
| IS | SHBG | rs79354983 | 0.86(0.77,0.95) |
| IS | SHBG | rs79391862 | 0.86(0.77,0.95) |
| IS | SHBG | rs7944853 | 0.86(0.77,0.95) |
| IS | SHBG | rs7953508 | 0.86(0.78,0.95) |
| IS | SHBG | rs796004 | 0.86(0.77,0.95) |
| IS | SHBG | rs79600740 | 0.86(0.78,0.95) |
| IS | SHBG | rs79683734 | 0.86(0.77,0.95) |
| IS | SHBG | rs79717793 | 0.86(0.78,0.96) |
| IS | SHBG | rs79875164 | 0.86(0.78,0.95) |
| IS | SHBG | rs8001781 | 0.86(0.78,0.95) |
| IS | SHBG | rs80226362 | 0.86(0.77,0.95) |
| IS | SHBG | rs8031716 | 0.86(0.77,0.95) |
| IS | SHBG | rs8043101 | 0.86(0.77,0.95) |
| IS | SHBG | rs8066941 | 0.86(0.77,0.95) |
| IS | SHBG | rs8069105 | 0.86(0.77,0.95) |
| IS | SHBG | rs8074363 | 0.86(0.77,0.95) |
| IS | SHBG | rs8077323 | 0.86(0.78,0.95) |
| IS | SHBG | rs8079418 | 0.86(0.78,0.96) |
| IS | SHBG | rs8107967 | 0.86(0.77,0.95) |
| IS | SHBG | rs8134638 | 0.86(0.77,0.95) |
| IS | SHBG | rs8176693 | 0.86(0.77,0.95) |
| IS | SHBG | rs820503 | 0.86(0.77,0.95) |
| IS | SHBG | rs822508 | 0.86(0.78,0.95) |
| IS | SHBG | rs841194 | 0.86(0.77,0.95) |
| IS | SHBG | rs857152 | 0.86(0.77,0.95) |
| IS | SHBG | rs864899 | 0.86(0.77,0.95) |
| IS | SHBG | rs885683 | 0.86(0.78,0.96) |
| IS | SHBG | rs901886 | 0.86(0.77,0.95) |
| IS | SHBG | rs9297994 | 0.86(0.78,0.96) |
| IS | SHBG | rs9379084 | 0.86(0.77,0.95) |
| IS | SHBG | rs9427104 | 0.86(0.77,0.95) |
| IS | SHBG | rs9439469 | 0.86(0.77,0.95) |
| IS | SHBG | rs9465601 | 0.86(0.77,0.95) |
| IS | SHBG | rs9492 | 0.86(0.77,0.95) |
| IS | SHBG | rs9495298 | 0.86(0.77,0.95) |
| IS | SHBG | rs9533843 | 0.86(0.78,0.95) |
| IS | SHBG | rs9556403 | 0.86(0.77,0.95) |
| IS | SHBG | rs9597811 | 0.86(0.77,0.95) |
| IS | SHBG | rs9610329 | 0.86(0.77,0.95) |
| IS | SHBG | rs9614162 | 0.86(0.77,0.95) |
| IS | SHBG | rs9686661 | 0.86(0.78,0.95) |
| IS | SHBG | rs9697210 | 0.86(0.78,0.96) |
| IS | SHBG | rs9823108 | 0.86(0.77,0.95) |
| IS | SHBG | rs9823118 | 0.86(0.77,0.95) |
| IS | SHBG | rs9831794 | 0.86(0.77,0.95) |
| IS | SHBG | rs9871160 | 0.86(0.77,0.95) |
| IS | SHBG | rs9893194 | 0.86(0.77,0.95) |
| IS | SHBG | rs9902384 | 0.86(0.77,0.95) |
| IS | SHBG | rs9968070 | 0.86(0.77,0.95) |
| IS | SHBG | rs9972653 | 0.86(0.78,0.95) |
| IS | SHBG | rs998584 | 0.86(0.78,0.95) |
| IS | SHBG | rs999634 | 0.86(0.78,0.95) |
| LAS | SHBG | rs10026753 | 0.83(0.65,1.07) |
| LAS | SHBG | rs10041660 | 0.83(0.65,1.07) |
| LAS | SHBG | rs1005421 | 0.84(0.65,1.08) |
| LAS | SHBG | rs1007851 | 0.83(0.65,1.08) |
| LAS | SHBG | rs10084025 | 0.83(0.64,1.07) |
| LAS | SHBG | rs10095103 | 0.83(0.65,1.07) |
| LAS | SHBG | rs10110651 | 0.83(0.64,1.07) |
| LAS | SHBG | rs10111451 | 0.83(0.65,1.07) |
| LAS | SHBG | rs10123811 | 0.83(0.64,1.07) |
| LAS | SHBG | rs10125995 | 0.83(0.64,1.07) |
| LAS | SHBG | rs10153315 | 0.83(0.64,1.07) |
| LAS | SHBG | rs10163091 | 0.83(0.65,1.07) |
| LAS | SHBG | rs10187560 | 0.83(0.65,1.07) |
| LAS | SHBG | rs10208512 | 0.83(0.65,1.08) |
| LAS | SHBG | rs10238028 | 0.83(0.65,1.08) |
| LAS | SHBG | rs10258433 | 0.84(0.65,1.08) |
| LAS | SHBG | rs10273476 | 0.83(0.64,1.07) |
| LAS | SHBG | rs10411932 | 0.83(0.64,1.07) |
| LAS | SHBG | rs10413329 | 0.83(0.65,1.07) |
| LAS | SHBG | rs1042725 | 0.84(0.66,1.09) |
| LAS | SHBG | rs10432029 | 0.85(0.66,1.10) |
| LAS | SHBG | rs10492118 | 0.83(0.64,1.07) |
| LAS | SHBG | rs10504731 | 0.84(0.65,1.08) |
| LAS | SHBG | rs1059698 | 0.83(0.64,1.07) |
| LAS | SHBG | rs10733608 | 0.84(0.65,1.08) |
| LAS | SHBG | rs10761676 | 0.83(0.64,1.07) |
| LAS | SHBG | rs10761749 | 0.84(0.65,1.08) |
| LAS | SHBG | rs10773049 | 0.84(0.65,1.08) |
| LAS | SHBG | rs10794307 | 0.83(0.65,1.08) |
| LAS | SHBG | rs10815276 | 0.84(0.65,1.08) |
| LAS | SHBG | rs10822130 | 0.84(0.65,1.09) |
| LAS | SHBG | rs10824742 | 0.83(0.65,1.07) |
| LAS | SHBG | rs10838681 | 0.84(0.65,1.08) |
| LAS | SHBG | rs10864070 | 0.83(0.65,1.08) |
| LAS | SHBG | rs10880872 | 0.83(0.65,1.07) |
| LAS | SHBG | rs10888696 | 0.84(0.65,1.08) |
| LAS | SHBG | rs10895276 | 0.83(0.65,1.08) |
| LAS | SHBG | rs10900446 | 0.84(0.65,1.08) |
| LAS | SHBG | rs10946313 | 0.83(0.64,1.07) |
| LAS | SHBG | rs10951130 | 0.83(0.64,1.07) |
| LAS | SHBG | rs10964337 | 0.84(0.65,1.08) |
| LAS | SHBG | rs11029441 | 0.83(0.65,1.07) |
| LAS | SHBG | rs11030100 | 0.84(0.65,1.08) |
| LAS | SHBG | rs11032076 | 0.83(0.64,1.07) |
| LAS | SHBG | rs11038673 | 0.83(0.65,1.07) |
| LAS | SHBG | rs11078597 | 0.84(0.65,1.08) |
| LAS | SHBG | rs11078681 | 0.83(0.65,1.08) |
| LAS | SHBG | rs11078701 | 0.83(0.64,1.07) |
| LAS | SHBG | rs11079872 | 0.84(0.65,1.08) |
| LAS | SHBG | rs11108061 | 0.83(0.65,1.07) |
| LAS | SHBG | rs11110390 | 0.83(0.64,1.07) |
| LAS | SHBG | rs11111274 | 0.83(0.64,1.07) |
| LAS | SHBG | rs11121522 | 0.83(0.64,1.07) |
| LAS | SHBG | rs111289824 | 0.83(0.65,1.07) |
| LAS | SHBG | rs111331455 | 0.83(0.65,1.08) |
| LAS | SHBG | rs111363146 | 0.83(0.65,1.08) |
| LAS | SHBG | rs11153046 | 0.83(0.65,1.07) |
| LAS | SHBG | rs11155787 | 0.83(0.64,1.07) |
| LAS | SHBG | rs111604078 | 0.83(0.65,1.07) |
| LAS | SHBG | rs111637026 | 0.83(0.64,1.07) |
| LAS | SHBG | rs11164095 | 0.83(0.65,1.07) |
| LAS | SHBG | rs11165493 | 0.83(0.64,1.07) |
| LAS | SHBG | rs111700120 | 0.83(0.65,1.07) |
| LAS | SHBG | rs11187142 | 0.83(0.64,1.07) |
| LAS | SHBG | rs11188604 | 0.83(0.65,1.07) |
| LAS | SHBG | rs11190245 | 0.83(0.64,1.07) |
| LAS | SHBG | rs111905890 | 0.83(0.65,1.08) |
| LAS | SHBG | rs11191841 | 0.83(0.64,1.06) |
| LAS | SHBG | rs111981233 | 0.84(0.65,1.08) |
| LAS | SHBG | rs11202594 | 0.83(0.64,1.07) |
| LAS | SHBG | rs112035922 | 0.84(0.65,1.08) |
| LAS | SHBG | rs112575738 | 0.83(0.64,1.07) |
| LAS | SHBG | rs112672290 | 0.83(0.64,1.07) |
| LAS | SHBG | rs112833123 | 0.83(0.64,1.07) |
| LAS | SHBG | rs112850234 | 0.83(0.65,1.07) |
| LAS | SHBG | rs112928223 | 0.83(0.64,1.07) |
| LAS | SHBG | rs113251204 | 0.83(0.65,1.08) |
| LAS | SHBG | rs113364399 | 0.83(0.65,1.07) |
| LAS | SHBG | rs113523273 | 0.83(0.64,1.07) |
| LAS | SHBG | rs113973451 | 0.83(0.64,1.07) |
| LAS | SHBG | rs114053844 | 0.83(0.64,1.07) |
| LAS | SHBG | rs114469183 | 0.83(0.65,1.07) |
| LAS | SHBG | rs114627598 | 0.83(0.64,1.07) |
| LAS | SHBG | rs114940462 | 0.83(0.64,1.07) |
| LAS | SHBG | rs114949263 | 0.82(0.64,1.06) |
| LAS | SHBG | rs11539938 | 0.83(0.65,1.08) |
| LAS | SHBG | rs11542663 | 0.83(0.65,1.07) |
| LAS | SHBG | rs11545185 | 0.83(0.64,1.07) |
| LAS | SHBG | rs115521489 | 0.83(0.65,1.07) |
| LAS | SHBG | rs11552708 | 0.82(0.63,1.06) |
| LAS | SHBG | rs11556924 | 0.84(0.65,1.08) |
| LAS | SHBG | rs11564722 | 0.84(0.65,1.08) |
| LAS | SHBG | rs11601507 | 0.83(0.64,1.07) |
| LAS | SHBG | rs11621792 | 0.84(0.65,1.08) |
| LAS | SHBG | rs11633147 | 0.83(0.64,1.07) |
| LAS | SHBG | rs116573491 | 0.83(0.65,1.08) |
| LAS | SHBG | rs116713089 | 0.83(0.65,1.07) |
| LAS | SHBG | rs11682084 | 0.83(0.65,1.07) |
| LAS | SHBG | rs11690176 | 0.83(0.64,1.07) |
| LAS | SHBG | rs117108573 | 0.83(0.65,1.07) |
| LAS | SHBG | rs117135073 | 0.83(0.64,1.07) |
| LAS | SHBG | rs117169274 | 0.84(0.65,1.08) |
| LAS | SHBG | rs117411982 | 0.83(0.65,1.08) |
| LAS | SHBG | rs11743810 | 0.83(0.64,1.07) |
| LAS | SHBG | rs11748938 | 0.83(0.64,1.07) |
| LAS | SHBG | rs117589665 | 0.83(0.65,1.08) |
| LAS | SHBG | rs11765639 | 0.83(0.65,1.07) |
| LAS | SHBG | rs11791747 | 0.83(0.65,1.07) |
| LAS | SHBG | rs118080406 | 0.83(0.64,1.07) |
| LAS | SHBG | rs11856606 | 0.83(0.65,1.07) |
| LAS | SHBG | rs11856926 | 0.83(0.64,1.07) |
| LAS | SHBG | rs11994858 | 0.83(0.65,1.08) |
| LAS | SHBG | rs11997548 | 0.84(0.65,1.08) |
| LAS | SHBG | rs12059956 | 0.83(0.64,1.07) |
| LAS | SHBG | rs12138461 | 0.83(0.65,1.07) |
| LAS | SHBG | rs12185242 | 0.83(0.65,1.08) |
| LAS | SHBG | rs12263369 | 0.83(0.64,1.07) |
| LAS | SHBG | rs12280075 | 0.83(0.64,1.07) |
| LAS | SHBG | rs1229498 | 0.83(0.65,1.07) |
| LAS | SHBG | rs12302952 | 0.83(0.64,1.07) |
| LAS | SHBG | rs12311848 | 0.84(0.65,1.08) |
| LAS | SHBG | rs12373799 | 0.83(0.65,1.07) |
| LAS | SHBG | rs12413488 | 0.83(0.64,1.07) |
| LAS | SHBG | rs12424336 | 0.83(0.64,1.07) |
| LAS | SHBG | rs12454712 | 0.83(0.64,1.06) |
| LAS | SHBG | rs12476661 | 0.83(0.65,1.07) |
| LAS | SHBG | rs12575636 | 0.83(0.64,1.07) |
| LAS | SHBG | rs12601778 | 0.83(0.65,1.07) |
| LAS | SHBG | rs12636106 | 0.83(0.64,1.06) |
| LAS | SHBG | rs12667888 | 0.84(0.65,1.08) |
| LAS | SHBG | rs12758998 | 0.83(0.64,1.07) |
| LAS | SHBG | rs12797706 | 0.84(0.65,1.09) |
| LAS | SHBG | rs12809946 | 0.83(0.64,1.07) |
| LAS | SHBG | rs12818938 | 0.83(0.64,1.07) |
| LAS | SHBG | rs12879423 | 0.83(0.65,1.07) |
| LAS | SHBG | rs12916 | 0.83(0.65,1.08) |
| LAS | SHBG | rs12926107 | 0.83(0.64,1.07) |
| LAS | SHBG | rs12928099 | 0.84(0.65,1.08) |
| LAS | SHBG | rs12965052 | 0.84(0.65,1.08) |
| LAS | SHBG | rs12989083 | 0.83(0.64,1.07) |
| LAS | SHBG | rs13000027 | 0.83(0.65,1.07) |
| LAS | SHBG | rs13035806 | 0.83(0.65,1.08) |
| LAS | SHBG | rs13057133 | 0.83(0.64,1.07) |
| LAS | SHBG | rs13086465 | 0.84(0.65,1.08) |
| LAS | SHBG | rs13094241 | 0.83(0.65,1.07) |
| LAS | SHBG | rs13108218 | 0.81(0.63,1.04) |
| LAS | SHBG | rs13149606 | 0.83(0.64,1.07) |
| LAS | SHBG | rs13150068 | 0.83(0.64,1.07) |
| LAS | SHBG | rs13251458 | 0.83(0.64,1.07) |
| LAS | SHBG | rs1330307 | 0.84(0.65,1.08) |
| LAS | SHBG | rs13315174 | 0.83(0.65,1.07) |
| LAS | SHBG | rs13379043 | 0.83(0.65,1.07) |
| LAS | SHBG | rs13389219 | 0.84(0.65,1.09) |
| LAS | SHBG | rs13405815 | 0.84(0.65,1.08) |
| LAS | SHBG | rs1352084 | 0.83(0.65,1.08) |
| LAS | SHBG | rs138526953 | 0.83(0.64,1.07) |
| LAS | SHBG | rs138755456 | 0.83(0.65,1.07) |
| LAS | SHBG | rs139805419 | 0.83(0.65,1.07) |
| LAS | SHBG | rs139974673 | 0.85(0.66,1.10) |
| LAS | SHBG | rs140105410 | 0.83(0.64,1.07) |
| LAS | SHBG | rs1408270 | 0.84(0.65,1.08) |
| LAS | SHBG | rs1411432 | 0.83(0.65,1.08) |
| LAS | SHBG | rs14129 | 0.84(0.65,1.08) |
| LAS | SHBG | rs141899843 | 0.83(0.64,1.07) |
| LAS | SHBG | rs142035705 | 0.83(0.65,1.08) |
| LAS | SHBG | rs1420385 | 0.83(0.64,1.07) |
| LAS | SHBG | rs142331290 | 0.83(0.64,1.07) |
| LAS | SHBG | rs1431659 | 0.83(0.65,1.08) |
| LAS | SHBG | rs1433210 | 0.83(0.64,1.07) |
| LAS | SHBG | rs143709973 | 0.83(0.64,1.07) |
| LAS | SHBG | rs144459202 | 0.83(0.64,1.07) |
| LAS | SHBG | rs144989856 | 0.83(0.64,1.07) |
| LAS | SHBG | rs145931818 | 0.83(0.65,1.07) |
| LAS | SHBG | rs147259681 | 0.83(0.65,1.08) |
| LAS | SHBG | rs148118632 | 0.83(0.64,1.07) |
| LAS | SHBG | rs149092986 | 0.83(0.65,1.07) |
| LAS | SHBG | rs149102638 | 0.83(0.64,1.07) |
| LAS | SHBG | rs150895955 | 0.83(0.65,1.08) |
| LAS | SHBG | rs1530439 | 0.83(0.64,1.07) |
| LAS | SHBG | rs1534696 | 0.83(0.65,1.07) |
| LAS | SHBG | rs1561442 | 0.84(0.65,1.08) |
| LAS | SHBG | rs1570360 | 0.84(0.65,1.08) |
| LAS | SHBG | rs157935 | 0.83(0.65,1.07) |
| LAS | SHBG | rs1640269 | 0.83(0.64,1.07) |
| LAS | SHBG | rs16835135 | 0.83(0.65,1.08) |
| LAS | SHBG | rs16934748 | 0.83(0.64,1.07) |
| LAS | SHBG | rs17008851 | 0.83(0.64,1.06) |
| LAS | SHBG | rs1708302 | 0.83(0.65,1.07) |
| LAS | SHBG | rs17184382 | 0.83(0.65,1.08) |
| LAS | SHBG | rs17207107 | 0.83(0.65,1.08) |
| LAS | SHBG | rs1730862 | 0.82(0.64,1.07) |
| LAS | SHBG | rs17356664 | 0.83(0.65,1.08) |
| LAS | SHBG | rs17372936 | 0.84(0.65,1.08) |
| LAS | SHBG | rs1738380 | 0.83(0.65,1.07) |
| LAS | SHBG | rs1743954 | 0.83(0.65,1.08) |
| LAS | SHBG | rs174554 | 0.82(0.63,1.05) |
| LAS | SHBG | rs17583875 | 0.83(0.64,1.07) |
| LAS | SHBG | rs17592998 | 0.83(0.64,1.07) |
| LAS | SHBG | rs17669311 | 0.82(0.64,1.06) |
| LAS | SHBG | rs1775125 | 0.83(0.64,1.07) |
| LAS | SHBG | rs17751614 | 0.83(0.65,1.07) |
| LAS | SHBG | rs17755271 | 0.83(0.65,1.07) |
| LAS | SHBG | rs17881850 | 0.82(0.64,1.06) |
| LAS | SHBG | rs1799831 | 0.84(0.65,1.08) |
| LAS | SHBG | rs1801689 | 0.84(0.65,1.08) |
| LAS | SHBG | rs182132993 | 0.83(0.65,1.08) |
| LAS | SHBG | rs1823227 | 0.83(0.64,1.07) |
| LAS | SHBG | rs182848434 | 0.83(0.64,1.07) |
| LAS | SHBG | rs183015141 | 0.83(0.64,1.07) |
| LAS | SHBG | rs184304 | 0.83(0.65,1.07) |
| LAS | SHBG | rs185406435 | 0.83(0.65,1.08) |
| LAS | SHBG | rs1864390 | 0.82(0.63,1.06) |
| LAS | SHBG | rs186766320 | 0.83(0.64,1.07) |
| LAS | SHBG | rs1883783 | 0.84(0.65,1.08) |
| LAS | SHBG | rs188889872 | 0.83(0.64,1.07) |
| LAS | SHBG | rs188949713 | 0.83(0.64,1.07) |
| LAS | SHBG | rs189595752 | 0.83(0.64,1.07) |
| LAS | SHBG | rs190712219 | 0.82(0.64,1.06) |
| LAS | SHBG | rs1951244 | 0.83(0.64,1.07) |
| LAS | SHBG | rs1969213 | 0.83(0.65,1.08) |
| LAS | SHBG | rs1993669 | 0.83(0.65,1.07) |
| LAS | SHBG | rs201570119 | 0.83(0.65,1.08) |
| LAS | SHBG | rs2022865 | 0.83(0.65,1.07) |
| LAS | SHBG | rs203777 | 0.83(0.65,1.07) |
| LAS | SHBG | rs2063245 | 0.83(0.65,1.07) |
| LAS | SHBG | rs2074683 | 0.83(0.64,1.07) |
| LAS | SHBG | rs2075915 | 0.83(0.65,1.07) |
| LAS | SHBG | rs2121650 | 0.84(0.65,1.08) |
| LAS | SHBG | rs2122982 | 0.83(0.64,1.07) |
| LAS | SHBG | rs2156804 | 0.83(0.65,1.08) |
| LAS | SHBG | rs2176887 | 0.83(0.64,1.07) |
| LAS | SHBG | rs2196943 | 0.83(0.65,1.07) |
| LAS | SHBG | rs2233364 | 0.83(0.65,1.08) |
| LAS | SHBG | rs2234922 | 0.83(0.64,1.07) |
| LAS | SHBG | rs2239222 | 0.83(0.65,1.08) |
| LAS | SHBG | rs2241261 | 0.83(0.65,1.07) |
| LAS | SHBG | rs2246223 | 0.83(0.65,1.08) |
| LAS | SHBG | rs2273368 | 0.83(0.65,1.07) |
| LAS | SHBG | rs2275355 | 0.83(0.65,1.08) |
| LAS | SHBG | rs2277283 | 0.84(0.65,1.08) |
| LAS | SHBG | rs2277641 | 0.83(0.64,1.07) |
| LAS | SHBG | rs2280838 | 0.83(0.64,1.07) |
| LAS | SHBG | rs2304686 | 0.83(0.65,1.07) |
| LAS | SHBG | rs2305144 | 0.83(0.65,1.07) |
| LAS | SHBG | rs234051 | 0.83(0.65,1.07) |
| LAS | SHBG | rs2351958 | 0.83(0.65,1.08) |
| LAS | SHBG | rs237438 | 0.83(0.65,1.08) |
| LAS | SHBG | rs2393791 | 0.83(0.64,1.07) |
| LAS | SHBG | rs2404976 | 0.83(0.65,1.08) |
| LAS | SHBG | rs2431752 | 0.83(0.64,1.07) |
| LAS | SHBG | rs2450128 | 0.83(0.64,1.07) |
| LAS | SHBG | rs2459974 | 0.84(0.65,1.08) |
| LAS | SHBG | rs2522054 | 0.84(0.65,1.08) |
| LAS | SHBG | rs2535404 | 0.83(0.65,1.07) |
| LAS | SHBG | rs2551774 | 0.83(0.64,1.07) |
| LAS | SHBG | rs2602856 | 0.83(0.64,1.07) |
| LAS | SHBG | rs2618566 | 0.83(0.64,1.07) |
| LAS | SHBG | rs2627690 | 0.83(0.65,1.08) |
| LAS | SHBG | rs2642438 | 0.83(0.64,1.07) |
| LAS | SHBG | rs267733 | 0.83(0.65,1.08) |
| LAS | SHBG | rs2705619 | 0.83(0.64,1.07) |
| LAS | SHBG | rs2723067 | 0.83(0.65,1.07) |
| LAS | SHBG | rs2724475 | 0.83(0.65,1.08) |
| LAS | SHBG | rs2746829 | 0.83(0.65,1.07) |
| LAS | SHBG | rs2792022 | 0.83(0.64,1.07) |
| LAS | SHBG | rs2807861 | 0.83(0.64,1.07) |
| LAS | SHBG | rs28507491 | 0.83(0.65,1.08) |
| LAS | SHBG | rs28562483 | 0.84(0.65,1.08) |
| LAS | SHBG | rs2862954 | 0.83(0.64,1.07) |
| LAS | SHBG | rs2885582 | 0.83(0.64,1.07) |
| LAS | SHBG | rs28890929 | 0.83(0.65,1.07) |
| LAS | SHBG | rs28925904 | 0.83(0.64,1.07) |
| LAS | SHBG | rs28929474 | 0.83(0.64,1.07) |
| LAS | SHBG | rs2914005 | 0.84(0.65,1.08) |
| LAS | SHBG | rs2925979 | 0.84(0.65,1.08) |
| LAS | SHBG | rs2942202 | 0.84(0.65,1.08) |
| LAS | SHBG | rs2965196 | 0.83(0.64,1.07) |
| LAS | SHBG | rs2970877 | 0.83(0.64,1.07) |
| LAS | SHBG | rs2972145 | 0.84(0.65,1.08) |
| LAS | SHBG | rs2972438 | 0.83(0.64,1.07) |
| LAS | SHBG | rs3001032 | 0.83(0.64,1.07) |
| LAS | SHBG | rs3004179 | 0.83(0.65,1.07) |
| LAS | SHBG | rs3116625 | 0.83(0.64,1.07) |
| LAS | SHBG | rs3217860 | 0.83(0.65,1.08) |
| LAS | SHBG | rs329122 | 0.83(0.65,1.08) |
| LAS | SHBG | rs33807 | 0.83(0.64,1.07) |
| LAS | SHBG | rs33999979 | 0.83(0.64,1.07) |
| LAS | SHBG | rs34010237 | 0.83(0.65,1.08) |
| LAS | SHBG | rs340835 | 0.83(0.64,1.06) |
| LAS | SHBG | rs34255979 | 0.83(0.64,1.07) |
| LAS | SHBG | rs34311866 | 0.84(0.65,1.08) |
| LAS | SHBG | rs34325 | 0.83(0.65,1.07) |
| LAS | SHBG | rs34372369 | 0.83(0.64,1.07) |
| LAS | SHBG | rs34587839 | 0.83(0.64,1.07) |
| LAS | SHBG | rs34651 | 0.83(0.65,1.07) |
| LAS | SHBG | rs3468 | 0.84(0.65,1.08) |
| LAS | SHBG | rs34707604 | 0.83(0.64,1.07) |
| LAS | SHBG | rs34880012 | 0.83(0.65,1.08) |
| LAS | SHBG | rs34970607 | 0.84(0.65,1.08) |
| LAS | SHBG | rs35070405 | 0.83(0.64,1.07) |
| LAS | SHBG | rs350832 | 0.83(0.65,1.07) |
| LAS | SHBG | rs35102588 | 0.84(0.65,1.08) |
| LAS | SHBG | rs35226891 | 0.83(0.65,1.07) |
| LAS | SHBG | rs35233014 | 0.83(0.64,1.07) |
| LAS | SHBG | rs35333155 | 0.83(0.65,1.07) |
| LAS | SHBG | rs35346083 | 0.83(0.65,1.07) |
| LAS | SHBG | rs35391516 | 0.83(0.65,1.07) |
| LAS | SHBG | rs35547626 | 0.84(0.65,1.09) |
| LAS | SHBG | rs35598889 | 0.84(0.65,1.08) |
| LAS | SHBG | rs35633876 | 0.83(0.64,1.07) |
| LAS | SHBG | rs35812759 | 0.83(0.65,1.08) |
| LAS | SHBG | rs359431 | 0.83(0.64,1.07) |
| LAS | SHBG | rs35983031 | 0.83(0.64,1.07) |
| LAS | SHBG | rs36086195 | 0.82(0.64,1.06) |
| LAS | SHBG | rs36124182 | 0.82(0.64,1.06) |
| LAS | SHBG | rs362413 | 0.83(0.65,1.08) |
| LAS | SHBG | rs368214 | 0.84(0.65,1.08) |
| LAS | SHBG | rs3733892 | 0.84(0.65,1.08) |
| LAS | SHBG | rs3737178 | 0.83(0.64,1.07) |
| LAS | SHBG | rs3741368 | 0.83(0.64,1.07) |
| LAS | SHBG | rs3742366 | 0.83(0.65,1.07) |
| LAS | SHBG | rs3743588 | 0.83(0.64,1.06) |
| LAS | SHBG | rs3761706 | 0.83(0.65,1.07) |
| LAS | SHBG | rs3764002 | 0.83(0.65,1.07) |
| LAS | SHBG | rs3770781 | 0.83(0.64,1.07) |
| LAS | SHBG | rs3780190 | 0.83(0.64,1.07) |
| LAS | SHBG | rs3782735 | 0.83(0.64,1.07) |
| LAS | SHBG | rs3813498 | 0.83(0.65,1.07) |
| LAS | SHBG | rs3829639 | 0.83(0.64,1.07) |
| LAS | SHBG | rs38304 | 0.83(0.64,1.07) |
| LAS | SHBG | rs3848119 | 0.83(0.64,1.07) |
| LAS | SHBG | rs3848125 | 0.83(0.64,1.07) |
| LAS | SHBG | rs38855 | 0.83(0.65,1.07) |
| LAS | SHBG | rs40270 | 0.83(0.64,1.07) |
| LAS | SHBG | rs403694 | 0.83(0.65,1.08) |
| LAS | SHBG | rs4073358 | 0.84(0.65,1.08) |
| LAS | SHBG | rs4092465 | 0.83(0.64,1.07) |
| LAS | SHBG | rs41280463 | 0.83(0.65,1.08) |
| LAS | SHBG | rs41309159 | 0.84(0.65,1.08) |
| LAS | SHBG | rs4135240 | 0.84(0.65,1.08) |
| LAS | SHBG | rs4147563 | 0.83(0.64,1.07) |
| LAS | SHBG | rs4147913 | 0.83(0.64,1.07) |
| LAS | SHBG | rs42238 | 0.83(0.64,1.07) |
| LAS | SHBG | rs4274814 | 0.83(0.65,1.08) |
| LAS | SHBG | rs4297769 | 0.84(0.65,1.08) |
| LAS | SHBG | rs4300303 | 0.84(0.65,1.08) |
| LAS | SHBG | rs4309185 | 0.83(0.65,1.07) |
| LAS | SHBG | rs4338849 | 0.83(0.64,1.07) |
| LAS | SHBG | rs434325 | 0.84(0.65,1.08) |
| LAS | SHBG | rs4381470 | 0.84(0.65,1.08) |
| LAS | SHBG | rs445 | 0.84(0.65,1.08) |
| LAS | SHBG | rs45512696 | 0.83(0.64,1.07) |
| LAS | SHBG | rs4568281 | 0.83(0.64,1.07) |
| LAS | SHBG | rs4599176 | 0.83(0.64,1.07) |
| LAS | SHBG | rs4639796 | 0.83(0.64,1.07) |
| LAS | SHBG | rs4665710 | 0.83(0.65,1.08) |
| LAS | SHBG | rs4665972 | 0.85(0.65,1.09) |
| LAS | SHBG | rs4671328 | 0.83(0.64,1.07) |
| LAS | SHBG | rs4674669 | 0.83(0.64,1.07) |
| LAS | SHBG | rs4680 | 0.84(0.65,1.08) |
| LAS | SHBG | rs4709746 | 0.83(0.65,1.08) |
| LAS | SHBG | rs4745876 | 0.83(0.65,1.07) |
| LAS | SHBG | rs4762962 | 0.83(0.65,1.07) |
| LAS | SHBG | rs4794008 | 0.84(0.65,1.08) |
| LAS | SHBG | rs4804414 | 0.84(0.65,1.08) |
| LAS | SHBG | rs4811050 | 0.84(0.65,1.08) |
| LAS | SHBG | rs4812336 | 0.83(0.65,1.07) |
| LAS | SHBG | rs4820091 | 0.82(0.64,1.06) |
| LAS | SHBG | rs483082 | 0.84(0.65,1.08) |
| LAS | SHBG | rs4837794 | 0.82(0.64,1.06) |
| LAS | SHBG | rs4841133 | 0.84(0.65,1.08) |
| LAS | SHBG | rs484943 | 0.83(0.64,1.07) |
| LAS | SHBG | rs4970837 | 0.83(0.64,1.07) |
| LAS | SHBG | rs4974310 | 0.83(0.65,1.07) |
| LAS | SHBG | rs4976033 | 0.83(0.65,1.07) |
| LAS | SHBG | rs5017726 | 0.83(0.65,1.07) |
| LAS | SHBG | rs55737395 | 0.83(0.65,1.07) |
| LAS | SHBG | rs55754498 | 0.83(0.64,1.07) |
| LAS | SHBG | rs55761545 | 0.83(0.64,1.07) |
| LAS | SHBG | rs55771168 | 0.83(0.64,1.07) |
| LAS | SHBG | rs55831924 | 0.83(0.64,1.07) |
| LAS | SHBG | rs55910553 | 0.85(0.66,1.09) |
| LAS | SHBG | rs55974289 | 0.83(0.64,1.07) |
| LAS | SHBG | rs55987409 | 0.83(0.65,1.08) |
| LAS | SHBG | rs559986 | 0.83(0.64,1.07) |
| LAS | SHBG | rs56021343 | 0.83(0.65,1.07) |
| LAS | SHBG | rs56196860 | 0.83(0.65,1.07) |
| LAS | SHBG | rs56292801 | 0.83(0.64,1.07) |
| LAS | SHBG | rs56332871 | 0.81(0.63,1.05) |
| LAS | SHBG | rs56365029 | 0.83(0.65,1.07) |
| LAS | SHBG | rs57158761 | 0.83(0.64,1.06) |
| LAS | SHBG | rs57467915 | 0.84(0.65,1.08) |
| LAS | SHBG | rs575452 | 0.85(0.66,1.09) |
| LAS | SHBG | rs5760120 | 0.83(0.64,1.07) |
| LAS | SHBG | rs57754494 | 0.83(0.64,1.06) |
| LAS | SHBG | rs58321169 | 0.83(0.65,1.08) |
| LAS | SHBG | rs58429317 | 0.83(0.64,1.07) |
| LAS | SHBG | rs591939 | 0.83(0.64,1.07) |
| LAS | SHBG | rs59662471 | 0.83(0.64,1.07) |
| LAS | SHBG | rs59708846 | 0.83(0.64,1.07) |
| LAS | SHBG | rs59708898 | 0.83(0.65,1.08) |
| LAS | SHBG | rs60018147 | 0.83(0.64,1.06) |
| LAS | SHBG | rs6018424 | 0.83(0.64,1.07) |
| LAS | SHBG | rs6048205 | 0.83(0.65,1.08) |
| LAS | SHBG | rs6062381 | 0.83(0.64,1.07) |
| LAS | SHBG | rs6073431 | 0.82(0.64,1.06) |
| LAS | SHBG | rs608300 | 0.83(0.64,1.06) |
| LAS | SHBG | rs6118 | 0.83(0.65,1.07) |
| LAS | SHBG | rs6120663 | 0.83(0.65,1.08) |
| LAS | SHBG | rs61292904 | 0.83(0.65,1.08) |
| LAS | SHBG | rs6129802 | 0.83(0.64,1.07) |
| LAS | SHBG | rs61599759 | 0.83(0.65,1.07) |
| LAS | SHBG | rs61733486 | 0.83(0.64,1.07) |
| LAS | SHBG | rs61759532 | 0.83(0.64,1.07) |
| LAS | SHBG | rs61779331 | 0.83(0.64,1.07) |
| LAS | SHBG | rs61853560 | 0.83(0.64,1.07) |
| LAS | SHBG | rs61854630 | 0.84(0.65,1.08) |
| LAS | SHBG | rs61856602 | 0.84(0.65,1.08) |
| LAS | SHBG | rs619526 | 0.84(0.65,1.08) |
| LAS | SHBG | rs62012946 | 0.83(0.65,1.07) |
| LAS | SHBG | rs62037803 | 0.83(0.64,1.07) |
| LAS | SHBG | rs62111692 | 0.83(0.64,1.07) |
| LAS | SHBG | rs62182125 | 0.83(0.64,1.07) |
| LAS | SHBG | rs62195072 | 0.83(0.65,1.07) |
| LAS | SHBG | rs62334584 | 0.83(0.64,1.07) |
| LAS | SHBG | rs62396733 | 0.83(0.64,1.07) |
| LAS | SHBG | rs62515079 | 0.83(0.64,1.07) |
| LAS | SHBG | rs62565259 | 0.83(0.64,1.07) |
| LAS | SHBG | rs62580766 | 0.83(0.65,1.08) |
| LAS | SHBG | rs62618693 | 0.82(0.64,1.06) |
| LAS | SHBG | rs6422513 | 0.83(0.64,1.07) |
| LAS | SHBG | rs6476065 | 0.83(0.64,1.07) |
| LAS | SHBG | rs6486122 | 0.84(0.65,1.08) |
| LAS | SHBG | rs6495962 | 0.83(0.64,1.07) |
| LAS | SHBG | rs6546096 | 0.84(0.65,1.08) |
| LAS | SHBG | rs6552186 | 0.83(0.65,1.07) |
| LAS | SHBG | rs6556402 | 0.83(0.65,1.07) |
| LAS | SHBG | rs6567160 | 0.83(0.65,1.07) |
| LAS | SHBG | rs6575439 | 0.83(0.64,1.07) |
| LAS | SHBG | rs662026 | 0.83(0.64,1.07) |
| LAS | SHBG | rs6684464 | 0.84(0.65,1.08) |
| LAS | SHBG | rs668871 | 0.84(0.65,1.08) |
| LAS | SHBG | rs66921136 | 0.82(0.64,1.06) |
| LAS | SHBG | rs6710171 | 0.83(0.65,1.07) |
| LAS | SHBG | rs671948 | 0.83(0.64,1.07) |
| LAS | SHBG | rs6736913 | 0.82(0.64,1.06) |
| LAS | SHBG | rs6741180 | 0.83(0.65,1.07) |
| LAS | SHBG | rs6755571 | 0.83(0.65,1.07) |
| LAS | SHBG | rs6791074 | 0.83(0.64,1.07) |
| LAS | SHBG | rs6792725 | 0.83(0.64,1.07) |
| LAS | SHBG | rs6804915 | 0.83(0.65,1.07) |
| LAS | SHBG | rs68062403 | 0.83(0.64,1.07) |
| LAS | SHBG | rs687339 | 0.81(0.63,1.04) |
| LAS | SHBG | rs6910879 | 0.83(0.64,1.07) |
| LAS | SHBG | rs6939861 | 0.84(0.65,1.08) |
| LAS | SHBG | rs695272 | 0.83(0.64,1.07) |
| LAS | SHBG | rs7000496 | 0.83(0.64,1.07) |
| LAS | SHBG | rs700085 | 0.83(0.65,1.07) |
| LAS | SHBG | rs7015 | 0.84(0.65,1.08) |
| LAS | SHBG | rs702876 | 0.83(0.65,1.07) |
| LAS | SHBG | rs7117818 | 0.84(0.65,1.08) |
| LAS | SHBG | rs7123361 | 0.82(0.64,1.06) |
| LAS | SHBG | rs7131509 | 0.83(0.65,1.08) |
| LAS | SHBG | rs715 | 0.84(0.65,1.08) |
| LAS | SHBG | rs7157184 | 0.83(0.64,1.07) |
| LAS | SHBG | rs7164175 | 0.83(0.64,1.07) |
| LAS | SHBG | rs7183456 | 0.83(0.65,1.07) |
| LAS | SHBG | rs720130 | 0.84(0.65,1.08) |
| LAS | SHBG | rs7221716 | 0.83(0.65,1.08) |
| LAS | SHBG | rs7250869 | 0.83(0.65,1.08) |
| LAS | SHBG | rs7262150 | 0.84(0.65,1.08) |
| LAS | SHBG | rs72663937 | 0.83(0.64,1.07) |
| LAS | SHBG | rs72666817 | 0.83(0.64,1.07) |
| LAS | SHBG | rs72683923 | 0.84(0.65,1.08) |
| LAS | SHBG | rs72694845 | 0.83(0.65,1.07) |
| LAS | SHBG | rs72709458 | 0.83(0.64,1.07) |
| LAS | SHBG | rs72753349 | 0.83(0.64,1.07) |
| LAS | SHBG | rs72753908 | 0.83(0.64,1.07) |
| LAS | SHBG | rs72766607 | 0.83(0.64,1.07) |
| LAS | SHBG | rs72767773 | 0.83(0.64,1.07) |
| LAS | SHBG | rs72810505 | 0.83(0.64,1.07) |
| LAS | SHBG | rs72815155 | 0.83(0.64,1.07) |
| LAS | SHBG | rs72840987 | 0.83(0.65,1.07) |
| LAS | SHBG | rs72842808 | 0.84(0.65,1.08) |
| LAS | SHBG | rs72844546 | 0.82(0.64,1.06) |
| LAS | SHBG | rs7298924 | 0.83(0.64,1.07) |
| LAS | SHBG | rs7301634 | 0.83(0.64,1.07) |
| LAS | SHBG | rs73047887 | 0.83(0.64,1.07) |
| LAS | SHBG | rs73079476 | 0.81(0.63,1.04) |
| LAS | SHBG | rs7308634 | 0.84(0.65,1.08) |
| LAS | SHBG | rs7314285 | 0.84(0.65,1.08) |
| LAS | SHBG | rs73193388 | 0.83(0.65,1.08) |
| LAS | SHBG | rs73223295 | 0.83(0.64,1.07) |
| LAS | SHBG | rs7323372 | 0.83(0.64,1.07) |
| LAS | SHBG | rs73375029 | 0.83(0.65,1.07) |
| LAS | SHBG | rs73597479 | 0.83(0.65,1.07) |
| LAS | SHBG | rs736820 | 0.83(0.65,1.08) |
| LAS | SHBG | rs73705826 | 0.84(0.65,1.08) |
| LAS | SHBG | rs73972648 | 0.83(0.64,1.07) |
| LAS | SHBG | rs7406661 | 0.84(0.65,1.08) |
| LAS | SHBG | rs744200 | 0.83(0.64,1.07) |
| LAS | SHBG | rs7451021 | 0.84(0.65,1.08) |
| LAS | SHBG | rs74551598 | 0.83(0.65,1.08) |
| LAS | SHBG | rs74998771 | 0.83(0.64,1.07) |
| LAS | SHBG | rs750155 | 0.83(0.64,1.07) |
| LAS | SHBG | rs75077113 | 0.83(0.65,1.07) |
| LAS | SHBG | rs75156222 | 0.83(0.65,1.08) |
| LAS | SHBG | rs7535528 | 0.83(0.65,1.08) |
| LAS | SHBG | rs7540115 | 0.83(0.64,1.07) |
| LAS | SHBG | rs7552207 | 0.83(0.65,1.07) |
| LAS | SHBG | rs75713100 | 0.83(0.65,1.07) |
| LAS | SHBG | rs757869 | 0.83(0.64,1.07) |
| LAS | SHBG | rs759404 | 0.84(0.65,1.08) |
| LAS | SHBG | rs7617967 | 0.83(0.65,1.08) |
| LAS | SHBG | rs76345703 | 0.83(0.65,1.07) |
| LAS | SHBG | rs7638782 | 0.83(0.65,1.08) |
| LAS | SHBG | rs76475417 | 0.84(0.65,1.08) |
| LAS | SHBG | rs76549335 | 0.84(0.65,1.08) |
| LAS | SHBG | rs7668413 | 0.83(0.64,1.07) |
| LAS | SHBG | rs76708468 | 0.83(0.65,1.07) |
| LAS | SHBG | rs7678138 | 0.83(0.65,1.08) |
| LAS | SHBG | rs76895963 | 0.84(0.65,1.08) |
| LAS | SHBG | rs7696472 | 0.83(0.64,1.07) |
| LAS | SHBG | rs7756992 | 0.83(0.65,1.07) |
| LAS | SHBG | rs77597993 | 0.83(0.65,1.07) |
| LAS | SHBG | rs78058190 | 0.83(0.64,1.07) |
| LAS | SHBG | rs7809920 | 0.83(0.64,1.07) |
| LAS | SHBG | rs7828742 | 0.82(0.64,1.06) |
| LAS | SHBG | rs78319058 | 0.83(0.64,1.07) |
| LAS | SHBG | rs78444298 | 0.84(0.65,1.08) |
| LAS | SHBG | rs78496430 | 0.83(0.64,1.08) |
| LAS | SHBG | rs78536975 | 0.83(0.65,1.08) |
| LAS | SHBG | rs78555071 | 0.84(0.65,1.09) |
| LAS | SHBG | rs7863263 | 0.83(0.65,1.07) |
| LAS | SHBG | rs787976 | 0.83(0.64,1.07) |
| LAS | SHBG | rs78890745 | 0.82(0.64,1.06) |
| LAS | SHBG | rs78973091 | 0.83(0.64,1.07) |
| LAS | SHBG | rs7899096 | 0.83(0.64,1.07) |
| LAS | SHBG | rs7918533 | 0.84(0.65,1.08) |
| LAS | SHBG | rs79287178 | 0.83(0.65,1.08) |
| LAS | SHBG | rs79354983 | 0.83(0.64,1.06) |
| LAS | SHBG | rs7944853 | 0.83(0.64,1.07) |
| LAS | SHBG | rs7953508 | 0.83(0.65,1.07) |
| LAS | SHBG | rs796004 | 0.83(0.64,1.07) |
| LAS | SHBG | rs79600740 | 0.83(0.64,1.07) |
| LAS | SHBG | rs79683734 | 0.83(0.65,1.08) |
| LAS | SHBG | rs79717793 | 0.83(0.65,1.08) |
| LAS | SHBG | rs79875164 | 0.84(0.65,1.08) |
| LAS | SHBG | rs8001781 | 0.83(0.65,1.07) |
| LAS | SHBG | rs80226362 | 0.83(0.65,1.08) |
| LAS | SHBG | rs8031716 | 0.83(0.64,1.07) |
| LAS | SHBG | rs8043101 | 0.83(0.64,1.07) |
| LAS | SHBG | rs8066941 | 0.84(0.65,1.08) |
| LAS | SHBG | rs8069105 | 0.84(0.65,1.08) |
| LAS | SHBG | rs8074363 | 0.83(0.65,1.08) |
| LAS | SHBG | rs8077323 | 0.83(0.65,1.07) |
| LAS | SHBG | rs8079418 | 0.85(0.66,1.09) |
| LAS | SHBG | rs8107967 | 0.83(0.64,1.07) |
| LAS | SHBG | rs8134638 | 0.84(0.65,1.08) |
| LAS | SHBG | rs8176693 | 0.83(0.65,1.08) |
| LAS | SHBG | rs820503 | 0.83(0.64,1.07) |
| LAS | SHBG | rs822508 | 0.83(0.65,1.08) |
| LAS | SHBG | rs841194 | 0.83(0.65,1.07) |
| LAS | SHBG | rs857152 | 0.83(0.64,1.07) |
| LAS | SHBG | rs864899 | 0.83(0.64,1.07) |
| LAS | SHBG | rs885683 | 0.83(0.65,1.07) |
| LAS | SHBG | rs901886 | 0.83(0.65,1.07) |
| LAS | SHBG | rs9297994 | 0.83(0.64,1.07) |
| LAS | SHBG | rs9379084 | 0.83(0.64,1.07) |
| LAS | SHBG | rs9427104 | 0.84(0.65,1.08) |
| LAS | SHBG | rs9439469 | 0.83(0.64,1.07) |
| LAS | SHBG | rs9465601 | 0.83(0.64,1.07) |
| LAS | SHBG | rs9492 | 0.83(0.65,1.07) |
| LAS | SHBG | rs9495298 | 0.83(0.64,1.07) |
| LAS | SHBG | rs9533843 | 0.83(0.64,1.07) |
| LAS | SHBG | rs9556403 | 0.84(0.65,1.08) |
| LAS | SHBG | rs9597811 | 0.83(0.64,1.07) |
| LAS | SHBG | rs9610329 | 0.83(0.65,1.07) |
| LAS | SHBG | rs9614162 | 0.83(0.65,1.07) |
| LAS | SHBG | rs9686661 | 0.84(0.65,1.08) |
| LAS | SHBG | rs9697210 | 0.84(0.65,1.08) |
| LAS | SHBG | rs9823108 | 0.83(0.64,1.07) |
| LAS | SHBG | rs9823118 | 0.83(0.64,1.07) |
| LAS | SHBG | rs9831794 | 0.83(0.65,1.07) |
| LAS | SHBG | rs9871160 | 0.83(0.64,1.07) |
| LAS | SHBG | rs9893194 | 0.83(0.65,1.08) |
| LAS | SHBG | rs9902384 | 0.83(0.64,1.07) |
| LAS | SHBG | rs9968070 | 0.83(0.65,1.07) |
| LAS | SHBG | rs9972653 | 0.84(0.65,1.08) |
| LAS | SHBG | rs998584 | 0.83(0.65,1.07) |
| LAS | SHBG | rs999634 | 0.83(0.64,1.07) |
| CES | SHBG | rs10026753 | 1.02(0.85,1.23) |
| CES | SHBG | rs10041660 | 1.03(0.85,1.24) |
| CES | SHBG | rs1005421 | 1.02(0.85,1.23) |
| CES | SHBG | rs1007851 | 1.03(0.85,1.24) |
| CES | SHBG | rs10084025 | 1.03(0.85,1.24) |
| CES | SHBG | rs10095103 | 1.03(0.85,1.24) |
| CES | SHBG | rs10110651 | 1.03(0.85,1.24) |
| CES | SHBG | rs10111451 | 1.02(0.85,1.24) |
| CES | SHBG | rs10123811 | 1.03(0.85,1.24) |
| CES | SHBG | rs10125995 | 1.03(0.85,1.24) |
| CES | SHBG | rs10153315 | 1.02(0.85,1.23) |
| CES | SHBG | rs10163091 | 1.03(0.85,1.24) |
| CES | SHBG | rs10187560 | 1.03(0.85,1.24) |
| CES | SHBG | rs10208512 | 1.02(0.85,1.24) |
| CES | SHBG | rs10238028 | 1.02(0.85,1.23) |
| CES | SHBG | rs10258433 | 1.02(0.85,1.23) |
| CES | SHBG | rs10273476 | 1.03(0.85,1.24) |
| CES | SHBG | rs10411932 | 1.03(0.85,1.24) |
| CES | SHBG | rs10413329 | 1.02(0.85,1.23) |
| CES | SHBG | rs10416080 | 1.02(0.85,1.23) |
| CES | SHBG | rs1042725 | 1.03(0.85,1.24) |
| CES | SHBG | rs10432029 | 1.05(0.87,1.27) |
| CES | SHBG | rs10492118 | 1.02(0.85,1.24) |
| CES | SHBG | rs10504731 | 1.03(0.85,1.24) |
| CES | SHBG | rs1059698 | 1.02(0.85,1.24) |
| CES | SHBG | rs10733608 | 1.02(0.85,1.23) |
| CES | SHBG | rs10761676 | 1.01(0.84,1.22) |
| CES | SHBG | rs10761749 | 1.02(0.85,1.24) |
| CES | SHBG | rs10773049 | 1.02(0.85,1.24) |
| CES | SHBG | rs10794307 | 1.02(0.85,1.23) |
| CES | SHBG | rs10815276 | 1.03(0.85,1.24) |
| CES | SHBG | rs10822130 | 1.02(0.85,1.24) |
| CES | SHBG | rs10824742 | 1.02(0.85,1.24) |
| CES | SHBG | rs10838681 | 1.02(0.84,1.23) |
| CES | SHBG | rs10864070 | 1.03(0.85,1.24) |
| CES | SHBG | rs10880872 | 1.02(0.85,1.23) |
| CES | SHBG | rs10888696 | 1.03(0.85,1.24) |
| CES | SHBG | rs10895276 | 1.02(0.85,1.24) |
| CES | SHBG | rs10900446 | 1.02(0.85,1.23) |
| CES | SHBG | rs10946313 | 1.02(0.85,1.24) |
| CES | SHBG | rs10951130 | 1.02(0.85,1.24) |
| CES | SHBG | rs10964337 | 1.03(0.85,1.24) |
| CES | SHBG | rs11029441 | 1.03(0.85,1.24) |
| CES | SHBG | rs11030100 | 1.03(0.85,1.24) |
| CES | SHBG | rs11032076 | 1.03(0.85,1.24) |
| CES | SHBG | rs11038673 | 1.02(0.85,1.24) |
| CES | SHBG | rs11078597 | 1.02(0.85,1.23) |
| CES | SHBG | rs11078681 | 1.03(0.85,1.24) |
| CES | SHBG | rs11078701 | 1.04(0.86,1.25) |
| CES | SHBG | rs11079872 | 1.02(0.85,1.23) |
| CES | SHBG | rs11108061 | 1.03(0.85,1.24) |
| CES | SHBG | rs11110390 | 1.02(0.85,1.23) |
| CES | SHBG | rs11111274 | 1.02(0.84,1.23) |
| CES | SHBG | rs11121522 | 1.02(0.85,1.23) |
| CES | SHBG | rs111289824 | 1.02(0.85,1.23) |
| CES | SHBG | rs111331455 | 1.03(0.85,1.24) |
| CES | SHBG | rs111363146 | 1.02(0.85,1.23) |
| CES | SHBG | rs11153046 | 1.02(0.85,1.23) |
| CES | SHBG | rs11155787 | 1.02(0.85,1.23) |
| CES | SHBG | rs111604078 | 1.02(0.85,1.23) |
| CES | SHBG | rs111637026 | 1.03(0.85,1.24) |
| CES | SHBG | rs11164095 | 1.03(0.85,1.24) |
| CES | SHBG | rs11165493 | 1.02(0.85,1.23) |
| CES | SHBG | rs111700120 | 1.03(0.85,1.24) |
| CES | SHBG | rs11187142 | 1.02(0.85,1.24) |
| CES | SHBG | rs11188604 | 1.02(0.85,1.23) |
| CES | SHBG | rs11190245 | 1.03(0.85,1.24) |
| CES | SHBG | rs111905890 | 1.02(0.85,1.24) |
| CES | SHBG | rs11191841 | 1.02(0.85,1.23) |
| CES | SHBG | rs111981233 | 1.02(0.85,1.23) |
| CES | SHBG | rs11202594 | 1.02(0.85,1.23) |
| CES | SHBG | rs112035922 | 1.03(0.85,1.24) |
| CES | SHBG | rs112575738 | 1.03(0.85,1.24) |
| CES | SHBG | rs112672290 | 1.02(0.85,1.24) |
| CES | SHBG | rs112833123 | 1.03(0.85,1.24) |
| CES | SHBG | rs112850234 | 1.03(0.85,1.24) |
| CES | SHBG | rs112928223 | 1.02(0.85,1.23) |
| CES | SHBG | rs113251204 | 1.03(0.85,1.24) |
| CES | SHBG | rs113364399 | 1.03(0.85,1.24) |
| CES | SHBG | rs113523273 | 1.02(0.85,1.24) |
| CES | SHBG | rs113973451 | 1.03(0.85,1.24) |
| CES | SHBG | rs114053844 | 1.03(0.85,1.24) |
| CES | SHBG | rs114469183 | 1.02(0.85,1.23) |
| CES | SHBG | rs114627598 | 1.03(0.85,1.24) |
| CES | SHBG | rs114940462 | 1.02(0.85,1.24) |
| CES | SHBG | rs114949263 | 1.02(0.85,1.23) |
| CES | SHBG | rs11539938 | 1.02(0.85,1.24) |
| CES | SHBG | rs11542663 | 1.03(0.85,1.24) |
| CES | SHBG | rs11545185 | 1.03(0.85,1.24) |
| CES | SHBG | rs115521489 | 1.03(0.85,1.24) |
| CES | SHBG | rs11552708 | 1.00(0.83,1.21) |
| CES | SHBG | rs11556924 | 1.02(0.85,1.23) |
| CES | SHBG | rs11564722 | 1.02(0.85,1.23) |
| CES | SHBG | rs11601507 | 1.03(0.85,1.24) |
| CES | SHBG | rs11621792 | 1.02(0.84,1.23) |
| CES | SHBG | rs11633147 | 1.02(0.85,1.24) |
| CES | SHBG | rs116573491 | 1.02(0.85,1.24) |
| CES | SHBG | rs116713089 | 1.03(0.85,1.24) |
| CES | SHBG | rs11682084 | 1.03(0.85,1.24) |
| CES | SHBG | rs11690176 | 1.03(0.85,1.24) |
| CES | SHBG | rs117108573 | 1.02(0.85,1.24) |
| CES | SHBG | rs117135073 | 1.02(0.85,1.23) |
| CES | SHBG | rs117169274 | 1.03(0.85,1.24) |
| CES | SHBG | rs117411982 | 1.03(0.85,1.24) |
| CES | SHBG | rs11743810 | 1.03(0.85,1.24) |
| CES | SHBG | rs11748938 | 1.03(0.85,1.24) |
| CES | SHBG | rs117589665 | 1.03(0.85,1.24) |
| CES | SHBG | rs11765639 | 1.02(0.85,1.23) |
| CES | SHBG | rs11791747 | 1.02(0.85,1.23) |
| CES | SHBG | rs118080406 | 1.02(0.85,1.24) |
| CES | SHBG | rs11856606 | 1.02(0.85,1.23) |
| CES | SHBG | rs11856926 | 1.01(0.84,1.22) |
| CES | SHBG | rs11994858 | 1.02(0.84,1.23) |
| CES | SHBG | rs11997548 | 1.03(0.85,1.24) |
| CES | SHBG | rs12059956 | 1.02(0.85,1.23) |
| CES | SHBG | rs12138461 | 1.03(0.85,1.24) |
| CES | SHBG | rs12185242 | 1.01(0.84,1.22) |
| CES | SHBG | rs12263369 | 1.03(0.86,1.24) |
| CES | SHBG | rs12280075 | 1.02(0.85,1.23) |
| CES | SHBG | rs1229498 | 1.03(0.85,1.24) |
| CES | SHBG | rs12302952 | 1.02(0.85,1.23) |
| CES | SHBG | rs12311848 | 1.03(0.85,1.24) |
| CES | SHBG | rs12373799 | 1.02(0.85,1.23) |
| CES | SHBG | rs12413488 | 1.02(0.85,1.23) |
| CES | SHBG | rs12424336 | 1.02(0.85,1.24) |
| CES | SHBG | rs12454712 | 1.02(0.84,1.23) |
| CES | SHBG | rs12476661 | 1.03(0.85,1.24) |
| CES | SHBG | rs12575636 | 1.03(0.85,1.24) |
| CES | SHBG | rs12601778 | 1.02(0.85,1.23) |
| CES | SHBG | rs12636106 | 1.02(0.85,1.23) |
| CES | SHBG | rs12667888 | 1.02(0.85,1.23) |
| CES | SHBG | rs12758998 | 1.02(0.85,1.23) |
| CES | SHBG | rs12797706 | 1.03(0.85,1.24) |
| CES | SHBG | rs12809946 | 1.02(0.85,1.24) |
| CES | SHBG | rs12818938 | 1.02(0.85,1.23) |
| CES | SHBG | rs12879423 | 1.03(0.85,1.24) |
| CES | SHBG | rs12916 | 1.03(0.85,1.24) |
| CES | SHBG | rs12926107 | 1.04(0.86,1.25) |
| CES | SHBG | rs12928099 | 1.02(0.84,1.23) |
| CES | SHBG | rs12965052 | 1.02(0.85,1.23) |
| CES | SHBG | rs12989083 | 1.03(0.85,1.24) |
| CES | SHBG | rs13000027 | 1.03(0.85,1.24) |
| CES | SHBG | rs13035806 | 1.03(0.85,1.24) |
| CES | SHBG | rs13057133 | 1.02(0.85,1.23) |
| CES | SHBG | rs13086465 | 1.02(0.85,1.24) |
| CES | SHBG | rs13094241 | 1.03(0.85,1.24) |
| CES | SHBG | rs13108218 | 1.03(0.85,1.25) |
| CES | SHBG | rs13149606 | 1.03(0.85,1.24) |
| CES | SHBG | rs13150068 | 1.03(0.86,1.25) |
| CES | SHBG | rs13251458 | 1.02(0.85,1.23) |
| CES | SHBG | rs1330307 | 1.03(0.85,1.24) |
| CES | SHBG | rs13315174 | 1.02(0.85,1.23) |
| CES | SHBG | rs13379043 | 1.02(0.85,1.23) |
| CES | SHBG | rs13389219 | 1.02(0.85,1.23) |
| CES | SHBG | rs13405815 | 1.02(0.85,1.23) |
| CES | SHBG | rs1352084 | 1.03(0.85,1.24) |
| CES | SHBG | rs138526953 | 1.02(0.85,1.24) |
| CES | SHBG | rs138755456 | 1.03(0.85,1.24) |
| CES | SHBG | rs139805419 | 1.03(0.85,1.24) |
| CES | SHBG | rs139974673 | 1.02(0.85,1.24) |
| CES | SHBG | rs140105410 | 1.02(0.85,1.23) |
| CES | SHBG | rs1408270 | 1.02(0.85,1.23) |
| CES | SHBG | rs1411432 | 1.02(0.85,1.23) |
| CES | SHBG | rs14129 | 1.02(0.85,1.23) |
| CES | SHBG | rs141899843 | 1.02(0.84,1.23) |
| CES | SHBG | rs1420385 | 1.02(0.85,1.23) |
| CES | SHBG | rs142331290 | 1.03(0.86,1.25) |
| CES | SHBG | rs1431659 | 1.03(0.85,1.24) |
| CES | SHBG | rs1433210 | 1.02(0.85,1.23) |
| CES | SHBG | rs143709973 | 1.02(0.85,1.24) |
| CES | SHBG | rs144989856 | 1.02(0.85,1.23) |
| CES | SHBG | rs145931818 | 1.02(0.85,1.23) |
| CES | SHBG | rs147259681 | 1.02(0.85,1.24) |
| CES | SHBG | rs148118632 | 1.02(0.85,1.23) |
| CES | SHBG | rs149092986 | 1.03(0.85,1.24) |
| CES | SHBG | rs149102638 | 1.02(0.85,1.23) |
| CES | SHBG | rs150895955 | 1.03(0.85,1.24) |
| CES | SHBG | rs1530439 | 1.02(0.85,1.24) |
| CES | SHBG | rs1534696 | 1.02(0.85,1.23) |
| CES | SHBG | rs1561442 | 1.03(0.85,1.24) |
| CES | SHBG | rs1570360 | 1.02(0.85,1.23) |
| CES | SHBG | rs157935 | 1.03(0.85,1.24) |
| CES | SHBG | rs1640269 | 1.03(0.85,1.24) |
| CES | SHBG | rs16835135 | 1.02(0.85,1.23) |
| CES | SHBG | rs16934748 | 1.03(0.85,1.24) |
| CES | SHBG | rs17008851 | 1.02(0.85,1.23) |
| CES | SHBG | rs1708302 | 1.02(0.85,1.23) |
| CES | SHBG | rs17184382 | 1.03(0.85,1.24) |
| CES | SHBG | rs17207107 | 1.02(0.85,1.23) |
| CES | SHBG | rs1730862 | 1.03(0.85,1.24) |
| CES | SHBG | rs17356664 | 1.03(0.85,1.24) |
| CES | SHBG | rs17372936 | 1.02(0.85,1.23) |
| CES | SHBG | rs1738380 | 1.02(0.85,1.23) |
| CES | SHBG | rs1743954 | 1.03(0.85,1.24) |
| CES | SHBG | rs174554 | 1.02(0.84,1.23) |
| CES | SHBG | rs17583875 | 1.03(0.85,1.24) |
| CES | SHBG | rs17592998 | 1.03(0.85,1.24) |
| CES | SHBG | rs17669311 | 1.02(0.85,1.24) |
| CES | SHBG | rs1775125 | 1.03(0.85,1.24) |
| CES | SHBG | rs17751614 | 1.03(0.85,1.24) |
| CES | SHBG | rs17755271 | 1.03(0.85,1.24) |
| CES | SHBG | rs17881850 | 1.03(0.85,1.24) |
| CES | SHBG | rs1799831 | 1.03(0.85,1.24) |
| CES | SHBG | rs1801689 | 1.03(0.85,1.24) |
| CES | SHBG | rs182132993 | 1.02(0.85,1.23) |
| CES | SHBG | rs1823227 | 1.02(0.85,1.23) |
| CES | SHBG | rs182848434 | 1.02(0.85,1.23) |
| CES | SHBG | rs183015141 | 1.02(0.85,1.24) |
| CES | SHBG | rs184304 | 1.03(0.85,1.24) |
| CES | SHBG | rs185406435 | 1.02(0.85,1.24) |
| CES | SHBG | rs1864390 | 1.03(0.85,1.24) |
| CES | SHBG | rs186766320 | 1.02(0.85,1.24) |
| CES | SHBG | rs1883783 | 1.02(0.85,1.23) |
| CES | SHBG | rs188889872 | 1.03(0.85,1.24) |
| CES | SHBG | rs188949713 | 1.02(0.85,1.23) |
| CES | SHBG | rs189595752 | 1.02(0.85,1.23) |
| CES | SHBG | rs190712219 | 1.02(0.85,1.24) |
| CES | SHBG | rs1951244 | 1.03(0.85,1.24) |
| CES | SHBG | rs1969213 | 1.02(0.85,1.23) |
| CES | SHBG | rs1993669 | 1.02(0.85,1.23) |
| CES | SHBG | rs201570119 | 1.02(0.85,1.24) |
| CES | SHBG | rs2022865 | 1.02(0.85,1.24) |
| CES | SHBG | rs203777 | 1.02(0.85,1.23) |
| CES | SHBG | rs2063245 | 1.03(0.85,1.24) |
| CES | SHBG | rs2074683 | 1.02(0.85,1.23) |
| CES | SHBG | rs2075915 | 1.03(0.85,1.24) |
| CES | SHBG | rs2121650 | 1.02(0.85,1.23) |
| CES | SHBG | rs2122982 | 1.02(0.85,1.24) |
| CES | SHBG | rs2156804 | 1.02(0.85,1.24) |
| CES | SHBG | rs2176887 | 1.02(0.85,1.23) |
| CES | SHBG | rs2196943 | 1.02(0.85,1.24) |
| CES | SHBG | rs2233364 | 1.02(0.85,1.23) |
| CES | SHBG | rs2234922 | 1.02(0.84,1.23) |
| CES | SHBG | rs2239222 | 1.02(0.85,1.23) |
| CES | SHBG | rs2241261 | 1.03(0.85,1.24) |
| CES | SHBG | rs2246223 | 1.02(0.85,1.24) |
| CES | SHBG | rs2273368 | 1.02(0.85,1.23) |
| CES | SHBG | rs2275355 | 1.03(0.85,1.24) |
| CES | SHBG | rs2277283 | 1.03(0.85,1.24) |
| CES | SHBG | rs2277641 | 1.03(0.85,1.24) |
| CES | SHBG | rs2280838 | 1.02(0.85,1.23) |
| CES | SHBG | rs2304686 | 1.02(0.85,1.24) |
| CES | SHBG | rs2305144 | 1.03(0.85,1.24) |
| CES | SHBG | rs234051 | 1.03(0.85,1.24) |
| CES | SHBG | rs2351958 | 1.03(0.85,1.24) |
| CES | SHBG | rs237438 | 1.02(0.85,1.24) |
| CES | SHBG | rs2393791 | 1.02(0.85,1.24) |
| CES | SHBG | rs2404976 | 1.03(0.85,1.24) |
| CES | SHBG | rs2431752 | 1.02(0.85,1.23) |
| CES | SHBG | rs2450128 | 1.02(0.85,1.23) |
| CES | SHBG | rs2459974 | 1.03(0.86,1.24) |
| CES | SHBG | rs2522054 | 1.02(0.85,1.23) |
| CES | SHBG | rs2535404 | 1.02(0.85,1.23) |
| CES | SHBG | rs2551774 | 1.02(0.85,1.23) |
| CES | SHBG | rs2602856 | 1.02(0.85,1.23) |
| CES | SHBG | rs2618566 | 1.02(0.85,1.23) |
| CES | SHBG | rs2627690 | 1.03(0.85,1.24) |
| CES | SHBG | rs2642438 | 1.03(0.85,1.24) |
| CES | SHBG | rs267733 | 1.02(0.85,1.23) |
| CES | SHBG | rs2705619 | 1.03(0.85,1.24) |
| CES | SHBG | rs2723067 | 1.03(0.85,1.24) |
| CES | SHBG | rs2724475 | 1.03(0.85,1.24) |
| CES | SHBG | rs2746829 | 1.03(0.85,1.24) |
| CES | SHBG | rs2792022 | 1.02(0.85,1.23) |
| CES | SHBG | rs2807861 | 1.03(0.85,1.24) |
| CES | SHBG | rs28507491 | 1.03(0.86,1.25) |
| CES | SHBG | rs28562483 | 1.02(0.85,1.23) |
| CES | SHBG | rs2862954 | 1.02(0.85,1.23) |
| CES | SHBG | rs2885582 | 1.02(0.85,1.23) |
| CES | SHBG | rs28890929 | 1.02(0.85,1.24) |
| CES | SHBG | rs28925904 | 1.03(0.85,1.24) |
| CES | SHBG | rs28929474 | 1.02(0.84,1.23) |
| CES | SHBG | rs2914005 | 1.03(0.85,1.24) |
| CES | SHBG | rs2925979 | 1.03(0.85,1.24) |
| CES | SHBG | rs2942202 | 1.02(0.85,1.23) |
| CES | SHBG | rs2965196 | 1.02(0.85,1.23) |
| CES | SHBG | rs2970877 | 1.02(0.85,1.24) |
| CES | SHBG | rs2972145 | 1.02(0.84,1.23) |
| CES | SHBG | rs2972438 | 1.03(0.85,1.24) |
| CES | SHBG | rs3001032 | 1.02(0.85,1.23) |
| CES | SHBG | rs3004179 | 1.02(0.85,1.24) |
| CES | SHBG | rs3116625 | 1.03(0.85,1.24) |
| CES | SHBG | rs3217860 | 1.02(0.85,1.24) |
| CES | SHBG | rs329122 | 1.02(0.85,1.24) |
| CES | SHBG | rs33807 | 1.03(0.85,1.24) |
| CES | SHBG | rs33999979 | 1.02(0.84,1.23) |
| CES | SHBG | rs34010237 | 1.02(0.85,1.23) |
| CES | SHBG | rs340835 | 1.02(0.85,1.24) |
| CES | SHBG | rs34255979 | 1.02(0.84,1.23) |
| CES | SHBG | rs34311866 | 1.02(0.85,1.24) |
| CES | SHBG | rs34325 | 1.03(0.85,1.24) |
| CES | SHBG | rs34372369 | 1.02(0.85,1.24) |
| CES | SHBG | rs34587839 | 1.03(0.86,1.25) |
| CES | SHBG | rs34651 | 1.03(0.85,1.24) |
| CES | SHBG | rs3468 | 1.03(0.85,1.24) |
| CES | SHBG | rs34707604 | 1.03(0.85,1.24) |
| CES | SHBG | rs34970607 | 1.03(0.85,1.24) |
| CES | SHBG | rs35070405 | 1.02(0.85,1.23) |
| CES | SHBG | rs350832 | 1.03(0.85,1.24) |
| CES | SHBG | rs35102588 | 1.03(0.85,1.24) |
| CES | SHBG | rs35226891 | 1.03(0.85,1.24) |
| CES | SHBG | rs35233014 | 1.03(0.86,1.25) |
| CES | SHBG | rs35333155 | 1.02(0.85,1.23) |
| CES | SHBG | rs35346083 | 1.03(0.85,1.24) |
| CES | SHBG | rs35391516 | 1.03(0.85,1.24) |
| CES | SHBG | rs35547626 | 1.05(0.87,1.27) |
| CES | SHBG | rs35598889 | 1.02(0.85,1.23) |
| CES | SHBG | rs35633876 | 1.02(0.85,1.24) |
| CES | SHBG | rs35812759 | 1.03(0.85,1.24) |
| CES | SHBG | rs359431 | 1.02(0.85,1.24) |
| CES | SHBG | rs35983031 | 1.02(0.85,1.23) |
| CES | SHBG | rs36086195 | 1.02(0.84,1.23) |
| CES | SHBG | rs36124182 | 1.02(0.85,1.24) |
| CES | SHBG | rs362413 | 1.03(0.85,1.24) |
| CES | SHBG | rs368214 | 1.02(0.85,1.23) |
| CES | SHBG | rs3733892 | 1.03(0.85,1.24) |
| CES | SHBG | rs3737178 | 1.03(0.85,1.24) |
| CES | SHBG | rs3741368 | 1.02(0.85,1.23) |
| CES | SHBG | rs3742366 | 1.02(0.85,1.23) |
| CES | SHBG | rs3743588 | 1.03(0.85,1.24) |
| CES | SHBG | rs3761706 | 1.03(0.85,1.24) |
| CES | SHBG | rs3764002 | 1.02(0.85,1.24) |
| CES | SHBG | rs3770781 | 1.03(0.85,1.24) |
| CES | SHBG | rs3780190 | 1.02(0.84,1.23) |
| CES | SHBG | rs3782735 | 1.02(0.85,1.23) |
| CES | SHBG | rs3813498 | 1.02(0.85,1.23) |
| CES | SHBG | rs3829639 | 1.03(0.85,1.24) |
| CES | SHBG | rs38304 | 1.02(0.85,1.23) |
| CES | SHBG | rs3848119 | 1.02(0.85,1.23) |
| CES | SHBG | rs3848125 | 1.02(0.85,1.23) |
| CES | SHBG | rs38855 | 1.03(0.85,1.24) |
| CES | SHBG | rs40270 | 1.02(0.85,1.24) |
| CES | SHBG | rs403694 | 1.02(0.85,1.24) |
| CES | SHBG | rs4073358 | 1.03(0.85,1.24) |
| CES | SHBG | rs4092465 | 1.02(0.85,1.24) |
| CES | SHBG | rs41280463 | 1.03(0.85,1.24) |
| CES | SHBG | rs41309159 | 1.03(0.85,1.24) |
| CES | SHBG | rs4135240 | 1.03(0.85,1.24) |
| CES | SHBG | rs4147563 | 1.03(0.85,1.24) |
| CES | SHBG | rs4147913 | 1.03(0.85,1.24) |
| CES | SHBG | rs42238 | 1.03(0.85,1.24) |
| CES | SHBG | rs4274814 | 1.02(0.85,1.24) |
| CES | SHBG | rs4297769 | 1.02(0.85,1.23) |
| CES | SHBG | rs4300303 | 1.02(0.85,1.23) |
| CES | SHBG | rs4309185 | 1.03(0.85,1.24) |
| CES | SHBG | rs4338849 | 1.03(0.85,1.24) |
| CES | SHBG | rs434325 | 1.03(0.85,1.24) |
| CES | SHBG | rs4381470 | 1.02(0.85,1.24) |
| CES | SHBG | rs445 | 1.03(0.85,1.24) |
| CES | SHBG | rs45512696 | 1.03(0.85,1.24) |
| CES | SHBG | rs4568281 | 1.03(0.85,1.24) |
| CES | SHBG | rs4599176 | 1.03(0.85,1.24) |
| CES | SHBG | rs4639796 | 1.03(0.85,1.24) |
| CES | SHBG | rs4665710 | 1.02(0.85,1.24) |
| CES | SHBG | rs4665972 | 1.02(0.84,1.23) |
| CES | SHBG | rs4671328 | 1.02(0.85,1.23) |
| CES | SHBG | rs4674669 | 1.02(0.85,1.24) |
| CES | SHBG | rs4680 | 1.02(0.85,1.23) |
| CES | SHBG | rs4709746 | 1.02(0.85,1.23) |
| CES | SHBG | rs4745876 | 1.02(0.84,1.23) |
| CES | SHBG | rs4762962 | 1.02(0.85,1.23) |
| CES | SHBG | rs4794008 | 1.03(0.85,1.24) |
| CES | SHBG | rs4804414 | 1.02(0.84,1.23) |
| CES | SHBG | rs4811050 | 1.03(0.85,1.24) |
| CES | SHBG | rs4812336 | 1.03(0.85,1.24) |
| CES | SHBG | rs4820091 | 1.02(0.84,1.23) |
| CES | SHBG | rs483082 | 1.03(0.85,1.24) |
| CES | SHBG | rs4837794 | 1.03(0.85,1.24) |
| CES | SHBG | rs4841133 | 1.03(0.85,1.24) |
| CES | SHBG | rs484943 | 1.03(0.85,1.24) |
| CES | SHBG | rs4970837 | 1.03(0.85,1.24) |
| CES | SHBG | rs4974310 | 1.02(0.85,1.24) |
| CES | SHBG | rs4976033 | 1.02(0.85,1.24) |
| CES | SHBG | rs5017726 | 1.02(0.85,1.23) |
| CES | SHBG | rs55737395 | 1.02(0.85,1.23) |
| CES | SHBG | rs55754498 | 1.02(0.85,1.23) |
| CES | SHBG | rs55761545 | 1.02(0.85,1.23) |
| CES | SHBG | rs55771168 | 1.02(0.84,1.23) |
| CES | SHBG | rs55831924 | 1.02(0.85,1.24) |
| CES | SHBG | rs55910553 | 1.03(0.86,1.25) |
| CES | SHBG | rs55974289 | 1.02(0.85,1.23) |
| CES | SHBG | rs55987409 | 1.02(0.84,1.23) |
| CES | SHBG | rs559986 | 1.02(0.85,1.24) |
| CES | SHBG | rs56021343 | 1.03(0.85,1.24) |
| CES | SHBG | rs56196860 | 1.02(0.85,1.23) |
| CES | SHBG | rs56292801 | 1.02(0.85,1.24) |
| CES | SHBG | rs56332871 | 1.01(0.83,1.22) |
| CES | SHBG | rs56365029 | 1.03(0.85,1.24) |
| CES | SHBG | rs57158761 | 1.02(0.85,1.23) |
| CES | SHBG | rs57467915 | 1.03(0.85,1.24) |
| CES | SHBG | rs575452 | 1.02(0.85,1.23) |
| CES | SHBG | rs5760120 | 1.03(0.85,1.24) |
| CES | SHBG | rs57754494 | 1.02(0.84,1.23) |
| CES | SHBG | rs58321169 | 1.03(0.85,1.24) |
| CES | SHBG | rs591939 | 1.03(0.85,1.24) |
| CES | SHBG | rs59662471 | 1.02(0.85,1.23) |
| CES | SHBG | rs59708846 | 1.02(0.85,1.24) |
| CES | SHBG | rs59708898 | 1.03(0.85,1.24) |
| CES | SHBG | rs60018147 | 1.03(0.85,1.24) |
| CES | SHBG | rs6018424 | 1.02(0.85,1.23) |
| CES | SHBG | rs6048205 | 1.03(0.85,1.24) |
| CES | SHBG | rs6062381 | 1.02(0.85,1.23) |
| CES | SHBG | rs6073431 | 1.01(0.84,1.22) |
| CES | SHBG | rs608300 | 1.02(0.84,1.23) |
| CES | SHBG | rs6118 | 1.03(0.85,1.24) |
| CES | SHBG | rs6120663 | 1.03(0.85,1.24) |
| CES | SHBG | rs61292904 | 1.03(0.85,1.24) |
| CES | SHBG | rs6129802 | 1.03(0.85,1.24) |
| CES | SHBG | rs61599759 | 1.03(0.85,1.24) |
| CES | SHBG | rs61733486 | 1.02(0.85,1.23) |
| CES | SHBG | rs61759532 | 1.03(0.85,1.24) |
| CES | SHBG | rs61779331 | 1.02(0.85,1.23) |
| CES | SHBG | rs61853560 | 1.02(0.84,1.23) |
| CES | SHBG | rs61854630 | 1.02(0.85,1.23) |
| CES | SHBG | rs61856602 | 1.03(0.85,1.24) |
| CES | SHBG | rs619526 | 1.02(0.85,1.24) |
| CES | SHBG | rs62012946 | 1.03(0.85,1.24) |
| CES | SHBG | rs62037803 | 1.02(0.85,1.24) |
| CES | SHBG | rs62111692 | 1.02(0.85,1.23) |
| CES | SHBG | rs62182125 | 1.02(0.85,1.23) |
| CES | SHBG | rs62195072 | 1.02(0.85,1.23) |
| CES | SHBG | rs62334584 | 1.03(0.85,1.24) |
| CES | SHBG | rs62396733 | 1.03(0.85,1.24) |
| CES | SHBG | rs62515079 | 1.02(0.85,1.23) |
| CES | SHBG | rs62565259 | 1.02(0.85,1.24) |
| CES | SHBG | rs62580766 | 1.03(0.85,1.24) |
| CES | SHBG | rs62618693 | 1.03(0.85,1.24) |
| CES | SHBG | rs6422513 | 1.03(0.85,1.24) |
| CES | SHBG | rs6476065 | 1.02(0.85,1.23) |
| CES | SHBG | rs6486122 | 1.03(0.86,1.25) |
| CES | SHBG | rs6495962 | 1.03(0.85,1.24) |
| CES | SHBG | rs6546096 | 1.03(0.85,1.24) |
| CES | SHBG | rs6552186 | 1.03(0.85,1.24) |
| CES | SHBG | rs6556402 | 1.03(0.85,1.24) |
| CES | SHBG | rs6567160 | 1.03(0.85,1.24) |
| CES | SHBG | rs6575439 | 1.03(0.85,1.24) |
| CES | SHBG | rs662026 | 1.02(0.85,1.24) |
| CES | SHBG | rs6684464 | 1.02(0.85,1.24) |
| CES | SHBG | rs668871 | 1.02(0.84,1.23) |
| CES | SHBG | rs66921136 | 1.03(0.85,1.24) |
| CES | SHBG | rs6710171 | 1.02(0.85,1.23) |
| CES | SHBG | rs671948 | 1.02(0.85,1.23) |
| CES | SHBG | rs6736913 | 1.02(0.85,1.23) |
| CES | SHBG | rs6741180 | 1.02(0.85,1.23) |
| CES | SHBG | rs6755571 | 1.02(0.85,1.23) |
| CES | SHBG | rs6791074 | 1.02(0.85,1.23) |
| CES | SHBG | rs6792725 | 1.02(0.85,1.24) |
| CES | SHBG | rs6804915 | 1.03(0.85,1.24) |
| CES | SHBG | rs68062403 | 1.02(0.85,1.23) |
| CES | SHBG | rs687339 | 1.02(0.84,1.23) |
| CES | SHBG | rs6910879 | 1.02(0.85,1.24) |
| CES | SHBG | rs6939861 | 1.03(0.85,1.24) |
| CES | SHBG | rs695272 | 1.03(0.85,1.24) |
| CES | SHBG | rs7000496 | 1.02(0.85,1.24) |
| CES | SHBG | rs700085 | 1.03(0.85,1.24) |
| CES | SHBG | rs7015 | 1.05(0.87,1.26) |
| CES | SHBG | rs702876 | 1.02(0.85,1.23) |
| CES | SHBG | rs7117818 | 1.02(0.85,1.23) |
| CES | SHBG | rs7123361 | 1.02(0.85,1.24) |
| CES | SHBG | rs7131509 | 1.03(0.85,1.24) |
| CES | SHBG | rs715 | 1.02(0.85,1.24) |
| CES | SHBG | rs7157184 | 1.03(0.85,1.24) |
| CES | SHBG | rs7164175 | 1.02(0.85,1.24) |
| CES | SHBG | rs7183456 | 1.02(0.85,1.24) |
| CES | SHBG | rs720130 | 1.02(0.85,1.23) |
| CES | SHBG | rs7221716 | 1.02(0.85,1.23) |
| CES | SHBG | rs7250869 | 1.03(0.85,1.24) |
| CES | SHBG | rs7262150 | 1.03(0.85,1.24) |
| CES | SHBG | rs72663937 | 1.02(0.85,1.23) |
| CES | SHBG | rs72666817 | 1.02(0.85,1.23) |
| CES | SHBG | rs72683923 | 1.02(0.84,1.23) |
| CES | SHBG | rs72694845 | 1.03(0.85,1.24) |
| CES | SHBG | rs72709458 | 1.03(0.85,1.24) |
| CES | SHBG | rs72753349 | 1.03(0.85,1.24) |
| CES | SHBG | rs72753908 | 1.02(0.85,1.23) |
| CES | SHBG | rs72766607 | 1.03(0.85,1.24) |
| CES | SHBG | rs72767773 | 1.03(0.85,1.24) |
| CES | SHBG | rs72810505 | 1.03(0.85,1.24) |
| CES | SHBG | rs72815155 | 1.02(0.85,1.24) |
| CES | SHBG | rs72840987 | 1.02(0.85,1.23) |
| CES | SHBG | rs72842808 | 1.03(0.86,1.25) |
| CES | SHBG | rs72844546 | 1.02(0.85,1.23) |
| CES | SHBG | rs7298924 | 1.03(0.85,1.24) |
| CES | SHBG | rs7301634 | 1.02(0.85,1.23) |
| CES | SHBG | rs73047887 | 1.03(0.85,1.24) |
| CES | SHBG | rs73079476 | 1.01(0.84,1.22) |
| CES | SHBG | rs7308634 | 1.02(0.85,1.23) |
| CES | SHBG | rs7314285 | 1.03(0.85,1.24) |
| CES | SHBG | rs73193388 | 1.02(0.85,1.24) |
| CES | SHBG | rs73223295 | 1.02(0.85,1.23) |
| CES | SHBG | rs7323372 | 1.02(0.85,1.23) |
| CES | SHBG | rs73375029 | 1.02(0.85,1.24) |
| CES | SHBG | rs73597479 | 1.03(0.85,1.24) |
| CES | SHBG | rs736820 | 1.02(0.85,1.24) |
| CES | SHBG | rs73705826 | 1.02(0.85,1.24) |
| CES | SHBG | rs73972648 | 1.02(0.85,1.23) |
| CES | SHBG | rs7406661 | 1.01(0.84,1.22) |
| CES | SHBG | rs744200 | 1.03(0.85,1.24) |
| CES | SHBG | rs7451021 | 1.02(0.85,1.24) |
| CES | SHBG | rs74551598 | 1.02(0.85,1.24) |
| CES | SHBG | rs74998771 | 1.02(0.85,1.24) |
| CES | SHBG | rs750155 | 1.02(0.85,1.24) |
| CES | SHBG | rs75077113 | 1.03(0.85,1.24) |
| CES | SHBG | rs75156222 | 1.02(0.85,1.23) |
| CES | SHBG | rs7535528 | 1.02(0.85,1.24) |
| CES | SHBG | rs7540115 | 1.02(0.85,1.23) |
| CES | SHBG | rs7552207 | 1.02(0.85,1.24) |
| CES | SHBG | rs75713100 | 1.02(0.85,1.23) |
| CES | SHBG | rs757869 | 1.03(0.86,1.24) |
| CES | SHBG | rs759404 | 1.02(0.85,1.24) |
| CES | SHBG | rs7617967 | 1.02(0.85,1.24) |
| CES | SHBG | rs76345703 | 1.03(0.85,1.24) |
| CES | SHBG | rs7638782 | 1.02(0.85,1.23) |
| CES | SHBG | rs76475417 | 1.02(0.85,1.24) |
| CES | SHBG | rs76549335 | 1.02(0.85,1.24) |
| CES | SHBG | rs7668413 | 1.02(0.85,1.23) |
| CES | SHBG | rs76708468 | 1.02(0.85,1.24) |
| CES | SHBG | rs7678138 | 1.03(0.85,1.24) |
| CES | SHBG | rs76895963 | 1.03(0.85,1.24) |
| CES | SHBG | rs7696472 | 1.02(0.85,1.24) |
| CES | SHBG | rs7756992 | 1.02(0.85,1.24) |
| CES | SHBG | rs77597993 | 1.03(0.85,1.24) |
| CES | SHBG | rs78058190 | 1.02(0.85,1.23) |
| CES | SHBG | rs7809920 | 1.03(0.85,1.24) |
| CES | SHBG | rs7828742 | 1.02(0.85,1.24) |
| CES | SHBG | rs78319058 | 1.02(0.85,1.23) |
| CES | SHBG | rs78444298 | 1.03(0.85,1.24) |
| CES | SHBG | rs78496430 | 1.04(0.86,1.26) |
| CES | SHBG | rs78536975 | 1.03(0.85,1.24) |
| CES | SHBG | rs78555071 | 1.02(0.85,1.24) |
| CES | SHBG | rs7863263 | 1.03(0.85,1.24) |
| CES | SHBG | rs787976 | 1.02(0.85,1.23) |
| CES | SHBG | rs78890745 | 1.02(0.85,1.24) |
| CES | SHBG | rs78973091 | 1.02(0.85,1.24) |
| CES | SHBG | rs7899096 | 1.02(0.85,1.24) |
| CES | SHBG | rs7918533 | 1.02(0.85,1.23) |
| CES | SHBG | rs79287178 | 1.02(0.85,1.24) |
| CES | SHBG | rs79354983 | 1.02(0.85,1.24) |
| CES | SHBG | rs79391862 | 1.03(0.85,1.24) |
| CES | SHBG | rs7944853 | 1.02(0.85,1.23) |
| CES | SHBG | rs7953508 | 1.02(0.85,1.23) |
| CES | SHBG | rs796004 | 1.01(0.83,1.21) |
| CES | SHBG | rs79600740 | 1.03(0.85,1.24) |
| CES | SHBG | rs79683734 | 1.03(0.85,1.24) |
| CES | SHBG | rs79717793 | 1.02(0.84,1.23) |
| CES | SHBG | rs79875164 | 1.03(0.85,1.24) |
| CES | SHBG | rs8001781 | 1.03(0.85,1.24) |
| CES | SHBG | rs80226362 | 1.02(0.85,1.23) |
| CES | SHBG | rs8031716 | 1.03(0.85,1.24) |
| CES | SHBG | rs8043101 | 1.02(0.85,1.23) |
| CES | SHBG | rs8066941 | 1.02(0.85,1.24) |
| CES | SHBG | rs8069105 | 1.03(0.85,1.24) |
| CES | SHBG | rs8074363 | 1.03(0.85,1.24) |
| CES | SHBG | rs8077323 | 1.03(0.85,1.24) |
| CES | SHBG | rs8079418 | 1.03(0.86,1.25) |
| CES | SHBG | rs8107967 | 1.02(0.85,1.24) |
| CES | SHBG | rs8134638 | 1.02(0.84,1.23) |
| CES | SHBG | rs8176693 | 1.03(0.85,1.24) |
| CES | SHBG | rs820503 | 1.03(0.85,1.24) |
| CES | SHBG | rs822508 | 1.02(0.85,1.23) |
| CES | SHBG | rs841194 | 1.03(0.85,1.24) |
| CES | SHBG | rs857152 | 1.02(0.85,1.24) |
| CES | SHBG | rs864899 | 1.02(0.85,1.23) |
| CES | SHBG | rs885683 | 1.03(0.85,1.24) |
| CES | SHBG | rs901886 | 1.02(0.85,1.23) |
| CES | SHBG | rs9297994 | 1.02(0.85,1.23) |
| CES | SHBG | rs9379084 | 1.03(0.85,1.24) |
| CES | SHBG | rs9427104 | 1.02(0.84,1.23) |
| CES | SHBG | rs9439469 | 1.02(0.85,1.23) |
| CES | SHBG | rs9465601 | 1.02(0.85,1.23) |
| CES | SHBG | rs9492 | 1.03(0.85,1.24) |
| CES | SHBG | rs9495298 | 1.02(0.85,1.23) |
| CES | SHBG | rs9533843 | 1.02(0.85,1.24) |
| CES | SHBG | rs9556403 | 1.03(0.85,1.24) |
| CES | SHBG | rs9597811 | 1.03(0.85,1.24) |
| CES | SHBG | rs9610329 | 1.02(0.85,1.23) |
| CES | SHBG | rs9614162 | 1.02(0.85,1.24) |
| CES | SHBG | rs9686661 | 1.03(0.85,1.24) |
| CES | SHBG | rs9697210 | 1.02(0.85,1.23) |
| CES | SHBG | rs9823108 | 1.02(0.85,1.23) |
| CES | SHBG | rs9823118 | 1.02(0.85,1.24) |
| CES | SHBG | rs9831794 | 1.02(0.85,1.24) |
| CES | SHBG | rs9871160 | 1.02(0.84,1.22) |
| CES | SHBG | rs9893194 | 1.02(0.85,1.24) |
| CES | SHBG | rs9902384 | 1.03(0.85,1.24) |
| CES | SHBG | rs9968070 | 1.02(0.85,1.23) |
| CES | SHBG | rs9972653 | 1.03(0.85,1.24) |
| CES | SHBG | rs998584 | 1.02(0.85,1.23) |
| CES | SHBG | rs999634 | 1.03(0.85,1.24) |
| SVS | SHBG | rs1005421 | 1.03(0.74,1.45) |
| SVS | SHBG | rs1007851 | 1.05(0.75,1.47) |
| SVS | SHBG | rs10084025 | 1.04(0.74,1.46) |
| SVS | SHBG | rs10153315 | 1.06(0.76,1.48) |
| SVS | SHBG | rs10163091 | 1.04(0.74,1.45) |
| SVS | SHBG | rs10411932 | 1.05(0.75,1.46) |
| SVS | SHBG | rs10413329 | 1.04(0.74,1.46) |
| SVS | SHBG | rs10416080 | 1.04(0.75,1.46) |
| SVS | SHBG | rs10432029 | 1.11(0.78,1.57) |
| SVS | SHBG | rs11078597 | 1.04(0.74,1.45) |
| SVS | SHBG | rs11078681 | 1.04(0.74,1.45) |
| SVS | SHBG | rs11078701 | 1.06(0.75,1.49) |
| SVS | SHBG | rs11079872 | 1.05(0.75,1.47) |
| SVS | SHBG | rs111604078 | 1.05(0.75,1.47) |
| SVS | SHBG | rs111700120 | 1.04(0.75,1.46) |
| SVS | SHBG | rs111981233 | 1.03(0.74,1.44) |
| SVS | SHBG | rs112035922 | 1.05(0.75,1.47) |
| SVS | SHBG | rs113251204 | 1.03(0.74,1.44) |
| SVS | SHBG | rs113364399 | 1.05(0.75,1.46) |
| SVS | SHBG | rs113523273 | 1.05(0.75,1.47) |
| SVS | SHBG | rs11539938 | 1.03(0.74,1.44) |
| SVS | SHBG | rs11545185 | 1.04(0.74,1.46) |
| SVS | SHBG | rs11552708 | 1.05(0.74,1.47) |
| SVS | SHBG | rs11621792 | 1.07(0.76,1.50) |
| SVS | SHBG | rs11633147 | 1.04(0.74,1.46) |
| SVS | SHBG | rs116573491 | 1.04(0.74,1.46) |
| SVS | SHBG | rs117169274 | 1.05(0.75,1.46) |
| SVS | SHBG | rs117411982 | 1.05(0.75,1.46) |
| SVS | SHBG | rs11856606 | 1.04(0.74,1.46) |
| SVS | SHBG | rs11856926 | 1.05(0.75,1.47) |
| SVS | SHBG | rs12185242 | 1.11(0.79,1.56) |
| SVS | SHBG | rs12454712 | 1.03(0.74,1.44) |
| SVS | SHBG | rs12601778 | 1.03(0.74,1.43) |
| SVS | SHBG | rs12879423 | 1.02(0.73,1.43) |
| SVS | SHBG | rs12926107 | 1.06(0.76,1.48) |
| SVS | SHBG | rs12928099 | 1.04(0.74,1.45) |
| SVS | SHBG | rs12965052 | 1.03(0.74,1.44) |
| SVS | SHBG | rs13057133 | 1.04(0.74,1.45) |
| SVS | SHBG | rs13379043 | 1.04(0.74,1.45) |
| SVS | SHBG | rs139805419 | 1.04(0.74,1.45) |
| SVS | SHBG | rs139974673 | 1.10(0.79,1.54) |
| SVS | SHBG | rs140105410 | 1.03(0.74,1.45) |
| SVS | SHBG | rs14129 | 1.02(0.73,1.42) |
| SVS | SHBG | rs142331290 | 1.03(0.73,1.44) |
| SVS | SHBG | rs144989856 | 1.05(0.75,1.47) |
| SVS | SHBG | rs147259681 | 1.05(0.75,1.47) |
| SVS | SHBG | rs150895955 | 1.04(0.74,1.46) |
| SVS | SHBG | rs1640269 | 1.09(0.78,1.53) |
| SVS | SHBG | rs17184382 | 1.03(0.74,1.45) |
| SVS | SHBG | rs17207107 | 1.04(0.75,1.46) |
| SVS | SHBG | rs17356664 | 1.04(0.74,1.45) |
| SVS | SHBG | rs17669311 | 1.03(0.74,1.44) |
| SVS | SHBG | rs17751614 | 1.04(0.74,1.45) |
| SVS | SHBG | rs17881850 | 1.04(0.75,1.46) |
| SVS | SHBG | rs1801689 | 1.05(0.75,1.47) |
| SVS | SHBG | rs188889872 | 1.05(0.75,1.48) |
| SVS | SHBG | rs1951244 | 1.05(0.75,1.47) |
| SVS | SHBG | rs2075915 | 1.03(0.74,1.45) |
| SVS | SHBG | rs2233364 | 1.05(0.75,1.47) |
| SVS | SHBG | rs2239222 | 1.04(0.74,1.46) |
| SVS | SHBG | rs2277641 | 1.05(0.75,1.47) |
| SVS | SHBG | rs2618566 | 1.04(0.74,1.45) |
| SVS | SHBG | rs2746829 | 1.05(0.75,1.47) |
| SVS | SHBG | rs28562483 | 1.04(0.74,1.45) |
| SVS | SHBG | rs28929474 | 0.97(0.69,1.35) |
| SVS | SHBG | rs2914005 | 1.05(0.75,1.47) |
| SVS | SHBG | rs2925979 | 1.04(0.74,1.46) |
| SVS | SHBG | rs2965196 | 1.04(0.74,1.46) |
| SVS | SHBG | rs2972438 | 1.04(0.75,1.46) |
| SVS | SHBG | rs3116625 | 1.05(0.75,1.47) |
| SVS | SHBG | rs34010237 | 1.05(0.75,1.47) |
| SVS | SHBG | rs34255979 | 1.04(0.74,1.46) |
| SVS | SHBG | rs34587839 | 1.06(0.76,1.48) |
| SVS | SHBG | rs350832 | 1.04(0.75,1.46) |
| SVS | SHBG | rs35102588 | 1.05(0.75,1.47) |
| SVS | SHBG | rs35391516 | 1.04(0.75,1.46) |
| SVS | SHBG | rs35547626 | 1.03(0.73,1.45) |
| SVS | SHBG | rs35598889 | 1.04(0.74,1.46) |
| SVS | SHBG | rs35812759 | 1.05(0.75,1.48) |
| SVS | SHBG | rs362413 | 1.04(0.74,1.46) |
| SVS | SHBG | rs3742366 | 1.03(0.74,1.43) |
| SVS | SHBG | rs3743588 | 1.04(0.74,1.45) |
| SVS | SHBG | rs3829639 | 1.05(0.75,1.47) |
| SVS | SHBG | rs3848119 | 1.03(0.74,1.44) |
| SVS | SHBG | rs3848125 | 1.04(0.74,1.45) |
| SVS | SHBG | rs403694 | 1.04(0.74,1.45) |
| SVS | SHBG | rs4092465 | 1.04(0.74,1.46) |
| SVS | SHBG | rs4147913 | 1.04(0.74,1.45) |
| SVS | SHBG | rs4297769 | 1.04(0.74,1.45) |
| SVS | SHBG | rs4338849 | 1.05(0.75,1.47) |
| SVS | SHBG | rs434325 | 1.04(0.74,1.46) |
| SVS | SHBG | rs4381470 | 1.05(0.75,1.47) |
| SVS | SHBG | rs45512696 | 1.03(0.73,1.44) |
| SVS | SHBG | rs4599176 | 1.03(0.74,1.45) |
| SVS | SHBG | rs4680 | 1.04(0.74,1.45) |
| SVS | SHBG | rs4794008 | 1.05(0.75,1.47) |
| SVS | SHBG | rs4804414 | 1.04(0.74,1.46) |
| SVS | SHBG | rs4811050 | 1.04(0.74,1.46) |
| SVS | SHBG | rs4812336 | 1.05(0.75,1.46) |
| SVS | SHBG | rs4820091 | 1.03(0.73,1.44) |
| SVS | SHBG | rs483082 | 1.05(0.75,1.47) |
| SVS | SHBG | rs484943 | 1.06(0.76,1.47) |
| SVS | SHBG | rs5017726 | 1.03(0.74,1.43) |
| SVS | SHBG | rs55737395 | 1.04(0.74,1.45) |
| SVS | SHBG | rs55754498 | 1.04(0.74,1.45) |
| SVS | SHBG | rs55910553 | 1.01(0.72,1.41) |
| SVS | SHBG | rs55987409 | 1.03(0.74,1.45) |
| SVS | SHBG | rs56292801 | 1.04(0.74,1.46) |
| SVS | SHBG | rs56332871 | 1.05(0.75,1.49) |
| SVS | SHBG | rs5760120 | 1.05(0.75,1.47) |
| SVS | SHBG | rs591939 | 1.04(0.74,1.46) |
| SVS | SHBG | rs59708898 | 1.04(0.74,1.45) |
| SVS | SHBG | rs60018147 | 1.04(0.74,1.45) |
| SVS | SHBG | rs6018424 | 1.05(0.76,1.47) |
| SVS | SHBG | rs6048205 | 1.03(0.74,1.44) |
| SVS | SHBG | rs6062381 | 1.05(0.75,1.47) |
| SVS | SHBG | rs6073431 | 1.05(0.75,1.48) |
| SVS | SHBG | rs6118 | 1.04(0.74,1.46) |
| SVS | SHBG | rs6120663 | 1.04(0.74,1.45) |
| SVS | SHBG | rs6129802 | 1.05(0.75,1.47) |
| SVS | SHBG | rs61599759 | 1.04(0.74,1.45) |
| SVS | SHBG | rs61733486 | 1.04(0.75,1.46) |
| SVS | SHBG | rs61759532 | 1.05(0.75,1.47) |
| SVS | SHBG | rs619526 | 1.04(0.74,1.46) |
| SVS | SHBG | rs62012946 | 1.05(0.75,1.46) |
| SVS | SHBG | rs62037803 | 1.04(0.74,1.46) |
| SVS | SHBG | rs62111692 | 1.04(0.74,1.46) |
| SVS | SHBG | rs6495962 | 1.04(0.75,1.46) |
| SVS | SHBG | rs6567160 | 1.03(0.74,1.44) |
| SVS | SHBG | rs6575439 | 1.04(0.74,1.46) |
| SVS | SHBG | rs66921136 | 1.03(0.74,1.45) |
| SVS | SHBG | rs671948 | 1.05(0.75,1.48) |
| SVS | SHBG | rs68062403 | 1.03(0.74,1.44) |
| SVS | SHBG | rs695272 | 1.04(0.74,1.45) |
| SVS | SHBG | rs7157184 | 1.04(0.74,1.45) |
| SVS | SHBG | rs7164175 | 1.04(0.75,1.46) |
| SVS | SHBG | rs7183456 | 1.03(0.74,1.44) |
| SVS | SHBG | rs720130 | 1.05(0.75,1.47) |
| SVS | SHBG | rs7221716 | 1.05(0.75,1.47) |
| SVS | SHBG | rs7250869 | 1.05(0.75,1.47) |
| SVS | SHBG | rs7262150 | 1.04(0.75,1.46) |
| SVS | SHBG | rs72683923 | 1.03(0.74,1.45) |
| SVS | SHBG | rs72753908 | 1.04(0.74,1.45) |
| SVS | SHBG | rs72767773 | 1.02(0.73,1.42) |
| SVS | SHBG | rs72840987 | 1.04(0.74,1.46) |
| SVS | SHBG | rs72842808 | 1.04(0.74,1.46) |
| SVS | SHBG | rs72844546 | 1.03(0.74,1.44) |
| SVS | SHBG | rs7323372 | 1.05(0.75,1.47) |
| SVS | SHBG | rs73597479 | 1.04(0.74,1.46) |
| SVS | SHBG | rs736820 | 1.03(0.74,1.45) |
| SVS | SHBG | rs73972648 | 1.04(0.74,1.45) |
| SVS | SHBG | rs7406661 | 1.06(0.75,1.48) |
| SVS | SHBG | rs744200 | 1.04(0.74,1.46) |
| SVS | SHBG | rs74998771 | 1.04(0.74,1.45) |
| SVS | SHBG | rs750155 | 1.05(0.75,1.46) |
| SVS | SHBG | rs757869 | 1.06(0.76,1.48) |
| SVS | SHBG | rs759404 | 1.04(0.74,1.46) |
| SVS | SHBG | rs76549335 | 1.04(0.74,1.46) |
| SVS | SHBG | rs76708468 | 1.04(0.74,1.46) |
| SVS | SHBG | rs78319058 | 1.04(0.74,1.45) |
| SVS | SHBG | rs78496430 | 1.03(0.73,1.45) |
| SVS | SHBG | rs78555071 | 1.04(0.73,1.46) |
| SVS | SHBG | rs79600740 | 1.04(0.74,1.46) |
| SVS | SHBG | rs79875164 | 1.06(0.76,1.48) |
| SVS | SHBG | rs8001781 | 1.05(0.75,1.47) |
| SVS | SHBG | rs8031716 | 1.04(0.74,1.46) |
| SVS | SHBG | rs8043101 | 1.03(0.74,1.43) |
| SVS | SHBG | rs8066941 | 1.02(0.73,1.43) |
| SVS | SHBG | rs8069105 | 1.03(0.74,1.44) |
| SVS | SHBG | rs8074363 | 1.03(0.74,1.44) |
| SVS | SHBG | rs8077323 | 1.06(0.76,1.47) |
| SVS | SHBG | rs8079418 | 1.05(0.75,1.47) |
| SVS | SHBG | rs8107967 | 1.04(0.74,1.45) |
| SVS | SHBG | rs8134638 | 1.05(0.75,1.46) |
| SVS | SHBG | rs841194 | 1.04(0.74,1.45) |
| SVS | SHBG | rs885683 | 1.04(0.74,1.46) |
| SVS | SHBG | rs901886 | 1.04(0.75,1.46) |
| SVS | SHBG | rs9533843 | 1.05(0.75,1.46) |
| SVS | SHBG | rs9556403 | 1.03(0.74,1.44) |
| SVS | SHBG | rs9597811 | 1.03(0.74,1.43) |
| SVS | SHBG | rs9610329 | 1.05(0.75,1.47) |
| SVS | SHBG | rs9614162 | 1.04(0.74,1.46) |
| SVS | SHBG | rs9893194 | 1.04(0.75,1.46) |
| SVS | SHBG | rs9902384 | 1.02(0.73,1.42) |
| SVS | SHBG | rs9972653 | 1.05(0.75,1.47) |

**Table S3** SNPs used as IVs for stroke in reverse MR analysis

| SNP | effect allele | | | other allele | | se | pval | | | beta |
| --- | --- | --- | --- | --- | --- | --- | --- | --- | --- | --- |
| rs11587860 | C | | G | | 2.54E-12 | 0.0098 | | | -0.0689 | |
| rs2634074 | A | T | 6.56E-14 | | 0.0112 | -0.0840 | |  |  |  |
| rs11242678 | T | C | 8.71E-10 | | 0.0105 | 0.0643 | |  |  |  |
| rs2107595 | A | G | 3.59E-11 | | 0.0121 | 0.0803 | |  |  |  |
| rs1537375 | C | T | 1.24E-08 | | 0.0091 | 0.0519 | |  |  |  |
| rs475937 | C | A | 2.92E-08 | | 0.0137 | -0.0757 | |  |  |  |
| rs10774624 | A | G | 4.04E-12 | | 0.0094 | -0.0654 | |  |  |  |
| rs4942561 | T | G | 2.05E-09 | | 0.0107 | 0.0640 | |  |  |  |

SNP, single nucleotide polymorphism; se, standard error; IV, instrumental variable; MR, mendelian randomization

**Table S4** SNPs used as IVs for IS in reverse MR analysis

| SNP | effect allele | | | other allele | | se | pval | | | beta |
| --- | --- | --- | --- | --- | --- | --- | --- | --- | --- | --- |
| rs2758612 | C | | T | | 0.0111 | 3.68E-09 | | | -0.0653 | |
| rs34311906 | C | T | 0.0113 | | 1.07E-08 | 0.0649 | |  |  |  |
| rs2634074 | A | T | 0.0121 | | 5.90E-15 | -0.0941 | |  |  |  |
| rs2066864 | A | G | 0.0115 | | 3.51E-08 | 0.0634 | |  |  |  |
| rs11242678 | T | C | 0.0114 | | 2.70E-10 | 0.0723 | |  |  |  |
| rs2107595 | A | G | 0.0132 | | 2.33E-11 | 0.0882 | |  |  |  |
| rs473238 | C | T | 0.0147 | | 1.65E-08 | -0.0831 | |  |  |  |
| rs3184504 | C | T | 0.0101 | | 1.23E-14 | -0.0779 | |  |  |  |
| rs4942561 | T | G | 0.0116 | | 1.77E-08 | 0.0655 | |  |  |  |

SNP, single nucleotide polymorphism; se, standard error; IV, instrumental variable; MR, mendelian randomization, IS, ischemic stroke

**Table S5** SNPs used as IVs for LAS in reverse MR analysis

| SNP | effect allele | other allele | se | pval | beta |
| --- | --- | --- | --- | --- | --- |
| rs7610618 | T | C | 1.44E-08 | 0.1490 | 0.8449 |
| rs2107595 | A | G | 1.44E-13 | 0.0319 | 0.2358 |
| rs10820405 | A | G | 4.51E-08 | 0.0331 | -0.1812 |
| rs476762 | A | T | 1.22E-08 | 0.0353 | 0.2010 |

SNP, single nucleotide polymorphism; se, standard error; IV, instrumental variable; MR, mendelian randomization, LAS, large artery stroke

**Table S6** SNPs used as IVs for CES in reverse MR analysis

| SNP | | effect allele | | other allele | | | se | | pval | | | beta |
| --- | --- | --- | --- | --- | --- | --- | --- | --- | --- | --- | --- | --- |
| rs146390073 | | T | | C | | 2.20E-08 | | 0.6688 | | | 0.1195 | |
| rs2466455 | T | | C | 2.75E-41 | -0.2992 | | 0.0222 | | |  |  |  |
| rs7680240 | C | | A | 1.17E-08 | -0.1163 | | 0.0204 | | |  |  |  |
| rs12932445 | C | | T | 6.88E-13 | 0.1758 | | 0.0245 | | |  |  |  |

SNP, single nucleotide polymorphism; se, standard error; IV, instrumental variable; MR, mendelian randomization, CES, cardioembolic stroke

**Table S7** SNPs used as IVs for BMI, HDL-C, Estradiol, DHEAS and SHBG in MVMR analysis

| SNP | effect allele | | | | | | other allele | | | | se | | | pval | | | | | beta | | | | |
| --- | --- | --- | --- | --- | --- | --- | --- | --- | --- | --- | --- | --- | --- | --- | --- | --- | --- | --- | --- | --- | --- | --- | --- |
| **BMI** | |  | | | | |  | | | |  | | | | | |  | | |  | | | |
| rs10009336 | | | | T | | | C | | | | -1.40E-02 | | | | 2.20E-03 | | | | | | 2.20E-10 | | |
| rs10027275 | | | C | | | G | | | -4.80E-03 | | | 2.10E-03 | | | | 1.90E-02 | | | | | |  |  |
| rs10123811 | | | T | | | C | | | -1.60E-03 | | | 1.70E-03 | | | | 3.70E-01 | | | | | |  |  |
| rs10132280 | | | A | | | C | | | -2.23E-02 | | | 1.80E-03 | | | | 5.60E-35 | | | | | |  |  |
| rs10182181 | | | G | | | A | | | 3.25E-02 | | | 1.60E-03 | | | | 6.70E-90 | | | | | |  |  |
| rs10197031 | | | C | | | T | | | 1.66E-02 | | | 1.90E-03 | | | | 1.90E-18 | | | | | |  |  |
| rs10208512 | | | A | | | G | | | -3.84E-02 | | | 4.30E-03 | | | | 4.50E-19 | | | | | |  |  |
| rs102275 | | | C | | | T | | | 3.20E-03 | | | 1.70E-03 | | | | 6.30E-02 | | | | | |  |  |
| rs10238028 | | | G | | | A | | | -1.09E-02 | | | 3.40E-03 | | | | 1.40E-03 | | | | | |  |  |
| rs10243319 | | | C | | | T | | | -1.07E-02 | | | 1.80E-03 | | | | 1.20E-09 | | | | | |  |  |
| rs10245356 | | | T | | | C | | | -5.00E-04 | | | 1.80E-03 | | | | 7.60E-01 | | | | | |  |  |
| rs10247983 | | | A | | | G | | | 2.01E-02 | | | 3.30E-03 | | | | 1.70E-09 | | | | | |  |  |
| rs10248136 | | | T | | | C | | | -9.70E-03 | | | 1.70E-03 | | | | 2.00E-08 | | | | | |  |  |
| rs10278546 | | | C | | | A | | | -1.60E-03 | | | 2.20E-03 | | | | 4.80E-01 | | | | | |  |  |
| rs10468017 | | | T | | | C | | | -1.70E-03 | | | 1.80E-03 | | | | 3.50E-01 | | | | | |  |  |
| rs10478110 | | | C | | | A | | | 1.00E-02 | | | 1.70E-03 | | | | 9.60E-09 | | | | | |  |  |
| rs10742752 | | | C | | | T | | | 1.24E-02 | | | 1.70E-03 | | | | 1.10E-13 | | | | | |  |  |
| rs10747488 | | | A | | | C | | | -1.23E-02 | | | 2.00E-03 | | | | 1.20E-09 | | | | | |  |  |
| rs10768994 | | | C | | | T | | | -1.14E-02 | | | 1.70E-03 | | | | 6.40E-12 | | | | | |  |  |
| rs10795422 | | | G | | | A | | | 1.39E-02 | | | 1.90E-03 | | | | 9.30E-14 | | | | | |  |  |
| rs10808546 | | | T | | | C | | | 8.90E-03 | | | 1.60E-03 | | | | 5.20E-08 | | | | | |  |  |
| rs10864070 | | | A | | | G | | | -1.00E-03 | | | 3.00E-03 | | | | 7.30E-01 | | | | | |  |  |
| rs10867256 | | | T | | | C | | | -1.18E-02 | | | 1.70E-03 | | | | 8.70E-12 | | | | | |  |  |
| rs10895276 | | | T | | | C | | | -3.70E-03 | | | 1.80E-03 | | | | 4.10E-02 | | | | | |  |  |
| rs10920678 | | | G | | | A | | | -1.55E-02 | | | 1.60E-03 | | | | 1.50E-21 | | | | | |  |  |
| rs10938397 | | | G | | | A | | | 3.24E-02 | | | 1.60E-03 | | | | 3.40E-86 | | | | | |  |  |
| rs10942267 | | | G | | | A | | | -1.56E-02 | | | 1.90E-03 | | | | 3.90E-17 | | | | | |  |  |
| rs10953740 | | | G | | | A | | | -1.53E-02 | | | 1.70E-03 | | | | 1.00E-18 | | | | | |  |  |
| rs10968114 | | | C | | | A | | | -1.13E-02 | | | 1.70E-03 | | | | 6.10E-11 | | | | | |  |  |
| rs10971709 | | | T | | | C | | | 1.32E-02 | | | 2.10E-03 | | | | 6.20E-10 | | | | | |  |  |
| rs11030618 | | | T | | | C | | | 1.10E-02 | | | 1.70E-03 | | | | 2.40E-10 | | | | | |  |  |
| rs1105977 | | | T | | | G | | | -4.40E-03 | | | 2.00E-03 | | | | 2.50E-02 | | | | | |  |  |
| rs11084553 | | | G | | | A | | | -2.10E-02 | | | 2.40E-03 | | | | 1.80E-18 | | | | | |  |  |
| rs11115176 | | | C | | | T | | | -1.21E-02 | | | 1.90E-03 | | | | 2.00E-10 | | | | | |  |  |
| rs1112613 | | | A | | | G | | | -1.33E-02 | | | 2.30E-03 | | | | 3.40E-09 | | | | | |  |  |
| rs11155787 | | | T | | | C | | | -2.90E-03 | | | 1.80E-03 | | | | 1.10E-01 | | | | | |  |  |
| rs11165643 | | | T | | | C | | | 2.06E-02 | | | 1.70E-03 | | | | 1.40E-35 | | | | | |  |  |
| rs11170468 | | | C | | | A | | | -1.23E-02 | | | 1.90E-03 | | | | 1.90E-10 | | | | | |  |  |
| rs11251352 | | | G | | | A | | | 1.09E-02 | | | 1.80E-03 | | | | 7.00E-10 | | | | | |  |  |
| rs1144387 | | | C | | | G | | | 9.80E-03 | | | 1.70E-03 | | | | 1.60E-08 | | | | | |  |  |
| rs11505821 | | | T | | | A | | | 3.11E-02 | | | 3.50E-03 | | | | 2.70E-19 | | | | | |  |  |
| rs11556924 | | | T | | | C | | | -5.60E-03 | | | 1.70E-03 | | | | 1.10E-03 | | | | | |  |  |
| rs1158805 | | | A | | | C | | | -1.37E-02 | | | 1.80E-03 | | | | 1.20E-14 | | | | | |  |  |
| rs11621792 | | | C | | | T | | | -4.00E-04 | | | 1.70E-03 | | | | 8.10E-01 | | | | | |  |  |
| rs11640366 | | | A | | | C | | | 8.10E-03 | | | 1.70E-03 | | | | 2.70E-06 | | | | | |  |  |
| rs11656076 | | | A | | | G | | | -1.42E-02 | | | 2.10E-03 | | | | 5.60E-12 | | | | | |  |  |
| rs11713193 | | | A | | | G | | | 2.39E-02 | | | 1.70E-03 | | | | 2.40E-44 | | | | | |  |  |
| rs11738695 | | | A | | | C | | | 9.70E-03 | | | 1.70E-03 | | | | 2.00E-08 | | | | | |  |  |
| rs117589665 | | | G | | | A | | | -5.40E-03 | | | 3.50E-03 | | | | 1.20E-01 | | | | | |  |  |
| rs11765639 | | | A | | | G | | | 2.80E-03 | | | 1.80E-03 | | | | 1.20E-01 | | | | | |  |  |
| rs11855853 | | | T | | | C | | | -1.45E-02 | | | 2.00E-03 | | | | 2.40E-13 | | | | | |  |  |
| rs1187352 | | | C | | | T | | | 1.19E-02 | | | 1.80E-03 | | | | 6.00E-11 | | | | | |  |  |
| rs11880870 | | | G | | | A | | | -1.89E-02 | | | 1.70E-03 | | | | 1.00E-28 | | | | | |  |  |
| rs12044597 | | | G | | | A | | | 1.43E-02 | | | 1.60E-03 | | | | 1.70E-18 | | | | | |  |  |
| rs12049202 | | | T | | | C | | | 2.40E-02 | | | 2.20E-03 | | | | 1.00E-28 | | | | | |  |  |
| rs12098284 | | | T | | | C | | | 1.78E-02 | | | 2.60E-03 | | | | 1.80E-11 | | | | | |  |  |
| rs12150665 | | | C | | | T | | | -1.62E-02 | | | 1.70E-03 | | | | 1.60E-22 | | | | | |  |  |
| rs1218822 | | | A | | | G | | | 1.68E-02 | | | 1.70E-03 | | | | 1.90E-22 | | | | | |  |  |
| rs12299814 | | | A | | | C | | | -1.57E-02 | | | 2.00E-03 | | | | 5.20E-15 | | | | | |  |  |
| rs12320328 | | | G | | | A | | | -4.00E-04 | | | 3.00E-03 | | | | 9.00E-01 | | | | | |  |  |
| rs12364470 | | | G | | | T | | | 1.78E-02 | | | 2.20E-03 | | | | 1.10E-15 | | | | | |  |  |
| rs1241986 | | | A | | | G | | | -1.39E-02 | | | 2.40E-03 | | | | 1.10E-08 | | | | | |  |  |
| rs12429545 | | | A | | | G | | | 3.16E-02 | | | 2.50E-03 | | | | 9.60E-38 | | | | | |  |  |
| rs12448257 | | | A | | | G | | | 1.84E-02 | | | 2.00E-03 | | | | 8.10E-20 | | | | | |  |  |
| rs12543287 | | | C | | | G | | | -3.10E-03 | | | 1.90E-03 | | | | 9.30E-02 | | | | | |  |  |
| rs12629015 | | | G | | | A | | | -1.35E-02 | | | 2.30E-03 | | | | 2.10E-09 | | | | | |  |  |
| rs1266874 | | | G | | | A | | | 1.40E-02 | | | 1.80E-03 | | | | 9.80E-15 | | | | | |  |  |
| rs12675063 | | | T | | | A | | | 1.56E-02 | | | 2.60E-03 | | | | 1.30E-09 | | | | | |  |  |
| rs1268065 | | | A | | | G | | | -1.02E-02 | | | 1.70E-03 | | | | 1.00E-09 | | | | | |  |  |
| rs12680842 | | | G | | | A | | | -1.33E-02 | | | 1.80E-03 | | | | 4.40E-14 | | | | | |  |  |
| rs12696304 | | | G | | | C | | | -3.70E-03 | | | 1.90E-03 | | | | 5.60E-02 | | | | | |  |  |
| rs12718572 | | | T | | | C | | | -1.17E-02 | | | 1.80E-03 | | | | 3.00E-11 | | | | | |  |  |
| rs12762034 | | | C | | | T | | | 2.40E-02 | | | 3.20E-03 | | | | 7.30E-14 | | | | | |  |  |
| rs12914489 | | | A | | | G | | | 1.65E-02 | | | 2.60E-03 | | | | 3.80E-10 | | | | | |  |  |
| rs12939549 | | | G | | | A | | | -1.80E-02 | | | 1.60E-03 | | | | 2.70E-28 | | | | | |  |  |
| rs1296328 | | | C | | | A | | | -1.79E-02 | | | 1.80E-03 | | | | 4.90E-24 | | | | | |  |  |
| rs13021737 | | | G | | | A | | | 5.74E-02 | | | 2.10E-03 | | | | 7.50E-157 | | | | | |  |  |
| rs13035806 | | | A | | | G | | | 8.00E-04 | | | 2.70E-03 | | | | 7.70E-01 | | | | | |  |  |
| rs13047416 | | | G | | | C | | | -1.54E-02 | | | 1.80E-03 | | | | 2.20E-17 | | | | | |  |  |
| rs13069244 | | | A | | | G | | | 1.87E-02 | | | 3.20E-03 | | | | 3.00E-09 | | | | | |  |  |
| rs13094241 | | | G | | | T | | | -8.20E-03 | | | 2.00E-03 | | | | 2.80E-05 | | | | | |  |  |
| rs13107325 | | | T | | | C | | | 4.70E-02 | | | 3.20E-03 | | | | 1.10E-47 | | | | | |  |  |
| rs13110266 | | | A | | | G | | | -1.17E-02 | | | 1.70E-03 | | | | 1.90E-12 | | | | | |  |  |
| rs13150068 | | | G | | | A | | | 4.90E-03 | | | 1.70E-03 | | | | 4.40E-03 | | | | | |  |  |
| rs13174863 | | | G | | | A | | | 1.92E-02 | | | 2.30E-03 | | | | 2.90E-16 | | | | | |  |  |
| rs13191362 | | | G | | | A | | | -2.36E-02 | | | 2.50E-03 | | | | 5.90E-21 | | | | | |  |  |
| rs1320903 | | | A | | | G | | | 2.16E-02 | | | 1.80E-03 | | | | 9.20E-32 | | | | | |  |  |
| rs1321432 | | | C | | | A | | | 2.01E-02 | | | 1.80E-03 | | | | 3.50E-29 | | | | | |  |  |
| rs13251458 | | | A | | | G | | | -1.00E-03 | | | 1.70E-03 | | | | 5.70E-01 | | | | | |  |  |
| rs1327259 | | | G | | | A | | | -1.55E-02 | | | 1.80E-03 | | | | 1.70E-18 | | | | | |  |  |
| rs13389219 | | | T | | | C | | | 1.12E-02 | | | 1.70E-03 | | | | 1.20E-11 | | | | | |  |  |
| rs1365466 | | | T | | | C | | | -1.37E-02 | | | 1.90E-03 | | | | 3.30E-13 | | | | | |  |  |
| rs13702 | | | C | | | T | | | -3.80E-03 | | | 1.80E-03 | | | | 3.60E-02 | | | | | |  |  |
| rs1371108 | | | A | | | C | | | 1.19E-02 | | | 1.80E-03 | | | | 9.00E-11 | | | | | |  |  |
| rs1409818 | | | T | | | C | | | 2.01E-02 | | | 2.90E-03 | | | | 2.50E-12 | | | | | |  |  |
| rs1412235 | | | C | | | G | | | 2.46E-02 | | | 1.70E-03 | | | | 6.00E-45 | | | | | |  |  |
| rs1421334 | | | C | | | A | | | -1.25E-02 | | | 1.80E-03 | | | | 1.00E-12 | | | | | |  |  |
| rs1452075 | | | T | | | C | | | 1.41E-02 | | | 1.80E-03 | | | | 1.30E-14 | | | | | |  |  |
| rs1454687 | | | G | | | C | | | -2.02E-02 | | | 1.70E-03 | | | | 5.20E-32 | | | | | |  |  |
| rs1465900 | | | C | | | A | | | -1.25E-02 | | | 2.00E-03 | | | | 4.80E-10 | | | | | |  |  |
| rs1472169 | | | T | | | C | | | -1.39E-02 | | | 1.80E-03 | | | | 2.80E-15 | | | | | |  |  |
| rs150449323 | | | C | | | T | | | -5.70E-03 | | | 3.10E-03 | | | | 6.50E-02 | | | | | |  |  |
| rs1521527 | | | C | | | G | | | -1.21E-02 | | | 1.70E-03 | | | | 3.10E-12 | | | | | |  |  |
| rs1528435 | | | T | | | C | | | 1.64E-02 | | | 1.70E-03 | | | | 9.10E-23 | | | | | |  |  |
| rs1534696 | | | A | | | C | | | -2.60E-03 | | | 1.70E-03 | | | | 1.10E-01 | | | | | |  |  |
| rs1561442 | | | A | | | G | | | 6.30E-03 | | | 2.60E-03 | | | | 1.30E-02 | | | | | |  |  |
| rs157935 | | | G | | | T | | | 2.40E-03 | | | 1.90E-03 | | | | 2.10E-01 | | | | | |  |  |
| rs1624134 | | | C | | | G | | | 1.01E-02 | | | 1.80E-03 | | | | 1.10E-08 | | | | | |  |  |
| rs1640269 | | | C | | | A | | | -5.30E-03 | | | 1.90E-03 | | | | 5.90E-03 | | | | | |  |  |
| rs1650548 | | | C | | | G | | | -6.40E-03 | | | 2.10E-03 | | | | 2.10E-03 | | | | | |  |  |
| rs1656377 | | | C | | | T | | | 9.90E-03 | | | 1.70E-03 | | | | 1.60E-08 | | | | | |  |  |
| rs1681740 | | | C | | | A | | | -1.15E-02 | | | 1.80E-03 | | | | 1.10E-10 | | | | | |  |  |
| rs16849710 | | | G | | | A | | | -1.16E-02 | | | 1.80E-03 | | | | 6.00E-11 | | | | | |  |  |
| rs16851483 | | | T | | | G | | | 3.69E-02 | | | 3.50E-03 | | | | 3.20E-26 | | | | | |  |  |
| rs16903285 | | | C | | | T | | | 3.31E-02 | | | 2.60E-03 | | | | 7.60E-38 | | | | | |  |  |
| rs16942887 | | | A | | | G | | | -4.30E-03 | | | 2.50E-03 | | | | 8.90E-02 | | | | | |  |  |
| rs17014375 | | | G | | | T | | | 1.72E-02 | | | 2.50E-03 | | | | 1.10E-11 | | | | | |  |  |
| rs17033117 | | | T | | | C | | | 1.37E-02 | | | 2.20E-03 | | | | 8.90E-10 | | | | | |  |  |
| rs17056301 | | | C | | | T | | | 1.18E-02 | | | 2.00E-03 | | | | 2.40E-09 | | | | | |  |  |
| rs17119937 | | | C | | | T | | | 2.12E-02 | | | 3.60E-03 | | | | 5.60E-09 | | | | | |  |  |
| rs17184382 | | | C | | | A | | | 3.30E-03 | | | 1.70E-03 | | | | 5.00E-02 | | | | | |  |  |
| rs17207196 | | | T | | | C | | | -2.21E-02 | | | 1.80E-03 | | | | 2.10E-35 | | | | | |  |  |
| rs1730862 | | | A | | | G | | | -1.19E-02 | | | 1.80E-03 | | | | 7.90E-11 | | | | | |  |  |
| rs17399237 | | | C | | | T | | | -1.29E-02 | | | 1.70E-03 | | | | 6.70E-14 | | | | | |  |  |
| rs17425707 | | | C | | | T | | | 1.67E-02 | | | 2.80E-03 | | | | 4.40E-09 | | | | | |  |  |
| rs17535749 | | | A | | | G | | | 1.50E-02 | | | 2.70E-03 | | | | 2.50E-08 | | | | | |  |  |
| rs17551974 | | | A | | | C | | | -1.41E-02 | | | 2.20E-03 | | | | 1.90E-10 | | | | | |  |  |
| rs1772189 | | | A | | | T | | | -4.50E-03 | | | 1.70E-03 | | | | 8.20E-03 | | | | | |  |  |
| rs17789218 | | | C | | | T | | | 1.30E-02 | | | 1.90E-03 | | | | 7.40E-12 | | | | | |  |  |
| rs1801282 | | | G | | | C | | | 1.73E-02 | | | 2.50E-03 | | | | 2.50E-12 | | | | | |  |  |
| rs1863652 | | | A | | | G | | | -1.15E-02 | | | 1.80E-03 | | | | 1.40E-10 | | | | | |  |  |
| rs1866956 | | | T | | | C | | | -3.20E-03 | | | 1.80E-03 | | | | 7.40E-02 | | | | | |  |  |
| rs1883025 | | | T | | | C | | | 6.50E-03 | | | 1.90E-03 | | | | 5.80E-04 | | | | | |  |  |
| rs1891216 | | | G | | | T | | | 1.07E-02 | | | 1.80E-03 | | | | 2.40E-09 | | | | | |  |  |
| rs189595752 | | | G | | | A | | | -8.00E-03 | | | 2.80E-03 | | | | 4.20E-03 | | | | | |  |  |
| rs1928295 | | | C | | | T | | | -1.41E-02 | | | 1.60E-03 | | | | 5.40E-18 | | | | | |  |  |
| rs1982725 | | | T | | | C | | | 9.70E-03 | | | 1.70E-03 | | | | 3.30E-08 | | | | | |  |  |
| rs200810 | | | C | | | T | | | -1.36E-02 | | | 1.70E-03 | | | | 5.50E-16 | | | | | |  |  |
| rs2009416 | | | T | | | C | | | -1.21E-02 | | | 1.80E-03 | | | | 1.10E-11 | | | | | |  |  |
| rs2065418 | | | G | | | T | | | -1.66E-02 | | | 1.80E-03 | | | | 3.60E-20 | | | | | |  |  |
| rs215634 | | | G | | | A | | | -1.52E-02 | | | 1.80E-03 | | | | 2.60E-17 | | | | | |  |  |
| rs2174307 | | | C | | | G | | | 1.21E-02 | | | 1.70E-03 | | | | 4.90E-12 | | | | | |  |  |
| rs217671 | | | G | | | A | | | 1.44E-02 | | | 1.90E-03 | | | | 1.30E-13 | | | | | |  |  |
| rs2224585 | | | A | | | G | | | -3.00E-03 | | | 2.00E-03 | | | | 1.40E-01 | | | | | |  |  |
| rs2241210 | | | G | | | A | | | 8.80E-03 | | | 1.60E-03 | | | | 7.00E-08 | | | | | |  |  |
| rs2246012 | | | C | | | T | | | 1.58E-02 | | | 2.20E-03 | | | | 3.10E-13 | | | | | |  |  |
| rs2325036 | | | C | | | A | | | -1.81E-02 | | | 1.70E-03 | | | | 3.60E-27 | | | | | |  |  |
| rs2351958 | | | A | | | C | | | 1.30E-03 | | | 1.80E-03 | | | | 4.80E-01 | | | | | |  |  |
| rs2357760 | | | A | | | G | | | 1.45E-02 | | | 1.70E-03 | | | | 6.80E-17 | | | | | |  |  |
| rs2393791 | | | T | | | C | | | 1.50E-03 | | | 1.70E-03 | | | | 3.60E-01 | | | | | |  |  |
| rs2479958 | | | G | | | A | | | -1.54E-02 | | | 1.80E-03 | | | | 1.50E-17 | | | | | |  |  |
| rs2481665 | | | C | | | T | | | -1.61E-02 | | | 1.60E-03 | | | | 7.20E-23 | | | | | |  |  |
| rs2498786 | | | G | | | C | | | 5.70E-03 | | | 1.70E-03 | | | | 8.10E-04 | | | | | |  |  |
| rs2543132 | | | C | | | G | | | 1.46E-02 | | | 2.20E-03 | | | | 5.00E-11 | | | | | |  |  |
| rs2551644 | | | A | | | T | | | 9.90E-03 | | | 2.20E-03 | | | | 7.20E-06 | | | | | |  |  |
| rs2608703 | | | A | | | C | | | 1.42E-02 | | | 1.70E-03 | | | | 1.90E-16 | | | | | |  |  |
| rs2612038 | | | T | | | C | | | -2.00E-04 | | | 2.70E-03 | | | | 9.40E-01 | | | | | |  |  |
| rs2642438 | | | G | | | A | | | -3.20E-03 | | | 1.80E-03 | | | | 7.40E-02 | | | | | |  |  |
| rs2643452 | | | A | | | T | | | 1.36E-02 | | | 1.70E-03 | | | | 4.70E-15 | | | | | |  |  |
| rs2693826 | | | A | | | G | | | -1.37E-02 | | | 1.70E-03 | | | | 2.00E-15 | | | | | |  |  |
| rs2694047 | | | G | | | A | | | 1.88E-02 | | | 2.00E-03 | | | | 3.90E-21 | | | | | |  |  |
| rs273504 | | | G | | | A | | | 1.53E-02 | | | 1.80E-03 | | | | 4.40E-18 | | | | | |  |  |
| rs2744974 | | | T | | | C | | | 2.49E-02 | | | 1.80E-03 | | | | 1.40E-45 | | | | | |  |  |
| rs2791644 | | | | | C | | | T | | 1.42E-02 | | | 2.00E-03 | | | | | 1.20E-12 | | | | |  |
| rs28507491 | | | | | A | | | G | | 4.20E-03 | | | 1.80E-03 | | | | | 1.80E-02 | | | | |  |
| rs2861683 | | | | | C | | | A | | -1.44E-02 | | | 1.70E-03 | | | | | 1.30E-16 | | | | |  |
| rs2868975 | | | | | A | | | G | | -1.43E-02 | | | 2.30E-03 | | | | | 2.20E-10 | | | | |  |
| rs2875762 | | | | | C | | | G | | 1.39E-02 | | | 2.00E-03 | | | | | 1.20E-11 | | | | |  |
| rs2931434 | | | | | T | | | C | | -1.04E-02 | | | 1.80E-03 | | | | | 1.40E-08 | | | | |  |
| rs2972145 | | | | | C | | | T | | -6.10E-03 | | | 1.70E-03 | | | | | 2.80E-04 | | | | |  |
| rs3001032 | | | | | C | | | T | | 8.00E-03 | | | 1.80E-03 | | | | | 1.40E-05 | | | | |  |
| rs34255979 | | | | | T | | | C | | 4.00E-04 | | | 2.80E-03 | | | | | 8.80E-01 | | | | |  |
| rs349088 | | | | | A | | | C | | -1.28E-02 | | | 1.70E-03 | | | | | 1.80E-13 | | | | |  |
| rs35350976 | | | | | G | | | A | | 2.70E-03 | | | 2.20E-03 | | | | | 2.30E-01 | | | | |  |
| rs355777 | | | | | C | | | G | | 1.53E-02 | | | 1.70E-03 | | | | | 1.40E-18 | | | | |  |
| rs3749897 | | | | | T | | | C | | 1.22E-02 | | | 1.80E-03 | | | | | 8.40E-12 | | | | |  |
| rs3754963 | | | | | T | | | A | | -1.23E-02 | | | 2.00E-03 | | | | | 3.30E-10 | | | | |  |
| rs3772882 | | | | | A | | | C | | 1.27E-02 | | | 1.80E-03 | | | | | 6.60E-13 | | | | |  |
| rs3800229 | | | | | T | | | G | | 1.75E-02 | | | 1.80E-03 | | | | | 1.40E-22 | | | | |  |
| rs3806114 | | | | | A | | | G | | -1.13E-02 | | | 1.80E-03 | | | | | 3.40E-10 | | | | |  |
| rs3806572 | | | | | A | | | G | | -1.45E-02 | | | 1.90E-03 | | | | | 1.60E-14 | | | | |  |
| rs3807645 | | | | | A | | | G | | -1.66E-02 | | | 2.10E-03 | | | | | 2.40E-15 | | | | |  |
| rs380857 | | | | | A | | | C | | -1.51E-02 | | | 2.70E-03 | | | | | 3.60E-08 | | | | |  |
| rs3810027 | | | | | G | | | C | | -9.00E-04 | | | 1.80E-03 | | | | | 6.30E-01 | | | | |  |
| rs3829639 | | | | | G | | | A | | -6.00E-03 | | | 2.00E-03 | | | | | 3.20E-03 | | | | |  |
| rs38314 | | | | | A | | | G | | -1.20E-02 | | | 1.70E-03 | | | | | 4.70E-12 | | | | |  |
| rs3902951 | | | | | G | | | T | | 1.34E-02 | | | 2.00E-03 | | | | | 7.00E-12 | | | | |  |
| rs3904244 | | | | | A | | | T | | 1.55E-02 | | | 2.50E-03 | | | | | 4.30E-10 | | | | |  |
| rs40067 | | | | | A | | | G | | -2.66E-02 | | | 2.30E-03 | | | | | 7.10E-30 | | | | |  |
| rs40270 | | | | | C | | | A | | -1.50E-03 | | | 1.90E-03 | | | | | 4.20E-01 | | | | |  |
| rs4072917 | | | | | A | | | G | | 1.15E-02 | | | 1.80E-03 | | | | | 6.90E-11 | | | | |  |
| rs4148005 | | | | | G | | | T | | 8.00E-04 | | | 1.80E-03 | | | | | 6.60E-01 | | | | |  |
| rs4148155 | | | | | G | | | A | | -1.88E-02 | | | 2.60E-03 | | | | | 5.00E-13 | | | | |  |
| rs4237643 | | | | | G | | | T | | -2.23E-02 | | | 1.90E-03 | | | | | 4.30E-33 | | | | |  |
| rs4240624 | | | | | A | | | G | | -5.00E-04 | | | 2.80E-03 | | | | | 8.50E-01 | | | | |  |
| rs427943 | | | | | C | | | A | | 1.70E-02 | | | 1.70E-03 | | | | | 7.30E-23 | | | | |  |
| rs4307239 | | | | | G | | | A | | 1.15E-02 | | | 1.70E-03 | | | | | 3.90E-11 | | | | |  |
| rs4338849 | | | | | A | | | G | | 3.10E-03 | | | 1.90E-03 | | | | | 1.10E-01 | | | | |  |
| rs4430672 | | | | | C | | | T | | -1.27E-02 | | | 2.20E-03 | | | | | 3.90E-09 | | | | |  |
| rs4556997 | | | | | A | | | C | | 1.97E-02 | | | 2.40E-03 | | | | | 6.90E-17 | | | | |  |
| rs4567095 | | | | | T | | | C | | -3.00E-03 | | | 1.80E-03 | | | | | 9.20E-02 | | | | |  |
| rs4568281 | | | | | A | | | G | | 1.00E-03 | | | 2.00E-03 | | | | | 6.20E-01 | | | | |  |
| rs4589691 | | | | | G | | | C | | 1.41E-02 | | | 2.40E-03 | | | | | 4.70E-09 | | | | |  |
| rs4639527 | | | | | G | | | A | | 1.72E-02 | | | 1.90E-03 | | | | | 3.30E-20 | | | | |  |
| rs4639796 | | | | | A | | | G | | 5.50E-03 | | | 2.30E-03 | | | | | 1.70E-02 | | | | |  |
| rs4660443 | | | | | T | | | C | | 1.64E-02 | | | 2.10E-03 | | | | | 6.80E-15 | | | | |  |
| rs4665972 | | | | | C | | | T | | 1.05E-02 | | | 1.70E-03 | | | | | 3.90E-10 | | | | |  |
| rs4671328 | | | | | G | | | T | | -2.19E-02 | | | 1.70E-03 | | | | | 2.20E-36 | | | | |  |
| rs4674669 | | | | | T | | | C | | 2.40E-03 | | | 2.50E-03 | | | | | 3.40E-01 | | | | |  |
| rs4740619 | | | | | C | | | T | | -1.86E-02 | | | 1.60E-03 | | | | | 2.30E-30 | | | | |  |
| rs4800191 | | | | | C | | | G | | 1.03E-02 | | | 1.70E-03 | | | | | 2.50E-09 | | | | |  |
| rs4804414 | | | | | T | | | C | | -2.80E-03 | | | 1.70E-03 | | | | | 1.10E-01 | | | | |  |
| rs4812336 | | | | | A | | | G | | 5.60E-03 | | | 1.90E-03 | | | | | 3.40E-03 | | | | |  |
| rs4813619 | | | | | T | | | G | | -1.08E-02 | | | 1.80E-03 | | | | | 2.30E-09 | | | | |  |
| rs4820091 | | | | | G | | | T | | 6.10E-03 | | | 2.10E-03 | | | | | 3.70E-03 | | | | |  |
| rs4820408 | | | | | G | | | T | | -1.51E-02 | | | 1.70E-03 | | | | | 2.10E-19 | | | | |  |
| rs4842491 | | | | | T | | | C | | 9.80E-03 | | | 1.80E-03 | | | | | 4.00E-08 | | | | |  |
| rs4846914 | | | | | A | | | G | | -6.90E-03 | | | 1.70E-03 | | | | | 3.10E-05 | | | | |  |
| rs4851029 | | | | | G | | | T | | 1.21E-02 | | | 1.70E-03 | | | | | 1.70E-12 | | | | |  |
| rs4880341 | | | | | T | | | C | | -1.18E-02 | | | 1.70E-03 | | | | | 1.10E-11 | | | | |  |
| rs4936175 | | | | | C | | | T | | 1.22E-02 | | | 1.70E-03 | | | | | 1.40E-12 | | | | |  |
| rs4939883 | | | | | C | | | T | | 3.90E-03 | | | 2.10E-03 | | | | | 6.50E-02 | | | | |  |
| rs4954638 | | | | | C | | | A | | -1.18E-02 | | | 2.00E-03 | | | | | 2.90E-09 | | | | |  |
| rs538579 | | | | | C | | | G | | 1.37E-02 | | | 1.90E-03 | | | | | 1.30E-13 | | | | |  |
| rs543874 | | | | | G | | | A | | 4.75E-02 | | | 2.00E-03 | | | | | 1.20E-122 | | | | |  |
| rs559231 | | | | | T | | | G | | 1.35E-02 | | | 1.80E-03 | | | | | 2.40E-14 | | | | |  |
| rs56024084 | | | | | T | | | C | | -2.30E-03 | | | 1.80E-03 | | | | | 1.80E-01 | | | | |  |
| rs56077345 | | | | | C | | | G | | 7.90E-03 | | | 3.10E-03 | | | | | 1.10E-02 | | | | |  |
| rs587252 | | | | | C | | | A | | 4.20E-03 | | | 3.20E-03 | | | | | 1.80E-01 | | | | |  |
| rs61856602 | | | | | G | | | A | | -2.90E-03 | | | 1.80E-03 | | | | | 1.20E-01 | | | | |  |
| rs6235 | | | | | G | | | C | | 1.75E-02 | | | 1.90E-03 | | | | | 1.50E-19 | | | | |  |
| rs62623385 | | | | | T | | | A | | -1.33E-02 | | | 4.70E-03 | | | | | 4.50E-03 | | | | |  |
| rs6265 | | | | | T | | | C | | -4.12E-02 | | | 2.10E-03 | | | | | 1.00E-86 | | | | |  |
| rs633695 | | | | | G | | | A | | -3.10E-03 | | | 1.90E-03 | | | | | 1.10E-01 | | | | |  |
| rs6471941 | | | | | A | | | G | | 1.56E-02 | | | 2.10E-03 | | | | | 3.10E-13 | | | | |  |
| rs6486122 | | | | | T | | | C | | 9.50E-03 | | | 1.80E-03 | | | | | 2.30E-07 | | | | |  |
| rs6512302 | | | | | C | | | G | | 1.42E-02 | | | 2.00E-03 | | | | | 2.10E-12 | | | | |  |
| rs6545714 | | | | | A | | | G | | -1.91E-02 | | | 1.70E-03 | | | | | 9.10E-31 | | | | |  |
| rs6561943 | | | | | T | | | C | | 1.19E-02 | | | 1.90E-03 | | | | | 4.20E-10 | | | | |  |
| rs657452 | | | | | G | | | A | | -1.88E-02 | | | 1.70E-03 | | | | | 7.20E-29 | | | | |  |
| rs6591407 | | | | | A | | | C | | -1.18E-02 | | | 2.10E-03 | | | | | 1.90E-08 | | | | |  |
| rs6595205 | | | | | G | | | C | | -1.14E-02 | | | 1.60E-03 | | | | | 2.00E-12 | | | | |  |
| rs663129 | | | | | A | | | G | | 5.45E-02 | | | 1.90E-03 | | | | | 1.60E-178 | | | | |  |
| rs668871 | | | | | T | | | C | | 1.08E-02 | | | 1.60E-03 | | | | | 2.70E-11 | | | | |  |
| rs6758199 | | | | | T | | | C | | -2.00E-03 | | | 3.40E-03 | | | | | 5.70E-01 | | | | |  |
| rs6785245 | | | | | C | | | T | | 1.32E-02 | | | 1.70E-03 | | | | | 4.00E-14 | | | | |  |
| rs6815910 | | | | | A | | | T | | -1.28E-02 | | | 1.70E-03 | | | | | 1.40E-13 | | | | |  |
| rs6841761 | | | | | T | | | G | | -1.31E-02 | | | 1.60E-03 | | | | | 6.40E-16 | | | | |  |
| rs6860245 | | | | | C | | | G | | 6.70E-03 | | | 2.00E-03 | | | | | 9.50E-04 | | | | |  |
| rs686030 | | | | | A | | | C | | -2.40E-03 | | | 2.30E-03 | | | | | 3.10E-01 | | | | |  |
| rs695272 | | | | | C | | | T | | 3.50E-03 | | | 1.90E-03 | | | | | 6.10E-02 | | | | |  |
| rs7015 | | | | | A | | | G | | 2.90E-03 | | | 2.20E-03 | | | | | 1.90E-01 | | | | |  |
| rs7025938 | | | | | G | | | C | | 1.66E-02 | | | 1.90E-03 | | | | | 3.70E-19 | | | | |  |
| rs705704 | | | | | A | | | G | | -1.31E-02 | | | 1.80E-03 | | | | | 1.90E-13 | | | | |  |
| rs7084454 | | | | | A | | | G | | 1.93E-02 | | | 1.90E-03 | | | | | 4.00E-25 | | | | |  |
| rs7096764 | | | | | A | | | G | | -3.20E-03 | | | 1.90E-03 | | | | | 8.60E-02 | | | | |  |
| rs7117238 | | | | | A | | | G | | -1.31E-02 | | | 2.20E-03 | | | | | 2.50E-09 | | | | |  |
| rs7138803 | | | | | A | | | G | | 3.00E-02 | | | 1.70E-03 | | | | | 2.30E-71 | | | | |  |
| rs7144011 | | | | | T | | | G | | 2.82E-02 | | | 2.00E-03 | | | | | 5.20E-47 | | | | |  |
| rs7148846 | | | | | G | | | T | | 1.24E-02 | | | 2.20E-03 | | | | | 2.20E-08 | | | | |  |
| rs7196720 | | | | | C | | | T | | -1.29E-02 | | | 1.70E-03 | | | | | 7.30E-14 | | | | |  |
| rs7222349 | | | | | A | | | G | | 1.15E-02 | | | 1.80E-03 | | | | | 3.30E-10 | | | | |  |
| rs7239575 | | | | | C | | | T | | -2.02E-02 | | | 1.70E-03 | | | | | 7.40E-32 | | | | |  |
| rs7262150 | | | | | C | | | T | | -7.40E-03 | | | 2.00E-03 | | | | | 2.00E-04 | | | | |  |
| rs73079476 | | | | | C | | | A | | 1.50E-03 | | | 2.40E-03 | | | | | 5.20E-01 | | | | |  |
| rs7314285 | | | | | G | | | T | | 1.09E-02 | | | 3.20E-03 | | | | | 7.80E-04 | | | | |  |
| rs7318817 | | | | | T | | | C | | -1.55E-02 | | | 1.80E-03 | | | | | 2.70E-18 | | | | |  |
| rs738409 | | | | | G | | | C | | -7.00E-03 | | | 2.10E-03 | | | | | 9.40E-04 | | | | |  |
| rs7451021 | | | | | C | | | T | | -1.10E-02 | | | 1.90E-03 | | | | | 4.10E-09 | | | | |  |
| rs7498665 | | | | | G | | | A | | 2.71E-02 | | | 1.70E-03 | | | | | 5.60E-60 | | | | |  |
| rs7557796 | | | | | C | | | T | | -1.60E-02 | | | 1.80E-03 | | | | | 2.30E-19 | | | | |  |
| rs757869 | | | | | G | | | A | | 1.20E-03 | | | 1.90E-03 | | | | | 5.30E-01 | | | | |  |
| rs7615297 | | | | | G | | | C | | -1.49E-02 | | | 2.40E-03 | | | | | 5.70E-10 | | | | |  |
| rs7626079 | | | | | T | | | C | | 1.10E-02 | | | 1.80E-03 | | | | | 1.60E-09 | | | | |  |
| rs7678138 | | | | | A | | | G | | -1.00E-04 | | | 2.60E-03 | | | | | 9.80E-01 | | | | |  |
| rs7683836 | | | | | A | | | G | | -1.14E-02 | | | 1.70E-03 | | | | | 6.30E-11 | | | | |  |
| rs7703576 | | | | | C | | | T | | 1.03E-02 | | | 1.90E-03 | | | | | 4.80E-08 | | | | |  |
| rs7715256 | | | | | T | | | G | | -1.66E-02 | | | 1.60E-03 | | | | | 2.20E-24 | | | | |  |
| rs7724675 | | | | | A | | | G | | -1.19E-02 | | | 2.10E-03 | | | | | 9.50E-09 | | | | |  |
| rs7730898 | | | | | A | | | G | | 1.68E-02 | | | 1.80E-03 | | | | | 4.50E-20 | | | | |  |
| rs7761673 | | | | | A | | | T | | -1.26E-02 | | | 2.10E-03 | | | | | 1.90E-09 | | | | |  |
| rs7780752 | | | | | C | | | T | | 1.39E-02 | | | 1.80E-03 | | | | | 1.00E-14 | | | | |  |
| rs7788008 | | | | | A | | | G | | -1.57E-02 | | | 1.70E-03 | | | | | 1.10E-19 | | | | |  |
| rs7819514 | | | | | A | | | G | | -1.07E-02 | | | 1.80E-03 | | | | | 5.70E-09 | | | | |  |
| rs7844647 | | | | | C | | | T | | -1.23E-02 | | | 1.80E-03 | | | | | 2.80E-11 | | | | |  |
| rs7860634 | | | | | A | | | G | | -2.40E-03 | | | 1.80E-03 | | | | | 1.80E-01 | | | | |  |
| rs7869771 | | | | | C | | | A | | -1.40E-02 | | | 1.90E-03 | | | | | 4.90E-13 | | | | |  |
| rs7925214 | | | | | T | | | C | | 1.47E-02 | | | 1.80E-03 | | | | | 4.40E-17 | | | | |  |
| rs796004 | | | | | T | | | C | | -3.00E-03 | | | 2.00E-03 | | | | | 1.30E-01 | | | | |  |
| rs79717793 | | | | | A | | | G | | 6.70E-03 | | | 2.40E-03 | | | | | 5.20E-03 | | | | |  |
| rs7983065 | | | | | T | | | C | | -1.48E-02 | | | 1.70E-03 | | | | | 8.90E-18 | | | | |  |
| rs802685 | | | | | C | | | T | | -9.00E-04 | | | 2.00E-03 | | | | | 6.40E-01 | | | | |  |
| rs8033077 | | | | | C | | | T | | -4.70E-03 | | | 1.90E-03 | | | | | 1.50E-02 | | | | |  |
| rs8047395 | | | | | A | | | G | | 6.42E-02 | | | 1.70E-03 | | | | | 1.00E-200 | | | | |  |
| rs806600 | | | | | G | | | A | | -9.50E-03 | | | 1.70E-03 | | | | | 3.30E-08 | | | | |  |
| rs8079418 | | | | | C | | | T | | 7.60E-03 | | | 1.80E-03 | | | | | 2.50E-05 | | | | |  |
| rs8097672 | | | | | T | | | A | | 2.00E-02 | | | 2.50E-03 | | | | | 8.40E-16 | | | | |  |
| rs8097783 | | | | | A | | | G | | -3.89E-02 | | | 3.10E-03 | | | | | 7.20E-36 | | | | |  |
| rs8181823 | | | | | C | | | A | | 1.27E-02 | | | 2.00E-03 | | | | | 4.10E-10 | | | | |  |
| rs865809 | | | | | G | | | A | | -1.27E-02 | | | 2.00E-03 | | | | | 5.40E-10 | | | | |  |
| rs872281 | | | | | T | | | C | | -1.51E-02 | | | 2.30E-03 | | | | | 4.70E-11 | | | | |  |
| rs876605 | | | | | G | | | A | | -1.08E-02 | | | 2.00E-03 | | | | | 3.40E-08 | | | | |  |
| rs879620 | | | | | T | | | C | | 2.31E-02 | | | 1.80E-03 | | | | | 5.30E-38 | | | | |  |
| rs889398 | | | | | T | | | C | | -1.96E-02 | | | 1.60E-03 | | | | | 1.30E-32 | | | | |  |
| rs901630 | | | | | T | | | C | | -1.46E-02 | | | 1.70E-03 | | | | | 1.90E-18 | | | | |  |
| rs902695 | | | | | A | | | G | | -1.03E-02 | | | 1.70E-03 | | | | | 2.20E-09 | | | | |  |
| rs9267551 | | | | | G | | | C | | 3.90E-03 | | | 2.30E-03 | | | | | 8.90E-02 | | | | |  |
| rs9294260 | | | | | A | | | G | | 1.47E-02 | | | 1.60E-03 | | | | | 1.80E-19 | | | | |  |
| rs9297994 | | | | | A | | | G | | -2.80E-03 | | | 1.80E-03 | | | | | 1.20E-01 | | | | |  |
| rs9300422 | | | | | G | | | A | | -1.03E-02 | | | 1.80E-03 | | | | | 4.00E-09 | | | | |  |
| rs9379084 | | | | | A | | | G | | 7.50E-03 | | | 2.80E-03 | | | | | 7.70E-03 | | | | |  |
| rs9408882 | | | | | A | | | G | | -9.30E-03 | | | 1.60E-03 | | | | | 1.30E-08 | | | | |  |
| rs946824 | | | | | C | | | T | | -2.06E-02 | | | 2.60E-03 | | | | | 1.10E-15 | | | | |  |
| rs947612 | | | | | A | | | G | | -1.16E-02 | | | 2.00E-03 | | | | | 5.60E-09 | | | | |  |
| rs9522285 | | | | | A | | | G | | 1.27E-02 | | | 1.70E-03 | | | | | 2.50E-13 | | | | |  |
| rs9538162 | | | | | C | | | T | | -1.56E-02 | | | 1.80E-03 | | | | | 4.80E-19 | | | | |  |
| rs9547153 | | | | | G | | | A | | 9.80E-03 | | | 1.70E-03 | | | | | 8.70E-09 | | | | |  |
| rs9571687 | | | | | A | | | C | | -1.29E-02 | | | 1.80E-03 | | | | | 2.80E-12 | | | | |  |
| rs9615905 | | | | | T | | | C | | 1.10E-02 | | | 1.70E-03 | | | | | 2.70E-10 | | | | |  |
| rs964184 | | | | | C | | | G | | 1.70E-03 | | | 2.50E-03 | | | | | 5.10E-01 | | | | |  |
| rs970548 | | | | | C | | | A | | -2.50E-03 | | | 1.90E-03 | | | | | 1.80E-01 | | | | |  |
| rs9739640 | | | | | G | | | A | | 1.12E-02 | | | 2.20E-03 | | | | | 6.30E-07 | | | | |  |
| rs977747 | | | | | G | | | T | | -1.69E-02 | | | 1.70E-03 | | | | | 1.30E-24 | | | | |  |
| rs9783858 | | | | | T | | | C | | 9.10E-03 | | | 1.70E-03 | | | | | 3.30E-08 | | | | |  |
| rs9806742 | | | | | A | | | G | | 2.08E-02 | | | 2.60E-03 | | | | | 1.40E-15 | | | | |  |
| rs9816226 | | | | | T | | | A | | 3.23E-02 | | | 2.10E-03 | | | | | 1.60E-52 | | | | |  |
| rs9845966 | | | | | G | | | T | | -1.05E-02 | | | 1.70E-03 | | | | | 2.50E-10 | | | | |  |
| rs9849171 | | | | | C | | | G | | 2.80E-03 | | | 1.70E-03 | | | | | 1.00E-01 | | | | |  |
| rs987237 | | | | | G | | | A | | 4.09E-02 | | | 2.10E-03 | | | | | 9.30E-84 | | | | |  |
| rs9927848 | | | | | A | | | C | | -1.22E-02 | | | 2.00E-03 | | | | | 6.40E-10 | | | | |  |
| rs9989419 | | | | | G | | | A | | -2.00E-03 | | | 1.70E-03 | | | | | 2.30E-01 | | | | |  |
| rs999889 | | | | | A | | | G | | -1.08E-02 | | | 1.90E-03 | | | | | 1.40E-08 | | | | |  |
| **DHEAS** | | | | |  | | |  | |  | | |  | | | | |  | | | | |  |
| rs10009336 | | | | | T | | | C | | 1.00E-02 | | | 1.00E-02 | | | | | 2.38E-01 | | | | |  |
| rs10027275 | | | | | C | | | G | | -2.00E-02 | | | 1.00E-02 | | | | | 8.82E-02 | | | | |  |
| rs10123811 | | | | | T | | | C | | -2.00E-02 | | | 1.00E-02 | | | | | 5.78E-02 | | | | |  |
| rs10132280 | | | | | A | | | C | | 1.00E-02 | | | 1.00E-02 | | | | | 4.84E-01 | | | | |  |
| rs10182181 | | | | | G | | | A | | -1.00E-02 | | | 1.00E-02 | | | | | 4.57E-01 | | | | |  |
| rs10197031 | | | | | C | | | T | | -1.00E-02 | | | 1.00E-02 | | | | | 2.48E-01 | | | | |  |
| rs10208512 | | | | | A | | | G | | -1.00E-02 | | | 2.00E-02 | | | | | 6.82E-01 | | | | |  |
| rs102275 | | | | | C | | | T | | 1.00E-02 | | | 1.00E-02 | | | | | 5.37E-01 | | | | |  |
| rs10238028 | | | | | G | | | A | | -2.00E-02 | | | 2.00E-02 | | | | | 2.50E-01 | | | | |  |
| rs10243319 | | | | | C | | | T | | 1.00E-02 | | | 1.00E-02 | | | | | 1.97E-01 | | | | |  |
| rs10245356 | | | | | T | | | C | | -1.00E-02 | | | 1.00E-02 | | | | | 5.46E-01 | | | | |  |
| rs10247983 | | | | | A | | | G | | -3.00E-02 | | | 2.00E-02 | | | | | 7.10E-02 | | | | |  |
| rs10248136 | | | | | T | | | C | | -1.00E-02 | | | 1.00E-02 | | | | | 1.15E-01 | | | | |  |
| rs10278546 | | | | | C | | | A | | -3.00E-02 | | | 1.00E-02 | | | | | 1.57E-02 | | | | |  |
| rs10468017 | | | | | T | | | C | | 1.00E-02 | | | 1.00E-02 | | | | | 5.07E-01 | | | | |  |
| rs10478110 | | | | | C | | | A | | -1.00E-02 | | | 1.00E-02 | | | | | 5.08E-01 | | | | |  |
| rs10742752 | | | | | C | | | T | | -1.00E-02 | | | 1.00E-02 | | | | | 1.51E-01 | | | | |  |
| rs10747488 | | | | | A | | | C | | -2.00E-02 | | | 1.00E-02 | | | | | 1.19E-01 | | | | |  |
| rs10768994 | | | | | C | | | T | | 1.00E-02 | | | 1.00E-02 | | | | | 4.90E-01 | | | | |  |
| rs10795422 | | | | | G | | | A | | 2.00E-02 | | | 1.00E-02 | | | | | 1.74E-02 | | | | |  |
| rs10808546 | | | | | T | | | C | | 1.00E-02 | | | 1.00E-02 | | | | | 4.38E-01 | | | | |  |
| rs10864070 | | | | | A | | | G | | -1.00E-02 | | | 1.00E-02 | | | | | 3.43E-01 | | | | |  |
| rs10867256 | | | | | T | | | C | | 1.00E-02 | | | 1.00E-02 | | | | | 1.42E-01 | | | | |  |
| rs10895276 | | | | | T | | | C | | -1.00E-02 | | | 1.00E-02 | | | | | 5.38E-01 | | | | |  |
| rs10920678 | | | | | G | | | A | | -2.00E-02 | | | 1.00E-02 | | | | | 6.25E-02 | | | | |  |
| rs10938397 | | | | | G | | | A | | 2.00E-02 | | | 2.00E-02 | | | | | 1.88E-01 | | | | |  |
| rs10942267 | | | | | G | | | A | | -1.00E-02 | | | 1.00E-02 | | | | | 5.18E-01 | | | | |  |
| rs10953740 | | | | | G | | | A | | 1.00E-02 | | | 1.00E-02 | | | | | 9.66E-02 | | | | |  |
| rs10968114 | | | | | C | | | A | | -1.00E-02 | | | 1.00E-02 | | | | | 3.68E-01 | | | | |  |
| rs10971709 | | | | | T | | | C | | -1.00E-02 | | | 1.00E-02 | | | | | 2.78E-01 | | | | |  |
| rs11030618 | | | | | T | | | C | | -1.00E-02 | | | 1.00E-02 | | | | | 4.68E-01 | | | | |  |
| rs1105977 | | | | | T | | | G | | 5.00E-02 | | | 1.00E-02 | | | | | 1.06E-08 | | | | |  |
| rs11084553 | | | | | G | | | A | | -1.00E-02 | | | 1.00E-02 | | | | | 5.90E-01 | | | | |  |
| rs11115176 | | | | | C | | | T | | 1.00E-02 | | | 1.00E-02 | | | | | 5.04E-01 | | | | |  |
| rs1112613 | | | | | A | | | G | | -1.00E-02 | | | 1.00E-02 | | | | | 5.18E-01 | | | | |  |
| rs11155787 | | | | | T | | | C | | 1.00E-02 | | | 1.00E-02 | | | | | 5.29E-01 | | | | |  |
| rs11165643 | | | | | T | | | C | | 1.00E-02 | | | 1.00E-02 | | | | | 5.08E-01 | | | | |  |
| rs11170468 | | | | | C | | | A | | 1.00E-02 | | | 1.00E-02 | | | | | 4.20E-01 | | | | |  |
| rs11251352 | | | | | G | | | A | | 1.00E-02 | | | 1.00E-02 | | | | | 4.33E-01 | | | | |  |
| rs1144387 | | | | | C | | | G | | 2.00E-02 | | | 1.00E-02 | | | | | 2.05E-02 | | | | |  |
| rs11505821 | | | | | T | | | A | | -1.00E-02 | | | 2.00E-02 | | | | | 4.21E-01 | | | | |  |
| rs11556924 | | | | | T | | | C | | 2.00E-02 | | | 1.00E-02 | | | | | 1.63E-02 | | | | |  |
| rs1158805 | | | | | A | | | C | | 2.00E-02 | | | 1.00E-02 | | | | | 3.15E-02 | | | | |  |
| rs11621792 | | | | | C | | | T | | 1.00E-02 | | | 1.00E-02 | | | | | 1.18E-01 | | | | |  |
| rs11640366 | | | | | A | | | C | | 1.00E-02 | | | 1.00E-02 | | | | | 5.03E-01 | | | | |  |
| rs11656076 | | | | | A | | | G | | 1.00E-02 | | | 1.00E-02 | | | | | 4.95E-01 | | | | |  |
| rs11713193 | | | | | A | | | G | | 1.00E-02 | | | 1.00E-02 | | | | | 3.31E-01 | | | | |  |
| rs11738695 | | | | | A | | | C | | -1.00E-02 | | | 1.00E-02 | | | | | 3.37E-01 | | | | |  |
| rs117589665 | | | | | G | | | A | | 4.00E-02 | | | 2.00E-02 | | | | | 8.51E-03 | | | | |  |
| rs11765639 | | | | | A | | | G | | -1.00E-02 | | | 1.00E-02 | | | | | 5.51E-01 | | | | |  |
| rs11855853 | | | | | T | | | C | | 1.00E-02 | | | 1.00E-02 | | | | | 5.78E-01 | | | | |  |
| rs1187352 | | | | | C | | | T | | -1.00E-02 | | | 1.00E-02 | | | | | 4.54E-01 | | | | |  |
| rs11880870 | | | | | G | | | A | | 1.00E-02 | | | 1.00E-02 | | | | | 4.73E-01 | | | | |  |
| rs12044597 | | | | | G | | | A | | 1.00E-02 | | | 1.00E-02 | | | | | 4.73E-01 | | | | |  |
| rs12049202 | | | | | T | | | C | | 1.00E-02 | | | 1.00E-02 | | | | | 2.60E-01 | | | | |  |
| rs12098284 | | | | | T | | | C | | 1.00E-02 | | | 1.00E-02 | | | | | 4.48E-01 | | | | |  |
| rs12150665 | | | | | C | | | T | | -1.00E-02 | | | 1.00E-02 | | | | | 4.19E-01 | | | | |  |
| rs1218822 | | | | | A | | | G | | -1.00E-02 | | | 1.00E-02 | | | | | 5.09E-01 | | | | |  |
| rs12299814 | | | | | A | | | C | | -1.00E-02 | | | 1.00E-02 | | | | | 1.61E-01 | | | | |  |
| rs12320328 | | | | | G | | | A | | 1.00E-02 | | | 2.00E-02 | | | | | 3.79E-01 | | | | |  |
| rs12364470 | | | | | G | | | T | | -1.00E-02 | | | 1.00E-02 | | | | | 4.96E-01 | | | | |  |
| rs1241986 | | | | | A | | | G | | -1.00E-02 | | | 1.00E-02 | | | | | 4.96E-01 | | | | |  |
| rs12429545 | | | | | A | | | G | | 1.00E-02 | | | 1.00E-02 | | | | | 6.22E-01 | | | | |  |
| rs12448257 | | | | | A | | | G | | 1.00E-02 | | | 1.00E-02 | | | | | 2.71E-01 | | | | |  |
| rs12543287 | | | | | C | | | G | | -1.00E-02 | | | 1.00E-02 | | | | | 2.15E-01 | | | | |  |
| rs12629015 | | | | | G | | | A | | 1.00E-02 | | | 1.00E-02 | | | | | 5.98E-01 | | | | |  |
| rs1266874 | | | | | G | | | A | | 1.00E-02 | | | 1.00E-02 | | | | | 3.14E-01 | | | | |  |
| rs12675063 | | | | | T | | | A | | 2.00E-02 | | | 1.00E-02 | | | | | 1.15E-01 | | | | |  |
| rs1268065 | | | | | A | | | G | | -2.00E-02 | | | 1.00E-02 | | | | | 5.88E-03 | | | | |  |
| rs12680842 | | | | | G | | | A | | 1.00E-02 | | | 1.00E-02 | | | | | 1.05E-01 | | | | |  |
| rs12696304 | | | | | G | | | C | | -1.00E-02 | | | 1.00E-02 | | | | | 5.39E-01 | | | | |  |
| rs12718572 | | | | | T | | | C | | -1.00E-02 | | | 1.00E-02 | | | | | 3.08E-01 | | | | |  |
| rs12762034 | | | | | C | | | T | | 2.00E-02 | | | 2.00E-02 | | | | | 1.50E-01 | | | | |  |
| rs12914489 | | | | | A | | | G | | 2.00E-02 | | | 1.00E-02 | | | | | 2.05E-01 | | | | |  |
| rs12939549 | | | | | G | | | A | | 1.00E-02 | | | 1.00E-02 | | | | | 3.81E-01 | | | | |  |
| rs1296328 | | | | | C | | | A | | -1.00E-02 | | | 1.00E-02 | | | | | 9.74E-02 | | | | |  |
| rs13021737 | | | | | G | | | A | | -1.00E-02 | | | 1.00E-02 | | | | | 2.14E-01 | | | | |  |
| rs13035806 | | | | | A | | | G | | -1.00E-02 | | | 1.00E-02 | | | | | 4.32E-01 | | | | |  |
| rs13047416 | | | | | G | | | C | | 1.00E-02 | | | 1.00E-02 | | | | | 2.69E-01 | | | | |  |
| rs13069244 | | | | | A | | | G | | -1.00E-02 | | | 2.00E-02 | | | | | 7.26E-01 | | | | |  |
| rs13094241 | | | | | G | | | T | | -1.00E-02 | | | 1.00E-02 | | | | | 3.91E-01 | | | | |  |
| rs13107325 | | | | | T | | | C | | 3.00E-02 | | | 2.00E-02 | | | | | 2.28E-02 | | | | |  |
| rs13110266 | | | | | A | | | G | | -1.00E-02 | | | 1.00E-02 | | | | | 4.35E-01 | | | | |  |
| rs13150068 | | | | | G | | | A | | 2.00E-02 | | | 1.00E-02 | | | | | 2.35E-02 | | | | |  |
| rs13174863 | | | | | G | | | A | | -2.00E-02 | | | 1.00E-02 | | | | | 1.95E-01 | | | | |  |
| rs13191362 | | | | | G | | | A | | -1.00E-02 | | | 1.00E-02 | | | | | 5.26E-01 | | | | |  |
| rs1320903 | | | | | A | | | G | | 1.00E-02 | | | 1.00E-02 | | | | | 4.32E-01 | | | | |  |
| rs1321432 | | | | | C | | | A | | -1.00E-02 | | | 1.00E-02 | | | | | 2.61E-01 | | | | |  |
| rs13251458 | | | | | A | | | G | | 1.00E-02 | | | 1.00E-02 | | | | | 4.87E-01 | | | | |  |
| rs1327259 | | | | | G | | | A | | 1.00E-02 | | | 1.00E-02 | | | | | 3.04E-01 | | | | |  |
| rs13389219 | | | | | T | | | C | | 1.00E-02 | | | 1.00E-02 | | | | | 3.96E-01 | | | | |  |
| rs1365466 | | | | | T | | | C | | 1.00E-02 | | | 1.00E-02 | | | | | 5.14E-01 | | | | |  |
| rs13702 | | | | | C | | | T | | -1.00E-02 | | | 1.00E-02 | | | | | 4.17E-01 | | | | |  |
| rs1371108 | | | | | A | | | C | | 1.00E-02 | | | 1.00E-02 | | | | | 5.40E-01 | | | | |  |
| rs1409818 | | | | | T | | | C | | 2.00E-02 | | | 1.00E-02 | | | | | 1.71E-01 | | | | |  |
| rs1412235 | | | | | C | | | G | | 1.00E-02 | | | 1.00E-02 | | | | | 4.92E-01 | | | | |  |
| rs1421334 | | | | | C | | | A | | -1.00E-02 | | | 1.00E-02 | | | | | 8.91E-02 | | | | |  |
| rs1452075 | | | | | T | | | C | | -1.00E-02 | | | 1.00E-02 | | | | | 5.62E-01 | | | | |  |
| rs1454687 | | | | | G | | | C | | -1.00E-02 | | | 1.00E-02 | | | | | 1.97E-01 | | | | |  |
| rs1465900 | | | | | C | | | A | | 1.00E-02 | | | 1.00E-02 | | | | | 3.01E-01 | | | | |  |
| rs1472169 | | | | | T | | | C | | 1.00E-02 | | | 1.00E-02 | | | | | 2.27E-01 | | | | |  |
| rs150449323 | | | | | C | | | T | | 1.00E-02 | | | 1.00E-02 | | | | | 4.56E-01 | | | | |  |
| rs1521527 | | | | | C | | | G | | 1.00E-02 | | | 1.00E-02 | | | | | 4.74E-01 | | | | |  |
| rs1528435 | | | | | T | | | C | | -2.00E-02 | | | 1.00E-02 | | | | | 6.30E-02 | | | | |  |
| rs1534696 | | | | | A | | | C | | 1.00E-02 | | | 1.00E-02 | | | | | 4.17E-01 | | | | |  |
| rs1561442 | | | | | A | | | G | | -1.00E-02 | | | 1.00E-02 | | | | | 5.13E-01 | | | | |  |
| rs157935 | | | | | G | | | T | | 1.00E-02 | | | 1.00E-02 | | | | | 2.01E-01 | | | | |  |
| rs1624134 | | | | | C | | | G | | 1.00E-02 | | | 1.00E-02 | | | | | 4.86E-01 | | | | |  |
| rs1640269 | | | | | C | | | A | | -1.00E-02 | | | 1.00E-02 | | | | | 4.15E-01 | | | | |  |
| rs1650548 | | | | | C | | | G | | 1.00E-02 | | | 1.00E-02 | | | | | 4.57E-01 | | | | |  |
| rs1656377 | | | | | C | | | T | | 1.00E-02 | | | 1.00E-02 | | | | | 3.14E-01 | | | | |  |
| rs1681740 | | | | | C | | | A | | 1.00E-02 | | | 1.00E-02 | | | | | 1.80E-01 | | | | |  |
| rs16849710 | | | | | G | | | A | | -2.00E-02 | | | 1.00E-02 | | | | | 2.54E-02 | | | | |  |
| rs16851483 | | | | | T | | | G | | -1.00E-02 | | | 2.00E-02 | | | | | 5.87E-01 | | | | |  |
| rs16903285 | | | | | C | | | T | | 1.00E-02 | | | 1.00E-02 | | | | | 4.76E-01 | | | | |  |
| rs16942887 | | | | | A | | | G | | -1.00E-02 | | | 1.00E-02 | | | | | 5.25E-01 | | | | |  |
| rs17014375 | | | | | G | | | T | | -1.00E-02 | | | 1.00E-02 | | | | | 3.62E-01 | | | | |  |
| rs17033117 | | | | | T | | | C | | -1.00E-02 | | | 1.00E-02 | | | | | 2.40E-01 | | | | |  |
| rs17056301 | | | | | C | | | T | | -1.00E-02 | | | 1.00E-02 | | | | | 2.64E-01 | | | | |  |
| rs17119937 | | | | | C | | | T | | -2.00E-02 | | | 2.00E-02 | | | | | 2.57E-01 | | | | |  |
| rs17184382 | | | | | C | | | A | | 2.00E-02 | | | 1.00E-02 | | | | | 4.99E-02 | | | | |  |
| rs17207196 | | | | | T | | | C | | -2.00E-02 | | | 1.00E-02 | | | | | 2.22E-02 | | | | |  |
| rs1730862 | | | | | A | | | G | | -1.00E-02 | | | 1.00E-02 | | | | | 2.34E-01 | | | | |  |
| rs17399237 | | | | | C | | | T | | -1.00E-02 | | | 1.00E-02 | | | | | 4.81E-01 | | | | |  |
| rs17425707 | | | | | C | | | T | | 1.00E-02 | | | 1.00E-02 | | | | | 4.75E-01 | | | | |  |
| rs17535749 | | | | | A | | | G | | -2.00E-02 | | | 1.00E-02 | | | | | 7.57E-02 | | | | |  |
| rs17551974 | | | | | A | | | C | | -1.00E-02 | | | 1.00E-02 | | | | | 2.52E-01 | | | | |  |
| rs1772189 | | | | | A | | | T | | 2.00E-02 | | | 1.00E-02 | | | | | 3.38E-03 | | | | |  |
| rs17789218 | | | | | C | | | T | | 1.00E-02 | | | 1.00E-02 | | | | | 1.31E-01 | | | | |  |
| rs1801282 | | | | | G | | | C | | -2.00E-02 | | | 1.00E-02 | | | | | 1.26E-01 | | | | |  |
| rs1863652 | | | | | A | | | G | | 1.00E-02 | | | 1.00E-02 | | | | | 4.55E-01 | | | | |  |
| rs1866956 | | | | | T | | | C | | -2.00E-02 | | | 1.00E-02 | | | | | 1.44E-02 | | | | |  |
| rs1883025 | | | | | T | | | C | | 1.00E-02 | | | 1.00E-02 | | | | | 3.96E-01 | | | | |  |
| rs1891216 | | | | | G | | | T | | -1.00E-02 | | | 1.00E-02 | | | | | 2.29E-01 | | | | |  |
| rs189595752 | | | | | G | | | A | | 1.00E-02 | | | 1.00E-02 | | | | | 6.35E-01 | | | | |  |
| rs1928295 | | | | | C | | | T | | 1.00E-02 | | | 1.00E-02 | | | | | 1.99E-01 | | | | |  |
| rs1982725 | | | | | T | | | C | | 1.00E-02 | | | 1.00E-02 | | | | | 2.05E-01 | | | | |  |
| rs200810 | | | | | C | | | T | | 2.00E-02 | | | 1.00E-02 | | | | | 5.54E-02 | | | | |  |
| rs2009416 | | | | | T | | | C | | -1.00E-02 | | | 1.00E-02 | | | | | 5.45E-01 | | | | |  |
| rs2065418 | | | | | G | | | T | | 1.00E-02 | | | 1.00E-02 | | | | | 4.62E-01 | | | | |  |
| rs215634 | | | | | G | | | A | | -1.00E-02 | | | 1.00E-02 | | | | | 5.50E-01 | | | | |  |
| rs2174307 | | | | | C | | | G | | -1.00E-02 | | | 1.00E-02 | | | | | 5.33E-01 | | | | |  |
| rs217671 | | | | | G | | | A | | -1.00E-02 | | | 1.00E-02 | | | | | 4.74E-01 | | | | |  |
| rs2224585 | | | | | A | | | G | | 1.00E-02 | | | 1.00E-02 | | | | | 4.73E-01 | | | | |  |
| rs2241210 | | | | | G | | | A | | 1.00E-02 | | | 1.00E-02 | | | | | 2.10E-01 | | | | |  |
| rs2246012 | | | | | C | | | T | | -1.00E-02 | | | 1.00E-02 | | | | | 5.78E-01 | | | | |  |
| rs2325036 | | | | | C | | | A | | 1.00E-02 | | | 1.00E-02 | | | | | 3.28E-01 | | | | |  |
| rs2351958 | | | | | A | | | C | | -1.00E-02 | | | 1.00E-02 | | | | | 1.72E-01 | | | | |  |
| rs2357760 | | | | | A | | | G | | 1.00E-02 | | | 1.00E-02 | | | | | 1.46E-01 | | | | |  |
| rs2393791 | | | | | T | | | C | | -4.00E-02 | | | 1.00E-02 | | | | | 5.25E-05 | | | | |  |
| rs2479958 | | | | | G | | | A | | 2.00E-02 | | | 1.00E-02 | | | | | 3.12E-02 | | | | |  |
| rs2481665 | | | | | C | | | T | | 2.00E-02 | | | 1.00E-02 | | | | | 7.36E-02 | | | | |  |
| rs2498786 | | | | | G | | | C | | -1.00E-02 | | | 1.00E-02 | | | | | 5.41E-01 | | | | |  |
| rs2543132 | | | | | C | | | G | | 1.00E-02 | | | 1.00E-02 | | | | | 4.77E-01 | | | | |  |
| rs2551644 | | | | | A | | | T | | 1.00E-02 | | | 1.00E-02 | | | | | 6.21E-01 | | | | |  |
| rs2608703 | | | | | A | | | C | | 1.00E-02 | | | 1.00E-02 | | | | | 5.32E-01 | | | | |  |
| rs2612038 | | | | | T | | | C | | -3.00E-02 | | | 1.00E-02 | | | | | 3.54E-02 | | | | |  |
| rs2642438 | | | | | G | | | A | | -1.00E-02 | | | 1.00E-02 | | | | | 2.84E-01 | | | | |  |
| rs2643452 | | | | | A | | | T | | 1.00E-02 | | | 1.00E-02 | | | | | 2.31E-01 | | | | |  |
| rs2693826 | | | | | A | | | G | | -1.00E-02 | | | 1.00E-02 | | | | | 5.40E-01 | | | | |  |
| rs2694047 | | | | | G | | | A | | 1.00E-02 | | | 1.00E-02 | | | | | 5.31E-01 | | | | |  |
| rs273504 | | | | | G | | | A | | -1.00E-02 | | | 1.00E-02 | | | | | 2.10E-01 | | | | |  |
| rs2744974 | | | | | T | | | C | | 1.00E-02 | | | 1.00E-02 | | | | | 3.97E-01 | | | | |  |
| rs2791644 | | | | | C | | | T | | -1.00E-02 | | | 1.00E-02 | | | | | 3.47E-01 | | | | |  |
| rs28507491 | | | | | A | | | G | | -2.00E-02 | | | 1.00E-02 | | | | | 6.25E-02 | | | | |  |
| rs2861683 | | | | | C | | | A | | -1.00E-02 | | | 1.00E-02 | | | | | 3.14E-01 | | | | |  |
| rs2868975 | | | | | A | | | G | | 1.00E-02 | | | 1.00E-02 | | | | | 2.09E-01 | | | | |  |
| rs2875762 | | | | | C | | | G | | 1.00E-02 | | | 1.00E-02 | | | | | 4.52E-01 | | | | |  |
| rs2931434 | | | | | T | | | C | | 1.00E-02 | | | 1.00E-02 | | | | | 2.40E-01 | | | | |  |
| rs2972145 | | | | | C | | | T | | -1.00E-02 | | | 1.00E-02 | | | | | 2.31E-01 | | | | |  |
| rs3001032 | | | | | C | | | T | | -1.00E-02 | | | 1.00E-02 | | | | | 1.09E-01 | | | | |  |
| rs34255979 | | | | | T | | | C | | 2.00E-02 | | | 1.00E-02 | | | | | 1.38E-01 | | | | |  |
| rs349088 | | | | | A | | | C | | -1.00E-02 | | | 1.00E-02 | | | | | 3.46E-01 | | | | |  |
| rs35350976 | | | | | G | | | A | | -1.00E-02 | | | 1.00E-02 | | | | | 3.37E-01 | | | | |  |
| rs355777 | | | | | C | | | G | | -1.00E-02 | | | 1.00E-02 | | | | | 5.12E-01 | | | | |  |
| rs3749897 | | | | | T | | | C | | 1.00E-02 | | | 1.00E-02 | | | | | 3.91E-01 | | | | |  |
| rs3754963 | | | | | T | | | A | | 1.00E-02 | | | 1.00E-02 | | | | | 6.02E-01 | | | | |  |
| rs3772882 | | | | | A | | | C | | 2.00E-02 | | | 1.00E-02 | | | | | 4.08E-02 | | | | |  |
| rs3800229 | | | | | T | | | G | | -1.00E-02 | | | 1.00E-02 | | | | | 1.30E-01 | | | | |  |
| rs3806114 | | | | | A | | | G | | 1.00E-02 | | | 1.00E-02 | | | | | 4.00E-01 | | | | |  |
| rs3806572 | | | | | A | | | G | | -1.00E-02 | | | 1.00E-02 | | | | | 3.92E-01 | | | | |  |
| rs3807645 | | | | | A | | | G | | -1.00E-02 | | | 1.00E-02 | | | | | 2.96E-01 | | | | |  |
| rs380857 | | | | | A | | | C | | 3.00E-02 | | | 1.00E-02 | | | | | 2.79E-02 | | | | |  |
| rs3810027 | | | | | G | | | C | | 1.00E-02 | | | 1.00E-02 | | | | | 2.36E-01 | | | | |  |
| rs3829639 | | | | | G | | | A | | 1.00E-02 | | | 1.00E-02 | | | | | 5.58E-01 | | | | |  |
| rs38314 | | | | | A | | | G | | -1.00E-02 | | | 1.00E-02 | | | | | 2.86E-01 | | | | |  |
| rs3902951 | | | | | G | | | T | | -2.00E-02 | | | 1.00E-02 | | | | | 2.66E-02 | | | | |  |
| rs3904244 | | | | | A | | | T | | -1.00E-02 | | | 1.00E-02 | | | | | 4.96E-01 | | | | |  |
| rs40067 | | | | | A | | | G | | -1.00E-02 | | | 1.00E-02 | | | | | 5.18E-01 | | | | |  |
| rs40270 | | | | | C | | | A | | 1.00E-02 | | | 1.00E-02 | | | | | 2.10E-01 | | | | |  |
| rs4072917 | | | | | A | | | G | | 1.00E-02 | | | 1.00E-02 | | | | | 1.91E-01 | | | | |  |
| rs4148005 | | | | | G | | | T | | -1.00E-02 | | | 1.00E-02 | | | | | 1.81E-01 | | | | |  |
| rs4148155 | | | | | G | | | A | | 2.00E-02 | | | 1.00E-02 | | | | | 1.61E-01 | | | | |  |
| rs4237643 | | | | | G | | | T | | 1.00E-02 | | | 1.00E-02 | | | | | 1.55E-01 | | | | |  |
| rs4240624 | | | | | A | | | G | | -2.00E-02 | | | 1.00E-02 | | | | | 9.70E-02 | | | | |  |
| rs427943 | | | | | C | | | A | | 1.00E-02 | | | 1.00E-02 | | | | | 2.11E-01 | | | | |  |
| rs4307239 | | | | | G | | | A | | -1.00E-02 | | | 1.00E-02 | | | | | 4.77E-01 | | | | |  |
| rs4338849 | | | | | A | | | G | | -2.00E-02 | | | 1.00E-02 | | | | | 6.14E-03 | | | | |  |
| rs4430672 | | | | | C | | | T | | 1.00E-02 | | | 1.00E-02 | | | | | 2.65E-01 | | | | |  |
| rs4556997 | | | | | A | | | C | | -1.00E-02 | | | 1.00E-02 | | | | | 2.13E-01 | | | | |  |
| rs4567095 | | | | | T | | | C | | -1.00E-02 | | | 1.00E-02 | | | | | 3.08E-01 | | | | |  |
| rs4568281 | | | | | A | | | G | | 1.00E-02 | | | 1.00E-02 | | | | | 1.44E-01 | | | | |  |
| rs4589691 | | | | | G | | | C | | -1.00E-02 | | | 1.00E-02 | | | | | 3.42E-01 | | | | |  |
| rs4639527 | | | | | G | | | A | | 1.00E-02 | | | 1.00E-02 | | | | | 3.39E-01 | | | | |  |
| rs4639796 | | | | | A | | | G | | -1.00E-02 | | | 1.00E-02 | | | | | 3.59E-01 | | | | |  |
| rs4660443 | | | | | T | | | C | | 1.00E-02 | | | 1.00E-02 | | | | | 4.51E-01 | | | | |  |
| rs4665972 | | | | | C | | | T | | 1.00E-02 | | | 1.00E-02 | | | | | 1.66E-01 | | | | |  |
| rs4671328 | | | | | G | | | T | | 1.00E-02 | | | 1.00E-02 | | | | | 3.63E-01 | | | | |  |
| rs4674669 | | | | | T | | | C | | -1.00E-02 | | | 1.00E-02 | | | | | 6.62E-01 | | | | |  |
| rs4740619 | | | | | C | | | T | | 1.00E-02 | | | 1.00E-02 | | | | | 5.80E-01 | | | | |  |
| rs4800191 | | | | | C | | | G | | -1.00E-02 | | | 1.00E-02 | | | | | 2.00E-01 | | | | |  |
| rs4804414 | | | | | T | | | C | | -1.00E-02 | | | 1.00E-02 | | | | | 4.37E-01 | | | | |  |
| rs4812336 | | | | | A | | | G | | -1.00E-02 | | | 1.00E-02 | | | | | 2.83E-01 | | | | |  |
| rs4813619 | | | | | T | | | G | | -1.00E-02 | | | 1.00E-02 | | | | | 4.93E-01 | | | | |  |
| rs4820091 | | | | | G | | | T | | -1.00E-02 | | | 1.00E-02 | | | | | 3.31E-01 | | | | |  |
| rs4820408 | | | | | G | | | T | | -1.00E-02 | | | 1.00E-02 | | | | | 1.71E-01 | | | | |  |
| rs4842491 | | | | | T | | | C | | 1.00E-02 | | | 1.00E-02 | | | | | 3.64E-01 | | | | |  |
| rs4846914 | | | | | A | | | G | | -1.00E-02 | | | 1.00E-02 | | | | | 2.60E-01 | | | | |  |
| rs4851029 | | | | | G | | | T | | 1.00E-02 | | | 1.00E-02 | | | | | 5.33E-01 | | | | |  |
| rs4880341 | | | | | T | | | C | | 1.00E-02 | | | 1.00E-02 | | | | | 4.44E-01 | | | | |  |
| rs4936175 | | | | | C | | | T | | -1.00E-02 | | | 1.00E-02 | | | | | 2.03E-01 | | | | |  |
| rs4939883 | | | | | C | | | T | | 2.00E-02 | | | 1.00E-02 | | | | | 6.89E-02 | | | | |  |
| rs4954638 | | | | | C | | | A | | 1.00E-02 | | | 1.00E-02 | | | | | 3.08E-01 | | | | |  |
| rs538579 | | | | | C | | | G | | -1.00E-02 | | | 1.00E-02 | | | | | 2.55E-01 | | | | |  |
| rs543874 | | | | | G | | | A | | 1.00E-02 | | | 1.00E-02 | | | | | 5.44E-01 | | | | |  |
| rs559231 | | | | | T | | | G | | 1.00E-02 | | | 1.00E-02 | | | | | 5.15E-01 | | | | |  |
| rs56024084 | | | | | T | | | C | | -1.00E-02 | | | 1.00E-02 | | | | | 1.98E-01 | | | | |  |
| rs56077345 | | | | | C | | | G | | 1.00E-02 | | | 2.00E-02 | | | | | 6.70E-01 | | | | |  |
| rs587252 | | | | | C | | | A | | 2.00E-02 | | | 1.00E-02 | | | | | 1.30E-01 | | | | |  |
| rs61856602 | | | | | G | | | A | | -1.00E-02 | | | 1.00E-02 | | | | | 3.92E-01 | | | | |  |
| rs6235 | | | | | G | | | C | | -1.00E-02 | | | 1.00E-02 | | | | | 4.92E-01 | | | | |  |
| rs62623385 | | | | | T | | | A | | -2.00E-02 | | | 2.00E-02 | | | | | 3.81E-01 | | | | |  |
| rs6265 | | | | | T | | | C | | 2.00E-02 | | | 1.00E-02 | | | | | 4.92E-02 | | | | |  |
| rs633695 | | | | | G | | | A | | 1.00E-02 | | | 1.00E-02 | | | | | 4.10E-01 | | | | |  |
| rs6471941 | | | | | A | | | G | | -1.00E-02 | | | 1.00E-02 | | | | | 2.28E-01 | | | | |  |
| rs6486122 | | | | | T | | | C | | -1.00E-02 | | | 1.00E-02 | | | | | 3.93E-01 | | | | |  |
| rs6512302 | | | | | C | | | G | | 2.00E-02 | | | 1.00E-02 | | | | | 1.95E-02 | | | | |  |
| rs6545714 | | | | | A | | | G | | 1.00E-02 | | | 1.00E-02 | | | | | 1.73E-01 | | | | |  |
| rs6561943 | | | | | T | | | C | | -1.00E-02 | | | 1.00E-02 | | | | | 4.19E-01 | | | | |  |
| rs657452 | | | | | G | | | A | | 1.00E-02 | | | 1.00E-02 | | | | | 4.07E-01 | | | | |  |
| rs6591407 | | | | | A | | | C | | -1.00E-02 | | | 1.00E-02 | | | | | 4.87E-01 | | | | |  |
| rs6595205 | | | | | G | | | C | | 1.00E-02 | | | 1.00E-02 | | | | | 5.14E-01 | | | | |  |
| rs663129 | | | | | A | | | G | | 1.00E-02 | | | 1.00E-02 | | | | | 5.28E-01 | | | | |  |
| rs668871 | | | | | T | | | C | | -1.00E-02 | | | 1.00E-02 | | | | | 4.61E-01 | | | | |  |
| rs6758199 | | | | | T | | | C | | 1.00E-02 | | | 2.00E-02 | | | | | 5.72E-01 | | | | |  |
| rs6785245 | | | | | C | | | T | | 1.00E-02 | | | 1.00E-02 | | | | | 1.68E-01 | | | | |  |
| rs6815910 | | | | | A | | | T | | 2.00E-02 | | | 1.00E-02 | | | | | 1.50E-02 | | | | |  |
| rs6841761 | | | | | T | | | G | | -1.00E-02 | | | 1.00E-02 | | | | | 5.28E-01 | | | | |  |
| rs6860245 | | | | | C | | | G | | 1.00E-02 | | | 1.00E-02 | | | | | 2.03E-01 | | | | |  |
| rs686030 | | | | | A | | | C | | 1.00E-02 | | | 1.00E-02 | | | | | 3.69E-01 | | | | |  |
| rs695272 | | | | | C | | | T | | -1.00E-02 | | | 1.00E-02 | | | | | 2.77E-01 | | | | |  |
| rs7015 | | | | | A | | | G | | -1.00E-02 | | | 1.00E-02 | | | | | 2.00E-01 | | | | |  |
| rs7025938 | | | | | G | | | C | | -1.00E-02 | | | 1.00E-02 | | | | | 1.68E-01 | | | | |  |
| rs705704 | | | | | A | | | G | | -1.00E-02 | | | 1.00E-02 | | | | | 1.98E-01 | | | | |  |
| rs7084454 | | | | | A | | | G | | 1.00E-02 | | | 1.00E-02 | | | | | 5.62E-01 | | | | |  |
| rs7096764 | | | | | A | | | G | | 1.00E-02 | | | 1.00E-02 | | | | | 5.18E-01 | | | | |  |
| rs7117238 | | | | | A | | | G | | 1.00E-02 | | | 1.00E-02 | | | | | 4.33E-01 | | | | |  |
| rs7138803 | | | | | A | | | G | | 1.00E-02 | | | 1.00E-02 | | | | | 5.16E-01 | | | | |  |
| rs7144011 | | | | | T | | | G | | 1.00E-02 | | | 1.00E-02 | | | | | 3.55E-01 | | | | |  |
| rs7148846 | | | | | G | | | T | | -1.00E-02 | | | 1.00E-02 | | | | | 2.32E-01 | | | | |  |
| rs7196720 | | | | | C | | | T | | -1.00E-02 | | | 1.00E-02 | | | | | 5.16E-01 | | | | |  |
| rs7222349 | | | | | A | | | G | | -1.00E-02 | | | 1.00E-02 | | | | | 4.92E-01 | | | | |  |
| rs7239575 | | | | | C | | | T | | -1.00E-02 | | | 1.00E-02 | | | | | 4.99E-01 | | | | |  |
| rs7262150 | | | | | C | | | T | | 1.00E-02 | | | 1.00E-02 | | | | | 3.29E-01 | | | | |  |
| rs73079476 | | | | | C | | | A | | 6.00E-02 | | | 1.00E-02 | | | | | 2.52E-07 | | | | |  |
| rs7314285 | | | | | G | | | T | | -1.00E-02 | | | 2.00E-02 | | | | | 5.89E-01 | | | | |  |
| rs7318817 | | | | | T | | | C | | -1.00E-02 | | | 1.00E-02 | | | | | 1.02E-01 | | | | |  |
| rs738409 | | | | | G | | | C | | 1.00E-02 | | | 1.00E-02 | | | | | 4.90E-01 | | | | |  |
| rs7451021 | | | | | C | | | T | | 1.00E-02 | | | 1.00E-02 | | | | | 5.28E-01 | | | | |  |
| rs7498665 | | | | | G | | | A | | 1.00E-02 | | | 1.00E-02 | | | | | 2.45E-01 | | | | |  |
| rs7557796 | | | | | C | | | T | | -1.00E-02 | | | 1.00E-02 | | | | | 4.48E-01 | | | | |  |
| rs757869 | | | | | G | | | A | | 1.00E-02 | | | 1.00E-02 | | | | | 3.67E-01 | | | | |  |
| rs7615297 | | | | | G | | | C | | 2.00E-02 | | | 1.00E-02 | | | | | 1.80E-01 | | | | |  |
| rs7626079 | | | | | T | | | C | | -1.00E-02 | | | 1.00E-02 | | | | | 4.22E-01 | | | | |  |
| rs7678138 | | | | | A | | | G | | 1.00E-02 | | | 1.00E-02 | | | | | 3.78E-01 | | | | |  |
| rs7683836 | | | | | A | | | G | | -1.00E-02 | | | 1.00E-02 | | | | | 1.62E-01 | | | | |  |
| rs7703576 | | | | | C | | | T | | -1.00E-02 | | | 1.00E-02 | | | | | 4.87E-01 | | | | |  |
| rs7715256 | | | | | T | | | G | | 1.00E-02 | | | 1.00E-02 | | | | | 5.24E-01 | | | | |  |
| rs7724675 | | | | | A | | | G | | -1.00E-02 | | | 1.00E-02 | | | | | 5.33E-01 | | | | |  |
| rs7730898 | | | | | A | | | G | | 1.00E-02 | | | 1.00E-02 | | | | | 1.51E-01 | | | | |  |
| rs7761673 | | | | | A | | | T | | 1.00E-02 | | | 1.00E-02 | | | | | 3.58E-01 | | | | |  |
| rs7780752 | | | | | C | | | T | | 1.00E-02 | | | 1.00E-02 | | | | | 5.24E-01 | | | | |  |
| rs7788008 | | | | | A | | | G | | 1.00E-02 | | | 1.00E-02 | | | | | 3.15E-01 | | | | |  |
| rs7819514 | | | | | A | | | G | | -1.00E-02 | | | 1.00E-02 | | | | | 5.47E-01 | | | | |  |
| rs7844647 | | | | | C | | | T | | 1.00E-02 | | | 1.00E-02 | | | | | 2.54E-01 | | | | |  |
| rs7860634 | | | | | A | | | G | | 1.00E-02 | | | 1.00E-02 | | | | | 4.49E-01 | | | | |  |
| rs7869771 | | | | | C | | | A | | 1.00E-02 | | | 1.00E-02 | | | | | 1.72E-01 | | | | |  |
| rs7925214 | | | | | T | | | C | | -1.00E-02 | | | 1.00E-02 | | | | | 2.32E-01 | | | | |  |
| rs796004 | | | | | T | | | C | | -1.00E-02 | | | 1.00E-02 | | | | | 3.29E-01 | | | | |  |
| rs79717793 | | | | | A | | | G | | 1.00E-02 | | | 1.00E-02 | | | | | 4.79E-01 | | | | |  |
| rs7983065 | | | | | T | | | C | | -1.00E-02 | | | 1.00E-02 | | | | | 3.72E-01 | | | | |  |
| rs802685 | | | | | C | | | T | | 1.00E-02 | | | 1.00E-02 | | | | | 4.56E-01 | | | | |  |
| rs8033077 | | | | | C | | | T | | 1.00E-02 | | | 1.00E-02 | | | | | 3.50E-01 | | | | |  |
| rs8047395 | | | | | A | | | G | | 1.00E-02 | | | 1.00E-02 | | | | | 1.42E-01 | | | | |  |
| rs806600 | | | | | G | | | A | | -1.00E-02 | | | 1.00E-02 | | | | | 1.07E-01 | | | | |  |
| rs8079418 | | | | | C | | | T | | 1.00E-02 | | | 1.00E-02 | | | | | 5.47E-01 | | | | |  |
| rs8097672 | | | | | T | | | A | | -1.00E-02 | | | 1.00E-02 | | | | | 3.10E-01 | | | | |  |
| rs8097783 | | | | | A | | | G | | 3.00E-02 | | | 2.00E-02 | | | | | 9.56E-02 | | | | |  |
| rs8181823 | | | | | C | | | A | | 1.00E-02 | | | 1.00E-02 | | | | | 2.56E-01 | | | | |  |
| rs865809 | | | | | G | | | A | | 1.00E-02 | | | 1.00E-02 | | | | | 5.08E-01 | | | | |  |
| rs872281 | | | | | T | | | C | | -2.00E-02 | | | 1.00E-02 | | | | | 8.50E-02 | | | | |  |
| rs876605 | | | | | G | | | A | | 1.00E-02 | | | 1.00E-02 | | | | | 3.44E-01 | | | | |  |
| rs879620 | | | | | T | | | C | | -1.00E-02 | | | 1.00E-02 | | | | | 4.36E-01 | | | | |  |
| rs889398 | | | | | T | | | C | | 1.00E-02 | | | 1.00E-02 | | | | | 2.82E-01 | | | | |  |
| rs901630 | | | | | T | | | C | | 1.00E-02 | | | 1.00E-02 | | | | | 5.06E-01 | | | | |  |
| rs902695 | | | | | A | | | G | | 1.00E-02 | | | 1.00E-02 | | | | | 4.56E-01 | | | | |  |
| rs9267551 | | | | | G | | | C | | 1.00E-02 | | | 1.00E-02 | | | | | 7.12E-01 | | | | |  |
| rs9294260 | | | | | A | | | G | | 1.00E-02 | | | 1.00E-02 | | | | | 1.46E-01 | | | | |  |
| rs9297994 | | | | | A | | | G | | -1.00E-02 | | | 1.00E-02 | | | | | 5.67E-01 | | | | |  |
| rs9300422 | | | | | G | | | A | | -1.00E-02 | | | 1.00E-02 | | | | | 3.21E-01 | | | | |  |
| rs9379084 | | | | | A | | | G | | 1.00E-02 | | | 1.00E-02 | | | | | 2.48E-01 | | | | |  |
| rs9408882 | | | | | A | | | G | | 1.00E-02 | | | 1.00E-02 | | | | | 1.12E-01 | | | | |  |
| rs946824 | | | | | C | | | T | | 2.00E-02 | | | 1.00E-02 | | | | | 1.53E-01 | | | | |  |
| rs947612 | | | | | A | | | G | | -1.00E-02 | | | 1.00E-02 | | | | | 1.20E-01 | | | | |  |
| rs9522285 | | | | | A | | | G | | -1.00E-02 | | | 1.00E-02 | | | | | 1.04E-01 | | | | |  |
| rs9538162 | | | | | C | | | T | | -2.00E-02 | | | 1.00E-02 | | | | | 2.19E-02 | | | | |  |
| rs9547153 | | | | | G | | | A | | -1.00E-02 | | | 1.00E-02 | | | | | 4.18E-01 | | | | |  |
| rs9571687 | | | | | A | | | C | | -1.00E-02 | | | 1.00E-02 | | | | | 3.11E-01 | | | | |  |
| rs9615905 | | | | | T | | | C | | -1.00E-02 | | | 1.00E-02 | | | | | 2.48E-01 | | | | |  |
| rs964184 | | | | | C | | | G | | -2.00E-02 | | | 1.00E-02 | | | | | 1.80E-01 | | | | |  |
| rs970548 | | | | | C | | | A | | -1.00E-02 | | | 1.00E-02 | | | | | 5.00E-01 | | | | |  |
| rs9739640 | | | | | G | | | A | | -2.00E-02 | | | 1.00E-02 | | | | | 9.52E-02 | | | | |  |
| rs977747 | | | | | G | | | T | | -1.00E-02 | | | 1.00E-02 | | | | | 1.73E-01 | | | | |  |
| rs9783858 | | | | | T | | | C | | 2.00E-02 | | | 1.00E-02 | | | | | 1.14E-02 | | | | |  |
| rs9806742 | | | | | A | | | G | | 1.00E-02 | | | 1.00E-02 | | | | | 6.36E-01 | | | | |  |
| rs9816226 | | | | | T | | | A | | -2.00E-02 | | | 1.00E-02 | | | | | 6.78E-02 | | | | |  |
| rs9845966 | | | | | G | | | T | | 1.00E-02 | | | 1.00E-02 | | | | | 5.27E-01 | | | | |  |
| rs9849171 | | | | | C | | | G | | -1.00E-02 | | | 1.00E-02 | | | | | 1.34E-01 | | | | |  |
| rs987237 | | | | | G | | | A | | -2.00E-02 | | | 1.00E-02 | | | | | 1.04E-01 | | | | |  |
| rs9927848 | | | | | A | | | C | | -1.00E-02 | | | 1.00E-02 | | | | | 5.72E-01 | | | | |  |
| rs9989419 | | | | | G | | | A | | 1.00E-02 | | | 1.00E-02 | | | | | 3.94E-01 | | | | |  |
| rs999889 | | | | | A | | | G | | -2.00E-02 | | | 1.00E-02 | | | | | 9.71E-02 | | | | |  |
| **Estradiol levels** | | | | |  | | |  | |  | | |  | | | | |  | | | | |  |
| rs10009336 | | | | | T | | | C | | -1.31E-03 | | | 1.15E-03 | | | | | 2.60E-01 | | | | |  |
| rs10027275 | | | | | C | | | G | | -8.20E-06 | | | 9.74E-04 | | | | | 1.00E+00 | | | | |  |
| rs10123811 | | | | | T | | | C | | 8.06E-04 | | | 8.56E-04 | | | | | 3.40E-01 | | | | |  |
| rs10132280 | | | | | A | | | C | | -8.85E-04 | | | 9.27E-04 | | | | | 3.20E-01 | | | | |  |
| rs10182181 | | | | | G | | | A | | -7.24E-05 | | | 8.46E-04 | | | | | 9.30E-01 | | | | |  |
| rs10197031 | | | | | C | | | T | | -6.79E-04 | | | 9.35E-04 | | | | | 5.00E-01 | | | | |  |
| rs10208512 | | | | | A | | | G | | -5.82E-04 | | | 2.09E-03 | | | | | 7.80E-01 | | | | |  |
| rs102275 | | | | | C | | | T | | -3.55E-03 | | | 8.85E-04 | | | | | 4.80E-05 | | | | |  |
| rs10238028 | | | | | G | | | A | | -1.41E-03 | | | 1.70E-03 | | | | | 4.20E-01 | | | | |  |
| rs10243319 | | | | | C | | | T | | -2.18E-03 | | | 8.66E-04 | | | | | 1.10E-02 | | | | |  |
| rs10245356 | | | | | T | | | C | | -9.55E-04 | | | 8.75E-04 | | | | | 2.50E-01 | | | | |  |
| rs10247983 | | | | | A | | | G | | -2.62E-04 | | | 1.61E-03 | | | | | 8.60E-01 | | | | |  |
| rs10248136 | | | | | T | | | C | | -1.26E-03 | | | 8.48E-04 | | | | | 1.30E-01 | | | | |  |
| rs10278546 | | | | | C | | | A | | 1.50E-03 | | | 1.07E-03 | | | | | 1.70E-01 | | | | |  |
| rs10468017 | | | | | T | | | C | | -7.39E-04 | | | 9.26E-04 | | | | | 4.30E-01 | | | | |  |
| rs10478110 | | | | | C | | | A | | 1.11E-03 | | | 8.55E-04 | | | | | 1.90E-01 | | | | |  |
| rs10742752 | | | | | C | | | T | | -4.34E-04 | | | 8.67E-04 | | | | | 5.90E-01 | | | | |  |
| rs10747488 | | | | | A | | | C | | -5.08E-04 | | | 1.00E-03 | | | | | 5.60E-01 | | | | |  |
| rs10768994 | | | | | C | | | T | | -7.65E-04 | | | 8.56E-04 | | | | | 3.70E-01 | | | | |  |
| rs10795422 | | | | | G | | | A | | -5.45E-05 | | | 9.13E-04 | | | | | 9.50E-01 | | | | |  |
| rs10808546 | | | | | T | | | C | | 3.41E-03 | | | 8.54E-04 | | | | | 6.70E-05 | | | | |  |
| rs10864070 | | | | | A | | | G | | -1.90E-03 | | | 1.48E-03 | | | | | 2.20E-01 | | | | |  |
| rs10867256 | | | | | T | | | C | | 2.09E-04 | | | 8.51E-04 | | | | | 7.70E-01 | | | | |  |
| rs10895276 | | | | | T | | | C | | -7.26E-04 | | | 8.94E-04 | | | | | 3.90E-01 | | | | |  |
| rs10920678 | | | | | G | | | A | | 5.54E-05 | | | 8.53E-04 | | | | | 9.20E-01 | | | | |  |
| rs10938397 | | | | | G | | | A | | 6.59E-04 | | | 8.54E-04 | | | | | 4.00E-01 | | | | |  |
| rs10942267 | | | | | G | | | A | | -1.58E-03 | | | 9.07E-04 | | | | | 8.00E-02 | | | | |  |
| rs10953740 | | | | | G | | | A | | -5.53E-04 | | | 8.50E-04 | | | | | 5.10E-01 | | | | |  |
| rs10968114 | | | | | C | | | A | | -8.54E-04 | | | 8.49E-04 | | | | | 3.10E-01 | | | | |  |
| rs10971709 | | | | | T | | | C | | -5.82E-04 | | | 1.04E-03 | | | | | 5.30E-01 | | | | |  |
| rs11030618 | | | | | T | | | C | | 1.25E-03 | | | 8.54E-04 | | | | | 1.50E-01 | | | | |  |
| rs1105977 | | | | | T | | | G | | 1.73E-04 | | | 9.50E-04 | | | | | 8.00E-01 | | | | |  |
| rs11084553 | | | | | G | | | A | | 1.99E-03 | | | 1.16E-03 | | | | | 8.40E-02 | | | | |  |
| rs11115176 | | | | | C | | | T | | 1.03E-03 | | | 9.96E-04 | | | | | 2.70E-01 | | | | |  |
| rs1112613 | | | | | A | | | G | | 9.76E-04 | | | 1.09E-03 | | | | | 3.60E-01 | | | | |  |
| rs11155787 | | | | | T | | | C | | 1.34E-04 | | | 8.80E-04 | | | | | 8.50E-01 | | | | |  |
| rs11165643 | | | | | T | | | C | | 6.85E-04 | | | 8.61E-04 | | | | | 4.20E-01 | | | | |  |
| rs11170468 | | | | | C | | | A | | -8.58E-04 | | | 9.97E-04 | | | | | 3.90E-01 | | | | |  |
| rs11251352 | | | | | G | | | A | | 1.31E-04 | | | 8.67E-04 | | | | | 8.80E-01 | | | | |  |
| rs1144387 | | | | | C | | | G | | 6.48E-04 | | | 8.55E-04 | | | | | 4.60E-01 | | | | |  |
| rs11505821 | | | | | T | | | A | | 3.45E-04 | | | 1.73E-03 | | | | | 8.30E-01 | | | | |  |
| rs11556924 | | | | | T | | | C | | 6.61E-04 | | | 8.66E-04 | | | | | 4.70E-01 | | | | |  |
| rs1158805 | | | | | A | | | C | | 7.98E-04 | | | 8.74E-04 | | | | | 3.20E-01 | | | | |  |
| rs11621792 | | | | | C | | | T | | 2.83E-03 | | | 8.53E-04 | | | | | 8.30E-04 | | | | |  |
| rs11640366 | | | | | A | | | C | | -1.16E-04 | | | 8.48E-04 | | | | | 8.20E-01 | | | | |  |
| rs11656076 | | | | | A | | | G | | -1.42E-03 | | | 1.01E-03 | | | | | 1.90E-01 | | | | |  |
| rs11713193 | | | | | A | | | G | | 2.63E-03 | | | 8.46E-04 | | | | | 2.00E-03 | | | | |  |
| rs11738695 | | | | | A | | | C | | 2.53E-04 | | | 8.59E-04 | | | | | 7.80E-01 | | | | |  |
| rs117589665 | | | | | G | | | A | | -6.07E-04 | | | 1.68E-03 | | | | | 7.40E-01 | | | | |  |
| rs11765639 | | | | | A | | | G | | 9.03E-04 | | | 8.71E-04 | | | | | 2.70E-01 | | | | |  |
| rs11855853 | | | | | T | | | C | | -1.40E-04 | | | 9.59E-04 | | | | | 8.90E-01 | | | | |  |
| rs1187352 | | | | | C | | | T | | 7.24E-04 | | | 8.90E-04 | | | | | 4.40E-01 | | | | |  |
| rs11880870 | | | | | G | | | A | | -5.35E-04 | | | 8.46E-04 | | | | | 5.80E-01 | | | | |  |
| rs12044597 | | | | | G | | | A | | -2.04E-03 | | | 8.45E-04 | | | | | 1.90E-02 | | | | |  |
| rs12049202 | | | | | T | | | C | | -6.97E-04 | | | 1.06E-03 | | | | | 5.20E-01 | | | | |  |
| rs12098284 | | | | | T | | | C | | -2.34E-03 | | | 1.28E-03 | | | | | 6.00E-02 | | | | |  |
| rs12150665 | | | | | C | | | T | | -5.11E-04 | | | 8.60E-04 | | | | | 5.30E-01 | | | | |  |
| rs1218822 | | | | | A | | | G | | -9.85E-04 | | | 8.94E-04 | | | | | 3.00E-01 | | | | |  |
| rs12299814 | | | | | A | | | C | | -9.77E-04 | | | 9.86E-04 | | | | | 3.80E-01 | | | | |  |
| rs12320328 | | | | | G | | | A | | -2.17E-03 | | | 1.52E-03 | | | | | 1.60E-01 | | | | |  |
| rs12364470 | | | | | G | | | T | | 2.45E-03 | | | 1.14E-03 | | | | | 3.60E-02 | | | | |  |
| rs1241986 | | | | | A | | | G | | 9.78E-04 | | | 1.19E-03 | | | | | 4.10E-01 | | | | |  |
| rs12429545 | | | | | A | | | G | | 4.22E-05 | | | 1.27E-03 | | | | | 9.90E-01 | | | | |  |
| rs12448257 | | | | | A | | | G | | 1.55E-03 | | | 1.04E-03 | | | | | 1.30E-01 | | | | |  |
| rs12543287 | | | | | C | | | G | | 1.80E-03 | | | 8.81E-04 | | | | | 4.00E-02 | | | | |  |
| rs12629015 | | | | | G | | | A | | -6.91E-04 | | | 1.12E-03 | | | | | 5.80E-01 | | | | |  |
| rs1266874 | | | | | G | | | A | | -5.29E-05 | | | 8.87E-04 | | | | | 9.80E-01 | | | | |  |
| rs12675063 | | | | | T | | | A | | 7.93E-04 | | | 1.32E-03 | | | | | 6.00E-01 | | | | |  |
| rs1268065 | | | | | A | | | G | | -2.42E-03 | | | 8.52E-04 | | | | | 5.10E-03 | | | | |  |
| rs12680842 | | | | | G | | | A | | -1.92E-05 | | | 9.11E-04 | | | | | 9.70E-01 | | | | |  |
| rs12696304 | | | | | G | | | C | | -1.99E-04 | | | 9.58E-04 | | | | | 8.30E-01 | | | | |  |
| rs12718572 | | | | | T | | | C | | 3.03E-05 | | | 8.65E-04 | | | | | 9.90E-01 | | | | |  |
| rs12762034 | | | | | C | | | T | | -8.10E-04 | | | 1.59E-03 | | | | | 6.80E-01 | | | | |  |
| rs12914489 | | | | | A | | | G | | -3.26E-03 | | | 1.36E-03 | | | | | 1.70E-02 | | | | |  |
| rs12939549 | | | | | G | | | A | | -1.15E-04 | | | 8.50E-04 | | | | | 9.10E-01 | | | | |  |
| rs1296328 | | | | | C | | | A | | 5.92E-04 | | | 8.55E-04 | | | | | 5.00E-01 | | | | |  |
| rs13021737 | | | | | G | | | A | | 6.48E-04 | | | 1.12E-03 | | | | | 6.20E-01 | | | | |  |
| rs13035806 | | | | | A | | | G | | -9.78E-04 | | | 1.33E-03 | | | | | 4.60E-01 | | | | |  |
| rs13047416 | | | | | G | | | C | | -4.82E-04 | | | 8.76E-04 | | | | | 6.20E-01 | | | | |  |
| rs13069244 | | | | | A | | | G | | -1.30E-04 | | | 1.62E-03 | | | | | 8.90E-01 | | | | |  |
| rs13094241 | | | | | G | | | T | | -2.43E-04 | | | 9.50E-04 | | | | | 8.40E-01 | | | | |  |
| rs13107325 | | | | | T | | | C | | 1.87E-04 | | | 1.60E-03 | | | | | 9.50E-01 | | | | |  |
| rs13110266 | | | | | A | | | G | | -3.27E-04 | | | 8.60E-04 | | | | | 7.10E-01 | | | | |  |
| rs13150068 | | | | | G | | | A | | -6.29E-04 | | | 8.53E-04 | | | | | 4.50E-01 | | | | |  |
| rs13174863 | | | | | G | | | A | | -1.72E-03 | | | 1.20E-03 | | | | | 1.70E-01 | | | | |  |
| rs13191362 | | | | | G | | | A | | -2.16E-03 | | | 1.29E-03 | | | | | 8.10E-02 | | | | |  |
| rs1320903 | | | | | A | | | G | | -2.20E-04 | | | 9.09E-04 | | | | | 8.00E-01 | | | | |  |
| rs1321432 | | | | | C | | | A | | 1.20E-03 | | | 8.80E-04 | | | | | 1.60E-01 | | | | |  |
| rs13251458 | | | | | A | | | G | | 1.19E-03 | | | 8.49E-04 | | | | | 1.80E-01 | | | | |  |
| rs1327259 | | | | | G | | | A | | -3.10E-04 | | | 8.73E-04 | | | | | 7.70E-01 | | | | |  |
| rs13389219 | | | | | T | | | C | | 1.85E-03 | | | 8.65E-04 | | | | | 3.60E-02 | | | | |  |
| rs1365466 | | | | | T | | | C | | -5.67E-04 | | | 9.69E-04 | | | | | 5.80E-01 | | | | |  |
| rs13702 | | | | | C | | | T | | 2.00E-03 | | | 9.36E-04 | | | | | 3.50E-02 | | | | |  |
| rs1371108 | | | | | A | | | C | | -7.81E-04 | | | 9.10E-04 | | | | | 4.40E-01 | | | | |  |
| rs1409818 | | | | | T | | | C | | 7.78E-04 | | | 1.41E-03 | | | | | 5.30E-01 | | | | |  |
| rs1412235 | | | | | C | | | G | | -1.18E-03 | | | 9.02E-04 | | | | | 2.10E-01 | | | | |  |
| rs1421334 | | | | | C | | | A | | -7.79E-04 | | | 8.54E-04 | | | | | 3.70E-01 | | | | |  |
| rs1452075 | | | | | T | | | C | | 2.27E-03 | | | 9.57E-04 | | | | | 2.10E-02 | | | | |  |
| rs1454687 | | | | | G | | | C | | -1.09E-03 | | | 8.46E-04 | | | | | 1.90E-01 | | | | |  |
| rs1465900 | | | | | C | | | A | | 7.45E-04 | | | 1.04E-03 | | | | | 5.00E-01 | | | | |  |
| rs1472169 | | | | | T | | | C | | 5.58E-05 | | | 8.64E-04 | | | | | 9.20E-01 | | | | |  |
| rs150449323 | | | | | C | | | T | | -3.20E-03 | | | 1.45E-03 | | | | | 3.00E-02 | | | | |  |
| rs1521527 | | | | | C | | | G | | -1.33E-03 | | | 8.51E-04 | | | | | 1.20E-01 | | | | |  |
| rs1528435 | | | | | T | | | C | | -1.37E-03 | | | 8.72E-04 | | | | | 1.20E-01 | | | | |  |
| rs1534696 | | | | | A | | | C | | 3.71E-05 | | | 8.48E-04 | | | | | 9.70E-01 | | | | |  |
| rs1561442 | | | | | A | | | G | | -1.73E-03 | | | 1.24E-03 | | | | | 1.80E-01 | | | | |  |
| rs157935 | | | | | G | | | T | | 1.46E-03 | | | 9.23E-04 | | | | | 1.00E-01 | | | | |  |
| rs1624134 | | | | | C | | | G | | -1.90E-04 | | | 8.65E-04 | | | | | 9.10E-01 | | | | |  |
| rs1640269 | | | | | C | | | A | | 1.04E-03 | | | 9.36E-04 | | | | | 2.70E-01 | | | | |  |
| rs1650548 | | | | | C | | | G | | 4.89E-04 | | | 1.01E-03 | | | | | 6.50E-01 | | | | |  |
| rs1656377 | | | | | C | | | T | | 1.47E-03 | | | 8.58E-04 | | | | | 7.30E-02 | | | | |  |
| rs1681740 | | | | | C | | | A | | -1.25E-03 | | | 8.71E-04 | | | | | 1.60E-01 | | | | |  |
| rs16849710 | | | | | G | | | A | | -1.87E-03 | | | 8.53E-04 | | | | | 2.60E-02 | | | | |  |
| rs16851483 | | | | | T | | | G | | 8.31E-04 | | | 1.71E-03 | | | | | 6.60E-01 | | | | |  |
| rs16903285 | | | | | C | | | T | | 1.45E-03 | | | 1.26E-03 | | | | | 2.80E-01 | | | | |  |
| rs16942887 | | | | | A | | | G | | 6.62E-04 | | | 1.32E-03 | | | | | 6.50E-01 | | | | |  |
| rs17014375 | | | | | G | | | T | | 1.30E-03 | | | 1.25E-03 | | | | | 2.50E-01 | | | | |  |
| rs17033117 | | | | | T | | | C | | 6.97E-04 | | | 1.11E-03 | | | | | 5.10E-01 | | | | |  |
| rs17056301 | | | | | C | | | T | | 6.20E-04 | | | 9.71E-04 | | | | | 5.10E-01 | | | | |  |
| rs17119937 | | | | | C | | | T | | -1.19E-03 | | | 1.71E-03 | | | | | 5.20E-01 | | | | |  |
| rs17184382 | | | | | C | | | A | | 2.83E-04 | | | 8.55E-04 | | | | | 6.80E-01 | | | | |  |
| rs17207196 | | | | | T | | | C | | -1.43E-03 | | | 8.57E-04 | | | | | 9.80E-02 | | | | |  |
| rs1730862 | | | | | A | | | G | | -3.95E-03 | | | 8.96E-04 | | | | | 1.30E-05 | | | | |  |
| rs17399237 | | | | | C | | | T | | -2.27E-03 | | | 8.52E-04 | | | | | 8.90E-03 | | | | |  |
| rs17425707 | | | | | C | | | T | | -1.59E-03 | | | 1.39E-03 | | | | | 2.20E-01 | | | | |  |
| rs17535749 | | | | | A | | | G | | -1.48E-03 | | | 1.36E-03 | | | | | 2.80E-01 | | | | |  |
| rs17551974 | | | | | A | | | C | | -2.05E-03 | | | 1.09E-03 | | | | | 6.80E-02 | | | | |  |
| rs1772189 | | | | | A | | | T | | -4.46E-04 | | | 8.47E-04 | | | | | 6.00E-01 | | | | |  |
| rs17789218 | | | | | C | | | T | | 1.38E-03 | | | 9.86E-04 | | | | | 1.70E-01 | | | | |  |
| rs1801282 | | | | | G | | | C | | 8.06E-04 | | | 1.29E-03 | | | | | 5.20E-01 | | | | |  |
| rs1863652 | | | | | A | | | G | | -8.74E-04 | | | 8.88E-04 | | | | | 3.30E-01 | | | | |  |
| rs1866956 | | | | | T | | | C | | -2.72E-04 | | | 9.10E-04 | | | | | 7.90E-01 | | | | |  |
| rs1883025 | | | | | T | | | C | | 7.85E-06 | | | 9.71E-04 | | | | | 9.70E-01 | | | | |  |
| rs1891216 | | | | | G | | | T | | 4.57E-04 | | | 8.77E-04 | | | | | 6.10E-01 | | | | |  |
| rs189595752 | | | | | G | | | A | | 1.56E-03 | | | 1.37E-03 | | | | | 2.20E-01 | | | | |  |
| rs1928295 | | | | | C | | | T | | 7.75E-04 | | | 8.53E-04 | | | | | 3.60E-01 | | | | |  |
| rs1982725 | | | | | T | | | C | | 1.81E-03 | | | 8.50E-04 | | | | | 3.60E-02 | | | | |  |
| rs200810 | | | | | C | | | T | | 1.81E-03 | | | 8.75E-04 | | | | | 3.80E-02 | | | | |  |
| rs2009416 | | | | | T | | | C | | -5.07E-04 | | | 8.82E-04 | | | | | 5.40E-01 | | | | |  |
| rs2065418 | | | | | G | | | T | | 1.04E-03 | | | 8.80E-04 | | | | | 2.30E-01 | | | | |  |
| rs215634 | | | | | G | | | A | | 1.39E-03 | | | 8.71E-04 | | | | | 1.10E-01 | | | | |  |
| rs2174307 | | | | | C | | | G | | 8.33E-04 | | | 8.57E-04 | | | | | 3.40E-01 | | | | |  |
| rs217671 | | | | | G | | | A | | -9.96E-04 | | | 9.50E-04 | | | | | 2.90E-01 | | | | |  |
| rs2224585 | | | | | A | | | G | | -1.58E-04 | | | 9.88E-04 | | | | | 9.20E-01 | | | | |  |
| rs2241210 | | | | | G | | | A | | -1.71E-03 | | | 8.46E-04 | | | | | 3.60E-02 | | | | |  |
| rs2246012 | | | | | C | | | T | | -8.86E-04 | | | 1.13E-03 | | | | | 4.30E-01 | | | | |  |
| rs2325036 | | | | | C | | | A | | -3.16E-04 | | | 8.75E-04 | | | | | 7.30E-01 | | | | |  |
| rs2351958 | | | | | A | | | C | | 6.93E-04 | | | 8.67E-04 | | | | | 4.10E-01 | | | | |  |
| rs2357760 | | | | | A | | | G | | 3.81E-04 | | | 9.07E-04 | | | | | 6.30E-01 | | | | |  |
| rs2393791 | | | | | T | | | C | | -2.75E-04 | | | 8.73E-04 | | | | | 6.90E-01 | | | | |  |
| rs2479958 | | | | | G | | | A | | -9.26E-05 | | | 8.56E-04 | | | | | 9.20E-01 | | | | |  |
| rs2481665 | | | | | C | | | T | | -6.30E-04 | | | 8.51E-04 | | | | | 4.50E-01 | | | | |  |
| rs2498786 | | | | | G | | | C | | -1.16E-03 | | | 8.72E-04 | | | | | 2.00E-01 | | | | |  |
| rs2543132 | | | | | C | | | G | | -9.14E-04 | | | 1.09E-03 | | | | | 4.20E-01 | | | | |  |
| rs2551644 | | | | | A | | | T | | -2.60E-04 | | | 1.09E-03 | | | | | 8.20E-01 | | | | |  |
| rs2608703 | | | | | A | | | C | | -1.69E-03 | | | 8.49E-04 | | | | | 5.10E-02 | | | | |  |
| rs2612038 | | | | | T | | | C | | 2.88E-04 | | | 1.44E-03 | | | | | 8.50E-01 | | | | |  |
| rs2642438 | | | | | G | | | A | | 8.30E-05 | | | 9.24E-04 | | | | | 9.40E-01 | | | | |  |
| rs2643452 | | | | | A | | | T | | 2.01E-03 | | | 8.49E-04 | | | | | 2.00E-02 | | | | |  |
| rs2693826 | | | | | A | | | G | | 4.23E-04 | | | 8.53E-04 | | | | | 6.50E-01 | | | | |  |
| rs2694047 | | | | | G | | | A | | 2.80E-04 | | | 9.80E-04 | | | | | 7.60E-01 | | | | |  |
| rs273504 | | | | | G | | | A | | -7.97E-04 | | | 8.57E-04 | | | | | 3.60E-01 | | | | |  |
| rs2744974 | | | | | T | | | C | | 9.34E-05 | | | 9.01E-04 | | | | | 9.00E-01 | | | | |  |
| rs2791644 | | | | | C | | | T | | 1.91E-03 | | | 1.00E-03 | | | | | 4.40E-02 | | | | |  |
| rs28507491 | | | | | A | | | G | | -8.02E-04 | | | 8.73E-04 | | | | | 3.50E-01 | | | | |  |
| rs2861683 | | | | | C | | | A | | 8.25E-04 | | | 8.56E-04 | | | | | 3.30E-01 | | | | |  |
| rs2868975 | | | | | A | | | G | | 1.53E-03 | | | 1.11E-03 | | | | | 1.80E-01 | | | | |  |
| rs2875762 | | | | | C | | | G | | 2.86E-03 | | | 9.94E-04 | | | | | 3.40E-03 | | | | |  |
| rs2931434 | | | | | T | | | C | | -3.00E-04 | | | 8.95E-04 | | | | | 7.60E-01 | | | | |  |
| rs2972145 | | | | | C | | | T | | -1.62E-03 | | | 8.84E-04 | | | | | 6.50E-02 | | | | |  |
| rs3001032 | | | | | C | | | T | | 5.03E-04 | | | 9.06E-04 | | | | | 5.90E-01 | | | | |  |
| rs34255979 | | | | | T | | | C | | 3.71E-03 | | | 1.31E-03 | | | | | 5.60E-03 | | | | |  |
| rs349088 | | | | | A | | | C | | 1.10E-03 | | | 8.48E-04 | | | | | 2.00E-01 | | | | |  |
| rs35350976 | | | | | G | | | A | | -5.76E-04 | | | 1.10E-03 | | | | | 5.70E-01 | | | | |  |
| rs355777 | | | | | C | | | G | | 6.95E-04 | | | 8.63E-04 | | | | | 4.10E-01 | | | | |  |
| rs3749897 | | | | | T | | | C | | 1.72E-03 | | | 8.58E-04 | | | | | 4.20E-02 | | | | |  |
| rs3754963 | | | | | T | | | A | | -1.90E-03 | | | 9.67E-04 | | | | | 4.90E-02 | | | | |  |
| rs3772882 | | | | | A | | | C | | 1.50E-03 | | | 8.74E-04 | | | | | 8.30E-02 | | | | |  |
| rs3800229 | | | | | T | | | G | | 4.12E-05 | | | 9.39E-04 | | | | | 9.80E-01 | | | | |  |
| rs3806114 | | | | | A | | | G | | -6.73E-04 | | | 9.07E-04 | | | | | 4.60E-01 | | | | |  |
| rs3806572 | | | | | A | | | G | | -3.23E-04 | | | 9.32E-04 | | | | | 7.30E-01 | | | | |  |
| rs3807645 | | | | | A | | | G | | -2.47E-03 | | | 1.02E-03 | | | | | 1.70E-02 | | | | |  |
| rs380857 | | | | | A | | | C | | 8.85E-04 | | | 1.33E-03 | | | | | 5.70E-01 | | | | |  |
| rs3810027 | | | | | G | | | C | | -1.03E-03 | | | 8.98E-04 | | | | | 2.40E-01 | | | | |  |
| rs3829639 | | | | | G | | | A | | 1.28E-04 | | | 9.04E-04 | | | | | 8.30E-01 | | | | |  |
| rs38314 | | | | | A | | | G | | -1.77E-03 | | | 8.47E-04 | | | | | 4.00E-02 | | | | |  |
| rs3902951 | | | | | G | | | T | | -3.57E-05 | | | 1.00E-03 | | | | | 9.70E-01 | | | | |  |
| rs3904244 | | | | | A | | | T | | -9.95E-05 | | | 1.23E-03 | | | | | 9.00E-01 | | | | |  |
| rs40067 | | | | | A | | | G | | -6.27E-04 | | | 1.13E-03 | | | | | 6.10E-01 | | | | |  |
| rs40270 | | | | | C | | | A | | -8.91E-04 | | | 1.01E-03 | | | | | 3.40E-01 | | | | |  |
| rs4072917 | | | | | A | | | G | | 3.96E-04 | | | 8.49E-04 | | | | | 6.00E-01 | | | | |  |
| rs4148005 | | | | | G | | | T | | -6.76E-04 | | | 9.11E-04 | | | | | 4.70E-01 | | | | |  |
| rs4148155 | | | | | G | | | A | | 1.04E-03 | | | 1.32E-03 | | | | | 4.40E-01 | | | | |  |
| rs4237643 | | | | | G | | | T | | 3.20E-04 | | | 9.15E-04 | | | | | 7.00E-01 | | | | |  |
| rs4240624 | | | | | A | | | G | | -3.31E-03 | | | 1.47E-03 | | | | | 2.30E-02 | | | | |  |
| rs427943 | | | | | C | | | A | | -3.21E-04 | | | 8.54E-04 | | | | | 7.40E-01 | | | | |  |
| rs4307239 | | | | | G | | | A | | 8.82E-04 | | | 8.51E-04 | | | | | 2.80E-01 | | | | |  |
| rs4338849 | | | | | A | | | G | | -5.28E-04 | | | 8.48E-04 | | | | | 5.40E-01 | | | | |  |
| rs4430672 | | | | | C | | | T | | -1.61E-03 | | | 1.06E-03 | | | | | 1.40E-01 | | | | |  |
| rs4556997 | | | | | A | | | C | | -9.62E-04 | | | 1.24E-03 | | | | | 4.50E-01 | | | | |  |
| rs4567095 | | | | | T | | | C | | 1.06E-03 | | | 9.18E-04 | | | | | 2.40E-01 | | | | |  |
| rs4568281 | | | | | A | | | G | | 8.46E-05 | | | 9.06E-04 | | | | | 9.10E-01 | | | | |  |
| rs4589691 | | | | | G | | | C | | -4.03E-04 | | | 1.19E-03 | | | | | 7.50E-01 | | | | |  |
| rs4639527 | | | | | G | | | A | | -5.43E-05 | | | 9.16E-04 | | | | | 9.30E-01 | | | | |  |
| rs4639796 | | | | | A | | | G | | 6.23E-04 | | | 1.15E-03 | | | | | 5.60E-01 | | | | |  |
| rs4660443 | | | | | T | | | C | | 1.28E-03 | | | 1.03E-03 | | | | | 2.00E-01 | | | | |  |
| rs4665972 | | | | | C | | | T | | 5.58E-03 | | | 8.70E-04 | | | | | 1.20E-10 | | | | |  |
| rs4671328 | | | | | G | | | T | | -3.44E-04 | | | 8.58E-04 | | | | | 6.80E-01 | | | | |  |
| rs4674669 | | | | | T | | | C | | -1.05E-03 | | | 1.22E-03 | | | | | 3.70E-01 | | | | |  |
| rs4740619 | | | | | C | | | T | | -8.06E-04 | | | 8.51E-04 | | | | | 3.00E-01 | | | | |  |
| rs4800191 | | | | | C | | | G | | 5.13E-04 | | | 8.85E-04 | | | | | 5.80E-01 | | | | |  |
| rs4804414 | | | | | T | | | C | | -7.47E-05 | | | 8.53E-04 | | | | | 9.70E-01 | | | | |  |
| rs4812336 | | | | | A | | | G | | 1.12E-03 | | | 9.20E-04 | | | | | 2.10E-01 | | | | |  |
| rs4813619 | | | | | T | | | G | | -1.06E-03 | | | 8.52E-04 | | | | | 2.00E-01 | | | | |  |
| rs4820091 | | | | | G | | | T | | 5.98E-04 | | | 1.11E-03 | | | | | 5.90E-01 | | | | |  |
| rs4820408 | | | | | G | | | T | | 1.06E-03 | | | 8.63E-04 | | | | | 2.10E-01 | | | | |  |
| rs4842491 | | | | | T | | | C | | 1.23E-03 | | | 9.31E-04 | | | | | 2.00E-01 | | | | |  |
| rs4846914 | | | | | A | | | G | | 1.39E-04 | | | 8.65E-04 | | | | | 9.00E-01 | | | | |  |
| rs4851029 | | | | | G | | | T | | -7.93E-04 | | | 8.47E-04 | | | | | 3.70E-01 | | | | |  |
| rs4880341 | | | | | T | | | C | | 1.21E-04 | | | 8.57E-04 | | | | | 9.40E-01 | | | | |  |
| rs4936175 | | | | | C | | | T | | -1.38E-04 | | | 8.51E-04 | | | | | 9.00E-01 | | | | |  |
| rs4939883 | | | | | C | | | T | | -2.07E-03 | | | 1.10E-03 | | | | | 6.20E-02 | | | | |  |
| rs4954638 | | | | | C | | | A | | -1.75E-03 | | | 9.96E-04 | | | | | 6.80E-02 | | | | |  |
| rs538579 | | | | | C | | | G | | 7.12E-04 | | | 9.10E-04 | | | | | 4.10E-01 | | | | |  |
| rs543874 | | | | | G | | | A | | 2.29E-03 | | | 1.04E-03 | | | | | 2.80E-02 | | | | |  |
| rs559231 | | | | | T | | | G | | 5.02E-04 | | | 8.69E-04 | | | | | 6.00E-01 | | | | |  |
| rs56024084 | | | | | T | | | C | | 5.13E-04 | | | 8.63E-04 | | | | | 5.00E-01 | | | | |  |
| rs56077345 | | | | | C | | | G | | -1.67E-04 | | | 1.65E-03 | | | | | 9.40E-01 | | | | |  |
| rs587252 | | | | | C | | | A | | -7.35E-05 | | | 1.50E-03 | | | | | 9.60E-01 | | | | |  |
| rs61856602 | | | | | G | | | A | | -5.44E-04 | | | 9.04E-04 | | | | | 5.10E-01 | | | | |  |
| rs6235 | | | | | G | | | C | | 4.45E-04 | | | 9.54E-04 | | | | | 7.00E-01 | | | | |  |
| rs62623385 | | | | | T | | | A | | -4.68E-03 | | | 2.32E-03 | | | | | 4.20E-02 | | | | |  |
| rs6265 | | | | | T | | | C | | -1.46E-03 | | | 1.08E-03 | | | | | 2.00E-01 | | | | |  |
| rs633695 | | | | | G | | | A | | -2.96E-04 | | | 9.34E-04 | | | | | 7.30E-01 | | | | |  |
| rs6471941 | | | | | A | | | G | | 1.75E-03 | | | 1.13E-03 | | | | | 1.30E-01 | | | | |  |
| rs6486122 | | | | | T | | | C | | -1.93E-04 | | | 9.14E-04 | | | | | 8.80E-01 | | | | |  |
| rs6512302 | | | | | C | | | G | | 1.53E-03 | | | 9.72E-04 | | | | | 1.20E-01 | | | | |  |
| rs6545714 | | | | | A | | | G | | -3.41E-04 | | | 8.65E-04 | | | | | 6.30E-01 | | | | |  |
| rs6561943 | | | | | T | | | C | | 1.36E-03 | | | 9.66E-04 | | | | | 1.60E-01 | | | | |  |
| rs657452 | | | | | G | | | A | | -1.74E-03 | | | 8.70E-04 | | | | | 5.80E-02 | | | | |  |
| rs6591407 | | | | | A | | | C | | -6.56E-04 | | | 1.09E-03 | | | | | 5.20E-01 | | | | |  |
| rs6595205 | | | | | G | | | C | | -1.43E-03 | | | 8.48E-04 | | | | | 9.80E-02 | | | | |  |
| rs663129 | | | | | A | | | G | | -1.51E-04 | | | 1.00E-03 | | | | | 8.90E-01 | | | | |  |
| rs668871 | | | | | T | | | C | | 1.21E-03 | | | 8.48E-04 | | | | | 1.50E-01 | | | | |  |
| rs6758199 | | | | | T | | | C | | 1.76E-03 | | | 1.70E-03 | | | | | 3.20E-01 | | | | |  |
| rs6785245 | | | | | C | | | T | | 1.89E-04 | | | 8.62E-04 | | | | | 8.00E-01 | | | | |  |
| rs6815910 | | | | | A | | | T | | -2.09E-03 | | | 8.52E-04 | | | | | 1.40E-02 | | | | |  |
| rs6841761 | | | | | T | | | G | | 6.80E-04 | | | 8.49E-04 | | | | | 4.10E-01 | | | | |  |
| rs6860245 | | | | | C | | | G | | 4.77E-04 | | | 9.81E-04 | | | | | 6.60E-01 | | | | |  |
| rs686030 | | | | | A | | | C | | -9.69E-04 | | | 1.21E-03 | | | | | 4.20E-01 | | | | |  |
| rs695272 | | | | | C | | | T | | -1.40E-03 | | | 8.93E-04 | | | | | 1.10E-01 | | | | |  |
| rs7015 | | | | | A | | | G | | -9.33E-04 | | | 1.09E-03 | | | | | 4.00E-01 | | | | |  |
| rs7025938 | | | | | G | | | C | | -4.75E-04 | | | 9.12E-04 | | | | | 5.90E-01 | | | | |  |
| rs705704 | | | | | A | | | G | | -1.07E-04 | | | 8.91E-04 | | | | | 8.20E-01 | | | | |  |
| rs7084454 | | | | | A | | | G | | 1.49E-03 | | | 9.07E-04 | | | | | 9.80E-02 | | | | |  |
| rs7096764 | | | | | A | | | G | | -7.54E-04 | | | 9.25E-04 | | | | | 4.70E-01 | | | | |  |
| rs7117238 | | | | | A | | | G | | -2.19E-03 | | | 1.16E-03 | | | | | 5.40E-02 | | | | |  |
| rs7138803 | | | | | A | | | G | | -9.64E-04 | | | 8.75E-04 | | | | | 2.60E-01 | | | | |  |
| rs7144011 | | | | | T | | | G | | 7.53E-04 | | | 1.02E-03 | | | | | 4.60E-01 | | | | |  |
| rs7148846 | | | | | G | | | T | | -1.32E-03 | | | 1.08E-03 | | | | | 2.20E-01 | | | | |  |
| rs7196720 | | | | | C | | | T | | 1.02E-04 | | | 8.46E-04 | | | | | 9.10E-01 | | | | |  |
| rs7222349 | | | | | A | | | G | | -8.82E-04 | | | 8.93E-04 | | | | | 3.20E-01 | | | | |  |
| rs7239575 | | | | | C | | | T | | -7.82E-04 | | | 8.47E-04 | | | | | 3.60E-01 | | | | |  |
| rs7262150 | | | | | C | | | T | | -1.31E-04 | | | 1.00E-03 | | | | | 9.40E-01 | | | | |  |
| rs73079476 | | | | | C | | | A | | -7.78E-04 | | | 1.18E-03 | | | | | 5.40E-01 | | | | |  |
| rs7314285 | | | | | G | | | T | | 3.45E-03 | | | 1.68E-03 | | | | | 4.10E-02 | | | | |  |
| rs7318817 | | | | | T | | | C | | 8.06E-04 | | | 8.73E-04 | | | | | 3.30E-01 | | | | |  |
| rs738409 | | | | | G | | | C | | 2.00E-05 | | | 1.03E-03 | | | | | 9.70E-01 | | | | |  |
| rs7451021 | | | | | C | | | T | | -1.01E-03 | | | 9.14E-04 | | | | | 2.30E-01 | | | | |  |
| rs7498665 | | | | | G | | | A | | 4.53E-04 | | | 8.64E-04 | | | | | 6.00E-01 | | | | |  |
| rs7557796 | | | | | C | | | T | | 2.31E-04 | | | 8.85E-04 | | | | | 8.00E-01 | | | | |  |
| rs757869 | | | | | G | | | A | | 1.28E-03 | | | 9.29E-04 | | | | | 1.60E-01 | | | | |  |
| rs7615297 | | | | | G | | | C | | -9.23E-05 | | | 1.18E-03 | | | | | 8.70E-01 | | | | |  |
| rs7626079 | | | | | T | | | C | | -9.66E-04 | | | 8.93E-04 | | | | | 3.10E-01 | | | | |  |
| rs7678138 | | | | | A | | | G | | -9.66E-04 | | | 1.28E-03 | | | | | 4.60E-01 | | | | |  |
| rs7683836 | | | | | A | | | G | | 1.36E-03 | | | 8.52E-04 | | | | | 1.20E-01 | | | | |  |
| rs7703576 | | | | | C | | | T | | 5.72E-04 | | | 9.26E-04 | | | | | 5.10E-01 | | | | |  |
| rs7715256 | | | | | T | | | G | | 4.13E-04 | | | 8.53E-04 | | | | | 6.30E-01 | | | | |  |
| rs7724675 | | | | | A | | | G | | 5.41E-04 | | | 1.03E-03 | | | | | 5.70E-01 | | | | |  |
| rs7730898 | | | | | A | | | G | | 6.12E-04 | | | 9.50E-04 | | | | | 5.10E-01 | | | | |  |
| rs7761673 | | | | | A | | | T | | 4.49E-04 | | | 1.02E-03 | | | | | 7.40E-01 | | | | |  |
| rs7780752 | | | | | C | | | T | | -1.73E-04 | | | 8.80E-04 | | | | | 8.50E-01 | | | | |  |
| rs7788008 | | | | | A | | | G | | -3.47E-04 | | | 8.53E-04 | | | | | 6.90E-01 | | | | |  |
| rs7819514 | | | | | A | | | G | | 2.81E-05 | | | 8.98E-04 | | | | | 9.10E-01 | | | | |  |
| rs7844647 | | | | | C | | | T | | -7.40E-04 | | | 9.60E-04 | | | | | 4.40E-01 | | | | |  |
| rs7860634 | | | | | A | | | G | | -2.12E-04 | | | 8.56E-04 | | | | | 8.70E-01 | | | | |  |
| rs7869771 | | | | | C | | | A | | -2.78E-04 | | | 9.44E-04 | | | | | 6.70E-01 | | | | |  |
| rs7925214 | | | | | T | | | C | | 2.91E-04 | | | 8.53E-04 | | | | | 7.40E-01 | | | | |  |
| rs796004 | | | | | T | | | C | | 2.33E-03 | | | 9.67E-04 | | | | | 1.40E-02 | | | | |  |
| rs79717793 | | | | | A | | | G | | -1.13E-03 | | | 1.17E-03 | | | | | 3.40E-01 | | | | |  |
| rs7983065 | | | | | T | | | C | | -1.14E-03 | | | 8.48E-04 | | | | | 1.60E-01 | | | | |  |
| rs802685 | | | | | C | | | T | | 2.57E-05 | | | 9.82E-04 | | | | | 9.60E-01 | | | | |  |
| rs8033077 | | | | | C | | | T | | -8.64E-04 | | | 9.46E-04 | | | | | 3.70E-01 | | | | |  |
| rs8047395 | | | | | A | | | G | | 4.94E-04 | | | 8.49E-04 | | | | | 4.70E-01 | | | | |  |
| rs806600 | | | | | G | | | A | | 2.05E-03 | | | 8.46E-04 | | | | | 1.30E-02 | | | | |  |
| rs8079418 | | | | | C | | | T | | 8.59E-04 | | | 8.67E-04 | | | | | 3.20E-01 | | | | |  |
| rs8097672 | | | | | T | | | A | | 2.34E-03 | | | 1.20E-03 | | | | | 4.20E-02 | | | | |  |
| rs8097783 | | | | | A | | | G | | 1.80E-03 | | | 1.62E-03 | | | | | 2.70E-01 | | | | |  |
| rs8181823 | | | | | C | | | A | | -2.65E-04 | | | 9.92E-04 | | | | | 7.80E-01 | | | | |  |
| rs865809 | | | | | G | | | A | | -7.06E-04 | | | 1.00E-03 | | | | | 4.60E-01 | | | | |  |
| rs872281 | | | | | T | | | C | | -9.09E-05 | | | 1.11E-03 | | | | | 9.80E-01 | | | | |  |
| rs876605 | | | | | G | | | A | | -1.63E-04 | | | 9.63E-04 | | | | | 8.70E-01 | | | | |  |
| rs879620 | | | | | T | | | C | | -2.22E-04 | | | 8.72E-04 | | | | | 7.70E-01 | | | | |  |
| rs889398 | | | | | T | | | C | | -1.23E-03 | | | 8.62E-04 | | | | | 1.40E-01 | | | | |  |
| rs901630 | | | | | T | | | C | | -4.40E-04 | | | 8.66E-04 | | | | | 6.60E-01 | | | | |  |
| rs902695 | | | | | A | | | G | | 4.13E-04 | | | 8.50E-04 | | | | | 6.30E-01 | | | | |  |
| rs9267551 | | | | | G | | | C | | -9.98E-04 | | | 1.19E-03 | | | | | 4.00E-01 | | | | |  |
| rs9294260 | | | | | A | | | G | | -9.22E-04 | | | 8.52E-04 | | | | | 2.80E-01 | | | | |  |
| rs9297994 | | | | | A | | | G | | 1.68E-03 | | | 8.94E-04 | | | | | 5.70E-02 | | | | |  |
| rs9300422 | | | | | G | | | A | | -9.06E-04 | | | 9.05E-04 | | | | | 3.30E-01 | | | | |  |
| rs9379084 | | | | | A | | | G | | -1.45E-03 | | | 1.36E-03 | | | | | 3.10E-01 | | | | |  |
| rs9408882 | | | | | A | | | G | | -1.97E-04 | | | 8.49E-04 | | | | | 8.20E-01 | | | | |  |
| rs946824 | | | | | C | | | T | | -8.19E-04 | | | 1.26E-03 | | | | | 5.10E-01 | | | | |  |
| rs947612 | | | | | A | | | G | | -1.46E-03 | | | 9.79E-04 | | | | | 1.40E-01 | | | | |  |
| rs9522285 | | | | | A | | | G | | 8.46E-05 | | | 8.58E-04 | | | | | 9.10E-01 | | | | |  |
| rs9538162 | | | | | C | | | T | | -6.39E-04 | | | 8.55E-04 | | | | | 4.50E-01 | | | | |  |
| rs9547153 | | | | | G | | | A | | -4.80E-05 | | | 8.83E-04 | | | | | 1.00E+00 | | | | |  |
| rs9571687 | | | | | A | | | C | | -5.37E-04 | | | 9.02E-04 | | | | | 6.20E-01 | | | | |  |
| rs9615905 | | | | | T | | | C | | 2.45E-04 | | | 8.49E-04 | | | | | 8.00E-01 | | | | |  |
| rs964184 | | | | | C | | | G | | 3.75E-03 | | | 1.25E-03 | | | | | 2.70E-03 | | | | |  |
| rs970548 | | | | | C | | | A | | -3.47E-04 | | | 9.81E-04 | | | | | 6.70E-01 | | | | |  |
| rs9739640 | | | | | G | | | A | | 9.84E-04 | | | 1.15E-03 | | | | | 3.90E-01 | | | | |  |
| rs977747 | | | | | G | | | T | | 4.67E-04 | | | 8.59E-04 | | | | | 5.50E-01 | | | | |  |
| rs9783858 | | | | | T | | | C | | -2.60E-04 | | | 8.51E-04 | | | | | 7.80E-01 | | | | |  |
| rs9806742 | | | | | A | | | G | | 8.55E-04 | | | 1.26E-03 | | | | | 5.40E-01 | | | | |  |
| rs9816226 | | | | | T | | | A | | 2.10E-03 | | | 1.09E-03 | | | | | 6.00E-02 | | | | |  |
| rs9845966 | | | | | G | | | T | | 1.55E-03 | | | 8.49E-04 | | | | | 6.30E-02 | | | | |  |
| rs9849171 | | | | | C | | | G | | 4.18E-04 | | | 8.80E-04 | | | | | 6.30E-01 | | | | |  |
| rs987237 | | | | | G | | | A | | 6.00E-04 | | | 1.10E-03 | | | | | 5.90E-01 | | | | |  |
| rs9927848 | | | | | A | | | C | | -2.84E-03 | | | 9.58E-04 | | | | | 3.70E-03 | | | | |  |
| rs9989419 | | | | | G | | | A | | -6.04E-05 | | | 8.65E-04 | | | | | 9.10E-01 | | | | |  |
| rs999889 | | | | | A | | | G | | -4.00E-04 | | | 9.41E-04 | | | | | 6.80E-01 | | | | |  |
| **SHBG** | | | | |  | | |  | |  | | |  | | | | |  | | | | |  |
| rs10009336 | | | | | T | | | C | | 7.04E-04 | | | 1.27E-03 | | | | | 8.50E-01 | | | | |  |
| rs10027275 | | | | | C | | | G | | -1.07E-02 | | | 1.07E-03 | | | | | 1.10E-26 | | | | |  |
| rs10123811 | | | | | T | | | C | | 8.83E-03 | | | 9.46E-04 | | | | | 3.00E-22 | | | | |  |
| rs10132280 | | | | | A | | | C | | 8.79E-03 | | | 1.03E-03 | | | | | 2.00E-17 | | | | |  |
| rs10182181 | | | | | G | | | A | | -1.70E-03 | | | 9.29E-04 | | | | | 9.30E-02 | | | | |  |
| rs10197031 | | | | | C | | | T | | -2.99E-03 | | | 1.03E-03 | | | | | 2.80E-03 | | | | |  |
| rs10208512 | | | | | A | | | G | | 1.45E-02 | | | 2.27E-03 | | | | | 9.00E-12 | | | | |  |
| rs102275 | | | | | C | | | T | | -1.02E-02 | | | 9.76E-04 | | | | | 2.20E-26 | | | | |  |
| rs10238028 | | | | | G | | | A | | 1.64E-02 | | | 1.87E-03 | | | | | 3.60E-19 | | | | |  |
| rs10243319 | | | | | C | | | T | | 4.43E-03 | | | 9.54E-04 | | | | | 2.90E-06 | | | | |  |
| rs10245356 | | | | | T | | | C | | 6.59E-03 | | | 9.64E-04 | | | | | 5.40E-12 | | | | |  |
| rs10247983 | | | | | A | | | G | | -5.01E-03 | | | 1.78E-03 | | | | | 4.80E-03 | | | | |  |
| rs10248136 | | | | | T | | | C | | 2.49E-03 | | | 9.35E-04 | | | | | 5.40E-03 | | | | |  |
| rs10278546 | | | | | C | | | A | | 1.56E-02 | | | 1.18E-03 | | | | | 2.10E-40 | | | | |  |
| rs10468017 | | | | | T | | | C | | -2.14E-03 | | | 1.02E-03 | | | | | 1.10E-01 | | | | |  |
| rs10478110 | | | | | C | | | A | | -4.91E-03 | | | 9.44E-04 | | | | | 9.60E-08 | | | | |  |
| rs10742752 | | | | | C | | | T | | -8.25E-04 | | | 9.58E-04 | | | | | 4.30E-01 | | | | |  |
| rs10747488 | | | | | A | | | C | | 5.22E-03 | | | 1.10E-03 | | | | | 2.20E-06 | | | | |  |
| rs10768994 | | | | | C | | | T | | 2.26E-03 | | | 9.44E-04 | | | | | 2.20E-02 | | | | |  |
| rs10795422 | | | | | G | | | A | | -1.40E-03 | | | 1.00E-03 | | | | | 2.50E-01 | | | | |  |
| rs10808546 | | | | | T | | | C | | 1.04E-04 | | | 9.42E-04 | | | | | 8.40E-01 | | | | |  |
| rs10864070 | | | | | A | | | G | | 8.25E-03 | | | 1.62E-03 | | | | | 4.70E-08 | | | | |  |
| rs10867256 | | | | | T | | | C | | 2.31E-03 | | | 9.40E-04 | | | | | 2.30E-02 | | | | |  |
| rs10895276 | | | | | T | | | C | | -8.37E-03 | | | 9.88E-04 | | | | | 5.00E-18 | | | | |  |
| rs10920678 | | | | | G | | | A | | 2.15E-03 | | | 9.36E-04 | | | | | 8.60E-03 | | | | |  |
| rs10938397 | | | | | G | | | A | | -3.91E-03 | | | 9.41E-04 | | | | | 3.20E-05 | | | | |  |
| rs10942267 | | | | | G | | | A | | 2.67E-03 | | | 1.00E-03 | | | | | 3.20E-03 | | | | |  |
| rs10953740 | | | | | G | | | A | | 2.50E-03 | | | 9.37E-04 | | | | | 5.60E-03 | | | | |  |
| rs10968114 | | | | | C | | | A | | 1.39E-04 | | | 9.38E-04 | | | | | 9.50E-01 | | | | |  |
| rs10971709 | | | | | T | | | C | | -9.40E-04 | | | 1.15E-03 | | | | | 2.00E-01 | | | | |  |
| rs11030618 | | | | | T | | | C | | -9.59E-04 | | | 9.43E-04 | | | | | 4.60E-01 | | | | |  |
| rs1105977 | | | | | T | | | G | | 6.10E-04 | | | 1.05E-03 | | | | | 9.10E-01 | | | | |  |
| rs11084553 | | | | | G | | | A | | 5.27E-05 | | | 1.29E-03 | | | | | 7.00E-01 | | | | |  |
| rs11115176 | | | | | C | | | T | | 2.08E-03 | | | 1.10E-03 | | | | | 6.00E-02 | | | | |  |
| rs1112613 | | | | | A | | | G | | 3.70E-03 | | | 1.22E-03 | | | | | 1.20E-03 | | | | |  |
| rs11155787 | | | | | T | | | C | | -5.38E-03 | | | 9.72E-04 | | | | | 9.00E-10 | | | | |  |
| rs11165643 | | | | | T | | | C | | -3.57E-03 | | | 9.43E-04 | | | | | 9.40E-05 | | | | |  |
| rs11170468 | | | | | C | | | A | | 1.88E-03 | | | 1.10E-03 | | | | | 4.30E-02 | | | | |  |
| rs11251352 | | | | | G | | | A | | -4.07E-03 | | | 9.51E-04 | | | | | 1.50E-05 | | | | |  |
| rs1144387 | | | | | C | | | G | | 7.34E-04 | | | 9.48E-04 | | | | | 5.20E-01 | | | | |  |
| rs11505821 | | | | | T | | | A | | -5.02E-03 | | | 1.90E-03 | | | | | 3.00E-02 | | | | |  |
| rs11556924 | | | | | T | | | C | | 8.82E-03 | | | 9.56E-04 | | | | | 1.80E-19 | | | | |  |
| rs1158805 | | | | | A | | | C | | 2.71E-03 | | | 9.69E-04 | | | | | 1.40E-02 | | | | |  |
| rs11621792 | | | | | C | | | T | | 1.83E-02 | | | 9.44E-04 | | | | | 6.90E-90 | | | | |  |
| rs11640366 | | | | | A | | | C | | -1.14E-02 | | | 9.37E-04 | | | | | 7.40E-36 | | | | |  |
| rs11656076 | | | | | A | | | G | | 1.30E-03 | | | 1.09E-03 | | | | | 1.20E-01 | | | | |  |
| rs11713193 | | | | | A | | | G | | -8.48E-03 | | | 9.32E-04 | | | | | 7.90E-21 | | | | |  |
| rs11738695 | | | | | A | | | C | | -1.43E-03 | | | 9.48E-04 | | | | | 2.50E-01 | | | | |  |
| rs117589665 | | | | | G | | | A | | -1.51E-02 | | | 1.85E-03 | | | | | 5.80E-17 | | | | |  |
| rs11765639 | | | | | A | | | G | | -7.56E-03 | | | 9.61E-04 | | | | | 1.30E-15 | | | | |  |
| rs11855853 | | | | | T | | | C | | 2.99E-03 | | | 1.06E-03 | | | | | 5.60E-03 | | | | |  |
| rs1187352 | | | | | C | | | T | | -2.00E-03 | | | 9.82E-04 | | | | | 3.20E-02 | | | | |  |
| rs11880870 | | | | | G | | | A | | 3.72E-03 | | | 9.34E-04 | | | | | 5.70E-05 | | | | |  |
| rs12044597 | | | | | G | | | A | | -2.33E-03 | | | 9.28E-04 | | | | | 8.20E-02 | | | | |  |
| rs12049202 | | | | | T | | | C | | 2.25E-03 | | | 1.16E-03 | | | | | 1.30E-01 | | | | |  |
| rs12098284 | | | | | T | | | C | | -4.67E-03 | | | 1.41E-03 | | | | | 1.10E-03 | | | | |  |
| rs12150665 | | | | | C | | | T | | -1.84E-03 | | | 9.22E-04 | | | | | 2.90E-02 | | | | |  |
| rs1218822 | | | | | A | | | G | | -3.76E-03 | | | 9.90E-04 | | | | | 3.40E-05 | | | | |  |
| rs12299814 | | | | | A | | | C | | 1.99E-03 | | | 1.08E-03 | | | | | 4.20E-02 | | | | |  |
| rs12320328 | | | | | G | | | A | | -2.37E-02 | | | 1.68E-03 | | | | | 1.10E-49 | | | | |  |
| rs12364470 | | | | | G | | | T | | -3.39E-03 | | | 1.26E-03 | | | | | 2.40E-02 | | | | |  |
| rs1241986 | | | | | A | | | G | | 3.55E-03 | | | 1.32E-03 | | | | | 1.60E-02 | | | | |  |
| rs12429545 | | | | | A | | | G | | -3.97E-03 | | | 1.41E-03 | | | | | 6.00E-03 | | | | |  |
| rs12448257 | | | | | A | | | G | | -4.93E-03 | | | 1.14E-03 | | | | | 7.10E-06 | | | | |  |
| rs12543287 | | | | | C | | | G | | 1.06E-02 | | | 9.71E-04 | | | | | 1.50E-30 | | | | |  |
| rs12629015 | | | | | G | | | A | | 2.01E-03 | | | 1.24E-03 | | | | | 2.20E-01 | | | | |  |
| rs1266874 | | | | | G | | | A | | -2.18E-03 | | | 9.79E-04 | | | | | 2.90E-02 | | | | |  |
| rs12675063 | | | | | T | | | A | | -2.43E-03 | | | 1.46E-03 | | | | | 8.20E-02 | | | | |  |
| rs1268065 | | | | | A | | | G | | 3.39E-03 | | | 9.40E-04 | | | | | 2.80E-04 | | | | |  |
| rs12680842 | | | | | G | | | A | | 1.60E-03 | | | 1.00E-03 | | | | | 7.90E-02 | | | | |  |
| rs12696304 | | | | | G | | | C | | 6.77E-03 | | | 1.06E-03 | | | | | 1.60E-10 | | | | |  |
| rs12718572 | | | | | T | | | C | | 3.09E-03 | | | 9.53E-04 | | | | | 1.70E-03 | | | | |  |
| rs12762034 | | | | | C | | | T | | -8.60E-03 | | | 1.74E-03 | | | | | 1.60E-07 | | | | |  |
| rs12914489 | | | | | A | | | G | | -5.78E-03 | | | 1.51E-03 | | | | | 8.00E-04 | | | | |  |
| rs12939549 | | | | | G | | | A | | 1.45E-03 | | | 9.11E-04 | | | | | 1.40E-01 | | | | |  |
| rs1296328 | | | | | C | | | A | | 1.89E-03 | | | 9.42E-04 | | | | | 1.70E-02 | | | | |  |
| rs13021737 | | | | | G | | | A | | -5.33E-03 | | | 1.23E-03 | | | | | 1.40E-05 | | | | |  |
| rs13035806 | | | | | A | | | G | | -8.91E-03 | | | 1.45E-03 | | | | | 2.20E-10 | | | | |  |
| rs13047416 | | | | | G | | | C | | 1.19E-03 | | | 9.71E-04 | | | | | 3.00E-01 | | | | |  |
| rs13069244 | | | | | A | | | G | | -1.56E-05 | | | 1.78E-03 | | | | | 8.30E-01 | | | | |  |
| rs13094241 | | | | | G | | | T | | 7.99E-03 | | | 1.05E-03 | | | | | 3.40E-15 | | | | |  |
| rs13107325 | | | | | T | | | C | | -1.63E-03 | | | 1.77E-03 | | | | | 3.40E-01 | | | | |  |
| rs13110266 | | | | | A | | | G | | 8.78E-04 | | | 9.46E-04 | | | | | 2.40E-01 | | | | |  |
| rs13150068 | | | | | G | | | A | | -1.88E-02 | | | 9.40E-04 | | | | | 7.30E-95 | | | | |  |
| rs13174863 | | | | | G | | | A | | -2.46E-03 | | | 1.32E-03 | | | | | 7.30E-02 | | | | |  |
| rs13191362 | | | | | G | | | A | | 5.35E-03 | | | 1.42E-03 | | | | | 2.00E-04 | | | | |  |
| rs1320903 | | | | | A | | | G | | -2.89E-03 | | | 1.00E-03 | | | | | 5.90E-03 | | | | |  |
| rs1321432 | | | | | C | | | A | | -1.22E-03 | | | 9.73E-04 | | | | | 3.50E-01 | | | | |  |
| rs13251458 | | | | | A | | | G | | 9.55E-03 | | | 9.36E-04 | | | | | 9.20E-27 | | | | |  |
| rs1327259 | | | | | G | | | A | | 2.47E-03 | | | 9.63E-04 | | | | | 5.10E-03 | | | | |  |
| rs13389219 | | | | | T | | | C | | 1.37E-02 | | | 9.49E-04 | | | | | 6.40E-53 | | | | |  |
| rs1365466 | | | | | T | | | C | | 2.67E-03 | | | 1.08E-03 | | | | | 1.10E-02 | | | | |  |
| rs13702 | | | | | C | | | T | | 2.70E-03 | | | 1.03E-03 | | | | | 7.40E-03 | | | | |  |
| rs1371108 | | | | | A | | | C | | -2.44E-03 | | | 9.97E-04 | | | | | 1.10E-02 | | | | |  |
| rs1409818 | | | | | T | | | C | | -2.85E-03 | | | 1.55E-03 | | | | | 8.30E-02 | | | | |  |
| rs1412235 | | | | | C | | | G | | -3.67E-03 | | | 9.97E-04 | | | | | 3.20E-05 | | | | |  |
| rs1421334 | | | | | C | | | A | | 2.58E-03 | | | 9.43E-04 | | | | | 1.20E-02 | | | | |  |
| rs1452075 | | | | | T | | | C | | -2.51E-03 | | | 1.05E-03 | | | | | 1.40E-02 | | | | |  |
| rs1454687 | | | | | G | | | C | | 4.68E-03 | | | 9.31E-04 | | | | | 2.40E-06 | | | | |  |
| rs1465900 | | | | | C | | | A | | 2.25E-03 | | | 1.15E-03 | | | | | 1.80E-02 | | | | |  |
| rs1472169 | | | | | T | | | C | | 2.42E-03 | | | 9.55E-04 | | | | | 3.00E-03 | | | | |  |
| rs150449323 | | | | | C | | | T | | -1.06E-02 | | | 1.61E-03 | | | | | 6.70E-12 | | | | |  |
| rs1521527 | | | | | C | | | G | | -1.42E-03 | | | 9.35E-04 | | | | | 6.90E-02 | | | | |  |
| rs1528435 | | | | | T | | | C | | -2.30E-03 | | | 9.57E-04 | | | | | 2.10E-02 | | | | |  |
| rs1534696 | | | | | A | | | C | | 5.21E-03 | | | 9.34E-04 | | | | | 1.10E-08 | | | | |  |
| rs1561442 | | | | | A | | | G | | -7.50E-03 | | | 1.36E-03 | | | | | 1.80E-08 | | | | |  |
| rs157935 | | | | | G | | | T | | 1.10E-02 | | | 1.02E-03 | | | | | 5.70E-29 | | | | |  |
| rs1624134 | | | | | C | | | G | | -1.62E-03 | | | 9.50E-04 | | | | | 1.90E-01 | | | | |  |
| rs1640269 | | | | | C | | | A | | 1.95E-02 | | | 1.03E-03 | | | | | 2.10E-85 | | | | |  |
| rs1650548 | | | | | C | | | G | | -9.56E-03 | | | 1.11E-03 | | | | | 2.10E-18 | | | | |  |
| rs1656377 | | | | | C | | | T | | -4.04E-04 | | | 9.46E-04 | | | | | 4.30E-01 | | | | |  |
| rs1681740 | | | | | C | | | A | | 3.47E-03 | | | 9.55E-04 | | | | | 9.10E-05 | | | | |  |
| rs16849710 | | | | | G | | | A | | 1.57E-04 | | | 9.34E-04 | | | | | 7.40E-01 | | | | |  |
| rs16851483 | | | | | T | | | G | | -3.15E-03 | | | 1.88E-03 | | | | | 7.10E-02 | | | | |  |
| rs16903285 | | | | | C | | | T | | -4.97E-03 | | | 1.39E-03 | | | | | 5.10E-04 | | | | |  |
| rs16942887 | | | | | A | | | G | | -3.24E-03 | | | 1.47E-03 | | | | | 1.20E-02 | | | | |  |
| rs17014375 | | | | | G | | | T | | -3.77E-03 | | | 1.37E-03 | | | | | 1.60E-02 | | | | |  |
| rs17033117 | | | | | T | | | C | | -8.84E-04 | | | 1.22E-03 | | | | | 6.00E-01 | | | | |  |
| rs17056301 | | | | | C | | | T | | 3.97E-03 | | | 1.07E-03 | | | | | 2.90E-04 | | | | |  |
| rs17119937 | | | | | C | | | T | | -5.21E-03 | | | 1.89E-03 | | | | | 6.80E-03 | | | | |  |
| rs17184382 | | | | | C | | | A | | 1.24E-02 | | | 9.43E-04 | | | | | 3.80E-41 | | | | |  |
| rs17207196 | | | | | T | | | C | | 5.93E-04 | | | 9.44E-04 | | | | | 4.60E-01 | | | | |  |
| rs1730862 | | | | | A | | | G | | -2.37E-02 | | | 9.83E-04 | | | | | 5.11E-133 | | | | |  |
| rs17399237 | | | | | C | | | T | | 2.52E-03 | | | 9.35E-04 | | | | | 7.90E-03 | | | | |  |
| rs17425707 | | | | | C | | | T | | -4.04E-03 | | | 1.52E-03 | | | | | 1.10E-02 | | | | |  |
| rs17535749 | | | | | A | | | G | | -3.79E-03 | | | 1.50E-03 | | | | | 6.40E-03 | | | | |  |
| rs17551974 | | | | | A | | | C | | 8.48E-04 | | | 1.19E-03 | | | | | 5.90E-01 | | | | |  |
| rs1772189 | | | | | A | | | T | | -1.15E-02 | | | 9.28E-04 | | | | | 6.70E-39 | | | | |  |
| rs17789218 | | | | | C | | | T | | 4.18E-03 | | | 1.09E-03 | | | | | 5.30E-06 | | | | |  |
| rs1801282 | | | | | G | | | C | | 1.83E-02 | | | 1.43E-03 | | | | | 1.30E-37 | | | | |  |
| rs1863652 | | | | | A | | | G | | 3.15E-03 | | | 9.76E-04 | | | | | 1.10E-03 | | | | |  |
| rs1866956 | | | | | T | | | C | | 9.83E-04 | | | 1.00E-03 | | | | | 4.10E-01 | | | | |  |
| rs1883025 | | | | | T | | | C | | 1.05E-03 | | | 1.07E-03 | | | | | 3.10E-01 | | | | |  |
| rs1891216 | | | | | G | | | T | | -4.19E-03 | | | 9.61E-04 | | | | | 1.50E-05 | | | | |  |
| rs189595752 | | | | | G | | | A | | 1.17E-02 | | | 1.50E-03 | | | | | 4.90E-15 | | | | |  |
| rs1928295 | | | | | C | | | T | | 7.81E-04 | | | 9.42E-04 | | | | | 3.20E-01 | | | | |  |
| rs1982725 | | | | | T | | | C | | -2.30E-03 | | | 9.40E-04 | | | | | 9.50E-03 | | | | |  |
| rs200810 | | | | | C | | | T | | 2.77E-03 | | | 9.65E-04 | | | | | 1.20E-03 | | | | |  |
| rs2009416 | | | | | T | | | C | | 2.76E-03 | | | 9.72E-04 | | | | | 7.20E-03 | | | | |  |
| rs2065418 | | | | | G | | | T | | 1.55E-03 | | | 9.71E-04 | | | | | 9.70E-02 | | | | |  |
| rs215634 | | | | | G | | | A | | 2.44E-03 | | | 9.61E-04 | | | | | 5.20E-03 | | | | |  |
| rs2174307 | | | | | C | | | G | | -2.03E-03 | | | 9.45E-04 | | | | | 7.10E-03 | | | | |  |
| rs217671 | | | | | G | | | A | | -4.27E-03 | | | 1.05E-03 | | | | | 4.20E-05 | | | | |  |
| rs2224585 | | | | | A | | | G | | 5.85E-03 | | | 1.09E-03 | | | | | 1.30E-08 | | | | |  |
| rs2241210 | | | | | G | | | A | | -1.78E-03 | | | 9.30E-04 | | | | | 2.70E-02 | | | | |  |
| rs2246012 | | | | | C | | | T | | -4.93E-03 | | | 1.25E-03 | | | | | 1.70E-04 | | | | |  |
| rs2325036 | | | | | C | | | A | | 2.49E-03 | | | 9.62E-04 | | | | | 1.70E-02 | | | | |  |
| rs2351958 | | | | | A | | | C | | -1.04E-02 | | | 9.58E-04 | | | | | 2.20E-31 | | | | |  |
| rs2357760 | | | | | A | | | G | | -5.71E-04 | | | 1.00E-03 | | | | | 4.70E-01 | | | | |  |
| rs2393791 | | | | | T | | | C | | 1.55E-02 | | | 9.60E-04 | | | | | 7.10E-61 | | | | |  |
| rs2479958 | | | | | G | | | A | | 1.98E-03 | | | 9.47E-04 | | | | | 2.80E-02 | | | | |  |
| rs2481665 | | | | | C | | | T | | 2.31E-03 | | | 9.33E-04 | | | | | 1.40E-03 | | | | |  |
| rs2498786 | | | | | G | | | C | | -1.09E-02 | | | 9.62E-04 | | | | | 1.40E-30 | | | | |  |
| rs2543132 | | | | | C | | | G | | -1.42E-03 | | | 1.20E-03 | | | | | 1.40E-01 | | | | |  |
| rs2551644 | | | | | A | | | T | | -1.20E-02 | | | 1.20E-03 | | | | | 2.30E-25 | | | | |  |
| rs2608703 | | | | | A | | | C | | -1.79E-03 | | | 9.34E-04 | | | | | 1.20E-02 | | | | |  |
| rs2612038 | | | | | T | | | C | | -9.85E-03 | | | 1.59E-03 | | | | | 7.20E-09 | | | | |  |
| rs2642438 | | | | | G | | | A | | 1.21E-02 | | | 1.01E-03 | | | | | 2.00E-34 | | | | |  |
| rs2643452 | | | | | A | | | T | | -7.39E-05 | | | 9.35E-04 | | | | | 6.40E-01 | | | | |  |
| rs2693826 | | | | | A | | | G | | 6.98E-05 | | | 9.36E-04 | | | | | 7.60E-01 | | | | |  |
| rs2694047 | | | | | G | | | A | | -3.16E-03 | | | 1.08E-03 | | | | | 4.80E-04 | | | | |  |
| rs273504 | | | | | G | | | A | | -7.02E-03 | | | 9.45E-04 | | | | | 2.40E-15 | | | | |  |
| rs2744974 | | | | | T | | | C | | 6.08E-03 | | | 9.96E-04 | | | | | 1.10E-09 | | | | |  |
| rs2791644 | | | | | C | | | T | | 7.04E-03 | | | 1.10E-03 | | | | | 2.20E-10 | | | | |  |
| rs28507491 | | | | | A | | | G | | 1.25E-02 | | | 9.60E-04 | | | | | 1.90E-38 | | | | |  |
| rs2861683 | | | | | C | | | A | | 2.79E-03 | | | 9.40E-04 | | | | | 7.80E-04 | | | | |  |
| rs2868975 | | | | | A | | | G | | 2.69E-03 | | | 1.22E-03 | | | | | 1.60E-02 | | | | |  |
| rs2875762 | | | | | C | | | G | | -1.82E-03 | | | 1.10E-03 | | | | | 3.30E-02 | | | | |  |
| rs2931434 | | | | | T | | | C | | 1.70E-03 | | | 9.89E-04 | | | | | 9.80E-02 | | | | |  |
| rs2972145 | | | | | C | | | T | | -1.35E-02 | | | 9.69E-04 | | | | | 1.00E-46 | | | | |  |
| rs3001032 | | | | | C | | | T | | 9.19E-03 | | | 9.94E-04 | | | | | 1.70E-20 | | | | |  |
| rs34255979 | | | | | T | | | C | | 2.71E-02 | | | 1.44E-03 | | | | | 9.10E-82 | | | | |  |
| rs349088 | | | | | A | | | C | | 1.74E-03 | | | 9.37E-04 | | | | | 4.70E-02 | | | | |  |
| rs35350976 | | | | | G | | | A | | 8.23E-03 | | | 1.21E-03 | | | | | 1.50E-13 | | | | |  |
| rs355777 | | | | | C | | | G | | -2.28E-03 | | | 9.50E-04 | | | | | 5.50E-02 | | | | |  |
| rs3749897 | | | | | T | | | C | | -1.25E-03 | | | 9.48E-04 | | | | | 1.80E-01 | | | | |  |
| rs3754963 | | | | | T | | | A | | 1.95E-03 | | | 1.06E-03 | | | | | 5.60E-02 | | | | |  |
| rs3772882 | | | | | A | | | C | | -5.13E-03 | | | 9.62E-04 | | | | | 1.30E-07 | | | | |  |
| rs3800229 | | | | | T | | | G | | -4.80E-03 | | | 1.04E-03 | | | | | 1.30E-06 | | | | |  |
| rs3806114 | | | | | A | | | G | | 2.50E-03 | | | 1.00E-03 | | | | | 3.90E-03 | | | | |  |
| rs3806572 | | | | | A | | | G | | 5.71E-03 | | | 1.02E-03 | | | | | 7.70E-08 | | | | |  |
| rs3807645 | | | | | A | | | G | | 2.96E-03 | | | 1.12E-03 | | | | | 3.40E-03 | | | | |  |
| rs380857 | | | | | A | | | C | | 1.29E-03 | | | 1.47E-03 | | | | | 3.90E-01 | | | | |  |
| rs3810027 | | | | | G | | | C | | -7.14E-03 | | | 9.94E-04 | | | | | 2.00E-13 | | | | |  |
| rs3829639 | | | | | G | | | A | | -7.44E-03 | | | 1.00E-03 | | | | | 1.10E-13 | | | | |  |
| rs38314 | | | | | A | | | G | | -1.16E-03 | | | 9.33E-04 | | | | | 3.10E-01 | | | | |  |
| rs3902951 | | | | | G | | | T | | -3.53E-03 | | | 1.11E-03 | | | | | 5.30E-03 | | | | |  |
| rs3904244 | | | | | A | | | T | | -2.36E-03 | | | 1.35E-03 | | | | | 1.80E-01 | | | | |  |
| rs40067 | | | | | A | | | G | | 5.28E-03 | | | 1.24E-03 | | | | | 2.10E-05 | | | | |  |
| rs40270 | | | | | C | | | A | | -1.67E-02 | | | 1.11E-03 | | | | | 6.80E-51 | | | | |  |
| rs4072917 | | | | | A | | | G | | -3.78E-03 | | | 9.38E-04 | | | | | 2.30E-05 | | | | |  |
| rs4148005 | | | | | G | | | T | | 3.51E-03 | | | 9.76E-04 | | | | | 2.20E-04 | | | | |  |
| rs4148155 | | | | | G | | | A | | 2.27E-03 | | | 1.46E-03 | | | | | 1.60E-01 | | | | |  |
| rs4237643 | | | | | G | | | T | | 4.45E-03 | | | 1.01E-03 | | | | | 6.30E-07 | | | | |  |
| rs4240624 | | | | | A | | | G | | 2.16E-02 | | | 1.62E-03 | | | | | 1.10E-44 | | | | |  |
| rs427943 | | | | | C | | | A | | -5.78E-03 | | | 9.48E-04 | | | | | 3.70E-10 | | | | |  |
| rs4307239 | | | | | G | | | A | | -2.05E-03 | | | 9.37E-04 | | | | | 1.30E-02 | | | | |  |
| rs4338849 | | | | | A | | | G | | 1.48E-02 | | | 9.08E-04 | | | | | 9.00E-61 | | | | |  |
| rs4430672 | | | | | C | | | T | | -3.92E-05 | | | 1.17E-03 | | | | | 7.80E-01 | | | | |  |
| rs4556997 | | | | | A | | | C | | -2.21E-03 | | | 1.36E-03 | | | | | 6.90E-02 | | | | |  |
| rs4567095 | | | | | T | | | C | | 7.49E-03 | | | 1.01E-03 | | | | | 1.10E-15 | | | | |  |
| rs4568281 | | | | | A | | | G | | 8.18E-03 | | | 9.97E-04 | | | | | 4.10E-16 | | | | |  |
| rs4589691 | | | | | G | | | C | | -4.86E-03 | | | 1.30E-03 | | | | | 1.50E-04 | | | | |  |
| rs4639527 | | | | | G | | | A | | -5.75E-04 | | | 1.00E-03 | | | | | 7.50E-01 | | | | |  |
| rs4639796 | | | | | A | | | G | | -1.40E-02 | | | 1.26E-03 | | | | | 1.70E-28 | | | | |  |
| rs4660443 | | | | | T | | | C | | -1.38E-02 | | | 1.13E-03 | | | | | 2.00E-35 | | | | |  |
| rs4665972 | | | | | C | | | T | | 3.49E-02 | | | 9.54E-04 | | | | | 0.00E+00 | | | | |  |
| rs4671328 | | | | | G | | | T | | 6.66E-03 | | | 9.40E-04 | | | | | 1.40E-14 | | | | |  |
| rs4674669 | | | | | T | | | C | | -6.85E-03 | | | 1.34E-03 | | | | | 2.30E-08 | | | | |  |
| rs4740619 | | | | | C | | | T | | 3.34E-03 | | | 9.39E-04 | | | | | 1.20E-04 | | | | |  |
| rs4800191 | | | | | C | | | G | | -1.70E-03 | | | 9.82E-04 | | | | | 3.10E-02 | | | | |  |
| rs4804414 | | | | | T | | | C | | -1.46E-02 | | | 9.42E-04 | | | | | 1.20E-58 | | | | |  |
| rs4812336 | | | | | A | | | G | | 5.96E-03 | | | 1.02E-03 | | | | | 1.20E-09 | | | | |  |
| rs4813619 | | | | | T | | | G | | -6.25E-04 | | | 9.44E-04 | | | | | 3.00E-01 | | | | |  |
| rs4820091 | | | | | G | | | T | | 1.05E-02 | | | 1.22E-03 | | | | | 2.80E-18 | | | | |  |
| rs4820408 | | | | | G | | | T | | 3.94E-03 | | | 9.55E-04 | | | | | 8.60E-04 | | | | |  |
| rs4842491 | | | | | T | | | C | | 4.27E-05 | | | 1.03E-03 | | | | | 8.20E-01 | | | | |  |
| rs4846914 | | | | | A | | | G | | 3.80E-03 | | | 9.50E-04 | | | | | 1.60E-03 | | | | |  |
| rs4851029 | | | | | G | | | T | | -2.77E-03 | | | 9.29E-04 | | | | | 1.10E-03 | | | | |  |
| rs4880341 | | | | | T | | | C | | 4.11E-03 | | | 9.41E-04 | | | | | 1.50E-04 | | | | |  |
| rs4936175 | | | | | C | | | T | | -5.76E-04 | | | 9.38E-04 | | | | | 7.90E-01 | | | | |  |
| rs4939883 | | | | | C | | | T | | -1.42E-03 | | | 1.22E-03 | | | | | 2.20E-01 | | | | |  |
| rs4954638 | | | | | C | | | A | | 9.93E-04 | | | 1.09E-03 | | | | | 2.20E-01 | | | | |  |
| rs538579 | | | | | C | | | G | | -2.35E-03 | | | 1.00E-03 | | | | | 1.10E-02 | | | | |  |
| rs543874 | | | | | G | | | A | | 1.43E-03 | | | 1.15E-03 | | | | | 2.30E-01 | | | | |  |
| rs559231 | | | | | T | | | G | | -2.16E-03 | | | 9.63E-04 | | | | | 3.00E-02 | | | | |  |
| rs56024084 | | | | | T | | | C | | 1.03E-02 | | | 9.53E-04 | | | | | 3.30E-26 | | | | |  |
| rs56077345 | | | | | C | | | G | | 9.66E-03 | | | 1.82E-03 | | | | | 4.00E-08 | | | | |  |
| rs587252 | | | | | C | | | A | | -1.09E-02 | | | 1.66E-03 | | | | | 6.20E-10 | | | | |  |
| rs61856602 | | | | | G | | | A | | 5.63E-03 | | | 9.91E-04 | | | | | 4.00E-08 | | | | |  |
| rs6235 | | | | | G | | | C | | -1.38E-03 | | | 1.05E-03 | | | | | 2.00E-01 | | | | |  |
| rs62623385 | | | | | T | | | A | | -8.51E-02 | | | 2.49E-03 | | | | | 1.30E-259 | | | | |  |
| rs6265 | | | | | T | | | C | | 5.95E-03 | | | 1.19E-03 | | | | | 4.70E-07 | | | | |  |
| rs633695 | | | | | G | | | A | | -1.81E-03 | | | 1.03E-03 | | | | | 5.20E-02 | | | | |  |
| rs6471941 | | | | | A | | | G | | -1.80E-03 | | | 1.25E-03 | | | | | 1.30E-01 | | | | |  |
| rs6486122 | | | | | T | | | C | | -1.38E-02 | | | 1.01E-03 | | | | | 1.50E-44 | | | | |  |
| rs6512302 | | | | | C | | | G | | 7.37E-05 | | | 1.08E-03 | | | | | 9.10E-01 | | | | |  |
| rs6545714 | | | | | A | | | G | | 2.06E-03 | | | 9.48E-04 | | | | | 1.90E-02 | | | | |  |
| rs6561943 | | | | | T | | | C | | -1.92E-03 | | | 1.07E-03 | | | | | 3.00E-02 | | | | |  |
| rs657452 | | | | | G | | | A | | 6.53E-04 | | | 9.53E-04 | | | | | 5.20E-01 | | | | |  |
| rs6591407 | | | | | A | | | C | | 4.07E-04 | | | 1.21E-03 | | | | | 5.50E-01 | | | | |  |
| rs6595205 | | | | | G | | | C | | 2.62E-03 | | | 9.36E-04 | | | | | 4.70E-04 | | | | |  |
| rs663129 | | | | | A | | | G | | -9.66E-03 | | | 1.11E-03 | | | | | 2.50E-19 | | | | |  |
| rs668871 | | | | | T | | | C | | 1.61E-02 | | | 9.37E-04 | | | | | 1.70E-76 | | | | |  |
| rs6758199 | | | | | T | | | C | | -1.80E-02 | | | 1.87E-03 | | | | | 5.20E-23 | | | | |  |
| rs6785245 | | | | | C | | | T | | -2.25E-03 | | | 9.49E-04 | | | | | 3.50E-02 | | | | |  |
| rs6815910 | | | | | A | | | T | | 2.75E-03 | | | 9.38E-04 | | | | | 5.80E-03 | | | | |  |
| rs6841761 | | | | | T | | | G | | 3.02E-03 | | | 9.35E-04 | | | | | 3.60E-03 | | | | |  |
| rs6860245 | | | | | C | | | G | | 9.97E-03 | | | 1.08E-03 | | | | | 1.90E-20 | | | | |  |
| rs686030 | | | | | A | | | C | | 2.87E-03 | | | 1.34E-03 | | | | | 2.20E-02 | | | | |  |
| rs695272 | | | | | C | | | T | | -1.29E-02 | | | 9.93E-04 | | | | | 1.20E-38 | | | | |  |
| rs7015 | | | | | A | | | G | | -3.50E-02 | | | 1.20E-03 | | | | | 1.50E-195 | | | | |  |
| rs7025938 | | | | | G | | | C | | -2.76E-03 | | | 1.01E-03 | | | | | 1.20E-02 | | | | |  |
| rs705704 | | | | | A | | | G | | 2.99E-03 | | | 9.82E-04 | | | | | 2.50E-03 | | | | |  |
| rs7084454 | | | | | A | | | G | | 5.02E-04 | | | 9.96E-04 | | | | | 4.30E-01 | | | | |  |
| rs7096764 | | | | | A | | | G | | 5.78E-03 | | | 1.02E-03 | | | | | 2.60E-08 | | | | |  |
| rs7117238 | | | | | A | | | G | | 7.38E-03 | | | 1.28E-03 | | | | | 1.80E-08 | | | | |  |
| rs7138803 | | | | | A | | | G | | -1.31E-03 | | | 9.64E-04 | | | | | 1.20E-01 | | | | |  |
| rs7144011 | | | | | T | | | G | | -3.48E-03 | | | 1.13E-03 | | | | | 1.80E-04 | | | | |  |
| rs7148846 | | | | | G | | | T | | -2.14E-03 | | | 1.19E-03 | | | | | 4.10E-02 | | | | |  |
| rs7196720 | | | | | C | | | T | | -2.33E-03 | | | 9.36E-04 | | | | | 1.90E-02 | | | | |  |
| rs7222349 | | | | | A | | | G | | -3.19E-05 | | | 9.57E-04 | | | | | 7.80E-01 | | | | |  |
| rs7239575 | | | | | C | | | T | | 3.72E-03 | | | 9.38E-04 | | | | | 7.70E-05 | | | | |  |
| rs7262150 | | | | | C | | | T | | 9.29E-03 | | | 1.11E-03 | | | | | 1.00E-17 | | | | |  |
| rs73079476 | | | | | C | | | A | | -2.97E-02 | | | 1.30E-03 | | | | | 3.70E-120 | | | | |  |
| rs7314285 | | | | | G | | | T | | 3.46E-02 | | | 1.84E-03 | | | | | 5.40E-82 | | | | |  |
| rs7318817 | | | | | T | | | C | | 1.89E-03 | | | 9.68E-04 | | | | | 1.10E-01 | | | | |  |
| rs738409 | | | | | G | | | C | | 2.45E-02 | | | 1.14E-03 | | | | | 4.30E-104 | | | | |  |
| rs7451021 | | | | | C | | | T | | -9.35E-03 | | | 1.01E-03 | | | | | 3.10E-21 | | | | |  |
| rs7498665 | | | | | G | | | A | | -5.35E-03 | | | 9.55E-04 | | | | | 3.50E-08 | | | | |  |
| rs7557796 | | | | | C | | | T | | 2.09E-03 | | | 9.70E-04 | | | | | 1.40E-02 | | | | |  |
| rs757869 | | | | | G | | | A | | 1.13E-02 | | | 1.03E-03 | | | | | 1.20E-30 | | | | |  |
| rs7615297 | | | | | G | | | C | | 1.36E-03 | | | 1.30E-03 | | | | | 2.00E-01 | | | | |  |
| rs7626079 | | | | | T | | | C | | -2.99E-04 | | | 9.84E-04 | | | | | 6.60E-01 | | | | |  |
| rs7678138 | | | | | A | | | G | | -9.88E-03 | | | 1.41E-03 | | | | | 1.90E-13 | | | | |  |
| rs7683836 | | | | | A | | | G | | 2.91E-03 | | | 9.40E-04 | | | | | 1.70E-03 | | | | |  |
| rs7703576 | | | | | C | | | T | | -1.25E-03 | | | 1.02E-03 | | | | | 2.20E-01 | | | | |  |
| rs7715256 | | | | | T | | | G | | 2.01E-03 | | | 9.43E-04 | | | | | 3.10E-02 | | | | |  |
| rs7724675 | | | | | A | | | G | | 3.04E-03 | | | 1.13E-03 | | | | | 5.40E-03 | | | | |  |
| rs7730898 | | | | | A | | | G | | -1.07E-03 | | | 1.05E-03 | | | | | 2.90E-01 | | | | |  |
| rs7761673 | | | | | A | | | T | | 2.39E-03 | | | 1.13E-03 | | | | | 8.80E-02 | | | | |  |
| rs7780752 | | | | | C | | | T | | 1.78E-03 | | | 9.69E-04 | | | | | 2.90E-02 | | | | |  |
| rs7788008 | | | | | A | | | G | | 3.26E-03 | | | 9.40E-04 | | | | | 1.80E-03 | | | | |  |
| rs7819514 | | | | | A | | | G | | 3.31E-03 | | | 9.91E-04 | | | | | 7.40E-04 | | | | |  |
| rs7844647 | | | | | C | | | T | | 1.78E-03 | | | 1.06E-03 | | | | | 7.40E-02 | | | | |  |
| rs7860634 | | | | | A | | | G | | 9.91E-03 | | | 9.44E-04 | | | | | 2.70E-27 | | | | |  |
| rs7869771 | | | | | C | | | A | | 1.41E-03 | | | 1.04E-03 | | | | | 9.10E-02 | | | | |  |
| rs7925214 | | | | | T | | | C | | -3.19E-03 | | | 9.42E-04 | | | | | 2.30E-04 | | | | |  |
| rs796004 | | | | | T | | | C | | 2.35E-02 | | | 1.07E-03 | | | | | 1.80E-108 | | | | |  |
| rs79717793 | | | | | A | | | G | | -2.26E-02 | | | 1.28E-03 | | | | | 2.00E-78 | | | | |  |
| rs7983065 | | | | | T | | | C | | 5.28E-04 | | | 9.39E-04 | | | | | 5.30E-01 | | | | |  |
| rs802685 | | | | | C | | | T | | 6.32E-03 | | | 1.08E-03 | | | | | 2.50E-09 | | | | |  |
| rs8033077 | | | | | C | | | T | | 8.74E-03 | | | 1.04E-03 | | | | | 3.40E-18 | | | | |  |
| rs8047395 | | | | | A | | | G | | -7.59E-03 | | | 9.38E-04 | | | | | 4.60E-15 | | | | |  |
| rs806600 | | | | | G | | | A | | -7.03E-04 | | | 9.34E-04 | | | | | 4.90E-01 | | | | |  |
| rs8079418 | | | | | C | | | T | | 1.29E-02 | | | 9.30E-04 | | | | | 7.60E-44 | | | | |  |
| rs8097672 | | | | | T | | | A | | -2.86E-03 | | | 1.33E-03 | | | | | 3.90E-02 | | | | |  |
| rs8097783 | | | | | A | | | G | | 3.80E-03 | | | 1.80E-03 | | | | | 3.40E-02 | | | | |  |
| rs8181823 | | | | | C | | | A | | -1.06E-03 | | | 1.10E-03 | | | | | 1.30E-01 | | | | |  |
| rs865809 | | | | | G | | | A | | 2.37E-03 | | | 1.11E-03 | | | | | 3.20E-02 | | | | |  |
| rs872281 | | | | | T | | | C | | 1.50E-03 | | | 1.23E-03 | | | | | 3.50E-01 | | | | |  |
| rs876605 | | | | | G | | | A | | 3.11E-03 | | | 1.06E-03 | | | | | 6.50E-03 | | | | |  |
| rs879620 | | | | | T | | | C | | -2.27E-03 | | | 9.64E-04 | | | | | 7.00E-02 | | | | |  |
| rs889398 | | | | | T | | | C | | 3.83E-03 | | | 9.54E-04 | | | | | 3.40E-05 | | | | |  |
| rs901630 | | | | | T | | | C | | 5.15E-03 | | | 9.56E-04 | | | | | 1.80E-08 | | | | |  |
| rs902695 | | | | | A | | | G | | 9.65E-05 | | | 9.33E-04 | | | | | 8.40E-01 | | | | |  |
| rs9267551 | | | | | G | | | C | | -1.59E-02 | | | 1.32E-03 | | | | | 1.00E-29 | | | | |  |
| rs9294260 | | | | | A | | | G | | -3.45E-03 | | | 9.40E-04 | | | | | 1.60E-04 | | | | |  |
| rs9297994 | | | | | A | | | G | | 1.15E-02 | | | 9.89E-04 | | | | | 3.10E-31 | | | | |  |
| rs9300422 | | | | | G | | | A | | 9.64E-04 | | | 1.00E-03 | | | | | 3.80E-01 | | | | |  |
| rs9379084 | | | | | A | | | G | | -1.45E-02 | | | 1.50E-03 | | | | | 4.60E-23 | | | | |  |
| rs9408882 | | | | | A | | | G | | 7.93E-04 | | | 9.38E-04 | | | | | 4.70E-01 | | | | |  |
| rs946824 | | | | | C | | | T | | 2.90E-03 | | | 1.38E-03 | | | | | 1.50E-02 | | | | |  |
| rs947612 | | | | | A | | | G | | 2.55E-03 | | | 1.08E-03 | | | | | 2.40E-03 | | | | |  |
| rs9522285 | | | | | A | | | G | | -2.52E-03 | | | 9.50E-04 | | | | | 5.80E-03 | | | | |  |
| rs9538162 | | | | | C | | | T | | 4.02E-03 | | | 9.48E-04 | | | | | 2.10E-06 | | | | |  |
| rs9547153 | | | | | G | | | A | | -3.03E-03 | | | 9.77E-04 | | | | | 3.80E-03 | | | | |  |
| rs9571687 | | | | | A | | | C | | 1.14E-03 | | | 9.98E-04 | | | | | 9.50E-02 | | | | |  |
| rs9615905 | | | | | T | | | C | | -3.95E-03 | | | 9.40E-04 | | | | | 4.20E-05 | | | | |  |
| rs964184 | | | | | C | | | G | | -1.21E-03 | | | 1.37E-03 | | | | | 2.50E-01 | | | | |  |
| rs970548 | | | | | C | | | A | | -1.84E-03 | | | 1.07E-03 | | | | | 9.20E-02 | | | | |  |
| rs9739640 | | | | | G | | | A | | -1.05E-02 | | | 1.27E-03 | | | | | 2.00E-16 | | | | |  |
| rs977747 | | | | | G | | | T | | 1.77E-03 | | | 9.42E-04 | | | | | 1.10E-01 | | | | |  |
| rs9783858 | | | | | T | | | C | | 1.44E-03 | | | 9.42E-04 | | | | | 2.60E-01 | | | | |  |
| rs9806742 | | | | | A | | | G | | -2.45E-03 | | | 1.39E-03 | | | | | 5.20E-02 | | | | |  |
| rs9816226 | | | | | T | | | A | | -1.15E-03 | | | 1.20E-03 | | | | | 4.90E-01 | | | | |  |
| rs9845966 | | | | | G | | | T | | 1.57E-03 | | | 9.35E-04 | | | | | 1.20E-01 | | | | |  |
| rs9849171 | | | | | C | | | G | | 8.14E-03 | | | 9.70E-04 | | | | | 9.30E-18 | | | | |  |
| rs987237 | | | | | G | | | A | | -5.45E-03 | | | 1.22E-03 | | | | | 1.30E-06 | | | | |  |
| rs9927848 | | | | | A | | | C | | 4.05E-03 | | | 1.06E-03 | | | | | 1.50E-04 | | | | |  |
| rs9989419 | | | | | G | | | A | | 7.08E-04 | | | 9.55E-04 | | | | | 4.60E-01 | | | | |  |
| rs999889 | | | | | A | | | G | | 1.40E-03 | | | 1.03E-03 | | | | | 1.40E-01 | | | | |  |
| **HDL-C** | | | | |  | | |  | |  | | |  | | | | |  | | | | |  |
| rs10009336 | | | | | T | | | C | | 2.00E-03 | | | 4.60E-03 | | | | | 6.04E-01 | | | | |  |
| rs10027275 | | | | | C | | | G | | -8.80E-03 | | | 4.20E-03 | | | | | 1.86E-02 | | | | |  |
| rs10123811 | | | | | T | | | C | | 3.70E-03 | | | 4.90E-03 | | | | | 6.99E-01 | | | | |  |
| rs10132280 | | | | | A | | | C | | 8.60E-03 | | | 3.70E-03 | | | | | 3.85E-02 | | | | |  |
| rs10182181 | | | | | G | | | A | | -5.20E-03 | | | 3.40E-03 | | | | | 1.18E-01 | | | | |  |
| rs10197031 | | | | | C | | | T | | -8.20E-03 | | | 5.30E-03 | | | | | 7.06E-02 | | | | |  |
| rs10208512 | | | | | A | | | G | | 1.69E-02 | | | 1.20E-02 | | | | | 1.40E-01 | | | | |  |
| rs102275 | | | | | C | | | T | | -3.91E-02 | | | 3.50E-03 | | | | | 6.40E-28 | | | | |  |
| rs10238028 | | | | | G | | | A | | 8.80E-03 | | | 9.40E-03 | | | | | 2.33E-01 | | | | |  |
| rs10243319 | | | | | C | | | T | | 5.90E-03 | | | 4.90E-03 | | | | | 2.95E-01 | | | | |  |
| rs10245356 | | | | | T | | | C | | -2.70E-03 | | | 5.00E-03 | | | | | 6.17E-01 | | | | |  |
| rs10247983 | | | | | A | | | G | | -3.50E-03 | | | 9.00E-03 | | | | | 7.18E-01 | | | | |  |
| rs10248136 | | | | | T | | | C | | 1.90E-03 | | | 4.80E-03 | | | | | 3.92E-01 | | | | |  |
| rs10278546 | | | | | C | | | A | | 1.22E-02 | | | 6.30E-03 | | | | | 4.40E-02 | | | | |  |
| rs10468017 | | | | | T | | | C | | 1.18E-01 | | | 3.80E-03 | | | | | 1.21E-188 | | | | |  |
| rs10478110 | | | | | C | | | A | | -3.60E-03 | | | 4.90E-03 | | | | | 5.94E-01 | | | | |  |
| rs10742752 | | | | | C | | | T | | 2.00E-04 | | | 3.50E-03 | | | | | 9.48E-01 | | | | |  |
| rs10747488 | | | | | A | | | C | | 6.90E-03 | | | 5.70E-03 | | | | | 2.55E-01 | | | | |  |
| rs10768994 | | | | | C | | | T | | 6.30E-03 | | | 3.40E-03 | | | | | 9.04E-02 | | | | |  |
| rs10795422 | | | | | G | | | A | | -1.16E-02 | | | 5.20E-03 | | | | | 6.04E-02 | | | | |  |
| rs10808546 | | | | | T | | | C | | 4.09E-02 | | | 3.40E-03 | | | | | 4.11E-30 | | | | |  |
| rs10864070 | | | | | A | | | G | | 1.53E-02 | | | 8.40E-03 | | | | | 9.43E-02 | | | | |  |
| rs10867256 | | | | | T | | | C | | 1.01E-02 | | | 4.80E-03 | | | | | 1.09E-01 | | | | |  |
| rs10895276 | | | | | T | | | C | | 7.70E-03 | | | 5.00E-03 | | | | | 4.64E-01 | | | | |  |
| rs10920678 | | | | | G | | | A | | 4.00E-03 | | | 3.40E-03 | | | | | 2.17E-01 | | | | |  |
| rs10938397 | | | | | G | | | A | | -8.30E-03 | | | 3.60E-03 | | | | | 2.28E-02 | | | | |  |
| rs10942267 | | | | | G | | | A | | 5.70E-03 | | | 5.20E-03 | | | | | 2.33E-01 | | | | |  |
| rs10953740 | | | | | G | | | A | | 2.20E-03 | | | 4.80E-03 | | | | | 9.86E-01 | | | | |  |
| rs10968114 | | | | | C | | | A | | 1.10E-03 | | | 4.80E-03 | | | | | 8.56E-01 | | | | |  |
| rs10971709 | | | | | T | | | C | | 2.60E-03 | | | 5.90E-03 | | | | | 6.76E-01 | | | | |  |
| rs11030618 | | | | | T | | | C | | -4.00E-04 | | | 4.90E-03 | | | | | 6.99E-01 | | | | |  |
| rs1105977 | | | | | T | | | G | | -1.30E-03 | | | 6.30E-03 | | | | | 8.77E-01 | | | | |  |
| rs11084553 | | | | | G | | | A | | 3.30E-03 | | | 6.70E-03 | | | | | 9.70E-01 | | | | |  |
| rs11115176 | | | | | C | | | T | | 3.00E-04 | | | 4.10E-03 | | | | | 7.55E-01 | | | | |  |
| rs1112613 | | | | | A | | | G | | 7.80E-03 | | | 6.30E-03 | | | | | 4.53E-01 | | | | |  |
| rs11155787 | | | | | T | | | C | | 5.10E-03 | | | 5.10E-03 | | | | | 4.34E-01 | | | | |  |
| rs11165643 | | | | | T | | | C | | -4.00E-03 | | | 3.40E-03 | | | | | 2.46E-01 | | | | |  |
| rs11170468 | | | | | C | | | A | | 4.70E-03 | | | 4.00E-03 | | | | | 4.55E-01 | | | | |  |
| rs11251352 | | | | | G | | | A | | -2.60E-03 | | | 5.20E-03 | | | | | 9.66E-01 | | | | |  |
| rs1144387 | | | | | C | | | G | | -4.80E-03 | | | 4.80E-03 | | | | | 1.33E-01 | | | | |  |
| rs11505821 | | | | | T | | | A | | -4.70E-03 | | | 8.60E-03 | | | | | 6.83E-01 | | | | |  |
| rs11556924 | | | | | T | | | C | | 1.75E-02 | | | 3.60E-03 | | | | | 1.26E-05 | | | | |  |
| rs1158805 | | | | | A | | | C | | 7.50E-03 | | | 4.90E-03 | | | | | 1.37E-01 | | | | |  |
| rs11621792 | | | | | C | | | T | | 2.40E-03 | | | 3.80E-03 | | | | | 5.76E-01 | | | | |  |
| rs11640366 | | | | | A | | | C | | -3.60E-03 | | | 4.90E-03 | | | | | 4.43E-01 | | | | |  |
| rs11656076 | | | | | A | | | G | | 5.80E-03 | | | 5.70E-03 | | | | | 1.18E-01 | | | | |  |
| rs11713193 | | | | | A | | | G | | -1.96E-02 | | | 4.80E-03 | | | | | 9.34E-05 | | | | |  |
| rs11738695 | | | | | A | | | C | | -5.60E-03 | | | 4.80E-03 | | | | | 2.73E-01 | | | | |  |
| rs117589665 | | | | | G | | | A | | -1.07E-02 | | | 1.00E-02 | | | | | 5.23E-01 | | | | |  |
| rs11765639 | | | | | A | | | G | | 1.50E-03 | | | 5.10E-03 | | | | | 3.94E-01 | | | | |  |
| rs11855853 | | | | | T | | | C | | 2.50E-03 | | | 5.60E-03 | | | | | 7.69E-01 | | | | |  |
| rs1187352 | | | | | C | | | T | | -3.10E-03 | | | 5.00E-03 | | | | | 3.46E-01 | | | | |  |
| rs11880870 | | | | | G | | | A | | 4.70E-03 | | | 4.00E-03 | | | | | 3.09E-01 | | | | |  |
| rs12044597 | | | | | G | | | A | | -1.18E-02 | | | 3.50E-03 | | | | | 2.57E-03 | | | | |  |
| rs12049202 | | | | | T | | | C | | 9.00E-04 | | | 6.00E-03 | | | | | 6.87E-01 | | | | |  |
| rs12098284 | | | | | T | | | C | | 6.10E-03 | | | 7.40E-03 | | | | | 3.11E-01 | | | | |  |
| rs12150665 | | | | | C | | | T | | 1.30E-03 | | | 3.40E-03 | | | | | 6.44E-01 | | | | |  |
| rs1218822 | | | | | A | | | G | | 6.00E-04 | | | 3.60E-03 | | | | | 9.01E-01 | | | | |  |
| rs12299814 | | | | | A | | | C | | 1.02E-02 | | | 5.70E-03 | | | | | 3.97E-02 | | | | |  |
| rs12320328 | | | | | G | | | A | | -3.50E-03 | | | 6.80E-03 | | | | | 9.16E-01 | | | | |  |
| rs12364470 | | | | | G | | | T | | 3.00E-03 | | | 4.80E-03 | | | | | 7.10E-01 | | | | |  |
| rs1241986 | | | | | A | | | G | | 9.20E-03 | | | 6.80E-03 | | | | | 1.23E-01 | | | | |  |
| rs12429545 | | | | | A | | | G | | -1.64E-02 | | | 5.20E-03 | | | | | 3.89E-03 | | | | |  |
| rs12448257 | | | | | A | | | G | | 2.30E-03 | | | 4.30E-03 | | | | | 6.09E-01 | | | | |  |
| rs12543287 | | | | | C | | | G | | -6.70E-03 | | | 5.60E-03 | | | | | 3.47E-01 | | | | |  |
| rs12629015 | | | | | G | | | A | | 1.25E-02 | | | 6.30E-03 | | | | | 1.02E-02 | | | | |  |
| rs1266874 | | | | | G | | | A | | 1.90E-03 | | | 4.90E-03 | | | | | 3.89E-01 | | | | |  |
| rs12675063 | | | | | T | | | A | | -2.20E-03 | | | 5.70E-03 | | | | | 6.11E-01 | | | | |  |
| rs1268065 | | | | | A | | | G | | 5.50E-03 | | | 3.60E-03 | | | | | 9.87E-02 | | | | |  |
| rs12680842 | | | | | G | | | A | | 2.60E-03 | | | 3.60E-03 | | | | | 3.82E-01 | | | | |  |
| rs12696304 | | | | | G | | | C | | 6.90E-03 | | | 5.50E-03 | | | | | 3.88E-01 | | | | |  |
| rs12718572 | | | | | T | | | C | | -4.00E-04 | | | 4.90E-03 | | | | | 4.98E-01 | | | | |  |
| rs12762034 | | | | | C | | | T | | -6.90E-03 | | | 9.00E-03 | | | | | 1.30E-01 | | | | |  |
| rs12914489 | | | | | A | | | G | | 1.00E-04 | | | 5.60E-03 | | | | | 9.18E-01 | | | | |  |
| rs12939549 | | | | | G | | | A | | 2.80E-03 | | | 3.40E-03 | | | | | 2.26E-01 | | | | |  |
| rs1296328 | | | | | C | | | A | | -5.00E-04 | | | 5.00E-03 | | | | | 8.54E-01 | | | | |  |
| rs13021737 | | | | | G | | | A | | -1.25E-02 | | | 4.50E-03 | | | | | 8.27E-03 | | | | |  |
| rs13035806 | | | | | A | | | G | | -2.90E-03 | | | 7.60E-03 | | | | | 4.85E-01 | | | | |  |
| rs13047416 | | | | | G | | | C | | 2.40E-03 | | | 5.00E-03 | | | | | 5.80E-01 | | | | |  |
| rs13069244 | | | | | A | | | G | | -1.00E-03 | | | 6.80E-03 | | | | | 9.49E-01 | | | | |  |
| rs13094241 | | | | | G | | | T | | 4.80E-03 | | | 5.30E-03 | | | | | 4.56E-01 | | | | |  |
| rs13107325 | | | | | T | | | C | | -7.08E-02 | | | 7.80E-03 | | | | | 1.06E-15 | | | | |  |
| rs13110266 | | | | | A | | | G | | 1.16E-02 | | | 3.40E-03 | | | | | 4.61E-03 | | | | |  |
| rs13150068 | | | | | G | | | A | | -9.20E-03 | | | 4.80E-03 | | | | | 2.70E-02 | | | | |  |
| rs13174863 | | | | | G | | | A | | -1.00E-03 | | | 4.80E-03 | | | | | 4.53E-01 | | | | |  |
| rs13191362 | | | | | G | | | A | | 1.41E-02 | | | 5.40E-03 | | | | | 6.22E-03 | | | | |  |
| rs1320903 | | | | | A | | | G | | -1.41E-02 | | | 5.10E-03 | | | | | 7.43E-03 | | | | |  |
| rs1321432 | | | | | C | | | A | | 6.00E-03 | | | 5.00E-03 | | | | | 3.39E-01 | | | | |  |
| rs13251458 | | | | | A | | | G | | 2.00E-04 | | | 5.10E-03 | | | | | 6.29E-01 | | | | |  |
| rs1327259 | | | | | G | | | A | | 2.20E-03 | | | 5.00E-03 | | | | | 9.76E-01 | | | | |  |
| rs13389219 | | | | | T | | | C | | 2.26E-02 | | | 3.50E-03 | | | | | 4.54E-10 | | | | |  |
| rs1365466 | | | | | T | | | C | | 6.60E-03 | | | 3.90E-03 | | | | | 2.10E-01 | | | | |  |
| rs13702 | | | | | C | | | T | | 1.06E-01 | | | 3.80E-03 | | | | | 1.28E-160 | | | | |  |
| rs1371108 | | | | | A | | | C | | 4.00E-04 | | | 5.00E-03 | | | | | 6.09E-01 | | | | |  |
| rs1409818 | | | | | T | | | C | | -1.81E-02 | | | 8.00E-03 | | | | | 7.41E-02 | | | | |  |
| rs1412235 | | | | | C | | | G | | -5.10E-03 | | | 3.70E-03 | | | | | 1.75E-01 | | | | |  |
| rs1421334 | | | | | C | | | A | | 1.34E-02 | | | 4.90E-03 | | | | | 2.59E-02 | | | | |  |
| rs1452075 | | | | | T | | | C | | -7.10E-03 | | | 3.80E-03 | | | | | 7.92E-02 | | | | |  |
| rs1454687 | | | | | G | | | C | | 1.70E-03 | | | 4.80E-03 | | | | | 7.21E-01 | | | | |  |
| rs1465900 | | | | | C | | | A | | 9.30E-03 | | | 4.00E-03 | | | | | 2.70E-02 | | | | |  |
| rs1472169 | | | | | T | | | C | | 2.00E-04 | | | 4.80E-03 | | | | | 7.74E-01 | | | | |  |
| rs150449323 | | | | | C | | | T | | -2.98E-02 | | | 9.20E-03 | | | | | 8.54E-04 | | | | |  |
| rs1521527 | | | | | C | | | G | | -3.30E-03 | | | 4.80E-03 | | | | | 7.14E-01 | | | | |  |
| rs1528435 | | | | | T | | | C | | -3.70E-03 | | | 3.50E-03 | | | | | 2.19E-01 | | | | |  |
| rs1534696 | | | | | A | | | C | | 1.82E-02 | | | 3.70E-03 | | | | | 2.25E-06 | | | | |  |
| rs1561442 | | | | | A | | | G | | -7.00E-03 | | | 7.50E-03 | | | | | 1.97E-01 | | | | |  |
| rs157935 | | | | | G | | | T | | 1.30E-02 | | | 5.40E-03 | | | | | 2.21E-02 | | | | |  |
| rs1624134 | | | | | C | | | G | | -2.00E-03 | | | 5.00E-03 | | | | | 4.67E-01 | | | | |  |
| rs1640269 | | | | | C | | | A | | 1.38E-02 | | | 5.80E-03 | | | | | 1.14E-01 | | | | |  |
| rs1650548 | | | | | C | | | G | | -2.13E-02 | | | 5.90E-03 | | | | | 2.71E-04 | | | | |  |
| rs1656377 | | | | | C | | | T | | -9.00E-03 | | | 4.90E-03 | | | | | 8.86E-02 | | | | |  |
| rs1681740 | | | | | C | | | A | | 1.15E-02 | | | 5.00E-03 | | | | | 4.52E-03 | | | | |  |
| rs16849710 | | | | | G | | | A | | 2.00E-04 | | | 5.10E-03 | | | | | 9.80E-01 | | | | |  |
| rs16851483 | | | | | T | | | G | | -3.20E-03 | | | 1.00E-02 | | | | | 7.98E-01 | | | | |  |
| rs16903285 | | | | | C | | | T | | 1.60E-03 | | | 7.00E-03 | | | | | 7.20E-01 | | | | |  |
| rs16942887 | | | | | A | | | G | | 8.31E-02 | | | 5.10E-03 | | | | | 8.28E-54 | | | | |  |
| rs17014375 | | | | | G | | | T | | -8.20E-03 | | | 7.20E-03 | | | | | 6.85E-01 | | | | |  |
| rs17033117 | | | | | T | | | C | | -9.30E-03 | | | 6.20E-03 | | | | | 3.93E-01 | | | | |  |
| rs17056301 | | | | | C | | | T | | -3.40E-03 | | | 5.50E-03 | | | | | 6.31E-01 | | | | |  |
| rs17119937 | | | | | C | | | T | | -1.44E-02 | | | 1.07E-02 | | | | | 2.04E-01 | | | | |  |
| rs17184382 | | | | | C | | | A | | 9.00E-03 | | | 3.60E-03 | | | | | 1.80E-02 | | | | |  |
| rs17207196 | | | | | T | | | C | | 9.30E-03 | | | 5.60E-03 | | | | | 7.91E-02 | | | | |  |
| rs1730862 | | | | | A | | | G | | 7.30E-03 | | | 5.10E-03 | | | | | 6.80E-02 | | | | |  |
| rs17399237 | | | | | C | | | T | | 1.20E-03 | | | 4.80E-03 | | | | | 6.19E-01 | | | | |  |
| rs17425707 | | | | | C | | | T | | -9.00E-04 | | | 8.30E-03 | | | | | 8.04E-01 | | | | |  |
| rs17535749 | | | | | A | | | G | | -1.02E-02 | | | 5.90E-03 | | | | | 6.89E-02 | | | | |  |
| rs17551974 | | | | | A | | | C | | -8.10E-03 | | | 6.20E-03 | | | | | 3.38E-01 | | | | |  |
| rs1772189 | | | | | A | | | T | | 4.10E-03 | | | 4.80E-03 | | | | | 3.17E-01 | | | | |  |
| rs17789218 | | | | | C | | | T | | 4.10E-03 | | | 4.00E-03 | | | | | 1.35E-01 | | | | |  |
| rs1801282 | | | | | G | | | C | | 1.20E-02 | | | 5.10E-03 | | | | | 7.68E-03 | | | | |  |
| rs1863652 | | | | | A | | | G | | -8.00E-04 | | | 5.00E-03 | | | | | 6.35E-01 | | | | |  |
| rs1866956 | | | | | T | | | C | | 2.17E-02 | | | 3.70E-03 | | | | | 7.96E-10 | | | | |  |
| rs1883025 | | | | | T | | | C | | -6.98E-02 | | | 4.10E-03 | | | | | 1.50E-65 | | | | |  |
| rs1891216 | | | | | G | | | T | | -4.20E-03 | | | 5.30E-03 | | | | | 3.05E-01 | | | | |  |
| rs189595752 | | | | | G | | | A | | 7.50E-03 | | | 7.90E-03 | | | | | 3.55E-01 | | | | |  |
| rs1928295 | | | | | C | | | T | | 2.00E-03 | | | 3.40E-03 | | | | | 3.96E-01 | | | | |  |
| rs1982725 | | | | | T | | | C | | -4.90E-03 | | | 4.90E-03 | | | | | 2.50E-01 | | | | |  |
| rs200810 | | | | | C | | | T | | 1.20E-03 | | | 3.50E-03 | | | | | 7.23E-01 | | | | |  |
| rs2009416 | | | | | T | | | C | | 1.15E-02 | | | 5.20E-03 | | | | | 7.31E-02 | | | | |  |
| rs2065418 | | | | | G | | | T | | 3.10E-03 | | | 5.00E-03 | | | | | 7.50E-01 | | | | |  |
| rs215634 | | | | | G | | | A | | 8.00E-04 | | | 4.90E-03 | | | | | 6.72E-01 | | | | |  |
| rs2174307 | | | | | C | | | G | | 4.20E-03 | | | 4.80E-03 | | | | | 4.18E-01 | | | | |  |
| rs217671 | | | | | G | | | A | | 2.00E-04 | | | 5.60E-03 | | | | | 8.98E-01 | | | | |  |
| rs2224585 | | | | | A | | | G | | 1.27E-02 | | | 5.70E-03 | | | | | 1.38E-02 | | | | |  |
| rs2241210 | | | | | G | | | A | | 3.32E-02 | | | 3.50E-03 | | | | | 2.49E-20 | | | | |  |
| rs2246012 | | | | | C | | | T | | -1.53E-02 | | | 4.50E-03 | | | | | 1.08E-03 | | | | |  |
| rs2325036 | | | | | C | | | A | | 7.50E-03 | | | 3.50E-03 | | | | | 1.11E-01 | | | | |  |
| rs2351958 | | | | | A | | | C | | -6.30E-03 | | | 5.00E-03 | | | | | 1.46E-01 | | | | |  |
| rs2357760 | | | | | A | | | G | | -5.00E-04 | | | 3.60E-03 | | | | | 7.21E-01 | | | | |  |
| rs2393791 | | | | | T | | | C | | -1.24E-02 | | | 3.50E-03 | | | | | 7.17E-04 | | | | |  |
| rs2479958 | | | | | G | | | A | | 8.60E-03 | | | 5.50E-03 | | | | | 4.51E-01 | | | | |  |
| rs2481665 | | | | | C | | | T | | -3.20E-03 | | | 3.50E-03 | | | | | 7.60E-01 | | | | |  |
| rs2498786 | | | | | G | | | C | | -1.97E-02 | | | 3.60E-03 | | | | | 9.57E-09 | | | | |  |
| rs2543132 | | | | | C | | | G | | -1.09E-02 | | | 6.20E-03 | | | | | 1.51E-01 | | | | |  |
| rs2551644 | | | | | A | | | T | | -1.29E-02 | | | 6.10E-03 | | | | | 4.31E-02 | | | | |  |
| rs2608703 | | | | | A | | | C | | -1.00E-04 | | | 4.80E-03 | | | | | 9.32E-01 | | | | |  |
| rs2612038 | | | | | T | | | C | | -8.20E-03 | | | 5.80E-03 | | | | | 2.04E-01 | | | | |  |
| rs2642438 | | | | | G | | | A | | 3.03E-02 | | | 3.90E-03 | | | | | 7.78E-14 | | | | |  |
| rs2643452 | | | | | A | | | T | | 0.00E+00 | | | 4.80E-03 | | | | | 6.35E-01 | | | | |  |
| rs2693826 | | | | | A | | | G | | 7.90E-03 | | | 4.90E-03 | | | | | 2.56E-01 | | | | |  |
| rs2694047 | | | | | G | | | A | | 6.80E-03 | | | 5.60E-03 | | | | | 2.16E-01 | | | | |  |
| rs273504 | | | | | G | | | A | | 2.50E-03 | | | 5.30E-03 | | | | | 6.66E-01 | | | | |  |
| rs2744974 | | | | | T | | | C | | -2.42E-02 | | | 3.70E-03 | | | | | 3.25E-11 | | | | |  |
| rs2791644 | | | | | C | | | T | | 5.60E-03 | | | 5.40E-03 | | | | | 2.59E-01 | | | | |  |
| rs28507491 | | | | | A | | | G | | 4.30E-03 | | | 4.90E-03 | | | | | 2.43E-01 | | | | |  |
| rs2861683 | | | | | C | | | A | | -8.10E-03 | | | 4.80E-03 | | | | | 8.81E-02 | | | | |  |
| rs2868975 | | | | | A | | | G | | -7.20E-03 | | | 6.30E-03 | | | | | 3.45E-01 | | | | |  |
| rs2875762 | | | | | C | | | G | | -6.00E-04 | | | 5.80E-03 | | | | | 7.18E-01 | | | | |  |
| rs2931434 | | | | | T | | | C | | 3.50E-03 | | | 5.00E-03 | | | | | 4.78E-01 | | | | |  |
| rs2972145 | | | | | C | | | T | | -3.23E-02 | | | 5.00E-03 | | | | | 1.81E-09 | | | | |  |
| rs3001032 | | | | | C | | | T | | 1.48E-02 | | | 5.30E-03 | | | | | 8.14E-03 | | | | |  |
| rs34255979 | | | | | T | | | C | | -2.44E-02 | | | 8.10E-03 | | | | | 5.00E-03 | | | | |  |
| rs349088 | | | | | A | | | C | | 5.60E-03 | | | 4.90E-03 | | | | | 4.34E-01 | | | | |  |
| rs35350976 | | | | | G | | | A | | 1.10E-03 | | | 6.50E-03 | | | | | 9.55E-01 | | | | |  |
| rs355777 | | | | | C | | | G | | -4.60E-03 | | | 4.80E-03 | | | | | 4.17E-01 | | | | |  |
| rs3749897 | | | | | T | | | C | | -1.00E-03 | | | 4.80E-03 | | | | | 6.47E-01 | | | | |  |
| rs3754963 | | | | | T | | | A | | -2.40E-03 | | | 5.40E-03 | | | | | 7.65E-01 | | | | |  |
| rs3772882 | | | | | A | | | C | | -1.18E-02 | | | 4.90E-03 | | | | | 1.32E-02 | | | | |  |
| rs3800229 | | | | | T | | | G | | -9.60E-03 | | | 3.70E-03 | | | | | 1.89E-02 | | | | |  |
| rs3806114 | | | | | A | | | G | | -2.40E-03 | | | 4.00E-03 | | | | | 4.89E-01 | | | | |  |
| rs3806572 | | | | | A | | | G | | -5.60E-03 | | | 5.30E-03 | | | | | 3.08E-01 | | | | |  |
| rs3807645 | | | | | A | | | G | | 8.90E-03 | | | 6.00E-03 | | | | | 2.67E-01 | | | | |  |
| rs380857 | | | | | A | | | C | | 1.02E-02 | | | 8.70E-03 | | | | | 1.52E-01 | | | | |  |
| rs3810027 | | | | | G | | | C | | -4.20E-03 | | | 5.20E-03 | | | | | 2.90E-01 | | | | |  |
| rs3829639 | | | | | G | | | A | | -6.90E-03 | | | 5.30E-03 | | | | | 1.26E-01 | | | | |  |
| rs38314 | | | | | A | | | G | | 8.40E-03 | | | 4.90E-03 | | | | | 2.87E-01 | | | | |  |
| rs3902951 | | | | | G | | | T | | 3.10E-03 | | | 4.20E-03 | | | | | 7.23E-01 | | | | |  |
| rs3904244 | | | | | A | | | T | | -1.05E-02 | | | 6.70E-03 | | | | | 2.97E-01 | | | | |  |
| rs40067 | | | | | A | | | G | | 8.60E-03 | | | 6.70E-03 | | | | | 6.62E-02 | | | | |  |
| rs40270 | | | | | C | | | A | | -2.35E-02 | | | 3.90E-03 | | | | | 8.10E-09 | | | | |  |
| rs4072917 | | | | | A | | | G | | -2.30E-03 | | | 5.10E-03 | | | | | 5.51E-01 | | | | |  |
| rs4148005 | | | | | G | | | T | | -2.83E-02 | | | 3.60E-03 | | | | | 5.74E-14 | | | | |  |
| rs4148155 | | | | | G | | | A | | -1.23E-02 | | | 5.60E-03 | | | | | 2.56E-02 | | | | |  |
| rs4237643 | | | | | G | | | T | | 1.60E-03 | | | 5.10E-03 | | | | | 6.70E-01 | | | | |  |
| rs4240624 | | | | | A | | | G | | 8.18E-02 | | | 5.80E-03 | | | | | 1.32E-45 | | | | |  |
| rs427943 | | | | | C | | | A | | -9.70E-03 | | | 4.20E-03 | | | | | 1.48E-02 | | | | |  |
| rs4307239 | | | | | G | | | A | | -5.40E-03 | | | 4.90E-03 | | | | | 2.96E-01 | | | | |  |
| rs4338849 | | | | | A | | | G | | -1.13E-02 | | | 4.80E-03 | | | | | 3.19E-02 | | | | |  |
| rs4430672 | | | | | C | | | T | | 9.80E-03 | | | 5.90E-03 | | | | | 2.40E-01 | | | | |  |
| rs4556997 | | | | | A | | | C | | -2.40E-03 | | | 4.90E-03 | | | | | 6.73E-01 | | | | |  |
| rs4567095 | | | | | T | | | C | | -9.00E-04 | | | 3.90E-03 | | | | | 8.80E-01 | | | | |  |
| rs4568281 | | | | | A | | | G | | 1.68E-02 | | | 5.00E-03 | | | | | 3.18E-04 | | | | |  |
| rs4589691 | | | | | G | | | C | | -4.00E-03 | | | 6.60E-03 | | | | | 5.07E-01 | | | | |  |
| rs4639527 | | | | | G | | | A | | -6.80E-03 | | | 5.30E-03 | | | | | 2.19E-01 | | | | |  |
| rs4639796 | | | | | A | | | G | | 1.75E-02 | | | 6.30E-03 | | | | | 3.27E-03 | | | | |  |
| rs4660443 | | | | | T | | | C | | -3.63E-02 | | | 6.00E-03 | | | | | 2.53E-09 | | | | |  |
| rs4665972 | | | | | C | | | T | | 1.10E-02 | | | 3.50E-03 | | | | | 2.67E-03 | | | | |  |
| rs4671328 | | | | | G | | | T | | 8.40E-03 | | | 4.80E-03 | | | | | 3.31E-02 | | | | |  |
| rs4674669 | | | | | T | | | C | | -6.10E-03 | | | 7.20E-03 | | | | | 2.53E-01 | | | | |  |
| rs4740619 | | | | | C | | | T | | 4.90E-03 | | | 3.40E-03 | | | | | 1.08E-01 | | | | |  |
| rs4800191 | | | | | C | | | G | | -7.60E-03 | | | 3.50E-03 | | | | | 3.73E-02 | | | | |  |
| rs4804414 | | | | | T | | | C | | -8.70E-03 | | | 4.80E-03 | | | | | 3.62E-02 | | | | |  |
| rs4812336 | | | | | A | | | G | | -3.80E-03 | | | 5.10E-03 | | | | | 5.24E-01 | | | | |  |
| rs4813619 | | | | | T | | | G | | 8.30E-03 | | | 4.80E-03 | | | | | 8.35E-02 | | | | |  |
| rs4820091 | | | | | G | | | T | | -3.15E-02 | | | 5.90E-03 | | | | | 7.45E-08 | | | | |  |
| rs4820408 | | | | | G | | | T | | 1.20E-03 | | | 3.50E-03 | | | | | 7.33E-01 | | | | |  |
| rs4842491 | | | | | T | | | C | | -8.00E-04 | | | 3.80E-03 | | | | | 8.66E-01 | | | | |  |
| rs4846914 | | | | | A | | | G | | 4.79E-02 | | | 3.40E-03 | | | | | 3.51E-41 | | | | |  |
| rs4851029 | | | | | G | | | T | | -2.00E-03 | | | 5.00E-03 | | | | | 9.51E-01 | | | | |  |
| rs4880341 | | | | | T | | | C | | 4.00E-04 | | | 4.80E-03 | | | | | 7.89E-01 | | | | |  |
| rs4936175 | | | | | C | | | T | | -7.60E-03 | | | 4.80E-03 | | | | | 3.36E-02 | | | | |  |
| rs4939883 | | | | | C | | | T | | 7.99E-02 | | | 4.50E-03 | | | | | 1.80E-66 | | | | |  |
| rs4954638 | | | | | C | | | A | | 5.00E-03 | | | 5.30E-03 | | | | | 5.57E-01 | | | | |  |
| rs538579 | | | | | C | | | G | | -4.80E-03 | | | 5.00E-03 | | | | | 2.91E-01 | | | | |  |
| rs543874 | | | | | G | | | A | | -1.09E-02 | | | 4.30E-03 | | | | | 1.79E-02 | | | | |  |
| rs559231 | | | | | T | | | G | | 2.20E-03 | | | 5.00E-03 | | | | | 6.88E-01 | | | | |  |
| rs56024084 | | | | | T | | | C | | 8.00E-04 | | | 4.90E-03 | | | | | 6.65E-01 | | | | |  |
| rs56077345 | | | | | C | | | G | | -2.00E-02 | | | 6.80E-03 | | | | | 7.37E-03 | | | | |  |
| rs587252 | | | | | C | | | A | | -1.59E-02 | | | 1.05E-02 | | | | | 4.29E-01 | | | | |  |
| rs61856602 | | | | | G | | | A | | 8.40E-03 | | | 5.20E-03 | | | | | 7.96E-02 | | | | |  |
| rs6235 | | | | | G | | | C | | -1.30E-03 | | | 5.40E-03 | | | | | 6.39E-01 | | | | |  |
| rs62623385 | | | | | T | | | A | | -9.40E-03 | | | 1.24E-02 | | | | | 5.87E-01 | | | | |  |
| rs6265 | | | | | T | | | C | | 8.30E-03 | | | 4.30E-03 | | | | | 7.25E-02 | | | | |  |
| rs633695 | | | | | G | | | A | | 8.85E-02 | | | 5.40E-03 | | | | | 7.82E-58 | | | | |  |
| rs6471941 | | | | | A | | | G | | -1.70E-03 | | | 4.20E-03 | | | | | 7.31E-01 | | | | |  |
| rs6486122 | | | | | T | | | C | | -1.56E-02 | | | 5.10E-03 | | | | | 4.14E-03 | | | | |  |
| rs6512302 | | | | | C | | | G | | 7.00E-03 | | | 6.30E-03 | | | | | 2.60E-01 | | | | |  |
| rs6545714 | | | | | A | | | G | | 8.70E-03 | | | 3.40E-03 | | | | | 1.75E-02 | | | | |  |
| rs6561943 | | | | | T | | | C | | -9.30E-03 | | | 4.00E-03 | | | | | 2.30E-02 | | | | |  |
| rs657452 | | | | | G | | | A | | 1.00E-02 | | | 3.70E-03 | | | | | 1.34E-02 | | | | |  |
| rs6591407 | | | | | A | | | C | | 7.40E-03 | | | 4.40E-03 | | | | | 5.58E-02 | | | | |  |
| rs6595205 | | | | | G | | | C | | 4.60E-03 | | | 3.40E-03 | | | | | 1.24E-01 | | | | |  |
| rs663129 | | | | | A | | | G | | -2.54E-02 | | | 4.10E-03 | | | | | 5.54E-09 | | | | |  |
| rs668871 | | | | | T | | | C | | 3.00E-03 | | | 4.80E-03 | | | | | 5.13E-01 | | | | |  |
| rs6758199 | | | | | T | | | C | | -8.30E-03 | | | 9.00E-03 | | | | | 7.09E-01 | | | | |  |
| rs6785245 | | | | | C | | | T | | -1.00E-03 | | | 4.80E-03 | | | | | 5.14E-01 | | | | |  |
| rs6815910 | | | | | A | | | T | | -2.60E-03 | | | 4.80E-03 | | | | | 6.49E-01 | | | | |  |
| rs6841761 | | | | | T | | | G | | 5.20E-03 | | | 3.40E-03 | | | | | 8.66E-02 | | | | |  |
| rs6860245 | | | | | C | | | G | | 1.17E-02 | | | 5.60E-03 | | | | | 1.23E-02 | | | | |  |
| rs686030 | | | | | A | | | C | | 5.50E-02 | | | 4.90E-03 | | | | | 4.29E-27 | | | | |  |
| rs695272 | | | | | C | | | T | | -9.40E-03 | | | 5.30E-03 | | | | | 8.73E-02 | | | | |  |
| rs7015 | | | | | A | | | G | | -1.20E-03 | | | 4.40E-03 | | | | | 8.49E-01 | | | | |  |
| rs7025938 | | | | | G | | | C | | 3.00E-03 | | | 5.00E-03 | | | | | 9.79E-01 | | | | |  |
| rs705704 | | | | | A | | | G | | 8.30E-03 | | | 3.90E-03 | | | | | 2.91E-02 | | | | |  |
| rs7084454 | | | | | A | | | G | | -8.10E-03 | | | 5.30E-03 | | | | | 1.15E-01 | | | | |  |
| rs7096764 | | | | | A | | | G | | 6.60E-03 | | | 5.20E-03 | | | | | 3.33E-01 | | | | |  |
| rs7117238 | | | | | A | | | G | | -1.50E-03 | | | 4.60E-03 | | | | | 7.91E-01 | | | | |  |
| rs7138803 | | | | | A | | | G | | -1.22E-02 | | | 3.50E-03 | | | | | 5.54E-04 | | | | |  |
| rs7144011 | | | | | T | | | G | | -3.90E-03 | | | 4.10E-03 | | | | | 2.27E-01 | | | | |  |
| rs7148846 | | | | | G | | | T | | -3.60E-03 | | | 5.90E-03 | | | | | 4.28E-01 | | | | |  |
| rs7196720 | | | | | C | | | T | | -1.90E-03 | | | 4.90E-03 | | | | | 6.28E-01 | | | | |  |
| rs7222349 | | | | | A | | | G | | 9.00E-03 | | | 5.10E-03 | | | | | 3.86E-02 | | | | |  |
| rs7239575 | | | | | C | | | T | | 1.37E-02 | | | 4.80E-03 | | | | | 1.76E-02 | | | | |  |
| rs7262150 | | | | | C | | | T | | 1.26E-02 | | | 5.80E-03 | | | | | 6.37E-02 | | | | |  |
| rs73079476 | | | | | C | | | A | | 4.30E-03 | | | 6.40E-03 | | | | | 5.81E-01 | | | | |  |
| rs7314285 | | | | | G | | | T | | -6.70E-03 | | | 7.70E-03 | | | | | 2.11E-01 | | | | |  |
| rs7318817 | | | | | T | | | C | | -7.00E-04 | | | 4.90E-03 | | | | | 9.52E-01 | | | | |  |
| rs738409 | | | | | G | | | C | | -1.57E-02 | | | 5.80E-03 | | | | | 9.80E-03 | | | | |  |
| rs7451021 | | | | | C | | | T | | -1.48E-02 | | | 5.10E-03 | | | | | 9.15E-03 | | | | |  |
| rs7498665 | | | | | G | | | A | | -1.17E-02 | | | 3.60E-03 | | | | | 1.02E-03 | | | | |  |
| rs7557796 | | | | | C | | | T | | 2.90E-03 | | | 5.00E-03 | | | | | 7.04E-01 | | | | |  |
| rs757869 | | | | | G | | | A | | 6.00E-04 | | | 5.50E-03 | | | | | 8.72E-01 | | | | |  |
| rs7615297 | | | | | G | | | C | | 7.00E-03 | | | 6.70E-03 | | | | | 4.81E-01 | | | | |  |
| rs7626079 | | | | | T | | | C | | 6.50E-03 | | | 5.00E-03 | | | | | 1.48E-01 | | | | |  |
| rs7678138 | | | | | A | | | G | | -1.70E-02 | | | 7.30E-03 | | | | | 5.75E-03 | | | | |  |
| rs7683836 | | | | | A | | | G | | 6.30E-03 | | | 4.90E-03 | | | | | 4.24E-01 | | | | |  |
| rs7703576 | | | | | C | | | T | | -6.90E-03 | | | 5.20E-03 | | | | | 1.00E-01 | | | | |  |
| rs7715256 | | | | | T | | | G | | 2.20E-03 | | | 3.40E-03 | | | | | 5.36E-01 | | | | |  |
| rs7724675 | | | | | A | | | G | | -1.40E-03 | | | 5.70E-03 | | | | | 6.99E-01 | | | | |  |
| rs7730898 | | | | | A | | | G | | -1.88E-02 | | | 3.80E-03 | | | | | 1.63E-05 | | | | |  |
| rs7761673 | | | | | A | | | T | | 6.80E-03 | | | 6.20E-03 | | | | | 3.64E-01 | | | | |  |
| rs7780752 | | | | | C | | | T | | -2.80E-03 | | | 5.00E-03 | | | | | 3.74E-01 | | | | |  |
| rs7788008 | | | | | A | | | G | | 1.21E-02 | | | 4.80E-03 | | | | | 5.79E-02 | | | | |  |
| rs7819514 | | | | | A | | | G | | 3.20E-03 | | | 5.20E-03 | | | | | 3.45E-01 | | | | |  |
| rs7844647 | | | | | C | | | T | | 1.28E-02 | | | 3.80E-03 | | | | | 1.27E-03 | | | | |  |
| rs7860634 | | | | | A | | | G | | 2.90E-03 | | | 5.10E-03 | | | | | 5.96E-01 | | | | |  |
| rs7869771 | | | | | C | | | A | | 4.00E-04 | | | 5.50E-03 | | | | | 9.12E-01 | | | | |  |
| rs7925214 | | | | | T | | | C | | 5.00E-04 | | | 4.90E-03 | | | | | 9.26E-01 | | | | |  |
| rs796004 | | | | | T | | | C | | 1.74E-02 | | | 5.30E-03 | | | | | 2.43E-03 | | | | |  |
| rs79717793 | | | | | A | | | G | | -1.09E-02 | | | 6.60E-03 | | | | | 9.30E-02 | | | | |  |
| rs7983065 | | | | | T | | | C | | -1.40E-03 | | | 4.80E-03 | | | | | 8.80E-01 | | | | |  |
| rs802685 | | | | | C | | | T | | 8.80E-03 | | | 5.50E-03 | | | | | 9.11E-02 | | | | |  |
| rs8033077 | | | | | C | | | T | | 6.20E-03 | | | 5.40E-03 | | | | | 1.85E-01 | | | | |  |
| rs8047395 | | | | | A | | | G | | -1.87E-02 | | | 3.50E-03 | | | | | 2.88E-08 | | | | |  |
| rs806600 | | | | | G | | | A | | 1.24E-02 | | | 4.80E-03 | | | | | 1.92E-02 | | | | |  |
| rs8079418 | | | | | C | | | T | | 1.00E-02 | | | 4.80E-03 | | | | | 7.86E-02 | | | | |  |
| rs8097672 | | | | | T | | | A | | -1.03E-02 | | | 7.10E-03 | | | | | 1.02E-01 | | | | |  |
| rs8097783 | | | | | A | | | G | | 1.73E-02 | | | 6.70E-03 | | | | | 2.65E-02 | | | | |  |
| rs8181823 | | | | | C | | | A | | -4.90E-03 | | | 5.80E-03 | | | | | 2.69E-01 | | | | |  |
| rs865809 | | | | | G | | | A | | 5.20E-03 | | | 5.70E-03 | | | | | 2.91E-01 | | | | |  |
| rs872281 | | | | | T | | | C | | 4.90E-03 | | | 6.40E-03 | | | | | 4.12E-01 | | | | |  |
| rs876605 | | | | | G | | | A | | 1.04E-02 | | | 5.50E-03 | | | | | 7.29E-02 | | | | |  |
| rs879620 | | | | | T | | | C | | -9.40E-03 | | | 5.50E-03 | | | | | 3.00E-01 | | | | |  |
| rs889398 | | | | | T | | | C | | 1.13E-02 | | | 3.40E-03 | | | | | 4.77E-03 | | | | |  |
| rs901630 | | | | | T | | | C | | 7.20E-03 | | | 3.50E-03 | | | | | 4.81E-02 | | | | |  |
| rs902695 | | | | | A | | | G | | -1.17E-02 | | | 4.80E-03 | | | | | 1.02E-02 | | | | |  |
| rs9267551 | | | | | G | | | C | | -6.00E-03 | | | 7.00E-03 | | | | | 5.54E-01 | | | | |  |
| rs9294260 | | | | | A | | | G | | 9.00E-04 | | | 3.40E-03 | | | | | 8.25E-01 | | | | |  |
| rs9297994 | | | | | A | | | G | | -3.50E-03 | | | 5.00E-03 | | | | | 4.34E-01 | | | | |  |
| rs9300422 | | | | | G | | | A | | -3.50E-03 | | | 3.70E-03 | | | | | 3.99E-01 | | | | |  |
| rs9379084 | | | | | A | | | G | | 1.33E-02 | | | 6.80E-03 | | | | | 7.90E-02 | | | | |  |
| rs9408882 | | | | | A | | | G | | 3.00E-03 | | | 3.40E-03 | | | | | 4.08E-01 | | | | |  |
| rs946824 | | | | | C | | | T | | 2.60E-03 | | | 7.20E-03 | | | | | 4.19E-01 | | | | |  |
| rs947612 | | | | | A | | | G | | -8.90E-03 | | | 5.50E-03 | | | | | 6.66E-02 | | | | |  |
| rs9522285 | | | | | A | | | G | | -9.70E-03 | | | 4.90E-03 | | | | | 7.54E-02 | | | | |  |
| rs9538162 | | | | | C | | | T | | -9.30E-03 | | | 4.80E-03 | | | | | 2.03E-01 | | | | |  |
| rs9547153 | | | | | G | | | A | | -7.20E-03 | | | 3.50E-03 | | | | | 4.13E-02 | | | | |  |
| rs9571687 | | | | | A | | | C | | 1.18E-02 | | | 5.10E-03 | | | | | 2.26E-02 | | | | |  |
| rs9615905 | | | | | T | | | C | | -8.30E-03 | | | 5.10E-03 | | | | | 2.90E-01 | | | | |  |
| rs964184 | | | | | C | | | G | | 1.07E-01 | | | 7.10E-03 | | | | | 6.09E-48 | | | | |  |
| rs970548 | | | | | C | | | A | | 2.58E-02 | | | 3.90E-03 | | | | | 1.71E-10 | | | | |  |
| rs9739640 | | | | | G | | | A | | -1.41E-02 | | | 6.30E-03 | | | | | 3.58E-02 | | | | |  |
| rs977747 | | | | | G | | | T | | 4.60E-03 | | | 3.40E-03 | | | | | 2.11E-01 | | | | |  |
| rs9783858 | | | | | T | | | C | | 3.30E-03 | | | 3.50E-03 | | | | | 3.07E-01 | | | | |  |
| rs9806742 | | | | | A | | | G | | 6.30E-03 | | | 7.60E-03 | | | | | 5.91E-01 | | | | |  |
| rs9816226 | | | | | T | | | A | | 1.49E-02 | | | 4.50E-03 | | | | | 1.12E-03 | | | | |  |
| rs9845966 | | | | | G | | | T | | 2.70E-03 | | | 3.60E-03 | | | | | 3.42E-01 | | | | |  |
| rs9849171 | | | | | C | | | G | | 9.00E-04 | | | 5.00E-03 | | | | | 9.45E-01 | | | | |  |
| rs987237 | | | | | G | | | A | | 1.70E-03 | | | 4.40E-03 | | | | | 6.98E-01 | | | | |  |
| rs9927848 | | | | | A | | | C | | 6.00E-04 | | | 5.50E-03 | | | | | 7.98E-01 | | | | |  |
| rs9989419 | | | | | G | | | A | | 1.47E-01 | | | 3.60E-03 | | | | | 1.00E-200 | | | | |  |
| rs999889 | | | | | A | | | G | | 4.70E-03 | | | 5.30E-03 | | | | | 1.71E-01 | | | | |  |

SNP, single nucleotide polymorphism; se, standard error; IV, instrumental variable; MR, mendelian randomization, CES, cardioembolic stroke; BMI, body mass index; HDL-C, high-density lipoprotein cholesterol; DHEAL, dehydroepiandrosterone sulfate levels, MVMR, multivariable Mendelian randomization
